# NADPH Oxidase 5 Induces Changes in the Unfolded Protein Response in Human Aortic Endothelial Cells and in Endothelial-Specific *Knock-in* Mice

**DOI:** 10.3390/antiox10020194

**Published:** 2021-01-29

**Authors:** Adriana Cortés, Álvaro Pejenaute, Javier Marqués, Íñigo Izal, Silvia Cenoz, Eduardo Ansorena, Juan José Martínez-Irujo, Carlos de Miguel, Guillermo Zalba

**Affiliations:** 1 Department of Biochemistry and Genetics, University of Navarra, 31008 Pamplona, Spain; acortes.3@alumni.unav.es (A.C.); apejenaute@alumni.unav.es (Á.P.); jmarquesc@unav.es (J.M.); inizal@unav.es (Í.I.); scenoz@uanv.es (S.C.); eansorena@unav.es (E.A.); jjmirujo@unav.es (J.J.M.-I.); cdmiguel@unav.es (C.d.M.); 2 Navarra Institute for Health Research (IdiSNA), 31008 Pamplona, Spain

**Keywords:** oxidative stress, NADPH oxidase 5, unfolded protein response, chronic infarction, endothelial cells

## Abstract

Oxidative stress constitutes a key molecular mechanism in the development of cardiovascular diseases. A potential relationship between reactive oxygen species (ROS) driven by the NADPH oxidase family (NOX) and the unfolded protein response (UPR) has been postulated. Nevertheless, there is a lack of information about the crosstalk between NOX5 homologue and the UPR in a cardiovascular context. The main aim was to analyze NOX5-mediated ROS effects in the UPR and its importance in cardiovascular diseases. To this effect, we used an adenoviral NOX5-β overexpression model in human aortic endothelial cells (HAEC) and a conditional endothelial NOX5 *knock-in* mouse. Using expression arrays, we investigated NOX5-induced genomic changes in HAEC. Compared with the control HAEC, 298 genes were differentially expressed. Gene ontology analysis revealed the activation of numerous cellular routes, the most relevant being the UPR pathway. Using real-time PCR and Western Blot experiments, we confirmed that NOX5 overexpression induced changes in the expression of the UPR components, which were associated with increased apoptosis. Moreover, in endothelial-specific NOX5 *knock-in* mice, we found changes in the expression of the UPR components genes. In these mice, myocardial infarction was performed by permanent coronary artery ligation; however, NOX5 expression was not associated with differences in the UPR components mRNA levels. In these animals, we found significant associations between the UPR components gene expression and echocardiographic parameters. Our data support the idea that NOX5-derived ROS may modulate the UPR pathway in endothelial cells, which might play a relevant role in cardiac physiology.

## 1. Introduction

Cardiovascular diseases (CVDs) are the leading cause of death in the developed world, accounting for a 44% of non-communicable disease (non-transmissible diseases) deaths [1]. The annual number of deaths caused by CVDs will rise up to 25 million in 2030 [2]. Oxidative stress is an underlying mechanism of these diseases and their risk factors. Increased reactive oxygen species (ROS) production contributes to many aspects of pathological progression [3].

High production of ROS and attenuation of endogenous antioxidants promote an oxidative stress environment that precedes CVDs [4]. ROS can oxidize proteins, DNA and other cellular components causing cell damage [5]. All cellular types of the cardiovascular system produce ROS under physiological conditions, including endothelial cells, cardiomyocytes, smooth muscle cells and fibroblasts [6]. Their production also increases under pathological conditions [7].

Several enzymatic systems are able to generate ROS in the vascular wall, including the NADPH oxidase family (NOX) [8]. NOX are involved in redox signaling and produce ROS at specific subcellular locations, including the plasma membrane, the endoplasmic reticulum (ER) and the nucleus [9]. ROS generated by the NOX family also participate in pathophysiological mechanisms associated with vascular diseases, including endothelial dysfunction [10], media hypertrophy [11], adhesion molecule receptors expression and metalloprotease-induced vascular remodeling [9].

Within the NOX family, NOX5 is the last described homologue, and its role in CVDs has not been completely characterized. There is no optimal in vivo model for the study of NOX5 due to its evolutionary loss in rodents [9,10]. Previous works have shown that NOX5 is found at the ER of vascular endothelial cells [7,12]. Its regulation is different to other NOX homologues since it does not present cytoplasmic subunits that could modulate its catalytic activity [13]. NOX5 contains “EF-hand” domains that undergo conformational changes in the presence of Ca^2+^, promoting interaction with the catalytic domain and inducing superoxide production [14]. NOX5 presents five different isoforms associated to alternative splicing [11], and NOX5-β is the most relevant one in vessels [9,10]. NOX5 expression is altered in CVDs. For instance, NOX5 is overexpressed in cardiac cells after acute myocardial infarction [7], and it is progressively overexpressed in the vascular wall including endothelial cells during atheroma plaque formation [15]. In addition, NOX5 expression is higher in the glomeruli and mesangial cells of patients who suffer from diabetes, suggesting a potential role for this oxidase in diabetic nephropathy [16]. More studies will be necessary to better characterize NOX5 implications in cardiovascular pathophysiology.

Oxidative stress produced by NOX alters protein homeostasis [17] and is involved in the development of ER stress, triggering the unfolded protein response (UPR) [18,19]. Recent studies postulate NOX as relevant mediators of ER stress that leads to the UPR [20]. NOX4 was shown to interact physically with UPR components as protein disulfide isomerase (PDI) and, otherwise, UPR activation can induce NOX4 expression and ROS production [21]. Additionally, NOX4 specifically oxidizes the ER stress sensor of the endoplasmic reticulum to nucleus signaling 1 alpha (IRE1α), which triggers phosphorylation and activation of the X-box binding protein 1 (XBP1), leading to proteasome activation or apoptosis induction [22]. Additionally, NOX2 and NOX4 increase their activity under ER stress conditions [23]. Finally, NOX5 might be involved in ER stress in an acute model of peripheral vascular disease [24]. We hypothesize that NOX5 may also play a key role in CVDs progression by promoting UPR activation in vascular endothelial cells.

## 2. Materials and Methods

### 2.1. Cell Culture

Primary human aortic endothelial cells (HAEC) were obtained from American Type Culture Collection (ATCC, Manassas, VA, USA). Vascular Cell Basal Complete Medium (ATCC, Manassas, VA, USA) containing 2% of heat-inactivated fetal bovine serum (FBS, ATCC, Manassas, VA, USA), 0.5% of streptomycin-penicillin (ATTC, Manassas, VA, USA), 0.5% of Gentamicin/Amphoterin-B (ATCC, Manassas, VA, USA), 0.5% of Phenol Red (ATCC, Manassas, VA, USA) and an Endothelial Cell Growth Kit (ATCC, Manassas, VA, USA) was used for cell maintenance. Medium was replaced every 2–3 days, and cells were trypsinized before reaching confluence with Trypsin-EDTA (0.05%) phenol red (Gibco, Gaithersburg, MD, USA).

### 2.2. Adenovirus Transduction

An adenovirus encoding human NOX5-β construct was obtained using the AdEasy XL Adenoviral Vector System Kit (Agilent Technologies, Santa Clara, CA, USA), as previously reported [25]. Briefly, NOX5-β (accession number AF325189) was subcloned from an ORFEXPRESS shuttle clone (GC-E1558, GeneCopoeia, Rockville, MD, USA) into a pShuttle-CMV vector used to generate the NOX5-β adenovirus. The construction and production of the adenoviral vector carrying the green fluorescent protein (GFP) gene has been previously described [25]. HAEC were infected at 90% confluence by exposure to the NOX5-β adenovirus in infection medium (composed by Vascular Cell Basal Medium containing 2% heat-inactivated fetal bovine serum, L-glutamine 10 mM, ascorbic acid 50 μg/mL, 0.5% streptomycin-penicillin and 0.5% Gentamicin/Amphoterin-B) for 3 h at the indicated multiplicity of infection (MOI). After this time, medium was replaced with complete medium as required for each assay.

### 2.3. Transcriptome Analysis

High quality RNA, isolated from GFP- and NOX5-β-infected HAEC (MOI 100), was used to determine mRNA expression by a Human Gene 2.0 ST Array 2.0 (Affymetrix). Microarray data analysis consisted of background correction and normalization using the RMA algorithm [26]. A filtering process was performed in a R/Bioconductor [27] to eliminate low expression probe sets. Applying a criterion of expression value greater than 16 in more than 50% of the samples of at least one experimental condition, 38,692 probe sets were selected for statistical analysis. LIMMA (Linear Models for Microarray Data) [28] was used to find the probe sets with significant differential expression with a false discovery rate (FDR) cut off (FDR < 0.05) and a 1.5 folds change cut-off.

### 2.4. Real-Time Quantitative Polymerase Chain Reaction

RNA samples were extracted from in vitro culture cells and murine hearts using TRIzol (Thermo Scientific, Rockford, IL, USA). RNA concentration and purity were measured using a Nanodrop ND-1000 spectrophotometer (Thermo Scientific, Rockford, IL, USA). After that, 2 μg of RNA from each sample was reverse transcribed into cDNA using a SuperScript VILO cDNA Synthesis Kit (Thermo Scientific, Rockford, IL, USA). Real-time PCRs were performed using the iQ SYBR Green Supermix kit (Bio-Rad, Hercules, CA, USA) in an iQ5 Multicolor real-time PCR Detection System (Bio-Rad, Hercules, CA, USA). Glyceraldehyde 3-phosphate dehydrogenase (GAPDH) was chosen as the housekeeping gene to normalize gene expression. Measurements were done by triplicate to ensure data accuracy. Specific primers are presented in Table 1.

### 2.5. Western Blot Analysis

Protein extraction was performed with RIPA Buffer (1% NP-40, 150 mM NaCl, 50 mM Tris pH = 8, 0.1% SDS and 0.5% sodic deoxycholate). To avoid sample degradation, EDTA-free protease inhibitors (Roche, Basel, Switzerland) were added. Then, samples were sonicated to ensure membrane degradation. Also, 4x Laemmli buffer (Bio-Rad, Hercules, CA, USA) with 10% of β-mercaptoethanol was added to extracts and loaded into denaturing gels containing acrylamide (5–10%) and SDS (0.1%). After electrophoresis (70 min at constant 150 V), proteins were transferred to nitrocellulose membranes (2 h at constant 50 V) and blocked using Tris-Buffered saline with 5% milk for 1.5 h. Later, membranes were incubated with primary antibodies (overnight at 4 °C) and the pertinent secondary antibodies (1.5 h at room temperature) (Table 2). Proteins were visualized using the ECL Prime Western Blotting Detection Reagent (GE Healthcare Amersham, Chicago, IL, USA).

### 2.6. Reactive Oxygen Species Determination

Extracellular hydrogen peroxide (H_2_O_2_) production was measured using Amplex Red-based assay; Ampliflu Red (Sigma Aldrich, Saint Louis, MO, USA). The day before infection, 15,000 cells/well were seeded in 96-well plates in complete medium to ensure 90% cell confluence after overnight incubation. Then, cells were infected with GFP or NOX5-β adenovirus at the appropriate MOI (25, 50, 100 or 200). Cells were washed once with Krebs-Ringer solution, HEPES-buffered (Alfa Aesar, Thermo Fisher, Kandel, Germany) and, if needed, incubated for 1 h in 50 μL of Krebs-Ringer solution HEPES-buffered, containing 5, 10, 50 or 100 nM of NOX5 inhibitor ML090 [29]. Subsequently, up to 50 μM of Amplex Red reagent and 0.1 units/mL of HRP were added. The fluorescent intensity was measured after 30 min at 37 °C in a PolarStar fluorescence microplate reader (BMG Labtech, Ortenberg, Germany). Excitation and emission filters of 540 and 580 nm, respectively, were used. The increase in fluorescence (ΔFluorescence) was obtained by subtracting the initial fluorescent intensities from the final ones.

Dihydroethidium staining (DHE, Thermo Fisher) was performed in intact cells to identify intracellular superoxide anion production. Briefly, 100,000 cells in 12-well format plates were seeded and infected with the GFP or NOX5-β adenovirus at MOI 100. Then, cells were incubated for 1 h in 50 μL of Krebs-Ringer solution—HEPES-buffered, alone or containing 5–100 nM ML090—24 h after infection. Later, cells were incubated with 100 µM DHE for 10 min, and ZOE Fluorescent Cell Imager (Bio-Rad, Hercules, CA, USA) was used for image obtaining.

### 2.7. Caspase Activity Assay

Cell apoptosis was measured using the Caspase-Glo 3/7 assay kit (Promega, Madison, WI, USA) in 96-well plates in HAEC after infection with GFP or NOX5-β. Briefly, the day before infection, the 96-well plates were seeded with 10,000 cells/well in complete medium to ensure 90% cell confluence after overnight incubation. Then, cells were infected with GFP or NOX5-β adenoviruses at the appropriate MOI (25, 50, 100, 200 or 250), as described above. Measurements were performed on a LuminoskanTM Ascent plate luminometer (Thermo Scientific, Rockford, IL, USA) 24 h after infection.

### 2.8. Proteasome Activity Measurement

Proteasome activity was measured using the Proteasome Activity Assay Kit (Abnova, Walnut, CA, USA). 250,000 cells/well were seeded in 6-well plates in complete growth medium to ensure 90% cell confluence after overnight incubation. Then, cells were infected with GFP or NOX5-β adenovirus as before. Twelve hours and 24 h after infection, cells were collected in 0.5% Igepal (Sigma Aldrich, Saint Louis, MO, USA) in order to obtain the protein lysates from which the proteasome activity was measured. The activity was quantified in the TECAN Infinity F.200 fluorometer (Männedorf, Switzerland) at 37 °C with an excitation wavelength of 350 nm and an emission one of 440 nm.

### 2.9. Conditional Knock-in Mice

Conditional *knock-in* mice for the human *NOX5-β* gene were used in this study (NOX5^+/−^CRE^+/−^). C57BL/6 mice only expressed NOX5-β in endothelial cells after tamoxifen induction (40 mg/kg) during 3 non-consecutive days. This induced endothelial-specific CRE recombinase and transgenesis. As a control group, we used endothelial cell-specific CRE recombinase expressing mice (Cdh5(PAC)-CreERT2; CRE^+/−^) provided by Wang et al. [30]. For this study, 9 males/group were used (CRE^+/−^ and NOX5^+/−^CRE^+/−^ mice). Tamoxifen induction was performed 6–8 weeks after birth and animals were sacrificed one month later. Mice hearts were extracted and processed for the UPR components mRNA expression analysis.

To characterize the effect of NOX5-β endothelial expression in vascular stress, a chronic myocardial infarction model using left coronary artery descending (LAD) ligation was developed in NOX5^+/−^CRE^+/−^ mice. Myocardial infarction was induced in adult mice (15-week-old, male, *n* = 16/group) by permanent ligation of the LAD. Total RNA was isolated from the cardiac region situated above the infarcted zone by using TRIzol. Echocardiographic parameters were measured as previously described [31]. In the present study, in addition to the previously published echocardiographic parameters, data corresponding to the following variables were included: mitral valve wave E (MV_E), mitral valve wave A (MV_A), mitral valve waves ratio E/A (MV_E/A), E’, A’, E’/A’, isovolumetric contraction time (IVCT), isovolumetric relaxation time (IVRT), a, b, index tei and E/E’. Correlation analysis was performed using all echocardiographic parameters and cardiac mRNA expression of the UPR components genes in mice after myocardial infarction.

Experiments were performed in accordance with European Communities Council Directives (2010/63/EU) guidelines for the care and use of laboratory animals and were approved by the University of Navarra Animal Research Review Committee (Protocol 106-17).

### 2.10. Statistical Analysis

Data show the mean ± standard error of the mean (SEM) of at least three independent assays. The significance was calculated using ANOVA when data presented a normal distribution (Shapiro–Wilk and Levene test). When data did not present a normal distribution, the significance was estimated using the Kruskal–Wallis test; in these cases, the nonparametric test was indicated in the pertinent figure legends. In post-hoc analysis, the Tukey test was used for parametric samples and the Mann–Whitney U test for nonparametric samples. Statistical analysis was performed using GraphPad Prism 8 (GraphPad^®^, San Diego, CA, USA). For the in vivo studies, significance was calculated using a t-test. Bivariate correlations between data were obtained using the program Stata. Statistical significance was established as *p* < 0.05.

## 3. Results

### 3.1. In Vitro Model Characterization

**Figure 1:**
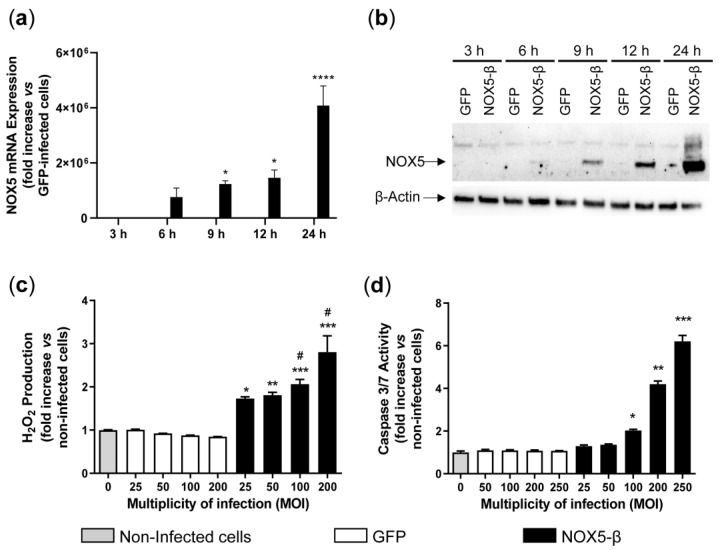
Characterization of NOX5-β overexpression in human aortic endothelial cells (HAEC) after infection with NOX5-β and control green fluorescent protein (GFP) adenoviruses. (**a**) NOX5 mRNA levels at different times after infection with GFP and NOX5-β adenoviruses. *n* = 3. * *p* < 0.05 and **** *p* < 0.0001 vs. GFP-infected cells. (**b**) Representative NOX5 and β-actin immunoblots in HAEC at different times after infection with GFP and NOX5-β adenoviruses. (**c**) Extracellular H_2_O_2_ production of non-infected cells and HAEC infected with GFP or NOX5-β adenoviruses at different multiplicity of infections (MOIs). *n* = 5. * *p* < 0.05 vs. non-infected and GFP-infected cells, ** *p* < 0.01 vs. non-infected and GFP-infected cells, *** *p* < 0.001 vs. non-infected cells and GFP-infected cells. ^#^ *p* < 0.05 vs. the pertinent lower MOIs of NOX5-β-infected cells. (**d**) Apoptotic levels of non-infected cells and HAEC infected with GFP or NOX5-β adenoviruses at different MOIs. *n* = 6. Kruskal-Wallis test was used to analyze these data: * *p* < 0.05, ** *p* < 0.01, *** *p* < 0.001 vs. non-infected cells and GFP-infected cells. Results expressed as mean ± SEM.

HAEC infection with NOX5-β adenovirus (MOI 100) resulted in a higher mRNA expression of this oxidase (Figure 1a). After 6 h of infection, NOX5 was already overexpressed, and its levels increased progressively over time, reaching a maximum 24 h after infection. Levels of NOX5 mRNA were hardly detectable in GFP-infected cells (Figure 1a).

Similar results were obtained when protein levels were evaluated (Figure 1b). Protein levels of NOX5 were detected 6 h after infection and increased progressively over time. The NOX5 protein was undetectable in HAEC infected with the GFP control adenovirus, suggesting a low expression of this gene in these cells at baseline.

Then, we checked whether overexpression of the NOX5 protein correlated with enhanced production of ROS. We measured H_2_O_2_ production 24 h after infection with adenoviruses at different MOIs (Figure 1c). H_2_O_2_ production was higher in NOX5-β-infected cells than in GFP-infected or non-infected cells, in a dose-dependent manner. There were no differences between non-infected and GFP-infected HAEC at any tested MOI (Figure 1c). H_2_O_2_ production decreased in NOX5-β-infected HAEC in the presence of ML090, a NOX5 inhibitor, in a dose-dependent manner (Figure S1a). Similarly, an increased production of intracellular superoxide anion was detected in NOX5-β-infected cells, which was reduced when incubated with ML090 (Figure S1b).

Finally, we analyzed the effect of ROS overproduction on cellular apoptosis. We measured caspase activity 24 h after infection with adenoviruses (Figure 1d). Caspase activity was higher in NOX5-β-infected cells than in GFP-infected or non-infected cells, in a MOI-dependent manner. There were no differences between non-infected and GFP-infected HAEC at any tested MOI (Figure 1d).

### 3.2. Transcriptome Analysis in HAEC

**Figure 2:**
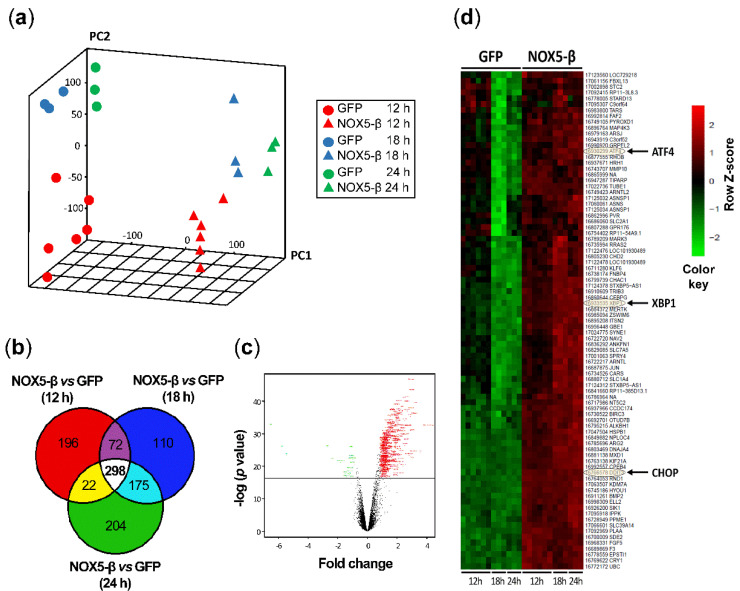
In silico analysis of the transcriptomic array obtained from GFP-infected and NOX5-β-infected HAEC. (**a**) Principal component analysis (PCA) of transcriptomes isolated from cells after infection with GFP and NOX5-β adenovirus for 12 h, 18 h and 24 h. (**b**) Venn diagram showing the genes that suffer alterations comparing NOX5-β vs. GFP. (**c**) Representative volcano plot indicating that most of the alterations triggered by NOX5-β expression were upregulations. (**d**) Representative clustering profiles showing alterations in three of the genes corresponding to the UPR: ATF4, XBP1 and CHOP. *n* = 6 for 12 h, *n* = 3 for 18 h and 24 h.

To examine the full spectrum of NOX5-β-induced effects on the HAEC transcriptome, the Gene Chip^®^ Human Transcriptome Array 2.0 was used. This analysis allows a thorough study of the entire human transcriptome (Figure 2).

Principal component analysis (PCA) showed a clear difference in sample distribution due to NOX5-β overexpression. There were no differences in sample distribution when comparing time variations (Figure 2a). In addition, the Venn diagram constitutes a graphic representation of the number of genes that were altered (NOX5-β vs. GFP) in each time condition (Tables S1–S3). NOX5-β overexpression resulted in the alteration of 298 genes expression at the three time conditions (Figure 2b; Table S4).

Volcano plot analysis showed that NOX5-β overexpression promoted mainly gene upregulation (Figure 2c). The clustering of genes differentially expressed in HAEC after NOX5-β overexpression also showed a clear relationship between gene expression regulation and the action of this oxidase (Figure 2d).

The UPR pathway was strongly altered in HAEC due to NOX5-β overexpression, constituting the most important ontological response (Figure S2). Taking this into account, transcriptomic analysis resulted in an increase in XBP1, IRE1α, CHOP, ATF6, ATF4, PERK, EIF2α and BIP expression after infection with NOX5-β adenovirus at all time conditions (Table S5). In some cases, changes in gene expression occurred only in the initial times (12 or 18h), identifying early players in the UPR response induced by NOX5 (Tables S1 and S2). This was the case for the NFE2L2/Nrf2 (Nuclear factor-E2-related factor 2) gene, which encodes a transcription factor that is involved in both oxidative stress and ER-stress response [32]. On the other hand, among the NADPH oxidase genes, only NOX4 was significantly reduced after infection with NOX5-β adenovirus (Table S6).

### 3.3. Validation of NOX5-β Overexpression on the Levels of the UPR Components Genes in HAEC

To validate the results of our transcriptomic analysis, we performed real-time quantitative PCR experiments. HAEC infection with NOX5-β adenovirus (MOI 100) produced different expression patterns in the mRNA levels of UPR-related genes, depending on the time of infection.

**Figure 3:**
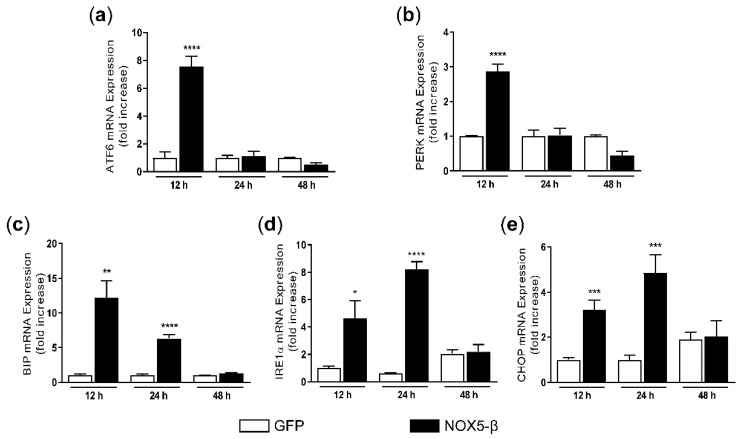
Effects of NOX5-β overexpression on UPR components mRNA in HAEC after infection with GFP and NOX5-β adenoviruses. (**a**) ATF6 mRNA levels. **** *p* < 0.0001 vs. the pertinent time of GFP-infected cells. (**b**) PERK mRNA levels. **** *p* < 0.0001 vs. the pertinent time of GFP-infected cells. (**c**) BIP mRNA levels. ** *p* < 0.01, **** *p* < 0.0001 vs. the pertinent time of GFP-infected cells. (**d**) IRE1α mRNA levels. * *p* < 0.05, **** *p* < 0.0001 vs. the pertinent time of GFP-infected cells. (**e**) CHOP mRNA levels. *** *p* < 0.001vs. the pertinent time of GFP-infected cells. Fold increase vs. GFP-infected cells at 12 h. *n* = 6–12. Results expressed as mean ± SEM.

ATF6 mRNA expression was strongly increased 12 h after infection with NOX5-β adenovirus compared with the GFP-infected group. This effect was absent 24 h and 48 h after infection (Figure 3a). A similar pattern was found for the PERK gene (Figure 3b). In the case of BIP, IRE1α and CHOP, their expression increased 12 h and 24 h after infection with NOX5-β adenovirus compared with GFP-infected cells (Figure 3c–e), while no differences were found 48 h after infection. As the overexpression of these genes remained even at 24 h, these findings support the idea that that NOX5-β overexpression constitutes a potent stimulus of the UPR in HAEC.

### 3.4. Effects of NOX5-β Overexpression on the UPR Components Protein Levels in HAEC

**Figure 4:**
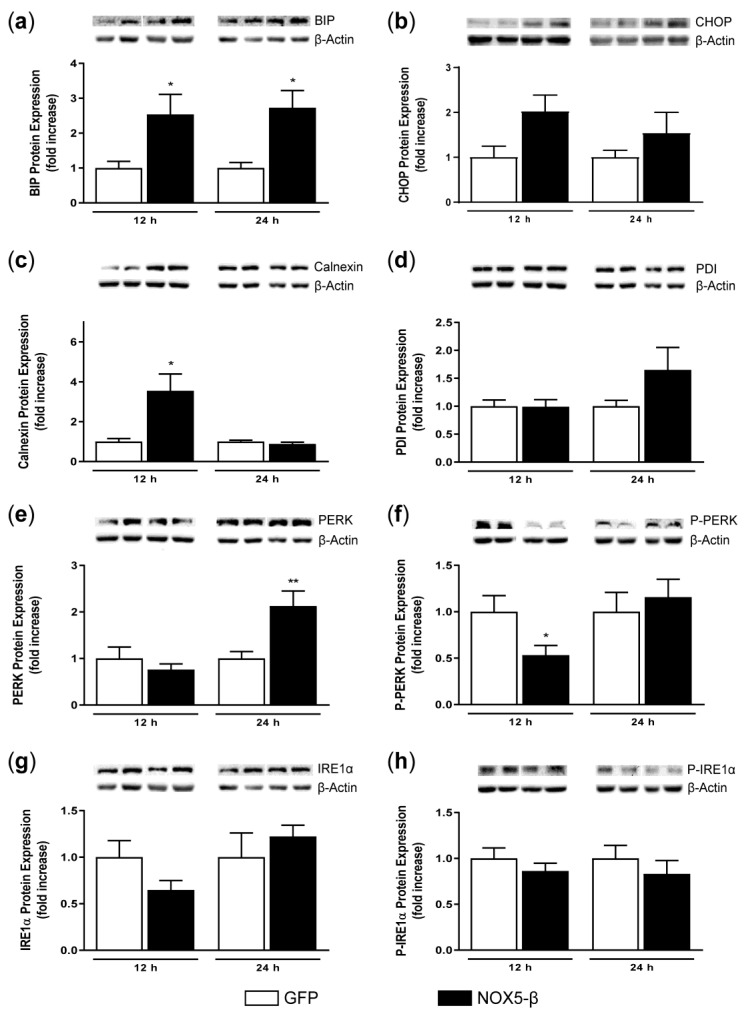
UPR components protein expression in HAEC after infection with GFP and NOX5-β adenoviruses. Representative immunoblots of the UPR components and β-Actin from cells infected cells and its quantification. (**a**) BIP protein expression. * *p* < 0.05 vs. the pertinent time of GFP-infected cells. (**b**) CHOP protein expression. (**c**) Calnexin protein expression. * *p* < 0.05 vs. the pertinent time of GFP-infected cells. (**d**) PDI protein expression. (**e**) PERK protein expression. ** *p* < 0.01 vs. the pertinent time of GFP-infected cells. (**f**) P-PERK protein expression. * *p* < 0.05 vs. the pertinent time of GFP-infected cells. (**g**) IRE1α protein expression. (**h**) P-IRE1α protein expression. Fold increase vs. GFP-infected cells at 12 h. *n* = 3–6. Results expressed as mean ± SEM.

We studied the UPR components protein expression to better characterize NOX5-β implications in this pathway. HAEC infection with NOX5-β adenovirus produced different expression patterns in the protein levels of UPR-related genes, depending on the time of infection compared with GFP-infected cells (MOI 100) (Figure 4).

NOX5-β overexpression induced an enhancement of BIP expression after 12 h and 24 h of infection (Figure 4a). Similarly, CHOP expression showed a slight but not significant increase in NOX5-β-infected cells compared with its respective GFP-infected cells (Figure 4b). Then, we analyzed calnexin and PDI to characterize the adaptive response of the UPR. Calnexin protein expression increased at 12 h in NOX5-β-infected cells (Figure 4c), while PDI suffered no changes at any time condition (Figure 4d). However, PDI protein levels decreased in GFP- and NOX5-β-infected cells at 24 h compared with 12 h.

IRE1α and PERK are two of the three sensors that, when misfolded proteins appear, suffer autophosphorylation and subsequently activate the adaptive response or the apoptotic pathway of the UPR [19]. P-PERK was decreased at 12 h, while no differences appeared in PERK levels at this time (Figure 4e,f). NOX5-β adenoviral infection induced an increase in PERK protein expression at 24 h although no differences were found on its phosphorylation. No differences were observed between groups when analyzing IRE1α or P-IRE1α protein expression (Figure 4g,h).

### 3.5. Proteasome Activity and Apoptosis Determination

**Figure 5:**
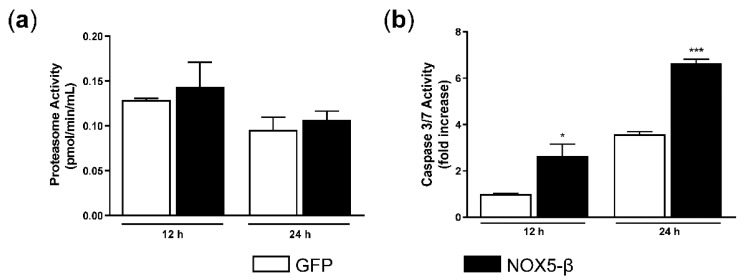
Proteasome activity and apoptosis levels in infected HAEC. (**a**) Proteasome activity determination. (**b**) Apoptosis levels. * *p* < 0.05 and *** *p* < 0.001 vs. the pertinent time of GFP-infected cells. MOI 100. Fold increase vs. GFP-infected cells at 12 h. *n* = 6–12. Results expressed as mean ± SEM. Kruskal-Wallis test was used to analyze these data in both experiments.

UPR activation could follow an adaptive pathway (proteasome activation) or an apoptotic pathway [18]. We found no differences in proteasome activity between GFP- and NOX5-β-infected (Figure 5a). However, NOX5-β overexpression increased apoptosis at 12 h and 24 h (Figure 5b). This assay may indicate that NOX5-β overexpression in HAEC and its consequent ROS production leads to apoptosis.

### 3.6. UPR Components Gene Expression in Knock-in Mice

**Figure 6:**
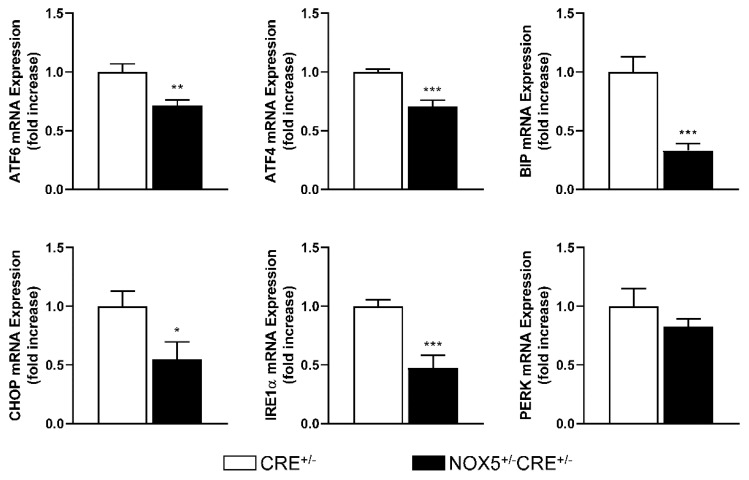
Cardiac UPR components mRNA expression of transgenic mice. CRE^+/−^, control mice overexpressing CRE recombinase. NOX5^+/−^CRE^+/−^, mice overexpressing NOX5-β. * *p* < 0.05, ** *p* < 0.01 and *** *p* < 0.001 vs. the pertinent CRE^+/−^ mice. *n* = 9. Results expressed as mean ± SEM.

Cardiac UPR components mRNA expression in control CRE mice (CRE^+/−^) and mice expressing NOX5-β in endothelial cells (NOX5^+/−^CRE^+/−^) was analyzed. In our experimental conditions, 6–8 weeks after tamoxifen induction of NOX5^+/−^CRE^+/−^ mice, ATF6, ATF4, CHOP, BIP and IRE1α were downregulated compared with control mice (Figure 6).

We evaluated the UPR components expression in a situation of vascular stress. Thus, cardiac mRNA expression of NOX5^+/−^CRE^+/−^ mice and control CRE^+/−^ mice suffering myocardial infarction was analyzed. No differences were found in the UPR components gene expression between mice CRE^+/−^ or NOX5^+/−^CRE^+/−^ (Figure S3). We observed no differences between groups in echocardiographic values, similarly to previous findings [31] (Table 3).

**Figure 7:**
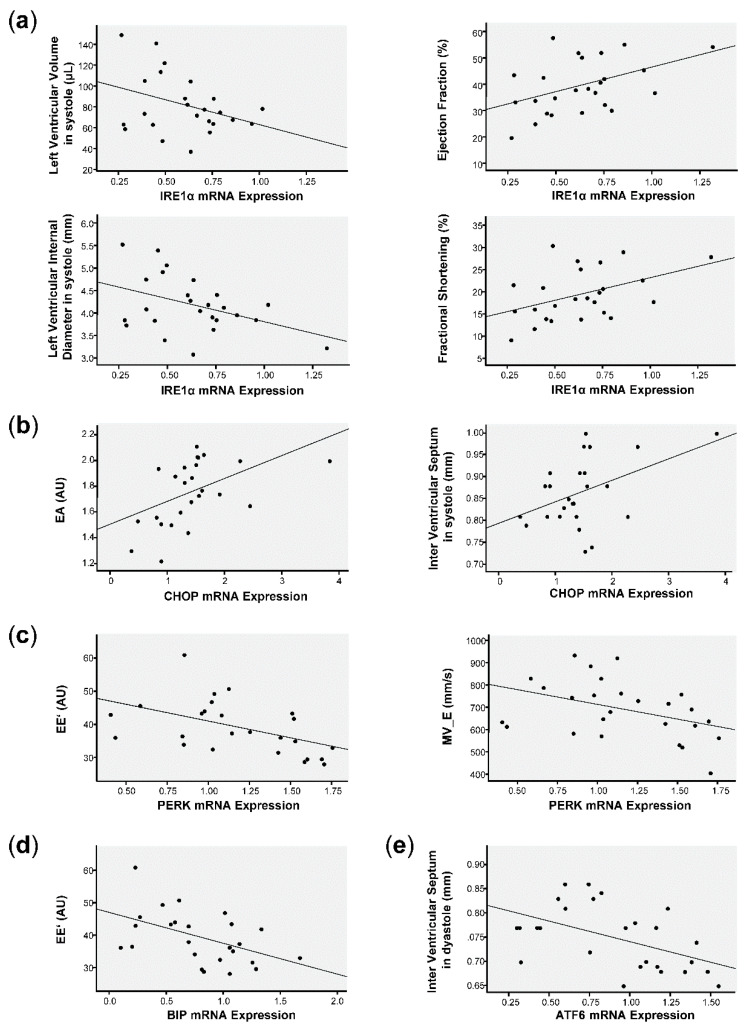
Correlations analysis between echocardiographic parameters and UPR components gene expression in CRE^+/−^ and NOX5^+/−^CRE^+/−^ mice, after LAD ligation. (**a**) IRE1α with ventricular volume in systole (R = −0.413, *p* = 0.04), left ventricular internal diameter in systole (R = −0.418, *p* = 0.037), ejection fraction (R = 0.450, *p* = 0.024) and fractional shortening (R = 0.441, *p* = 0.027). (**b**) CHOP with EA (R = 0.493, *p* = 0.012) and Inter Ventricular septum in systole (R = 0.443, *p* = 0.027). (**c**) PERK with EE’ (R = −0.503, *p* = 0.010) and MV E (R = −0.397, *p* = 0.050) (**d**) BIP with EE’ (R = −0.487, *p* = 0.014). (**e**) ATF6 with Inter Ventricular septum in dyastole (R = −0.518, *p* = 0.008). AU, arbitrary units. *n* = 25.

To further evaluate the relation between UPR components gene expression and echocardiographic parameters in these mice, correlation analysis was performed (Figure 7).

Correlations between IRE1α and the echocardiographic parameters resulted in four significant interactions. The analysis showed a negative correlation of IRE1α gene expression with the left ventricular volume and the left ventricular internal diameter both in systole. Additionally, a positive correlation of IRE1α gene expression was obtained with the ejection fraction and fractional shortening, both indicators of cardiac function (Figure 7a). Additionally, CHOP gene expression positively correlated with the EA, an indicative parameter of the left ventricle filling, and with the inter-ventricular septum in systole (Figure 7b). PERK showed a negative correlation with EE’, an indicator of diastolic dysfunction, and MV E (Figure 7c). BIP also negatively correlated with EE’ (Figure 7d). Finally, we found a negative correlation between ATF6 and the inter-ventricular septum in diastole (Figure 7e).

## 4. Discussion

The main findings obtained in this study are the following: (i) transcriptome analysis postulates UPR as the most important ontological response to NOX5-β overexpression in HAEC; (ii) NOX5-β overexpression produces time-dependent changes in the UPR expression patterns in HAEC; (iii) NOX5-β overexpression induces no differences in proteasome activity, while apoptosis activity is increased in NOX5-β-infected HAEC; (iv) endothelial NOX5-β overexpression downregulates the cardiac UPR pathway in *knock-in* mice at baseline; (v) cardiac IRE1α, CHOP, PERK, BIP and ATF6 mRNA levels associate with several echocardiographic parameters in mice undergoing chronic myocardial infarction. This work demonstrates, for the first time, a crosstalk between NOX5-β and the UPR in vascular endothelial cells, supporting a potential key role in cardiovascular pathophysiology.

The activation of the UPR emerges as a key mechanism that underlies several CVDs [17]. Among others, the UPR is related to many cardiac functions in several disorders including cardiogenesis, ischemia-reperfusion, cardiomyopathies, and even heart failure. ER stress plays an important role in the transition from cardiac hypertrophy to cardiac failure, since CHOP protein expression is increased during prolonged stress favoring the apoptotic pathway [23,33]. In addition, BIP gene expression is also increased in patients with heart failure. This directly relates to UPR alteration and vascular pathophysiology [18].

NOX5 expression in endothelial cells promotes cell proliferation and cellular reorganization in three-dimensional structures that resemble capillary networks [11]. This suggests that NOX5 is required for endothelial cell homeostasis. Transcriptome analysis performed in HAEC showed that NOX5-β-derived ROS induced changes in the expression of 298 genes, most of them corresponding to upregulations. The subsequent ontological study identified that the most notable expression changes induced by NOX5 corresponded to the UPR pathway. These findings provide a new role for NOX5-produced ROS by promoting activation of the UPR in situations associated with oxidative stress. In agreement with this, it is known that ROS produced by NOX enzymes take part in ER stress and protein misfolding [34]. In this way, ER oxidative stress can induce protein misfolding that is recognized by chaperone-binding immunoglobulins. A crosstalk between oxidative stress produced by NOXs and the UPR is supported by several studies. Firstly, PDI promotes NOX activation by regulating subunit assembling [35,36]. Secondly, NOX2 and NOX4 are involved in UPR regulation [11,22]. In this way, calnexin stablishes molecular complexes with NOX4, necessary for the appropriate function of this enzyme [37]. Finally, chaperones like Hsp90 and Hsp70 can interact with different NOX [38]. In fact, NOX5 activation is regulated by the action of Hsp90, Hsp70 and caveolin-1 [10]. Collectively, our findings provide a new role for NOX5-produced ROS by promoting UPR activation in oxidative stress situations.

Three transmembrane sensors (ATF6, IRE1α and PERK) that remain inactive due to its binding to BIP under normal conditions constitute the UPR. PERK and IRE1α are activated through trans-autophosphorylation and oligomerization when misfolded proteins appear, and in turn, they lead to an adaptive or apoptotic pathway [33,39]. We found an increase in PERK mRNA expression after 12 h of infection, which correlated with enhanced protein expression after 24 h. The decreased PERK phosphorylation at 12 h in NOX5-β-infected cells compared with GFP-infected cells identified an early shutdown of this sensor, which allows us to understand the later increase in protein levels as a compensatory mechanism. On the other hand, IRE1α is a transmembrane sensor that, when activated, induces the alternative splicing of XBP1 to its active form (XBP1s). This activation leads to the expression of chaperone-related genes, which induce the degradation of misfolded proteins associated with ER [19,33]. In our study, we found no changes to protein levels or phosphorylation degree of IRE1α. Finally, ATF6 mRNA expression increased as an early response to NOX5-β overexpression after 12 h, which was also maintained after 24 h. During ER stress, ATF6 is processed by the Golgi apparatus, and then translocated to the nucleus where it activates the expression of genes involved in the UPR. Through this process, misfolded proteins may be finally degraded by the proteasome system [18,33].

BIP is an immunoglobulin that remains attached to ATF6, PERK and IRE1α. Then, when the UPR is turned on, BIP dissociates and binds to misfolded proteins, acting like a chaperone and allowing sensor activation [40,41]. We found that BIP mRNA and protein expression increased as an early response to NOX5-β overexpression in HAEC. The UPR-triggered adaptive pathway leads to the expression of several chaperones that regulate the protein secretion ratio. Chaperones may also recognize and degrade misfolded proteins and protein aggregates by enhancing proteasome activity [42]. Calnexin and PDI are chaperones that guarantee correct protein folding. Whereas, PDI is a critical player in oxidative folding of ER and extracellular proteins [43], calnexin is a critical regulator of the mitochondria-ER contact site [44]. In the present study, the expression of calnexin was increased early, at 12 h, in NOX5-β-infected cells. On the contrary, PDI suffered no protein expression changes. According to these results, calnexin expression may be influenced by NOX5-generated ROS as an early compensatory mechanism. Additionally, CHOP is a transcription factor activated by the action of ATF6, PERK and IRE1α pathways [43]. This activation is triggered predominantly by PERK, which plays an important role in the induction of cellular apoptosis during ER stress [45]. In the two snapshots (at 12 and 24 h) obtained in this study, although NOX5-β induced upregulation of CHOP mRNA, we detected only a slight increase in its protein levels.

**Figure 8:**
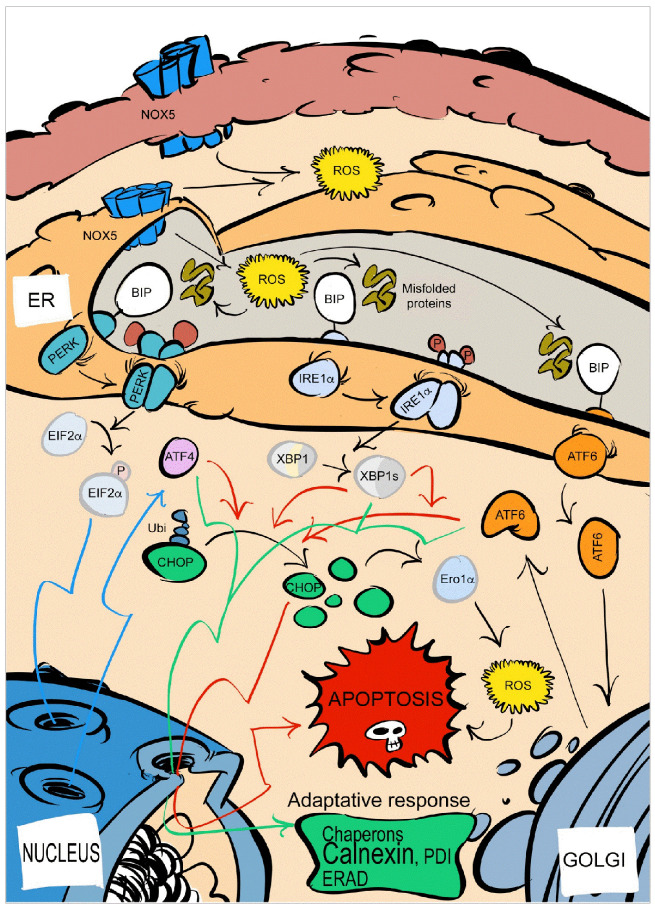
UPR schematic representation and the possible influence of NOX5-derived ROS. ER, endoplasmic reticulum; ROS, reactive oxygen species; ERAD, endoplasmic-reticulum-associated protein degradation; Ubi, ubiquitination; P, phosphate.

The in vitro results showed a close relationship between oxidative stress produced by NOX5-β and UPR activation in HAEC. These expression changes were not followed by changes in proteasome activity. Nonetheless, NOX-derived ROS may lead to cell apoptosis activation in states of ER stress [46,47]. Accordingly, NOX5-β overexpression-mediated oxidative stress promoted apoptosis in endothelial cells in a dose- and time-dependent manner. Considering all these data, we propose that the accumulation of unfolded proteins due to NOX5-β-derived ROS may directly lead to cell apoptosis through UPR activation (Figure 8).

Inhibition of the UPR has been shown to improve coronary artery function and reduce blood pressure in hypertensive rats [48]. These data postulate a crosstalk between the UPR and vascular disease. Our in vivo findings showed that endothelial overexpression of NOX5-β induced the downregulation of UPR components genes. However, although previous studies showed an upregulation of this pathway during CVDs [48], no differences were found when the UPR was analyzed in NOX5 transgenic mice with myocardial infarction. The absence of changes in the UPR might suggest that the LAD ligation induces intense alterations in the heart (cardiac remodeling), which predominate over the genotype. Interestingly, the mRNA levels of the UPR components genes correlated with several echocardiographic parameters, thus suggesting a potential link between the UPR and alterations in cardiac structure and functionality.

## 5. Conclusions

In summary, we demonstrated that NOX5-derived ROS induce changes to UPR pathway expression in human endothelial cells. Given that UPR activation is altered in vascular pathophysiology [18], our findings support the idea that NOX5-derived ROS may play a relevant role in turning on the UPR during the development of CVDs. In this context, NOX5 emerges as a new therapeutic target, as its inactivation could prevent cell damage associated with UPR activation in CVDs.

## Figures and Tables

**Table 1:** Specific primer sequences for the quantitative real-time polymerase chain reaction.

Gene	Accession N°	Primers	
*Hs ATF6*	NM_007348.4	forward	5′-GCTGGATGAAGTTGTGTCAGAG-3′
		reverse	5′-TGTTCCAACATGCTCATAGGTC-3′
*Hs BIP*	NM_005347.5	forward	5′-GAGCTGTGCAGAAACTCCGGCG-3′
		reverse	5′-ACCACCTGCTGAATCTTTGGAATTCGAGT-3′
*Hs IRE1α*	NM_001433.5	forward	5′-CCGATCGTGAAGCAGTTAGAG-3′
		reverse	5′- AGGTCCTGAATTTACGCAGGT-3′
*Hs PERK*	NM_004836.7	forward	5′-CAGGCAAAGGAAGGAGTCTG-3′
		reverse	5′-AACAACTCCAAAGCCACCAC-3′
*Hs CHOP*	NM_001195053.1	forward	5′-AGTGCCACGGAGAAAGCTAA-3′
		reverse	5′-CCATACAGCAGCCTGAGTGA-3′
*Hs NOX5*	NM_001184780.2	forward	5′-ATGAGTGCCGAGGAGGATG-3′
		reverse	5′-ATCGATGGCAGTGGCTCCAT-3′
*Hs GAPDH*	NM_001289745.3	forward	5′-CCAAGGTCATCCATGACAAC-3′
		reverse	5′-TGTCATACCAGGAAATGAGC-3′
*Mm ATF6*	NM_001081304.1	forward	5′-GATCACTCTGTGTCCAATGACAA-3′
		reverse	5′-TAGATTTGGTCCTTTCCACTTCA-3′
*Mm ATF4*	NM_009716.3	forward	5′-AAACCTCATGGGTTCTCCAG-3′
		reverse	5′-GAGGGGCAAAAAGATCACAT-3′
*Mm BIP*	NM_001163434.1	forward	5′-AGGAGACTGCTGAGGCGTAT-3′
		reverse	5′-CCAGGTCAAACACAAGGATG-3′
*Mm IRE1α*	NM_023913.2	forward	5′-AATAGAAAAGGAGGCCTTGGAC-3′
		reverse	5′-TGGTGTTTCTTGTTTCTCATGG-3′
*Mm PERK*	NM_001313918.1	forward	5′-CGATCAAATGGAAGCCCTTA-3′
		reverse	5′-TGCGGATGTTCTTGCTGTAG-3′
*Mm CHOP*	NM_007837.4	forward	5′-AGGTCCTGTCCTCAGATGAA-3′
		reverse	5′-GACCACTCTGTTTCCGTTTC-3′
*Mm GAPDH*	NM_001289726.1	forward	5′-ATGACAACTTTGTCAAGCTCATTT-3′
		reverse	5′-GGTCCACCACCCTGTTGCT-3′

*Hs, Homo sapiens; Mm, Mus musculus.*

**Table 2:** Antibodies used for the immunodetection of NOX5, unfolded protein response (UPR) and β-Actin.

Primary Antibody	Brand (Dilution)	Secondary Antibody	Brand (Dilution)	MW (kDa)
BIP	Cell Signalling (1:1000)	Anti-rabbit	Cell Signalling (1:2000)	78
Calnexin	Cell Signalling (1:1000)	Anti-rabbit	Cell Signalling (1:2000)	90
IRE1α	Cell Signalling (1:1000)	Anti-rabbit	Cell Signalling (1:2000)	130
P-IRE1α	Thermo Scientific (1:1000)	Anti-rabbit	GE Healthcare (1:2000)	110
PDI	Cell Signalling (1:1000)	Anti-rabbit	Cell Signalling (1:2000)	57
PERK	Cell Signalling (1:1000)	Anti-rabbit	Cell Signalling (1:2000)	140
P-PERK	Cell Signalling (1:1000)	Anti-rabbit	Cell Signalling (1:2000)	170
CHOP	Cell Signalling (1:500)	Anti-mouse	Cell Signalling (1:2000)	27
NOX5	Abcam (1:2000)	Anti-rabbit	Cell Signalling (1:4000)	86
β-Actin	Sigma-Aldrich (1:10,000)	Anti-mouse	GE Healthcare (1:10,000)	45

MW: Molecular weight.

**Table 3:** Echocardiographic parameters in mice baseline and after left coronary artery descending (LAD) ligation.

	Basal		2 Days		28 Days	
	NOX5^+/−^ CRE^+/−^	CRE^+/−^	NOX5^+/−^ CRE^+/−^	CRE^+/−^	NOX5^+/−^ CRE^+/−^	CRE^+/−^
**MV E, mm/s**	889 ± 18	900 ± 28	742 ± 28	726 ± 34	680 ± 38	655 ± 45
**MV A, mm/s**	527 ± 13	522 ± 16	317 ± 19	356 ± 28	376 ± 29	400 ± 41
**MV E/A, AU**	1.70 ± 0.03	1.73 ± 0.02	2.47 ± 0.17	2.12 ± 0.12	1.89 ± 0.10	1.71 ± 0.07
**E’, mm/s**	21.3 ± 0.5	20.8 ± 0.6	16.3 ± 0.8	16.0 ± 0.7	18.2 ± 0.9	17 ± 80.6
**A’, mm/s**	13.1 ± 0.5	12.5 ± 0.4	9.1 ± 0.5	8.6 ± 0.5	10.5 ± 0.7	10.6 ± 0.7
**E’/A’, AU**	1.64 ± 0.05	1.67 ± 0.03	1.81 ± 0.06	1.89 ± 0.06	1.77 ± 0.05	1.73 ± 0.07
**IVCT, ms**	8.83 ± 0.24	9.38 ± 0.36	10.75 ± 0.42	10.87 ± 0.64	10.80 ± 0.78	11.60 ± 1.03
**IVRT, ms**	13.75 ± 0.55	13.36 ± 0.60	17.92 ± 0.80	19.52 ± 0.82	20.45 ± 1.41	20.69 ± 0.88
**a, ms**	76.38 ± 1.80	77.34 ± 2.86	77.25 ± 2.67	80.67 ± 2.88	86.79 ± 5.01	92.71 ± 3.82
**b, ms**	54.02 ± 1.40	54.14 ± 2.17	45.58 ± 2.00	50.29 ± 2.30	55.89 ± 3.59	60.62 ± 2.58
**Index Tei, AU**	1.72 ± 0.01	1.43 ± 0.01	1.60 ± 0.02	1.62 ± 0.04	1.56 ± 0.03	1.53 ± 2.78
**E/E’, AU**	42.08 ± 1.04	43.47 ± 1.09	46.34 ± 2.01	45.74 ± 1.92	38.26 ± 2.40	37.23 ± 2.78

Data are represented as the mean ± SEM. CRE^+/−^, mice with the CRE genotype (*n* = 12); NOX5^+/−^CRE^+/−^, mice with the NOX5^+/−^CRE^+/−^ genotype (*n* = 13); MV E, mitral valve wave E; MV A, mitral valve wave A; MV E/A, mitral valve waves ratio E/A; IVCT, isovolumetric contraction time; IVRT, isovolumetric relaxation time; AU, arbitrary units.

## Data Availability

The data presented in this study are available on request from the corresponding author.

## Supplementary Materials

The following are available online at https://www.mdpi.com/2076-3921/10/2/194/s1, Figure S1: HAEC ROS production and ML090 NOX5-inhibition; Figure S2: Ontology analysis of HAEC infected with NOX5-β; Figure S3: UPR components mRNA expression in heart of infarcted transgenic mice; Table S1: Gene expression altered due to NOX5-β expression at 12 h; Table S2: Gene expression altered due to NOX5-β expression at 18 h; Table S3: Gene expression altered due to NOX5-β expression at 24 h; Table S4: Gene expression altered due to NOX5-β expression; Table S5: UPR components transcriptome expression in HAEC after infection with GFP and NOX5-β adenovirus; Table S6: NOX family transcriptome expression in HAEC cells after infection with GFP and NOX5-β adenoviruses.
