# Supplementary material for: NADPH Oxidase 5 Induces Changes in the Unfolded Protein Response in Human Aortic Endothelial Cells and in Endothelial-Specific Knock-in Mice
Source: Antioxidants (Basel). 2021 Jan 29;10(2):194. doi: 10.3390/antiox10020194 (PMC7911693; doi:10.3390/antiox10020194)
Supplement: Supplementary file 1 [file antioxidants-10-00194-s001.pdf]

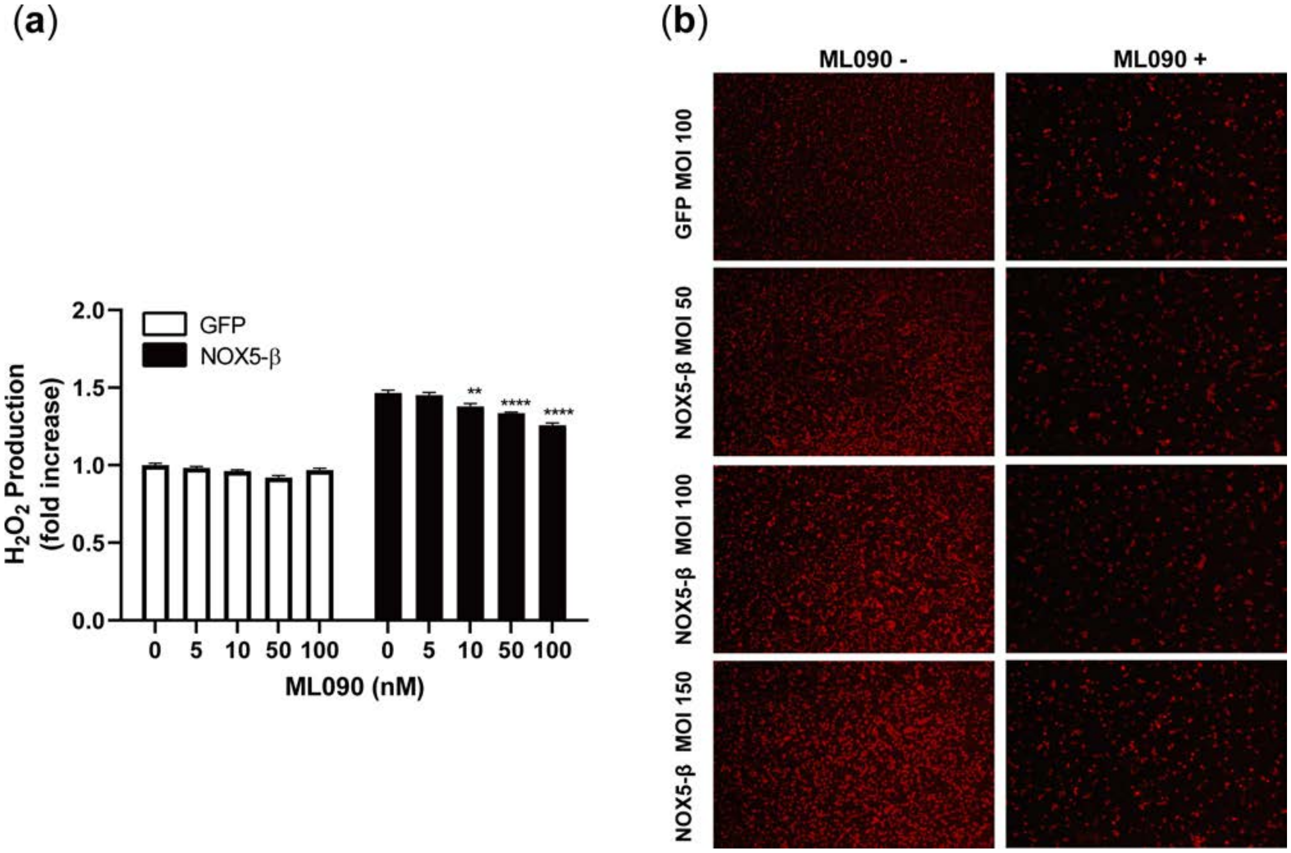

Figure S1. HAEC ROS production and ML090 NOX5-inhibition. (**a**) Extracellular H_2O_2 production in HAEC treated with ML090 at different concentrations and 24 h after being infected with GFP and NOX5-β adenoviruses (MOI 100). $^{**}p < 0.01$, and $^{****}p < 0.0001$ *vs* control NOX5-β-infected cells (ML090, 0 nM). Results expressed as mean ± SEM. Fold increase *vs* GFP-infected cells with 0 nM of ML090. $n = 6$. (**b**) NOX5-β overexpression and DHE oxidation in HAEC. Representative images from three experiments are shown. Intracellular superoxide anion production of HAEC 24 h after infection with GFP and NOX5-β adenoviruses at different MOIs treated or not with ML090 (10 nM). MOI: Multiplicity of infection.

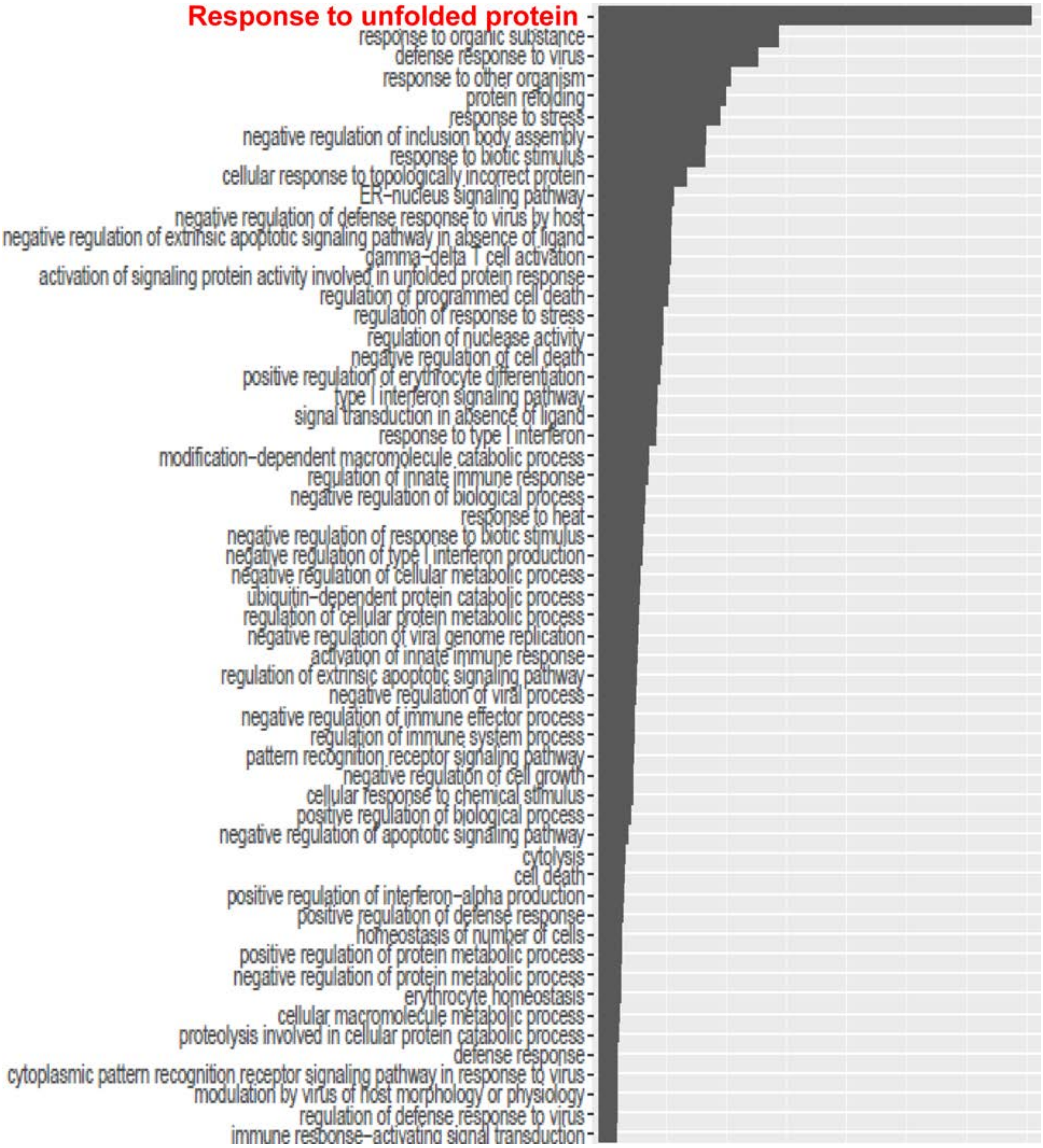

Figure S2. Ontology analysis of HAEC infected with NOX5-β. Ontology analysis shows that the response to unfolded protein is the most relevant.

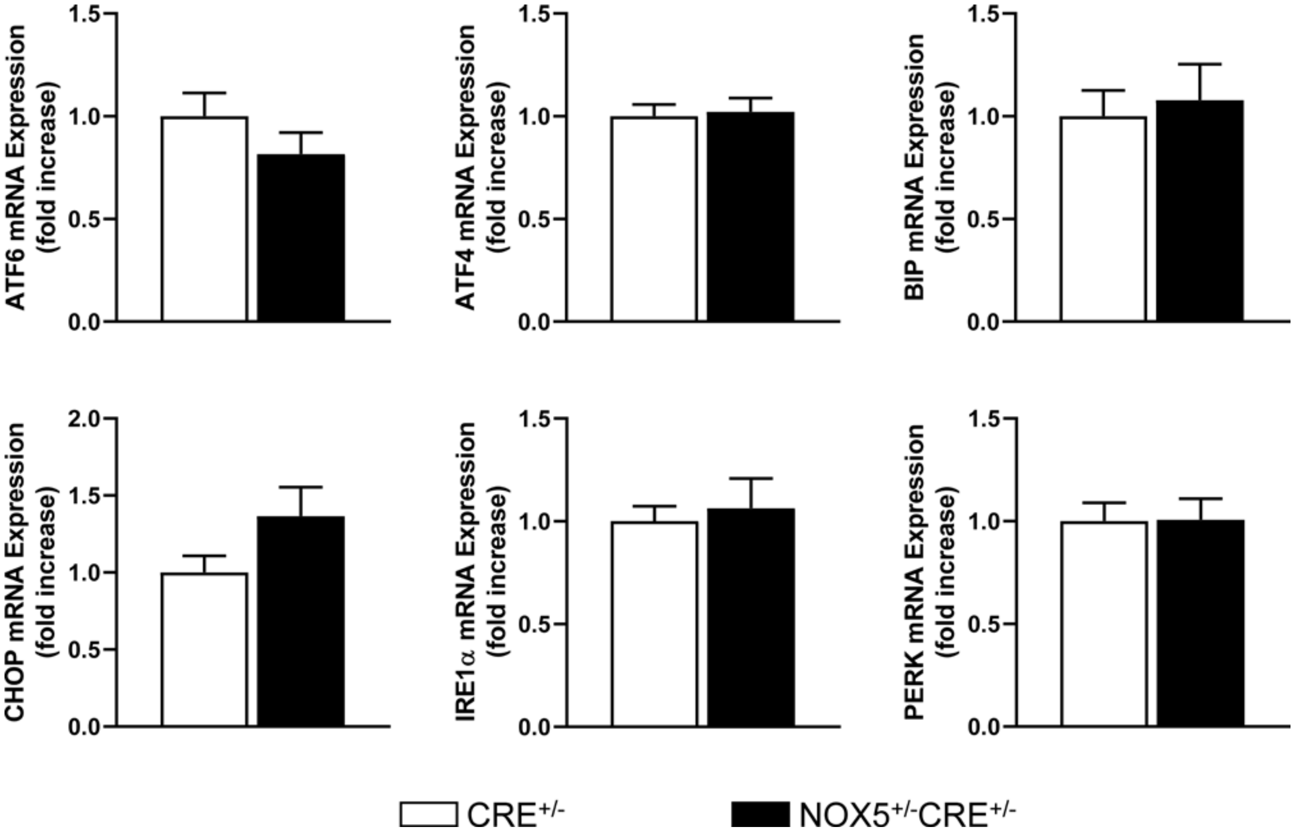

Figure S3. UPR mRNA expression in heart of infarcted transgenic mice. *n*= 12-13. Results expressed as mean ± SEM.

Table S1: Altered gene expression due to NOX5-β expression at 12 h (bold, highlighted in yellow).

GeneName	GeneDescription	TranscriptID	N12h*vs*G12h		N18h*vs*G18h		N24h*vs*G24h	
			logFC	*p-value*	logFC	*p-value*	logFC	*p-value*
FAM129A	family with sequence similarity 129. member A	NM_052966	**1.45**	**1.20E-17**	2.44	3.27E-19	2.96	6.24E-21
DNAJA4	DnaJ (Hsp40) homolog. subfamily A. member 4	NM_001130182	**2.19**	**9.83E-20**	2.94	2.90E-19	3.01	1.68E-19
PMAIP1	phorbol-12-myristate-13-acetate-induced protein 1	NM_021127	**0.93**	**1.84E-12**	2.41	1.32E-17	2.69	1.43E-18
E2F7	E2F transcription factor 7	NM_203394	**0.71**	**8.35E-11**	2.20	2.21E-17	2.48	1.84E-18
DNAJB1	DnaJ (Hsp40) homolog. subfamily B. member 1	NM_006145	**1.95**	**2.72E-20**	2.30	1.04E-18	2.13	5.07E-18
PTGS2	prostaglandin-endoperoxide synthase 2 (prostaglandin G/H synthase and cyclooxygenase)	NM_000963	**3.80**	**3.74E-24**	3.89	2.89E-21	2.68	5.83E-18
RND1	Rho family GTPase 1	ENST00000548445	**3.09**	**2.20E-20**	3.42	3.17E-18	3.25	8.99E-18
CHRNA5	cholinergic receptor. nicotinic. alpha 5 (neuronal)	NM_000745	**1.30**	**6.71E-17**	1.78	1.31E-16	1.99	1.44E-17
IL7R	interleukin 7 receptor	NM_002185	**2.17**	**1.43E-19**	2.62	3.61E-18	2.36	3.12E-17
ACTRT3	actin-related protein T3	NM_032487	**1.55**	**1.52E-14**	2.48	1.35E-15	2.94	4.50E-17
CCRN4L	CCR4 carbon catabolite repression 4-like (S. cerevisiae)	NM_012118	**1.92**	**4.22E-19**	2.05	1.30E-16	2.13	6.21E-17
SAT1	spermidine/spermine N1-acetyltransferase 1	NM_002970	**0.82**	**2.04E-13**	1.76	4.50E-17	1.73	6.60E-17
SLC7A5	solute carrier family 7 (amino acid transporter light chain. L system). member 5	NM_003486	**0.69**	**1.39E-08**	2.39	7.59E-16	2.69	7.18E-17
HERPUD1	homocysteine-inducible. endoplasmic reticulum stress-inducible. ubiquitin-like domain member 1	NM_001010989	**1.45**	**1.45E-18**	1.47	1.10E-15	0.36	3.46E-05
GPR89A	G protein-coupled receptor 89A	XM_006711492	**1.10**	**4.66E-16**	1.44	2.24E-15	1.69	8.05E-17
NFKBIZ	nuclear factor of kappa light polypeptide gene enhancer in B-cells inhibitor. zeta	NM_001005474	**0.84**	**2.64E-10**	2.32	7.66E-16	2.60	8.20E-17
GPR89A	G protein-coupled receptor 89A	NM_001097612	**1.05**	**1.23E-15**	1.44	2.19E-15	1.70	8.33E-17
DUSP1	dual specificity phosphatase 1	NM_004417	**0.76**	**2.57E-10**	1.84	1.11E-14	2.31	1.20E-16
HSPA4L	heat shock 70kDa protein 4-like	NM_014278	**1.22**	**9.72E-16**	1.52	1.25E-14	1.90	1.45E-16
STXBP5-AS1	STXBP5 antisense RNA 1	NR_034115	**0.56**	**2.46E-08**	1.46	3.28E-13	2.12	2.15E-16
DNAJB9	DnaJ (Hsp40) homolog. subfamily B. member 9	NM_012328	**1.81**	**5.93E-18**	1.19	2.02E-11	0.53	1.00E-05
CHAC1	ChaC. cation transport regulator homolog 1 (E. coli)	ENST00000446533	**1.01**	**1.68E-09**	3.74	1.72E-17	3.28	2.49E-16
GADD45B	growth arrest and DNA-damage-inducible. beta	NM_015675	**0.88**	**6.24E-13**	1.53	1.25E-14	1.86	2.66E-16
IER5	immediate early response 5	NM_016545	**1.01**	**1.06E-12**	1.85	6.09E-15	2.16	2.98E-16
MXD1	MAX dimerization protein 1	NM_001202513	**1.46**	**2.88E-17**	2.08	2.44E-17	1.81	3.86E-16
ULBP1	UL16 binding protein 1	NM_025218	**0.96**	**8.52E-10**	2.12	2.11E-13	2.91	3.91E-16
RP11-274H2.3	novel transcript	OTTHUMT00000355183	**2.01**	**1.27E-18**	2.29	9.69E-17	2.14	3.92E-16
JMJD1C	jumonji domain containing 1C	NM_001282948	**0.61**	**3.49E-11**	1.44	1.99E-15	1.56	4.05E-16
BAG3	BCL2-associated athanogene 3	NM_004281	**1.93**	**3.06E-19**	2.00	1.62E-16	1.91	4.28E-16
KITLG	KIT ligand	NM_000899	**1.52**	**7.96E-17**	2.12	1.05E-16	1.97	4.77E-16
BMP2	bone morphogenetic protein 2	NM_001200	**1.60**	**1.08E-16**	2.16	2.80E-16	2.04	9.00E-16

ERN1	endoplasmic reticulum to nucleus signaling 1	NM_001433	**1.32**	**1.37E-14**	2.24	4.02E-16	2.12	1.16E-15
SESN2	sestrin 2	NM_031459	**1.70**	**3.90E-15**	2.81	1.70E-16	2.53	1.37E-15
SIX4	SIX homeobox 4	NM_017420	**0.86**	**1.31E-11**	1.55	1.40E-13	1.95	1.39E-15
GDF15	growth differentiation factor 15	NM_004864	**0.67**	**3.73E-11**	1.54	2.78E-15	1.58	1.80E-15
CAP2	CAP. adenylate cyclase-associated protein. 2 (yeast)	NM_006366	**0.85**	**5.80E-15**	1.09	4.04E-14	1.27	1.90E-15
C7orf60	chromosome 7 open reading frame 60	NM_152556	**0.99**	**4.36E-12**	1.79	3.96E-14	2.07	2.35E-15
LOC727896	cysteine and histidine-rich domain (CHORD) containing 1 pseudogene	NR_026659	**1.33**	**1.29E-15**	1.64	1.89E-14	1.80	3.16E-15
HSPD1	heat shock 60kDa protein 1 (chaperonin)	NM_002156	**1.34**	**9.15E-18**	1.36	7.26E-15	1.40	3.76E-15
KIF21A	kinesin family member 21A	NM_001173463	**1.55**	**2.58E-17**	1.91	4.47E-16	1.70	4.18E-15
NA	NA	---	**1.50**	**2.08E-15**	1.86	2.82E-14	2.04	4.78E-15
USP53	ubiquitin specific peptidase 53	NM_019050	**0.93**	**5.15E-14**	1.45	7.98E-15	1.49	4.92E-15
SLFN11	schlafen family member 11	NM_001104587	**1.26**	**1.22E-15**	0.39	1.37E-04	0.07	3.83E-01
BACH1	BTB and CNC homology 1. basic leucine zipper transcription factor 1	NR_027655	**1.12**	**1.74E-14**	1.61	1.19E-14	1.67	5.86E-15
NA	NA	RPTR-U57609-1	**-7.43**	**5.14E-19**	-6.37	1.18E-14	-6.56	6.76E-15
NA	NA	RPTR-U43284-1	**-7.43**	**5.14E-19**	-6.37	1.18E-14	-6.56	6.76E-15
NA	NA	RPTR-AB076373-2	**-7.43**	**5.14E-19**	-6.37	1.18E-14	-6.56	6.76E-15
PPP1R15A	protein phosphatase 1. regulatory subunit 15A	NM_014330	**1.16**	**8.86E-13**	1.73	3.11E-13	2.08	8.84E-15
CPEB4	cytoplasmic polyadenylation element binding protein 4	NM_030627	**1.42**	**6.06E-17**	1.91	1.56E-16	1.56	9.34E-15
RP11-212D19.4	novel transcript. RBM7-REXO2 readthrough	OTTHUMT00000399017	**2.51**	**1.98E-13**	3.50	2.55E-13	4.13	1.03E-14
ADAMTS1	ADAM metallopeptidase with thrombospondin type 1 motif. 1	NM_006988	**1.05**	**5.61E-15**	0.05	5.50E-01	0.10	2.09E-01
LOC100128233	uncharacterized LOC100128233	NR_103769	**0.94**	**4.35E-09**	2.15	6.61E-13	2.66	1.05E-14
TNFAIP3	tumor necrosis factor. alpha-induced protein 3	NM_001270507	**1.20**	**5.69E-14**	1.72	4.34E-14	1.83	1.28E-14
NAA16	N(alpha)-acetyltransferase 16. NatA auxiliary subunit	NM_001110798	**0.91**	**3.86E-13**	1.44	3.87E-14	1.51	1.51E-14
MLKL	mixed lineage kinase domain-like	NM_152649	**0.71**	**3.62E-11**	1.20	1.16E-12	1.49	1.86E-14
HSPH1	heat shock 105kDa/110kDa protein 1	NM_001286503	**1.35**	**1.53E-17**	1.41	6.52E-15	1.34	1.93E-14
MB21D1	Mab-21 domain containing 1	NM_138441	**1.02**	**3.59E-10**	2.02	6.35E-13	2.41	2.08E-14
TUBE1	tubulin. epsilon 1	NM_016262	**0.57**	**6.14E-09**	1.22	3.75E-12	1.60	2.08E-14
GOT1	glutamic-oxaloacetic transaminase 1. soluble	NM_002079	**0.99**	**7.08E-13**	1.75	8.15E-15	1.67	2.11E-14
IL1A	interleukin 1. alpha	NM_000575	**1.35**	**5.27E-13**	2.04	1.44E-13	2.19	3.66E-14
HSPA1A	heat shock 70kDa protein 1A	NM_005345	**1.46**	**5.46E-19**	1.38	1.97E-15	1.17	5.07E-14
GCLM	glutamate-cysteine ligase. modifier subunit	NM_002061	**0.84**	**2.13E-14**	0.77	8.75E-11	0.36	1.56E-05
ERO1LB	ERO1-like beta (S. cerevisiae)	NM_019891	**1.29**	**2.88E-14**	1.56	6.36E-13	0.72	4.47E-07
KDM7A	lysine (K)-specific demethylase 7A	NM_030647	**1.61**	**1.28E-16**	2.02	1.52E-15	1.66	6.87E-14
TSPYL2	TSPY-like 2	ENST00000556808	**1.21**	**3.27E-15**	1.21	2.84E-12	1.46	7.97E-14
TMEM217	transmembrane protein 217	NM_001162900	**0.87**	**1.93E-10**	1.67	5.53E-13	1.85	7.99E-14
CCDC117	coiled-coil domain containing 117	NM_173510	**0.80**	**1.09E-11**	1.24	2.15E-12	1.47	8.43E-14

DUSP16	dual specificity phosphatase 16	NM_030640	**0.68**	**1.86E-11**	1.08	2.11E-12	1.28	9.15E-14
FGF2	fibroblast growth factor 2 (basic)	NM_002006	**0.62**	**2.31E-11**	1.01	1.68E-12	1.16	1.16E-13
RAB23	RAB23. member RAS oncogene family	NM_001278666	**0.96**	**7.49E-13**	1.51	8.40E-14	1.48	1.24E-13
FKBP4	FK506 binding protein 4. 59kDa	NM_002014	**0.98**	**1.37E-14**	1.02	4.80E-12	1.23	1.27E-13
HSPA1A	heat shock 70kDa protein 1A	NM_005345	**1.45**	**1.91E-18**	1.37	6.50E-15	1.17	1.43E-13
IFI44	interferon-induced protein 44	NM_006417	**1.85**	**1.07E-13**	1.35	1.93E-08	0.64	4.14E-04
HSPA1B	heat shock 70kDa protein 1B	NM_005346	**1.43**	**3.60E-18**	1.39	6.61E-15	1.18	1.58E-13
IFIH1	interferon induced with helicase C domain 1	NM_022168	**1.98**	**1.11E-13**	1.43	2.25E-08	0.97	7.57E-06
RP11-762H8.4	novel transcript . sense intronic to WDR61	OTTHUMT00000471376	**1.04**	**8.69E-10**	1.31	6.51E-09	2.31	1.86E-13
PPID	peptidylprolyl isomerase D	NM_005038	**0.71**	**1.59E-10**	0.99	1.86E-10	1.43	1.97E-13
AHSA1	AHA1. activator of heat shock 90kDa protein ATPase homolog 1 (yeast)	NM_012111	**1.05**	**1.15E-14**	1.13	2.45E-12	1.28	2.15E-13
CEBPG	CCAAT/enhancer binding protein (C/EBP). gamma	NM_001252296	**0.59**	**3.90E-09**	1.59	2.23E-14	1.41	2.28E-13
NXT2	nuclear transport factor 2-like export factor 2	NM_001242617	**0.63**	**3.97E-08**	1.11	9.77E-10	1.73	2.38E-13
KBTBD8	kelch repeat and BTB (POZ) domain containing 8	NM_032505	**0.97**	**5.52E-11**	1.49	1.17E-11	1.81	2.85E-13
IRAK2	interleukin-1 receptor-associated kinase 2	NM_001570	**0.90**	**1.63E-10**	1.65	1.28E-12	1.78	2.94E-13
XBP1	X-box binding protein 1	NM_001079539	**0.49**	**2.42E-10**	1.05	9.60E-14	0.98	3.37E-13
ABHD3	abhydrolase domain containing 3	NM_138340	**1.30**	**1.39E-12**	1.58	2.55E-11	1.97	3.65E-13
GBE1	glucan (1.4-alpha-). branching enzyme 1	ENST00000429644	**0.52**	**3.31E-09**	1.03	7.71E-12	1.20	3.85E-13
HSPA1B	heat shock 70kDa protein 1B	NM_005346	**1.25**	**1.30E-17**	1.23	1.68E-14	1.05	3.92E-13
HSPA1A	heat shock 70kDa protein 1A	NM_005345	**1.33**	**3.05E-18**	1.26	1.05E-14	1.04	4.03E-13
TXNL1	thioredoxin-like 1	ENST00000587807	**0.70**	**3.30E-13**	0.60	4.11E-09	0.45	4.37E-07
TRAF6	TNF receptor-associated factor 6. E3 ubiquitin protein ligase	NM_004620	**0.60**	**4.94E-10**	1.07	6.14E-12	1.23	4.08E-13
LRRC8B	leucine rich repeat containing 8 family. member B	NM_001134476	**1.01**	**3.23E-16**	1.03	1.96E-13	1.00	4.13E-13
HECW2	HECT. C2 and WW domain containing E3 ubiquitin protein ligase 2	NM_020760	**0.71**	**4.08E-11**	1.26	6.56E-13	1.28	4.57E-13
RGS2	regulator of G-protein signaling 2	NM_002923	**0.64**	**4.20E-09**	1.23	1.67E-11	1.48	4.72E-13
HSPA1A	heat shock 70kDa protein 1A	NM_005345	**1.33**	**2.81E-18**	1.24	1.08E-14	1.02	4.79E-13
HSPA1A	heat shock 70kDa protein 1A	NM_005345	**1.33**	**2.81E-18**	1.24	1.08E-14	1.02	4.79E-13
HSPA1A	heat shock 70kDa protein 1A	NM_005345	**1.33**	**2.81E-18**	1.24	1.08E-14	1.02	4.79E-13
NOP58	NOP58 ribonucleoprotein	ENST00000264279	**0.49**	**1.73E-10**	0.72	9.14E-11	0.95	5.39E-13
PRKACB	protein kinase. cAMP-dependent. catalytic. beta	NM_001242857	**0.58**	**3.88E-10**	0.78	1.10E-09	1.17	5.64E-13
FST	follistatin	NM_006350	**1.84**	**5.50E-13**	1.60	4.20E-09	1.34	8.17E-08
CXCL8	chemokine (C-X-C motif) ligand 8	NM_000584	**2.28**	**5.88E-17**	2.23	9.11E-14	2.03	5.65E-13
NAV3	neuron navigator 3	NM_001024383	**0.57**	**1.62E-10**	1.01	2.69E-12	1.09	5.75E-13
ATF7IP2	activating transcription factor 7 interacting protein 2	NM_001256160	**1.13**	**6.12E-13**	0.77	2.21E-07	0.46	2.19E-04
HBEGF	heparin-binding EGF-like growth factor	NM_001945	**0.49**	**1.10E-08**	1.02	1.35E-11	1.20	6.46E-13
HSPA1B	heat shock 70kDa protein 1B	NM_005346	**1.47**	**9.72E-18**	1.36	4.56E-14	1.18	7.30E-13

GXYLT2	glucoside xylosyltransferase 2	NM_001080393	**1.08**	**1.76E-08**	1.98	1.95E-10	2.66	7.69E-13
ARG2	arginase 2	NM_001172	**0.94**	**2.30E-13**	1.48	3.27E-14	1.25	8.53E-13
DDX58	DEAD (Asp-Glu-Ala-Asp) box polypeptide 58	NM_014314	**1.18**	**7.85E-13**	1.01	7.11E-09	0.81	2.58E-07
NCOA7	nuclear receptor coactivator 7	NM_001199619	**1.19**	**8.61E-13**	1.27	1.49E-10	0.79	4.38E-07
OTULIN	OTU deubiquitinase with linear linkage specificity	NM_138348	**0.74**	**1.31E-10**	1.17	1.74E-11	1.38	8.75E-13
SDF2L1	stromal cell-derived factor 2-like 1	NM_022044	**1.20**	**9.28E-13**	0.76	8.85E-07	0.32	8.29E-03
AC012668.1	novel transcript	OTTHUMT00000337321	**1.95**	**9.79E-13**	1.32	4.03E-07	0.49	1.37E-02
DEDD2	death effector domain containing 2	NM_001270614	**1.55**	**5.06E-13**	2.16	6.66E-13	2.12	9.04E-13
STX11	syntaxin 11	NM_003764	**0.72**	**3.44E-10**	1.22	1.30E-11	1.40	9.44E-13
HERC6	HECT and RLD domain containing E3 ubiquitin protein ligase family member 6	NM_001165136	**1.84**	**1.17E-12**	1.26	3.79E-07	0.83	1.05E-04
CYLD	cylindromatosis (turban tumor syndrome)	NM_001042355	**0.60**	**2.94E-10**	1.03	1.01E-11	1.16	9.51E-13
DDX60	DEAD (Asp-Glu-Ala-Asp) box polypeptide 60	NM_017631	**1.12**	**1.22E-12**	0.84	9.66E-08	0.61	1.05E-05
MANF	mesencephalic astrocyte-derived neurotrophic factor	ENST00000446668	**1.03**	**1.30E-12**	0.73	2.54E-07	0.47	8.72E-05
STK38L	serine/threonine kinase 38 like	NM_015000	**1.44**	**4.06E-17**	1.67	2.05E-15	1.22	9.69E-13
DNAJC3	DnaJ (Hsp40) homolog. subfamily C. member 3	NM_006260	**0.86**	**1.44E-12**	0.60	3.41E-07	0.11	2.04E-01
TNFRSF10D	tumor necrosis factor receptor superfamily. member 10d. decoy with truncated death domain	NM_003840	**-0.84**	**1.65E-12**	0.02	7.83E-01	0.59	3.25E-07
ISG15	ISG15 ubiquitin-like modifier	NM_005101	**1.45**	**1.83E-12**	1.37	2.97E-09	0.92	1.74E-06
HSPA1B	heat shock 70kDa protein 1B	NM_005346	**1.33**	**1.55E-17**	1.29	3.04E-14	1.07	1.06E-12
FAM46A	family with sequence similarity 46. member A	ENST00000369754	**1.76**	**3.05E-15**	1.82	1.37E-12	1.84	1.14E-12
LYSMD3	LysM. putative peptidoglycan-binding. domain containing 3	NM_198273	**0.82**	**2.09E-12**	0.72	1.19E-08	0.38	1.05E-04
DNAJB6	DnaJ (Hsp40) homolog. subfamily B. member 6	NM_005494	**0.77**	**1.56E-11**	1.24	1.26E-12	1.24	1.22E-12
TRIM26	tripartite motif containing 26	NM_001242783	**0.97**	**6.11E-14**	1.04	1.11E-11	1.17	1.41E-12
XAF1	XIAP associated factor 1	NM_017523	**1.97**	**3.63E-12**	1.96	2.10E-09	1.56	1.01E-07
OAS1	2-5-oligoadenylate synthetase 1. 40/46kDa	NM_001032409	**1.60**	**3.68E-12**	0.59	1.43E-03	0.37	2.97E-02
IFITM1	interferon induced transmembrane protein 1	NM_003641	**1.49**	**3.99E-12**	1.22	5.93E-08	0.99	1.61E-06
IKZF5	IKAROS family zinc finger 5 (Pegasus)	NM_001271840	**0.85**	**1.63E-13**	0.93	1.96E-11	1.07	1.52E-12
ABHD4	abhydrolase domain containing 4	ENST00000544562	**1.12**	**4.21E-12**	0.87	1.37E-07	0.74	1.60E-06
GPBP1	GC-rich promoter binding protein 1	NM_001203246	**0.53**	**1.73E-09**	0.94	2.41E-11	1.08	1.79E-12
CLIC2	chloride intracellular channel 2	NM_001289	**1.10**	**1.05E-10**	1.70	2.13E-11	1.93	1.82E-12
TRIM26	tripartite motif containing 26	NM_001242783	**1.00**	**9.55E-14**	1.09	1.37E-11	1.21	1.90E-12
EIF2AK2	eukaryotic translation initiation factor 2-alpha kinase 2	NM_002759	**0.93**	**4.97E-12**	0.72	1.72E-07	0.66	7.44E-07
IFIT5	interferon-induced protein with tetratricopeptide repeats 5	NM_012420	**1.35**	**5.25E-12**	0.82	7.00E-06	0.75	2.24E-05
TRIM26	tripartite motif containing 26	NM_001242783	**0.96**	**9.89E-14**	1.04	1.67E-11	1.16	2.20E-12
CACYBP	calcyclin binding protein	NM_001007214	**0.89**	**5.80E-13**	1.14	4.23E-12	1.18	2.27E-12

IFI35	interferon-induced protein 35	NM_005533	**1.03**	**7.97E-12**	0.71	1.42E-06	0.46	3.26E-04
UBR2	ubiquitin protein ligase E3 component n-recognin 2	NM_015255	**0.47**	**3.60E-10**	0.67	3.37E-10	0.87	2.29E-12
TRIB3	tribbles pseudokinase 3	uc002wdn.3	**0.83**	**1.68E-08**	1.68	3.13E-11	1.92	2.60E-12
FGF5	fibroblast growth factor 5	NM_004464	**0.85**	**8.97E-12**	0.69	1.69E-07	0.84	5.74E-09
INHBA	inhibin. beta A	NM_002192	**0.84**	**9.16E-12**	0.69	1.09E-07	0.57	1.89E-06
ELL2	elongation factor. RNA polymerase II. 2	NM_012081	**0.83**	**3.86E-14**	1.27	7.84E-15	0.94	2.71E-12
PDE4DIP	phosphodiesterase 4D interacting protein	NM_001198832	**0.85**	**1.25E-10**	1.48	2.57E-12	1.47	2.87E-12
CYB5R1	cytochrome b5 reductase 1	ENST00000482572	**0.76**	**1.07E-11**	0.69	2.79E-08	0.62	1.48E-07
TRIM26	tripartite motif containing 26	NM_001242783	**0.99**	**2.46E-13**	1.13	1.42E-11	1.23	2.88E-12
DNAJA1	DnaJ (Hsp40) homolog. subfamily A. member 1	NM_001539	**0.82**	**2.23E-13**	0.98	5.07E-12	1.01	2.92E-12
JMJD6	jumonji domain containing 6	NM_001081461	**0.59**	**2.88E-09**	0.78	8.85E-09	1.21	3.00E-12
HSPB8	heat shock 22kDa protein 8	NM_014365	**1.26**	**1.30E-11**	0.97	4.48E-07	1.35	1.80E-09
RAB39A	RAB39A. member RAS oncogene family	NM_017516	**1.21**	**3.78E-09**	2.32	1.56E-11	2.53	3.03E-12
MICB	MHC class I polypeptide-related sequence B	NM_001289160	**0.69**	**1.81E-11**	1.16	6.80E-13	1.06	3.71E-12
MERTK	MER proto-oncogene. tyrosine kinase	NM_006343	**0.49**	**5.20E-09**	0.97	1.09E-11	1.02	4.01E-12
SP110	SP110 nuclear body protein	NM_001185015	**1.09**	**1.40E-11**	0.74	2.83E-06	0.74	3.12E-06
UBXN4	UBX domain protein 4	NM_014607	**0.60**	**1.44E-11**	0.46	4.27E-07	0.31	1.07E-04
TRIM26	tripartite motif containing 26	NM_001242783	**0.97**	**1.89E-13**	1.05	3.46E-11	1.17	4.09E-12
TRIM26	tripartite motif containing 26	NM_001242783	**0.97**	**1.89E-13**	1.05	3.46E-11	1.17	4.09E-12
ABL2	ABL proto-oncogene 2. non-receptor tyrosine kinase	NM_001136000	**0.66**	**2.19E-11**	1.06	1.85E-12	1.01	4.58E-12
IFI6	interferon. alpha-inducible protein 6	NM_002038	**1.74**	**1.63E-11**	1.68	1.46E-08	1.39	3.13E-07
MICB	MHC class I polypeptide-related sequence B	NM_001289161	**0.71**	**2.62E-11**	1.13	2.94E-12	1.11	4.58E-12
ATP2C1	ATPase. Ca++ transporting. type 2C. member 1	NM_001001485	**0.51**	**1.09E-09**	0.76	4.95E-10	0.98	4.60E-12
NA	NA	RPTR-AF292560-1	**-2.57**	**1.70E-14**	-2.44	3.61E-11	-2.69	5.69E-12
SEC24A	SEC24 family member A	NM_021982	**0.71**	**1.89E-11**	0.66	3.19E-08	0.36	1.27E-04
MICB	MHC class I polypeptide-related sequence B	NM_005931	**0.58**	**2.18E-10**	1.01	4.82E-12	1.01	5.75E-12
SLC10A7	solute carrier family 10. member 7	uc010ioz.2	**0.94**	**2.25E-11**	0.73	5.54E-07	0.59	1.22E-05
MICB	MHC class I polypeptide-related sequence B	NM_005931	**0.58**	**2.18E-10**	1.01	4.82E-12	1.01	5.75E-12
NRIP3	nuclear receptor interacting protein 3	NM_020645	**0.90**	**8.82E-11**	1.32	4.46E-11	1.47	5.96E-12
SLC33A1	solute carrier family 33 (acetyl-CoA transporter). member 1	NM_001190992	**0.69**	**2.35E-11**	0.43	1.51E-05	0.27	1.79E-03
MICB	MHC class I polypeptide-related sequence B	NM_005931	**0.66**	**1.03E-10**	1.06	9.07E-12	1.08	6.13E-12
NA	NA	RPTR-AY189981-1	**-6.80**	**3.16E-16**	-5.56	1.37E-11	-5.79	6.41E-12
USP18	ubiquitin specific peptidase 18	NM_017414	**1.21**	**2.60E-11**	1.03	1.55E-07	0.78	8.82E-06
SEC24D	SEC24 family member D	NM_014822	**0.56**	**2.61E-11**	0.27	2.99E-04	-0.11	9.95E-02
C3orf38	chromosome 3 open reading frame 38	NM_173824	**0.60**	**5.36E-10**	1.00	2.46E-11	1.07	7.44E-12

PLOD2	procollagen-lysine. 2-oxoglutarate 5-dioxygenase 2	NM_000935	**0.74**	**5.44E-13**	0.95	2.84E-12	0.91	7.45E-12
BRF2	BRF2. RNA polymerase III transcription initiation factor 50 kDa subunit	NM_018310	**0.93**	**3.38E-11**	0.77	3.12E-07	0.65	3.68E-06
DNAJB11	DnaJ (Hsp40) homolog. subfamily B. member 11	ENST00000439351	**0.71**	**3.38E-11**	0.72	1.36E-08	0.36	1.90E-04
STXBP5-AS1	STXBP5 antisense RNA 1	ENST00000433308	**0.71**	**6.13E-08**	2.01	2.22E-13	1.66	8.67E-12
RYBP	RING1 and YY1 binding protein	NM_012234	**0.88**	**2.39E-11**	1.27	1.61E-11	1.31	9.43E-12
STC1	stanniocalcin 1	NM_003155	**-0.56**	**6.17E-10**	-0.94	2.78E-11	-1.00	9.56E-12
ANXA1	annexin A1	NM_000700	**0.43**	**5.67E-09**	0.89	6.71E-12	0.87	9.62E-12
LOC344887	NmrA-like family domain containing 1 pseudogene	NR_033752	**0.83**	**9.97E-13**	1.07	6.33E-12	1.04	1.02E-11
TRIM26	tripartite motif containing 26	ENST00000436219	**0.99**	**1.30E-13**	1.07	2.13E-11	1.11	1.07E-11
ZCCHC6	zinc finger. CCHC domain containing 6	NM_001185059	**0.57**	**3.17E-10**	0.91	3.44E-11	0.97	1.14E-11
SNAI1	snail family zinc finger 1	NM_005985	**0.79**	**3.35E-09**	1.84	3.73E-13	1.54	1.20E-11
RASSF8	Ras association (RalGDS/AF-6) domain family (N-terminal) member 8	NM_001164746	**0.56**	**1.69E-08**	0.90	1.98E-09	1.19	1.22E-11
STAT1	signal transducer and activator of transcription 1. 91kDa	NM_007315	**1.04**	**4.54E-11**	0.56	1.26E-04	0.20	1.04E-01
PARP9	poly (ADP-ribose) polymerase family. member 9	NM_001146102	**1.12**	**4.95E-11**	0.61	1.09E-04	0.23	8.50E-02
UHRF1BP1L	UHRF1 binding protein 1-like	NM_001006947	**1.07**	**4.84E-13**	1.37	3.60E-12	1.28	1.26E-11
NRG1	neuregulin 1	NM_001159995	**0.80**	**5.74E-11**	0.57	4.08E-06	0.45	7.90E-05
ZFAND2A	zinc finger. AN1-type domain 2A	NM_182491	**1.99**	**2.08E-19**	1.84	1.12E-15	1.13	1.36E-11
FERMT3	fermitin family member 3	NM_031471	**-0.65**	**6.25E-11**	-0.66	2.25E-08	-0.37	9.26E-05
STAT2	signal transducer and activator of transcription 2. 113kDa	NM_005419	**0.76**	**6.40E-11**	0.61	9.00E-07	0.66	2.92E-07
ENTPD7	ectonucleoside triphosphate diphosphohydrolase 7	NM_020354	**0.63**	**6.77E-11**	0.60	6.02E-08	0.51	8.23E-07
OSBP	oxysterol binding protein	NM_002556	**0.57**	**6.89E-11**	0.43	2.32E-06	0.08	2.54E-01
HSPA6	heat shock 70kDa protein 6 (HSP70B)	NM_002155	**1.95**	**1.18E-13**	2.17	1.16E-11	2.15	1.38E-11
TRIM26	tripartite motif containing 26	ENST00000415923	**0.99**	**3.56E-13**	1.11	2.71E-11	1.15	1.38E-11
SLFN5	schlafen family member 5	NM_144975	**0.76**	**3.70E-11**	1.27	1.48E-12	1.12	1.58E-11
MICB	MHC class I polypeptide-related sequence B	NM_005931	**0.62**	**2.78E-10**	1.05	9.28E-12	1.02	1.62E-11
MED13	mediator complex subunit 13	NM_005121	**0.51**	**8.74E-11**	0.86	3.30E-12	0.78	1.82E-11
RCAN1	regulator of calcineurin 1	NM_203418	**1.83**	**7.68E-11**	1.46	1.11E-06	0.93	3.00E-04
PDE4DIP	phosphodiesterase 4D interacting protein	NM_001002811	**0.63**	**5.31E-10**	0.91	3.77E-10	1.07	1.92E-11
HSPA9	heat shock 70kDa protein 9 (mortalin)	NM_004134	**0.69**	**4.25E-12**	0.88	3.08E-11	0.90	1.94E-11
SNAP23	synaptosomal-associated protein. 23kDa	NM_003825	**0.41**	**5.26E-09**	0.71	1.90E-10	0.80	2.06E-11
SRP68	signal recognition particle 68kDa	NM_001260502	**0.59**	**8.15E-11**	0.37	3.25E-05	0.39	1.40E-05
SLC5A3	solute carrier family 5 (sodium/myo-inositol cotransporter). member 3	NM_006933	**0.60**	**1.67E-08**	0.93	3.07E-09	1.23	2.16E-11
STARD4	StAR-related lipid transfer (START) domain containing 4	NM_139164	**0.84**	**8.74E-11**	0.48	8.92E-05	0.04	7.01E-01
PDE4DIP	phosphodiesterase 4D interacting protein	NM_001198832	**0.99**	**6.83E-10**	1.73	1.42E-11	1.68	2.38E-11

UBR1	ubiquitin protein ligase E3 component n-recognin 1	NM_174916	**0.57**	**2.62E-11**	0.70	3.09E-10	0.80	2.39E-11
GCNT2	glucosaminyl (N-acetyl) transferase 2. I-branching enzyme (I blood group)	NM_001491	**0.93**	**9.16E-11**	0.98	1.79E-08	0.77	7.03E-07
SLC3A2	solute carrier family 3 (amino acid transporter heavy chain). member 2	NM_001012662	**0.52**	**5.39E-08**	1.18	1.39E-11	1.14	2.52E-11
ALAS1	aminolevulinate. delta-. synthase 1	NM_000688	**0.57**	**6.15E-11**	0.55	4.82E-08	0.85	2.56E-11
HSP90AA1	heat shock protein 90kDa alpha (cytosolic). class A member 1	NM_001017963	**0.89**	**4.90E-14**	0.91	2.19E-11	0.90	2.93E-11
BST2	bone marrow stromal cell antigen 2	AK303593	**1.20**	**1.03E-10**	0.94	1.67E-06	1.11	1.38E-07
RNU6-71P	RNA. U6 small nuclear 71. pseudogene	NR_046940	**2.11**	**7.23E-11**	3.37	7.85E-12	3.14	2.98E-11
AZIN1	antizyme inhibitor 1	NM_015878	**0.72**	**3.87E-11**	0.93	1.99E-10	1.01	3.99E-11
DDIT3	DNA-damage-inducible transcript 3	NM_001195053	**1.92**	**1.77E-14**	2.10	2.74E-12	1.82	4.06E-11
PIGA	phosphatidylinositol glycan anchor biosynthesis. class A	NM_002641	**0.80**	**4.52E-12**	1.12	5.41E-12	1.00	4.27E-11
BRD2	bromodomain containing 2	NM_001113182	**0.49**	**1.16E-10**	0.65	4.13E-10	0.73	4.70E-11
DERL2	derlin 2	NM_016041	**0.61**	**1.09E-10**	0.36	6.98E-05	0.19	1.67E-02
P4HA1	prolyl 4-hydroxylase. alpha polypeptide I	NM_000917	**0.67**	**1.12E-10**	0.58	4.53E-07	0.66	5.67E-08
OAS2	2-5-oligoadenylate synthetase 2. 69/71kDa	NM_002535	**1.32**	**1.15E-10**	0.66	4.36E-04	0.40	1.87E-02
YRDC	yrdC N(6)-threonylcarbamoyltransferase domain containing	NM_024640	**0.67**	**6.52E-12**	0.83	8.03E-11	0.85	4.79E-11
HYOU1	hypoxia up-regulated 1	NM_001130991	**0.77**	**8.18E-14**	0.98	6.17E-13	0.77	4.97E-11
BRD2	bromodomain containing 2	NM_001113182	**0.50**	**1.19E-10**	0.66	3.74E-10	0.73	5.85E-11
BRD2	bromodomain containing 2	NM_001113182	**0.52**	**1.30E-10**	0.67	5.27E-10	0.76	6.14E-11
RNU6-329P	RNA. U6 small nuclear 329. pseudogene	ENST00000459618	**1.59**	**3.51E-09**	2.20	5.18E-09	2.82	6.16E-11
HSPA4	heat shock 70kDa protein 4	NM_002154	**0.78**	**2.06E-13**	0.97	2.32E-12	0.81	6.54E-11
OSGIN1	oxidative stress induced growth inhibitor 1	ENST00000343939	**0.79**	**1.35E-10**	0.49	4.45E-05	0.50	3.66E-05
UGDH	UDP-glucose 6-dehydrogenase	NM_003359	**0.60**	**1.39E-10**	0.36	7.21E-05	0.15	5.18E-02
HMGCS1	3-hydroxy-3-methylglutaryl-CoA synthase 1 (soluble)	NM_001098272	**1.03**	**1.45E-10**	0.75	5.71E-06	0.37	7.64E-03
HSPA5	heat shock 70kDa protein 5 (glucose-regulated protein. 78kDa)	NM_005347	**0.88**	**7.41E-16**	1.06	2.04E-14	0.69	6.60E-11
NA	NA	RPTR-AJ510163-3	**-6.55**	**3.02E-15**	-5.20	1.84E-10	-5.48	7.04E-11
MICB	MHC class I polypeptide-related sequence B	ENST00000458032	**0.57**	**2.57E-09**	0.95	1.20E-10	0.98	7.09E-11
PTGES3	prostaglandin E synthase 3 (cytosolic)	NM_001282601	**0.54**	**3.61E-10**	0.72	9.65E-10	0.83	7.31E-11
PSMD11	proteasome (prosome. macropain) 26S subunit. non-ATPase. 11	NM_001270482	**0.49**	**1.60E-10**	0.42	7.90E-07	0.25	5.17E-04
ULBP3	UL16 binding protein 3	NM_024518	**0.87**	**4.44E-08**	1.31	1.55E-08	1.78	7.47E-11
ME1	malic enzyme 1. NADP(+)-dependent. cytosolic	NM_002395	**0.68**	**7.04E-13**	0.90	2.39E-12	0.75	7.84E-11
RND3	Rho family GTPase 3	NM_001254738	**0.55**	**5.37E-10**	0.84	1.55E-10	0.87	8.40E-11

UBR4	ubiquitin protein ligase E3 component n-recognin 4	NM_020765	0.51	1.64E-10	0.57	1.02E-08	0.30	1.23E-04
SELK	selenoprotein K	ENST00000487571	0.94	1.65E-10	0.77	1.41E-06	0.35	6.10E-03
BRD2	bromodomain containing 2	NM_001291986	0.52	7.94E-11	0.69	2.54E-10	0.73	8.78E-11
NA	NA	NONHSAT098813	1.72	4.11E-14	1.48	4.62E-10	1.63	8.88E-11
MLLT11	myeloid/lymphoid or mixed-lineage leukemia (trithorax homolog. Drosophila); translocated to. 11	NM_006818	0.57	4.04E-12	0.69	7.50E-11	0.68	9.47E-11
SORBS1	sorbin and SH3 domain containing 1	NM_001034954	0.93	1.15E-08	1.67	1.57E-10	1.72	9.56E-11
OTUD7B	OTU deubiquitinase 7B	NM_020205	0.68	1.75E-10	0.72	2.46E-08	0.99	9.58E-11
DYRK3	dual-specificity tyrosine-(Y)-phosphorylation regulated kinase 3	ENST00000367106	0.53	6.04E-09	0.92	1.92E-10	0.95	9.77E-11
PLSCR1	phospholipid scramblase 1	NM_021105	1.02	2.04E-10	0.49	8.40E-04	0.24	7.22E-02
MAFF	v-maf avian musculoaponeurotic fibrosarcoma oncogene homolog F	NM_001161572	0.91	2.06E-10	1.00	1.45E-08	0.83	3.02E-07
AZI2	5-azacytidine induced 2	NM_001271650	0.76	2.10E-10	0.66	6.53E-07	0.85	1.09E-08
NA	NA	TCONS_l2_00014930-XLOC_l2_008285	0.80	8.67E-12	1.05	3.47E-11	0.99	1.03E-10
NA	NA	---	0.94	9.50E-11	1.19	6.98E-10	1.32	1.05E-10
KCTD12	potassium channel tetramerization domain containing 12	NM_138444	-0.64	2.55E-09	-1.25	7.53E-12	-1.08	1.10E-10
VEGFC	vascular endothelial growth factor C	NM_005429	0.74	8.06E-11	0.89	1.45E-09	1.03	1.22E-10
CABLES2	Cdk5 and Abl enzyme substrate 2	NM_031215	0.70	1.24E-09	1.07	2.92E-10	1.13	1.22E-10
TSC22D2	TSC22 domain family. member 2	ENST00000361875	0.79	1.50E-10	1.17	6.80E-11	1.13	1.24E-10
DSEL	dermatan sulfate epimerase-like	NM_032160	0.67	2.56E-10	0.30	1.92E-03	0.35	4.65E-04
GTPBP2	GTP binding protein 2	NM_001286216	0.56	7.47E-08	0.93	5.46E-09	1.15	1.28E-10
SQSTM1	sequestosome 1	NM_001142298	0.59	2.61E-10	0.61	5.53E-08	0.48	1.86E-06
CNST	consortin. connexin sorting protein	NM_152609	0.73	1.42E-09	1.15	1.82E-10	1.17	1.32E-10
TMEM47	transmembrane protein 47	NM_031442	0.47	1.58E-09	0.80	4.99E-11	0.75	1.45E-10
EIF5	eukaryotic translation initiation factor 5	NM_001969	0.65	3.68E-11	0.75	1.44E-09	0.85	1.45E-10
SAMD9	sterile alpha motif domain containing 9	NM_017654	1.03	2.96E-10	0.86	1.73E-06	0.66	5.36E-05
SPTY2D1	SPT2. Suppressor of Ty. domain containing 1 (S. cerevisiae)	NM_194285	0.68	3.12E-10	0.70	7.64E-08	0.63	4.36E-07
SPAG9	sperm associated antigen 9	NM_001130527	0.67	1.00E-10	1.03	1.86E-11	0.92	1.52E-10
SERPINH1	serpin peptidase inhibitor. clade H (heat shock protein 47). member 1. (collagen binding protein 1)	NM_001207014	0.56	1.55E-11	0.71	1.18E-10	0.70	1.58E-10
STIP1	stress-induced phosphoprotein 1	NM_001282652	0.70	1.20E-11	0.69	6.79E-09	0.86	1.66E-10
KDM2A	lysine (K)-specific demethylase 2A	NM_001256405	0.68	7.26E-11	0.99	5.07E-11	0.92	1.72E-10
PPME1	protein phosphatase methylesterase 1	ENST00000535205	0.79	1.18E-11	0.95	2.14E-10	0.96	1.83E-10
ATF3	activating transcription factor 3	NM_001030287	2.75	1.94E-12	3.39	2.52E-11	3.03	1.95E-10
HSPA1L	heat shock 70kDa protein 1-like	NM_005527	0.86	3.68E-10	1.05	4.84E-09	0.83	2.10E-07

RAD9A	RAD9 homolog A (S. pombe)	NM_001243224	**0.86**	**3.88E-10**	0.52	1.16E-04	0.09	4.38E-01
SLC39A14	solute carrier family 39 (zinc transporter). member 14	NM_001128431	**1.06**	**1.21E-12**	1.35	8.98E-12	1.14	2.10E-10
PUS3	pseudouridylate synthase 3	NM_001271985	**0.82**	**3.99E-10**	0.69	1.80E-06	0.80	2.10E-07
HSPB1	heat shock 27kDa protein 1	NM_001540	**0.51**	**2.21E-10**	0.76	8.35E-11	0.72	2.21E-10
ADAMTS18	ADAM metallopeptidase with thrombospondin type 1 motif. 18	NM_199355	**-0.58**	**1.05E-08**	-1.22	9.46E-12	-1.03	2.27E-10
TMEM57	transmembrane protein 57	NM_018202	**0.53**	**4.32E-10**	0.59	2.43E-08	0.25	1.60E-03
SERPINE2	serpin peptidase inhibitor. clade E (nexin. plasminogen activator inhibitor type 1). member 2	NM_001136528	**0.55**	**4.35E-10**	0.74	1.29E-09	0.51	5.36E-07
EPB41	erythrocyte membrane protein band 4.1	NM_001166005	**0.57**	**4.43E-10**	0.66	1.33E-08	0.59	1.06E-07
SELE	selectin E	NM_000450	**1.20**	**4.62E-10**	1.15	3.05E-07	1.09	6.87E-07
IFI44L	interferon-induced protein 44-like	NM_006820	**3.05**	**4.70E-10**	2.49	3.34E-06	1.30	3.57E-03
AJUBA	ajuba LIM protein	NM_032876	**-0.57**	**4.80E-10**	0.00	9.95E-01	0.36	6.66E-05
TGOLN2	trans-golgi network protein 2	NM_001206840	**-0.57**	**4.80E-10**	-0.62	3.85E-08	-0.38	4.88E-05
HERC4	HECT and RLD domain containing E3 ubiquitin protein ligase 4	NM_001278185	**0.45**	**7.73E-08**	0.64	6.49E-08	0.89	2.29E-10
OAS3	2-5-oligoadenylate synthetase 3. 100kDa	AB044545	**1.23**	**5.01E-10**	0.98	4.50E-06	0.59	1.39E-03
ACVRL1	activin A receptor type II-like 1	NM_000020	**-0.61**	**5.04E-10**	-0.56	6.43E-07	-0.44	1.61E-05
UBE2L6	ubiquitin-conjugating enzyme E2L 6	NM_198183	**0.65**	**5.06E-10**	0.07	4.09E-01	-0.03	7.25E-01
DHDDS	dehydrodolichyl diphosphate synthase	NM_001243564	**0.51**	**5.25E-10**	0.47	4.91E-07	0.33	5.77E-05
DNAJC24	DnaJ (Hsp40) homolog. subfamily C. member 24	ENST00000526042	**0.57**	**4.92E-08**	0.98	2.06E-09	1.10	2.63E-10
MX1	myxovirus (influenza virus) resistance 1. interferon-inducible protein p78 (mouse)	NM_001144925	**2.86**	**5.34E-10**	2.46	1.67E-06	1.59	3.49E-04
IGHJ1	immunoglobulin heavy joining 1	ENST00000390565	**-1.44**	**3.45E-09**	-1.76	3.67E-08	-2.34	2.79E-10
PHACTR4	phosphatase and actin regulator 4	NM_001048183	**0.57**	**5.37E-10**	0.66	1.64E-08	0.39	3.78E-05
FAM219A	family with sequence similarity 219. member A	NM_001184940	**0.69**	**5.19E-09**	1.14	3.65E-10	1.14	3.54E-10
FBXO30	F-box protein 30	NM_032145	**0.57**	**5.65E-10**	0.57	2.06E-07	0.39	3.95E-05
STRIP2	striatin interacting protein 2	NM_001134336	**0.73**	**5.71E-10**	0.47	7.36E-05	0.34	1.95E-03
CSRNP1	cysteine-serine-rich nuclear protein 1	NM_033027	**0.76**	**5.94E-09**	1.30	1.91E-10	1.25	3.90E-10
SAMD4B	sterile alpha motif domain containing 4B	NM_018028	**0.64**	**1.15E-09**	1.06	6.32E-11	0.95	4.22E-10
IRF9	interferon regulatory factor 9	NM_006084	**0.72**	**6.20E-10**	0.73	1.80E-07	0.56	8.83E-06
SIK1	salt-inducible kinase 1	NM_173354	**0.98**	**1.04E-11**	1.60	6.61E-13	1.12	4.44E-10
CCDC174	coiled-coil domain containing 174	NM_016474	**0.74**	**4.23E-11**	0.94	3.30E-10	0.92	4.67E-10
SEL1L	sel-1 suppressor of lin-12-like (C. elegans)	NM_005065	**0.53**	**6.50E-10**	0.31	2.28E-04	0.09	2.15E-01
IFIT3	interferon-induced protein with tetratricopeptide repeats 3	NM_001031683	**1.32**	**6.57E-10**	1.26	4.20E-07	1.04	6.63E-06
CTC-436P18.3	putative novel transcript	OTTHUMT00000368082	**-1.17**	**6.64E-10**	-0.35	3.61E-02	-0.26	1.07E-01
TYW3	tRNA-yW synthesizing protein 3 homolog (S. cerevisiae)	NM_001162916	**0.62**	**3.52E-08**	0.98	4.88E-09	1.12	4.87E-10

TMF1	TATA element modulatory factor 1	NM_007114	0.63	6.96E-10	0.49	8.09E-06	0.40	1.00E-04
SEC23B	Sec23 homolog B (S. cerevisiae)	NM_001172745	0.71	7.43E-10	0.31	3.49E-03	0.30	4.60E-03
HSPB1	heat shock 27kDa protein 1	NM_001540	0.48	1.82E-10	0.61	1.34E-09	0.64	5.91E-10
SPEN	spen family transcriptional repressor	NM_015001	0.70	1.27E-10	1.04	5.18E-11	0.91	6.34E-10
IDE	insulin-degrading enzyme	NM_001165946	0.49	8.46E-10	0.29	2.97E-04	0.22	3.80E-03
NA	NA	---	0.86	2.48E-08	1.17	4.42E-08	1.51	6.42E-10
MICA	MHC class I polypeptide-related sequence A	ENST00000415525	0.53	2.47E-09	0.83	3.46E-10	0.80	7.01E-10
KIAA0513	16q24.1	NM_001286565	1.60	5.92E-13	1.82	3.85E-11	1.55	7.15E-10
PER2	period circadian clock 2	NM_022817	0.74	9.31E-10	0.68	1.07E-06	0.13	1.95E-01
RNA5SP422	RNA. 5S ribosomal pseudogene 422	ENST00000516778	-1.23	9.59E-10	-1.03	3.97E-06	-1.79	5.64E-10
RP11-143M1.4	putative novel transcript	OTTHUMT00000051455	1.34	9.76E-10	1.78	2.99E-09	1.09	5.88E-06
UBB	ubiquitin B	NM_018955	0.52	9.98E-10	0.68	3.82E-09	0.56	9.67E-08
NDEL1	nudE neurodevelopment protein 1-like 1	NM_001025579	0.48	1.06E-09	0.47	4.25E-07	0.32	7.70E-05
FEM1B	fem-1 homolog b (C. elegans)	NM_015322	0.63	2.29E-10	0.91	1.85E-10	0.84	7.38E-10
TP53BP2	tumor protein p53 binding protein 2	NM_001031685	0.75	1.08E-10	1.02	2.25E-10	0.95	8.61E-10
KCTD5	potassium channel tetramerization domain containing 5	NM_018992	0.57	7.59E-11	0.62	7.52E-09	0.70	9.37E-10
IFIT1	interferon-induced protein with tetratricopeptide repeats 1	AK092813	2.52	1.17E-09	2.35	1.01E-06	1.07	5.22E-03
RPS6KA3	ribosomal protein S6 kinase. 90kDa. polypeptide 3	NM_004586	0.54	6.10E-10	0.72	1.65E-09	0.73	1.08E-09
LURAP1L	leucine rich adaptor protein 1-like	NM_203403	0.65	3.81E-08	0.95	2.48E-08	1.14	1.12E-09
NAP1L2	nucleosome assembly protein 1-like 2	NM_021963	0.95	1.25E-09	0.98	2.63E-07	0.89	1.10E-06
MIS12	MIS12 kinetochore complex component	NM_001258217	0.70	1.26E-09	0.29	7.41E-03	-0.02	8.76E-01
HSP90AB3P	heat shock protein 90kDa alpha (cytosolic). class B member 3. pseudogene	ENST00000505987	0.66	1.30E-09	0.65	4.67E-07	0.80	1.61E-08
C8orf4	chromosome 8 open reading frame 4	NM_020130	0.71	1.40E-09	0.65	1.67E-06	0.27	1.13E-02
CCDC59	coiled-coil domain containing 59	NM_014167	0.50	2.17E-09	0.86	7.63E-11	0.74	1.16E-09
CPA4	carboxypeptidase A4	NM_001163446	-0.65	4.33E-09	-1.23	2.31E-11	-0.99	1.22E-09
ARFGAP3	ADP-ribosylation factor GTPase activating protein 3	NM_001142293	0.50	1.51E-09	0.38	2.12E-05	0.26	1.32E-03
MORC4	MORC family CW-type zinc finger 4	NM_001085354	0.66	1.87E-10	0.75	8.07E-09	0.84	1.24E-09
PARP14	poly (ADP-ribose) polymerase family. member 14	NM_017554	0.88	1.57E-09	0.53	3.03E-04	0.33	1.35E-02
PSMC4	proteasome (prosome. macropain) 26S subunit. ATPase. 4	NM_006503	0.74	3.40E-12	0.92	3.74E-11	0.76	1.27E-09
UBB	ubiquitin B	NM_018955	0.60	1.62E-09	0.75	1.45E-08	0.60	5.17E-07
UBQLN1	ubiquilin 1	NM_013438	0.55	1.65E-09	0.58	2.20E-07	0.55	4.25E-07
IPPK	inositol 1.3.4.5.6-pentakisphosphate 2-kinase	NM_022755	0.67	1.97E-10	0.96	1.74E-10	0.85	1.32E-09
TLR4	toll-like receptor 4	NM_003266	0.55	1.72E-09	0.39	5.46E-05	0.02	8.31E-01

EDA2R	ectodysplasin A2 receptor	NM_001199687	**0.69**	**3.95E-09**	1.18	1.26E-10	1.03	1.42E-09
SAMHD1	SAM domain and HD domain 1	NM_015474	**0.92**	**1.74E-09**	0.54	4.19E-04	0.14	2.84E-01
ENC1	ectodermal-neural cortex 1 (with BTB domain)	NM_001256574	**-0.58**	**1.76E-09**	-0.69	3.00E-08	-0.57	5.60E-07
COG3	component of oligomeric golgi complex 3	NM_031431	**0.61**	**1.83E-09**	0.59	9.17E-07	0.51	8.18E-06
FXR1	fragile X mental retardation. autosomal homolog 1	NM_001013438	**0.50**	**1.55E-09**	0.69	2.19E-09	0.70	1.43E-09
RP11-88H10.2	novel transcript	OTTHUMT00000355090	**1.49**	**1.88E-09**	1.23	8.43E-06	1.64	1.19E-07
KIF1B	kinesin family member 1B	NM_015074	**0.48**	**1.89E-09**	0.57	3.06E-08	0.23	2.28E-03
DDX60L	DEAD (Asp-Glu-Ala-Asp) box polypeptide 60-like	NM_001012967	**0.73**	**1.91E-09**	0.34	3.11E-03	0.20	5.98E-02
TTLL7	tubulin tyrosine ligase-like family. member 7	NM_024686	**0.84**	**2.04E-09**	0.89	2.42E-07	0.82	8.84E-07
DNAJC2	DnaJ (Hsp40) homolog. subfamily C. member 2	NM_001129887	**0.56**	**2.06E-09**	0.42	2.93E-05	0.29	1.64E-03
LOC100129361	chromosome X open reading frame 69-like	NM_001271592	**0.64**	**2.07E-09**	0.40	2.60E-04	0.46	5.41E-05
IFRD1	interferon-related developmental regulator 1	NM_001007245	**0.64**	**7.77E-09**	0.96	2.49E-09	0.99	1.50E-09
LIN54	lin-54 homolog (C. elegans)	NM_001115007	**0.61**	**2.13E-09**	0.44	4.37E-05	0.37	3.50E-04
ERRFI1	ERBB receptor feedback inhibitor 1	ENST00000467067	**0.38**	**8.01E-08**	1.01	1.11E-12	0.68	1.66E-09
DYNC1I1	dynein. cytoplasmic 1. intermediate chain 1	NM_001278421	**0.91**	**2.17E-09**	1.04	8.14E-08	0.81	3.14E-06
ITGB8	integrin. beta 8	NM_002214	**-0.57**	**2.19E-09**	-0.33	5.13E-04	0.19	3.14E-02
RIN2	Ras and Rab interactor 2	NM_001242581	**-0.56**	**2.22E-09**	-0.37	1.32E-04	-0.17	3.99E-02
USP47	ubiquitin specific peptidase 47	NM_001282659	**0.53**	**2.28E-09**	0.56	2.91E-07	0.60	1.17E-07
PSMD3	proteasome (prosome. macropain) 26S subunit. non-ATPase. 3	NM_002809	**0.68**	**2.29E-09**	0.53	1.98E-05	0.50	4.20E-05
IRF7	interferon regulatory factor 7	NM_001572	**0.87**	**2.46E-09**	0.91	3.87E-07	0.81	1.95E-06
C18orf25	chromosome 18 open reading frame 25	NM_001008239	**0.84**	**1.70E-11**	0.99	5.25E-10	0.92	1.86E-09
PHF1	PHD finger protein 1	NM_002636	**0.55**	**2.48E-09**	0.67	3.10E-08	0.54	9.46E-07
SNX3	sorting nexin 3	NM_003795	**0.52**	**2.50E-09**	0.54	3.59E-07	0.64	2.55E-08
ITCH	itchy E3 ubiquitin protein ligase	NM_001257137	**0.48**	**2.52E-09**	0.58	3.67E-08	0.51	3.54E-07
BIRC2	baculoviral IAP repeat containing 2	NM_001166	**0.61**	**1.55E-10**	0.92	4.97E-11	0.75	1.86E-09
NA	NA	---	**0.65**	**1.61E-08**	1.05	1.85E-09	1.04	2.02E-09
PSMC6	proteasome (prosome. macropain) 26S subunit. ATPase. 6	NM_002806	**0.66**	**2.61E-09**	0.57	5.99E-06	0.63	1.39E-06
PSMD6	proteasome (prosome. macropain) 26S subunit. non-ATPase. 6	NM_001271779	**0.58**	**2.64E-09**	0.48	8.45E-06	0.44	2.86E-05
SAMD9L	sterile alpha motif domain containing 9-like	NM_152703	**0.72**	**2.74E-09**	0.42	5.58E-04	0.33	4.80E-03
BRD2	bromodomain containing 2	NM_001113182	**0.52**	**2.81E-09**	0.57	1.58E-07	0.68	9.86E-09
LARP4	La ribonucleoprotein domain family. member 4	NM_001170803	**0.71**	**2.20E-10**	0.81	9.24E-09	0.88	2.05E-09
DCP1A	decapping mRNA 1A	NM_001290204	**0.51**	**2.88E-09**	0.70	4.12E-09	0.58	8.91E-08
ALS2	amyotrophic lateral sclerosis 2 (juvenile)	NM_020919	**0.51**	**3.08E-09**	0.44	7.26E-06	0.45	4.18E-06
SOD1	superoxide dismutase 1. soluble	NM_000454	**0.50**	**3.25E-09**	0.70	3.69E-09	0.52	5.04E-07

GABARAPL1	GABA(A) receptor-associated protein like 1	ENST00000535576	**0.84**	**3.31E-09**	0.74	5.53E-06	0.32	1.68E-02
RP4-791M13.3	novel transcript	OTTHUMT00000384603	**0.81**	**1.55E-08**	1.35	8.35E-10	1.28	2.06E-09
HSPB1P1	heat shock 27kDa protein 1 pseudogene 1	ENST00000423240	**0.37**	**3.96E-09**	0.53	3.79E-09	0.54	2.28E-09
NFE2L2	nuclear factor. erythroid 2-like 2	NM_001145412	**0.46**	**3.35E-09**	0.65	3.87E-09	0.51	1.75E-07
SLC37A1	solute carrier family 37 (glucose-6-phosphate transporter). member 1	NM_018964	**0.50**	**4.10E-08**	0.71	3.51E-08	0.83	2.29E-09
BRPF3	bromodomain and PHD finger containing. 3	NM_015695	**0.68**	**4.66E-09**	0.97	3.69E-09	1.00	2.33E-09
ZNF229	zinc finger protein 229	NM_014518	**0.77**	**3.73E-09**	0.83	3.39E-07	0.67	6.59E-06
TBC1D4	TBC1 domain family. member 4	NM_001286658	**0.72**	**7.90E-10**	0.95	2.90E-09	0.96	2.34E-09
IPMK	inositol polyphosphate multikinase	NM_152230	**0.60**	**5.30E-08**	1.06	1.19E-09	1.01	2.41E-09
MICA	MHC class I polypeptide-related sequence A	ENST00000400325	**0.53**	**2.16E-08**	0.92	7.06E-10	0.85	2.47E-09
MICA	MHC class I polypeptide-related sequence A	ENST00000400325	**0.53**	**2.16E-08**	0.92	7.06E-10	0.85	2.47E-09
ZNF317	zinc finger protein 317	NM_001190791	**0.54**	**1.28E-08**	0.73	3.35E-08	0.84	2.60E-09
TAF7	TAF7 RNA polymerase II. TATA box binding protein (TBP)-associated factor. 55kDa	NM_005642	**0.49**	**4.30E-09**	0.45	3.09E-06	0.75	9.48E-10
ATG5	autophagy related 5	NM_001286106	**0.50**	**1.70E-08**	0.78	3.03E-09	0.78	2.85E-09
WAPAL	wings apart-like homolog (Drosophila)	AB065003	**0.47**	**4.34E-09**	0.40	1.14E-05	0.48	8.41E-07
PLAA	phospholipase A2-activating protein	NM_001031689	**0.80**	**1.65E-14**	0.73	6.74E-11	0.59	2.91E-09
VPS37A	vacuolar protein sorting 37 homolog A (S. cerevisiae)	NM_001145152	**0.54**	**4.38E-09**	0.66	3.89E-08	0.49	4.34E-06
BAG2	BCL2-associated athanogene 2	NM_004282	**0.71**	**4.62E-09**	0.57	2.10E-05	0.70	1.49E-06
ACBD3	acyl-CoA binding domain containing 3	NM_022735	**0.75**	**6.99E-11**	0.88	1.93E-09	0.86	2.97E-09
PDIA4	protein disulfide isomerase family A. member 4	NM_004911	**0.44**	**4.67E-09**	0.36	1.50E-05	0.14	3.80E-02
NUCB2	nucleobindin 2	ENST00000323688	**0.57**	**4.69E-09**	0.41	9.03E-05	0.27	3.89E-03
ORC3	origin recognition complex. subunit 3	NM_001197259	**0.52**	**5.08E-09**	0.20	1.49E-02	0.02	7.58E-01
FAM43A	family with sequence similarity 43. member A	NM_153690	**-0.49**	**5.10E-09**	-0.45	4.40E-06	-0.34	1.53E-04
ELL	elongation factor RNA polymerase II	NM_006532	**0.66**	**5.99E-08**	1.09	4.43E-09	1.11	2.99E-09
SLC7A2	solute carrier family 7 (cationic amino acid transporter. y+ system). member 2	NM_001008539	**0.76**	**4.21E-10**	1.37	5.33E-12	0.96	3.31E-09
HMOX1	heme oxygenase (decycling) 1	ENST00000216117	**0.61**	**5.21E-09**	0.75	5.84E-08	0.29	4.10E-03
GORAB	golgin. RAB6-interacting	NM_001146039	**0.62**	**5.24E-09**	0.65	6.23E-07	0.84	1.08E-08
RNU6-531P	RNA. U6 small nuclear 531. pseudogene	ENST00000516694	**1.71**	**1.32E-11**	1.97	5.54E-10	1.77	3.42E-09
ZNF484	zinc finger protein 484	NM_001007101	**0.71**	**5.45E-09**	0.82	1.70E-07	0.86	7.89E-08
TAF2	TAF2 RNA polymerase II. TATA box binding protein (TBP)-associated factor. 150kDa	NM_003184	**0.45**	**5.50E-09**	0.53	1.13E-07	0.57	4.01E-08
CRY1	cryptochrome circadian clock 1	NM_004075	**1.06**	**2.39E-13**	1.15	3.83E-11	0.89	3.66E-09
P4HA2	prolyl 4-hydroxylase. alpha polypeptide II	ENST00000401867	**0.62**	**1.59E-10**	0.74	3.34E-09	0.74	3.77E-09

FNIP1	folliculin interacting protein 1	NM_001008738	0.55	8.39E-10	0.70	5.64E-09	0.71	4.25E-09
ALDH2	aldehyde dehydrogenase 2 family (mitochondrial)	NM_000690	0.48	8.60E-09	0.73	2.33E-09	0.70	4.42E-09
PSMA5	proteasome (prosome. macropain) subunit. alpha type. 5	NM_001199772	0.52	6.23E-09	0.53	1.09E-06	0.35	1.95E-04
GEM	GTP binding protein overexpressed in skeletal muscle	NM_005261	1.42	6.32E-09	1.48	7.71E-07	1.33	3.50E-06
DNAJB4	DnaJ (Hsp40) homolog. subfamily B. member 4	NM_007034	0.66	6.46E-09	0.48	9.95E-05	0.36	1.55E-03
KCMF1	potassium channel modulatory factor 1	NM_020122	0.65	8.39E-12	0.76	2.34E-10	0.64	5.10E-09
FILIP1L	filamin A interacting protein 1-like	NM_001042459	0.73	6.67E-09	0.85	1.48E-07	0.84	1.70E-07
HSPA13	heat shock protein 70kDa family. member 13	NM_006948	0.66	7.78E-11	0.70	1.22E-08	0.73	5.53E-09
MX2	myxovirus (influenza virus) resistance 2 (mouse)	NM_002463	1.41	6.80E-09	1.06	6.89E-05	0.53	2.09E-02
SLC25A33	solute carrier family 25 (pyrimidine nucleotide carrier). member 33	NM_032315	0.63	6.83E-09	0.69	4.27E-07	0.80	4.03E-08
DOCK10	dedicator of cytokinesis 10	NM_001290263	0.47	6.85E-09	0.63	1.53E-08	0.48	1.00E-06
ATP2A2	ATPase. Ca++ transporting. cardiac muscle. slow twitch 2	NM_001681	0.52	6.92E-09	0.38	1.04E-04	0.19	2.52E-02
CLINT1	clathrin interactor 1	NM_001195555	0.41	6.95E-09	0.49	1.03E-07	0.24	7.98E-04
GBP1	guanylate binding protein 1. interferon-inducible	AB208912	0.81	7.08E-09	0.52	3.68E-04	0.37	6.53E-03
SYNM	synemin. intermediate filament protein	NM_015286	0.64	7.10E-09	0.76	1.08E-07	0.70	4.04E-07
SDE2	SDE2 telomere maintenance homolog (S. pombe)	NM_152608	1.17	1.99E-14	0.81	9.37E-09	0.84	5.71E-09
AHI1	Abelson helper integration site 1	NM_001134830	0.51	7.66E-09	0.58	2.27E-07	0.56	4.40E-07
LRIG1	leucine-rich repeats and immunoglobulin-like domains 1	ENST00000273261	0.41	7.26E-08	0.70	2.99E-09	0.67	6.26E-09
NA	NA	ENST00000615674	0.62	7.83E-09	0.06	5.64E-01	-0.61	2.40E-06
ANXA7	annexin A7	NM_001156	0.43	8.06E-09	0.39	8.03E-06	0.28	3.95E-04
EIF2AK3	eukaryotic translation initiation factor 2-alpha kinase 3	NM_004836	0.48	8.18E-09	0.35	1.18E-04	-0.25	2.92E-03
SYVN1	synovial apoptosis inhibitor 1. synoviolin	NM_032431	0.59	8.53E-09	0.43	1.02E-04	0.44	7.46E-05
FNDC3A	fibronectin type III domain containing 3A	NM_001079673	0.70	4.89E-13	0.94	1.22E-12	0.59	6.48E-09
APOOL	apolipoprotein O-like	NM_198450	0.55	8.69E-09	0.57	9.49E-07	0.62	3.14E-07
LARP6	La ribonucleoprotein domain family. member 6	NM_018357	0.65	8.84E-09	0.95	5.54E-09	0.71	5.30E-07
UBC	ubiquitin C	ENST00000536769	1.04	7.82E-12	1.27	1.30E-10	1.01	6.97E-09
OSER1	oxidative stress responsive serine-rich 1	NM_016470	0.79	1.07E-10	1.00	9.46E-10	0.89	7.10E-09
C5orf51	chromosome 5 open reading frame 51	NM_175921	0.57	9.25E-09	0.50	1.33E-05	0.51	8.55E-06
CXCR4	chemokine (C-X-C motif) receptor 4	NM_001008540	-0.62	9.29E-09	-0.01	8.93E-01	0.41	3.51E-04
DTX3L	deltex 3 like. E3 ubiquitin ligase	NM_138287	0.68	9.29E-09	0.35	3.67E-03	0.34	4.35E-03
CEBPB	CCAAT/enhancer binding protein (C/EBP). beta	NM_001285878	0.68	8.96E-09	1.40	1.21E-11	0.97	8.37E-09
AIFM2	apoptosis-inducing factor. mitochondrion-associated. 2	NM_001198696	0.66	9.78E-09	0.84	5.71E-08	0.75	3.78E-07

ZNF267	zinc finger protein 267	NM_001265588	0.64	1.78E-08	0.86	4.61E-08	0.95	8.52E-09
ATP13A3	ATPase type 13A3	NM_024524	0.46	1.01E-08	0.65	1.03E-08	0.55	1.32E-07
BLOC1S2	biogenesis of lysosomal organelles complex-1. subunit 2	NM_001001342	0.52	1.04E-08	0.66	7.60E-08	0.62	1.92E-07
UBR3	ubiquitin protein ligase E3 component n-recognin 3 (putative)	ENST00000272793	0.77	3.55E-13	0.74	4.82E-10	0.62	9.23E-09
PSMD14	proteasome (prosome. macropain) 26S subunit. non-ATPase. 14	NM_005805	0.72	9.29E-12	0.83	4.72E-10	0.70	9.45E-09
CPT1A	carnitine palmitoyltransferase 1A (liver)	NM_001031847	-0.49	9.69E-09	-0.78	1.14E-09	-0.69	9.64E-09
PRR3	proline rich 3	NM_001077497	0.59	1.15E-08	0.81	2.10E-08	0.71	1.64E-07
PRR3	proline rich 3	NM_001077497	0.59	1.15E-08	0.81	2.10E-08	0.71	1.64E-07
PRR3	proline rich 3	NM_001077497	0.59	1.15E-08	0.81	2.10E-08	0.71	1.64E-07
ELOVL5	ELOVL fatty acid elongase 5	NM_001242828	0.47	1.15E-08	0.50	1.28E-06	0.23	5.36E-03
ARL5B	ADP-ribosylation factor-like 5B	NM_178815	0.58	1.20E-08	0.39	3.49E-04	0.22	2.54E-02
ATP6V1B2	ATPase. H+ transporting. lysosomal 56/58kDa. V1 subunit B2	ENST00000523482	0.40	1.20E-08	0.46	4.16E-07	0.34	2.85E-05
GOLGA5	golgin A5	NM_005113	0.45	1.24E-08	0.36	4.89E-05	0.21	6.69E-03
OASL	2-5-oligoadenylate synthetase-like	NM_001261825	0.69	1.25E-08	0.83	1.97E-07	0.66	4.76E-06
TULP3	tubby like protein 3	NM_001160408	0.40	2.08E-08	0.78	7.17E-11	0.59	1.02E-08
SLC19A2	solute carrier family 19 (thiamine transporter). member 2	NM_006996	1.30	7.50E-17	1.32	5.41E-14	0.68	1.07E-08
HSD17B7P2	hydroxysteroid (17-beta) dehydrogenase 7 pseudogene 2	NR_003086	0.91	1.30E-08	1.24	2.66E-08	0.61	3.51E-04
IDI1	isopentenyl-diphosphate delta isomerase 1	NM_004508	0.46	1.36E-08	0.05	4.70E-01	-0.02	7.97E-01
EAF1	ELL associated factor 1	NM_033083	0.62	1.36E-08	0.82	4.34E-08	0.72	3.35E-07
ZBTB21	zinc finger and BTB domain containing 21	NM_001098402	0.79	3.33E-10	0.85	3.61E-08	0.91	1.14E-08
TRIM8	tripartite motif containing 8	NM_030912	-0.58	1.42E-08	-0.66	5.44E-07	-0.64	7.40E-07
MBNL2	muscleblind-like splicing regulator 2	NM_144778	0.47	1.43E-08	0.79	7.50E-10	0.57	1.54E-07
05-mar	membrane-associated ring finger (C3HC4) 5	ENST00000467521	0.43	1.45E-08	0.62	9.46E-09	0.62	1.17E-08
PNPLA8	patatin-like phospholipase domain containing 8	NM_001256007	0.56	1.50E-08	0.54	4.59E-06	0.52	7.69E-06
POLR3F	polymerase (RNA) III (DNA directed) polypeptide F. 39 kDa	NM_001282526	0.57	1.51E-08	0.52	1.24E-05	0.51	1.26E-05
FAM124B	family with sequence similarity 124B	NM_024785	-0.68	4.29E-10	-0.98	3.16E-10	-0.79	1.22E-08
RAB21	RAB21. member RAS oncogene family	NM_014999	0.51	1.55E-08	0.54	1.65E-06	0.51	3.30E-06
F2RL1	coagulation factor II (thrombin) receptor-like 1	NM_005242	-0.55	1.55E-08	-0.53	6.43E-06	-0.24	1.20E-02
RSAD2	radical S-adenosyl methionine domain containing 2	NM_080657	0.94	1.55E-08	0.77	4.04E-05	0.43	8.64E-03
PRICKLE1	prickle homolog 1 (Drosophila)	NM_001144881	-0.42	1.56E-08	-0.59	1.95E-08	-0.50	2.41E-07
SETD5	SET domain containing 5	NM_001080517	0.38	5.54E-08	0.76	1.16E-10	0.58	1.26E-08
PALMD	palmdelphin	NM_017734	-0.57	1.63E-08	-0.84	8.00E-09	-0.67	3.48E-07
TES	testis derived transcript (3 LIM domains)	NM_015641	0.61	1.63E-08	0.88	1.36E-08	0.56	1.12E-05

BTG3	BTG family. member 3	NM_001130914	**0.78**	**1.64E-08**	0.98	1.04E-07	0.93	2.41E-07
LARP1B	La ribonucleoprotein domain family. member 1B	NM_018078	**0.61**	**1.64E-08**	0.60	3.88E-06	0.40	4.51E-04
SUPV3L1	suppressor of var1. 3-like 1 (S. cerevisiae)	NM_003171	**0.51**	**1.67E-08**	0.57	5.56E-07	0.61	2.22E-07
CCT4	chaperonin containing TCP1. subunit 4 (delta)	NM_001256721	**0.46**	**1.24E-09**	0.59	6.28E-09	0.57	1.27E-08
PI4K2B	phosphatidylinositol 4-kinase type 2 beta	NM_018323	**0.68**	**1.47E-09**	0.77	6.20E-08	0.85	1.28E-08
EPSTI1	epithelial stromal interaction 1 (breast)	NM_001002264	**1.52**	**1.10E-13**	1.53	6.47E-11	1.13	1.43E-08
PDE4DIP	phosphodiesterase 4D interacting protein	NM_001198834	**0.86**	**6.52E-09**	1.18	1.10E-08	1.17	1.43E-08
AHNAK2	AHNAK nucleoprotein 2	NM_138420	**-0.55**	**1.61E-10**	-0.89	1.44E-11	-0.60	1.62E-08
MAFG	v-maf avian musculoaponeurotic fibrosarcoma oncogene homolog G	NM_032711	**0.59**	**1.86E-09**	0.77	6.59E-09	0.73	1.69E-08
HELZ2	helicase with zinc finger 2. transcriptional coactivator	NM_001037335	**1.05**	**1.80E-08**	1.28	1.77E-07	0.92	1.85E-05
LMAN1	lectin. mannose-binding. 1	NM_005570	**0.42**	**1.81E-08**	0.42	3.83E-06	0.36	2.76E-05
TSC22D2	TSC22 domain family. member 2	ENST00000361875	**0.65**	**1.82E-08**	0.80	1.40E-07	0.67	2.39E-06
XXYLT1-AS2	XXYLT1 antisense RNA 2	NR_102710	**-0.60**	**1.83E-08**	-1.00	1.16E-09	-0.57	7.30E-06
SARAF	store-operated calcium entry-associated regulatory factor	NM_016127	**0.39**	**1.83E-08**	0.47	1.90E-07	0.23	1.65E-03
SLC41A2	solute carrier family 41 (magnesium transporter). member 2	NM_032148	**0.64**	**1.85E-08**	0.72	7.10E-07	0.59	1.16E-05
LOC100506123	uncharacterized LOC100506123	NR_040097	**-0.57**	**1.89E-08**	-0.24	1.61E-02	-0.03	7.38E-01
ZUFSP	zinc finger with UFM1-specific peptidase domain	NM_145062	**0.62**	**1.89E-08**	0.67	1.44E-06	0.91	1.11E-08
TRIM14	tripartite motif containing 14	NM_014788	**0.75**	**1.90E-08**	0.54	2.14E-04	0.23	7.01E-02
RNF111	ring finger protein 111	NM_001270528	**0.43**	**1.94E-08**	0.41	7.14E-06	0.23	3.27E-03
RLF	rearranged L-myc fusion	NM_012421	**0.70**	**1.95E-08**	0.77	9.43E-07	1.14	1.83E-09
RP1-66C13.3	lectin. galactoside-binding. soluble. 9 (LGALS9) pseudogene	OTTHUMT00000445281	**0.85**	**1.98E-08**	0.20	1.51E-01	-0.03	8.04E-01
BIRC3	baculoviral IAP repeat containing 3	ENST00000263464	**1.30**	**1.99E-08**	1.45	8.10E-07	1.93	8.19E-09
CRY2	cryptochrome circadian clock 2	NM_001127457	**0.55**	**2.07E-08**	0.23	1.66E-02	0.13	1.64E-01
ARID5A	AT rich interactive domain 5A (MRF1-like)	NM_212481	**1.01**	**5.46E-13**	1.01	3.59E-10	0.80	1.93E-08
KLF4	Kruppel-like factor 4 (gut)	uc004bdh.3	**0.57**	**2.08E-08**	0.69	2.98E-07	0.62	1.27E-06
MPHOSPH10	M-phase phosphoprotein 10 (U3 small nucleolar ribonucleoprotein)	NM_005791	**0.43**	**2.10E-08**	-0.05	4.56E-01	-0.12	1.03E-01
CCDC186	coiled-coil domain containing 186	NM_018017	**0.45**	**2.12E-08**	0.58	1.14E-07	0.46	3.27E-06
GFPT1	glutamine--fructose-6-phosphate transaminase 1	NM_001244710	**0.66**	**1.35E-11**	0.72	1.65E-09	0.62	2.24E-08
N4BP1	NEDD4 binding protein 1	NM_153029	**0.59**	**1.14E-09**	0.73	1.37E-08	0.70	2.29E-08
LOC100129034	uncharacterized LOC100129034	NR_027406	**-0.63**	**2.20E-08**	-0.71	9.10E-07	-0.54	3.07E-05
IFNE	interferon. epsilon	NM_176891	**1.14**	**2.23E-08**	1.55	4.06E-08	1.02	1.79E-05
ZNF79	zinc finger protein 79	NM_001286696	**0.61**	**2.29E-08**	0.63	3.01E-06	0.73	3.55E-07
HEG1	heart development protein with EGF-like domains 1	NM_020733	**-0.39**	**2.32E-08**	-0.22	2.11E-03	-0.20	4.19E-03
ZNF654	zinc finger protein 654	NM_018293	**0.59**	**2.33E-08**	0.57	7.04E-06	0.57	7.53E-06

SNIP1	Smad nuclear interacting protein 1	ENST00000468040	**0.50**	**2.35E-08**	0.57	6.34E-07	0.71	1.88E-08
TNFRSF21	tumor necrosis factor receptor superfamily. member 21	NM_014452	**-0.44**	**2.39E-08**	-0.63	2.09E-08	-0.24	3.20E-03
KANSL1L	KAT8 regulatory NSL complex subunit 1-like	NM_152519	**0.61**	**3.32E-08**	1.21	1.08E-10	0.89	2.31E-08
AC226119.4	novel transcript	OTTHUMT00000359670	**-0.55**	**2.47E-08**	-0.88	2.93E-09	-0.46	5.27E-05
NPLOC4	nuclear protein localization 4 homolog (S. cerevisiae)	NM_017921	**0.55**	**1.03E-10**	0.73	3.90E-10	0.57	2.55E-08
ZBTB38	zinc finger and BTB domain containing 38	NM_001080412	**-0.48**	**2.57E-08**	-0.23	9.33E-03	-0.22	1.21E-02
C9orf72	chromosome 9 open reading frame 72	NM_001256054	**0.78**	**2.66E-08**	1.03	9.34E-08	1.23	4.87E-09
AFF1	AF4/FMR2 family. member 1	NM_001166693	**0.42**	**2.74E-08**	0.71	1.11E-09	0.41	7.76E-06
VCAM1	vascular cell adhesion molecule 1	NM_001078	**0.90**	**2.74E-08**	1.03	7.99E-07	0.62	4.02E-04
USP14	ubiquitin specific peptidase 14 (tRNA-guanine transglycosylase)	NM_001037334	**0.36**	**2.80E-08**	0.36	4.09E-06	0.25	3.65E-04
HSF2	heat shock transcription factor 2	NM_001135564	**0.70**	**2.85E-08**	0.15	2.19E-01	0.33	8.23E-03
SERTAD1	SERTA domain containing 1	NM_013376	**0.59**	**2.88E-08**	0.66	1.16E-06	1.05	5.56E-10
ZNF189	zinc finger protein 189	NM_001278231	**0.67**	**2.90E-08**	0.14	2.26E-01	0.00	9.68E-01
CDKL5	cyclin-dependent kinase-like 5	NM_001037343	**0.58**	**2.95E-08**	0.88	9.85E-09	0.50	4.02E-05
RP11-274H2.2	novel transcript. antisense to PLOD2	OTTHUMT00000355169	**1.44**	**2.99E-08**	0.43	8.30E-02	1.48	3.79E-06
GIMAP7	GTPase. IMAP family member 7	NM_153236	**-0.68**	**3.10E-08**	-1.04	8.76E-09	-0.80	5.93E-07
NOX5	NADPH oxidase. EF-hand calcium binding domain 5	NM_001184780	**1.08**	**3.10E-08**	0.80	2.14E-04	0.85	1.08E-04
DCUN1D3	DCN1. defective in cullin neddylation 1. domain containing 3	NM_173475	**0.51**	**3.28E-08**	0.46	2.40E-05	0.68	8.36E-08
EXOC8	exocyst complex component 8	NM_175876	**0.53**	**3.30E-08**	0.41	1.33E-04	0.54	4.33E-06
TMEM39A	transmembrane protein 39A	NM_018266	**0.58**	**9.47E-11**	0.66	4.26E-09	0.59	2.70E-08
PLD1	phospholipase D1. phosphatidylcholine-specific	NM_001130081	**-0.54**	**3.33E-08**	-0.54	5.90E-06	-0.77	3.61E-08
SERPIND1	serpin peptidase inhibitor. clade D (heparin cofactor). member 1	NM_000185	**-0.61**	**3.37E-08**	-0.12	2.53E-01	0.18	8.64E-02
SSH1	slingshot protein phosphatase 1	NM_001161330	**0.80**	**3.43E-11**	1.25	5.08E-12	0.77	2.78E-08
LINC01252	long intergenic non-protein coding RNA 1252	NR_033890	**0.66**	**3.67E-08**	0.86	1.35E-07	0.96	1.95E-08
HSPA8	heat shock 70kDa protein 8	NM_006597	**0.34**	**3.76E-08**	0.36	3.89E-06	0.43	2.97E-07
NFKB1	nuclear factor of kappa light polypeptide gene enhancer in B-cells 1	NM_001165412	**0.69**	**8.57E-10**	0.99	6.40E-10	0.79	3.02E-08
NEDD4L	neural precursor cell expressed. developmentally down-regulated 4-like. E3 ubiquitin protein ligase	NM_001144964	**0.46**	**3.81E-08**	0.78	2.31E-09	0.42	2.14E-05
ZFP36L2	ZFP36 ring finger protein-like 2	NM_006887	**-0.51**	**3.86E-08**	-0.62	4.00E-07	-0.53	3.66E-06
CRELD2	cysteine-rich with EGF-like domains 2	NM_001135101	**0.52**	**3.87E-08**	0.59	1.00E-06	0.37	3.40E-04
DAP3	death associated protein 3	ENST00000343043	**0.51**	**3.94E-08**	0.60	6.09E-07	0.57	1.53E-06
NRBF2	nuclear receptor binding factor 2	NM_001282405	**0.51**	**5.35E-08**	0.75	2.85E-08	0.74	3.56E-08
RP11-153M3.1	60 kDa heat shock protein pseudogene	OTTHUMT00000409621	**0.86**	**6.49E-10**	1.19	1.09E-09	0.97	3.60E-08
HEY1	hes-related family bHLH transcription factor with YRPW motif 1	NM_001040708	**0.73**	**4.23E-08**	0.88	5.64E-07	0.90	3.73E-07

MON1B	MON1 secretory trafficking family member B	NM_014940	**0.47**	**4.32E-08**	0.25	5.09E-03	0.42	3.75E-05
SRXN1	sulfiredoxin 1	NM_080725	**0.85**	**1.49E-11**	0.75	5.85E-08	0.77	3.73E-08
NFIB	nuclear factor I/B	NM_001190737	**-0.42**	**9.82E-09**	-0.55	3.14E-08	-0.55	3.90E-08
GLA	galactosidase. alpha	NM_000169	**0.58**	**4.58E-08**	0.36	1.38E-03	0.06	5.60E-01
TCP1	t-complex 1	NM_001008897	**0.45**	**4.68E-08**	0.56	2.67E-07	0.61	7.84E-08
PRR3	proline rich 3	NM_001077497	**0.63**	**4.69E-08**	0.81	2.06E-07	0.73	1.02E-06
PRR3	proline rich 3	NM_001077497	**0.63**	**4.69E-08**	0.81	2.06E-07	0.73	1.02E-06
PRR3	proline rich 3	NM_001077497	**0.63**	**4.69E-08**	0.81	2.06E-07	0.73	1.02E-06
PRR3	proline rich 3	NM_001077497	**0.63**	**4.69E-08**	0.81	2.06E-07	0.73	1.02E-06
GSR	glutathione reductase	NM_000637	**0.43**	**4.83E-08**	0.14	6.60E-02	-0.06	3.87E-01
EFCAB7	EF-hand calcium binding domain 7	NM_032437	**0.84**	**1.29E-08**	1.15	2.06E-08	1.11	3.90E-08
THBD	thrombomodulin	ENST00000377103	**-0.62**	**5.01E-08**	-0.32	6.69E-03	0.45	3.67E-04
MIR1202	microRNA 1202	NR_031606	**0.54**	**5.15E-08**	0.32	2.10E-03	0.31	3.24E-03
IFI27	interferon. alpha-inducible protein 27	NM_001130080	**0.51**	**5.27E-08**	0.49	1.49E-05	0.32	1.43E-03
ABHD17B	abhydrolase domain containing 17B	NM_001025780	**0.57**	**5.28E-08**	0.26	1.32E-02	0.40	5.78E-04
GULP1	GULP. engulfment adaptor PTB domain containing 1	NM_001252668	**0.62**	**9.21E-09**	1.03	5.61E-10	0.80	3.99E-08
RP11-264E18.1	putative novel transcript	OTTHUMT00000051004	**0.69**	**5.32E-08**	0.45	1.18E-03	0.30	2.01E-02
TBK1	TANK-binding kinase 1	NM_013254	**0.48**	**5.34E-08**	0.57	8.00E-07	0.54	1.75E-06
KDM6B	lysine (K)-specific demethylase 6B	NM_001080424	**0.82**	**2.42E-11**	0.95	8.23E-10	0.76	4.12E-08
TNFSF18	tumor necrosis factor (ligand) superfamily. member 18	NM_005092	**-1.03**	**2.38E-12**	-0.83	5.96E-08	-0.84	4.37E-08
SLC35B1	solute carrier family 35. member B1	ENST00000240333	**0.49**	**5.43E-08**	0.37	2.84E-04	0.37	2.59E-04
F3	coagulation factor III (thromboplastin. tissue factor)	NM_001178096	**1.01**	**2.35E-11**	0.92	4.49E-08	0.92	4.56E-08
ZW10	zw10 kinetochore protein	NM_004724	**0.51**	**5.64E-08**	0.31	2.14E-03	0.27	6.36E-03
HDX	highly divergent homeobox	NM_001177479	**0.70**	**5.67E-08**	0.70	1.01E-05	0.68	1.45E-05
CBLB	Cbl proto-oncogene B. E3 ubiquitin protein ligase	NM_170662	**0.49**	**5.67E-08**	0.68	7.47E-08	0.07	4.08E-01
RP1-66C13.3	lectin. galactoside-binding. soluble. 9 (LGALS9) pseudogene	OTTHUMT00000445281	**0.88**	**5.80E-08**	0.18	2.57E-01	-0.04	8.14E-01
ATF2	activating transcription factor 2	NM_001256090	**0.48**	**5.88E-08**	0.50	4.85E-06	0.44	3.00E-05
LOC101928100	uncharacterized LOC101928100	NR_120430	**1.07**	**5.90E-08**	1.05	1.16E-05	0.64	2.41E-03
TMEM41B	transmembrane protein 41B	NM_001165030	**0.53**	**5.91E-08**	0.72	1.20E-07	0.64	6.45E-07
DOLPP1	dolichyldiphosphatase 1	NM_001135917	**0.57**	**5.96E-08**	0.38	9.56E-04	0.35	1.97E-03
HOXB3	homeobox B3	NM_002146	**-0.53**	**5.96E-08**	-0.51	1.87E-05	-0.42	1.88E-04
RANBP9	RAN binding protein 9	NM_005493	**0.45**	**5.97E-08**	0.50	2.03E-06	0.46	7.38E-06
CNKSR3	CNKSR family member 3	NM_173515	**0.86**	**6.32E-10**	0.96	3.24E-08	0.94	4.66E-08
SOX4	SRY (sex determining region Y)-box 4	NM_003107	**-0.38**	**6.04E-08**	-0.52	1.21E-07	-0.52	1.09E-07
GBP3	guanylate binding protein 3	NM_018284	**0.63**	**2.12E-09**	0.72	6.85E-08	0.74	4.94E-08
TNFSF4	tumor necrosis factor (ligand) superfamily. member 4	NM_003326	**-0.58**	**6.30E-08**	-0.57	1.26E-05	-0.20	6.63E-02
ZNF547	zinc finger protein 547	NM_173631	**0.72**	**6.39E-08**	0.64	4.26E-05	0.68	2.28E-05
PHF1	PHD finger protein 1	NM_002636	**0.48**	**6.44E-08**	0.64	1.72E-07	0.45	2.09E-05
ZNF841	zinc finger protein 841	NM_001136499	**0.53**	**6.66E-08**	0.56	4.88E-06	0.52	1.27E-05

SCD5	stearoyl-CoA desaturase 5	NM_001037582	**-0.40**	**6.73E-08**	-0.74	6.43E-10	-0.46	1.53E-06
CBLL1	Cbl proto-oncogene-like 1. E3 ubiquitin protein ligase	NM_001284291	**0.42**	**6.78E-08**	0.63	2.27E-08	0.54	3.10E-07
PITPNB	phosphatidylinositol transfer protein. beta	NM_001284277	**0.48**	**6.89E-08**	0.67	7.22E-08	0.57	9.36E-07
NBPF25P	neuroblastoma breakpoint family. member 25. pseudogene	NR_104217	**0.80**	**7.01E-08**	1.24	1.62E-08	1.09	1.31E-07
NCOA3	nuclear receptor coactivator 3	NM_001174087	**0.40**	**7.07E-08**	0.49	7.25E-07	0.55	1.43E-07
NA	NA	---	**-0.58**	**7.09E-08**	-0.43	3.75E-04	-0.74	3.35E-07
NUFIP2	nuclear fragile X mental retardation protein interacting protein 2	NM_020772	**0.42**	**7.12E-08**	0.42	9.42E-06	0.56	1.74E-07
NEDD9	neural precursor cell expressed. developmentally down-regulated 9	ENST00000379433	**0.49**	**7.13E-08**	0.44	4.65E-05	-0.04	6.63E-01
NEDD9	neural precursor cell expressed. developmentally down-regulated 9	ENST00000379433	**0.49**	**7.13E-08**	0.44	4.65E-05	-0.04	6.63E-01
GOLGA4	golgin A4	NM_001172713	**0.45**	**7.19E-08**	0.32	5.19E-04	0.22	1.02E-02
RASSF3	Ras association (RalGDS/AF-6) domain family member 3	NM_178169	**-0.65**	**7.22E-08**	-0.55	8.04E-05	-0.31	1.30E-02
CTH	cystathionine gamma-lyase	ENST00000346806	**1.01**	**7.56E-09**	1.52	2.68E-09	1.27	5.98E-08
SDPR	serum deprivation response	NM_004657	**-0.61**	**6.68E-09**	-1.02	3.37E-10	-0.75	6.42E-08
TXNL4B	thioredoxin-like 4B	NM_001142317	**0.47**	**7.63E-08**	0.76	6.87E-09	0.52	2.74E-06
ABCB1	ATP-binding cassette. sub-family B (MDR/TAP). member 1	NM_000927	**0.45**	**4.40E-08**	0.69	1.23E-08	0.62	7.20E-08
TXNIP	thioredoxin interacting protein	NM_006472	**-0.78**	**7.33E-11**	-0.96	8.01E-10	-0.73	7.72E-08

NA; not applicable. FC; fold change n= 6 for 12 h. *n*= 3 for 18 h and 24 h.

Table S2: Altered gene expression due to NOX5-β expression at 18 h (bold, highlighted in yellow).

GeneName	GeneDescription	TranscriptID	N12h *vs* G12h		**N18h *vs* G18h**		N24h *vs* G24h	
			logFC	*p-value*	**logFC**	***p-value***	logFC	*p-value*
FAM129A	family with sequence similarity 129. member A	NM_052966	1.45	1.20E-17	**2.44**	**3.27E-19**	2.96	6.24E-21
DNAJA4	DnaJ (Hsp40) homolog. subfamily A. member 4	NM_001130182	2.19	9.83E-20	**2.94**	**2.90E-19**	3.01	1.68E-19
PMAIP1	phorbol-12-myristate-13-acetate-induced protein 1	NM_021127	0.93	1.84E-12	**2.41**	**1.32E-17**	2.69	1.43E-18
E2F7	E2F transcription factor 7	NM_203394	0.71	8.35E-11	**2.20**	**2.21E-17**	2.48	1.84E-18
DNAJB1	DnaJ (Hsp40) homolog. subfamily B. member 1	NM_006145	1.95	2.72E-20	**2.30**	**1.04E-18**	2.13	5.07E-18
PTGS2	prostaglandin-endoperoxide synthase 2 (prostaglandin G/H synthase and cyclooxygenase)	NM_000963	3.80	3.74E-24	**3.89**	**2.89E-21**	2.68	5.83E-18
RND1	Rho family GTPase 1	ENST00000548445	3.09	2.20E-20	**3.42**	**3.17E-18**	3.25	8.99E-18
CHRNA5	cholinergic receptor. nicotinic. alpha 5 (neuronal)	NM_000745	1.30	6.71E-17	**1.78**	**1.31E-16**	1.99	1.44E-17
IL7R	interleukin 7 receptor	NM_002185	2.17	1.43E-19	**2.62**	**3.61E-18**	2.36	3.12E-17
ACTRT3	actin-related protein T3	NM_032487	1.55	1.52E-14	**2.48**	**1.35E-15**	2.94	4.50E-17
CCRN4L	CCR4 carbon catabolite repression 4-like (S. cerevisiae)	NM_012118	1.92	4.22E-19	**2.05**	**1.30E-16**	2.13	6.21E-17
SAT1	spermidine/spermine N1-acetyltransferase 1	NM_002970	0.82	2.04E-13	**1.76**	**4.50E-17**	1.73	6.60E-17
SLC7A5	solute carrier family 7 (amino acid transporter light chain. L system). member 5	NM_003486	0.69	1.39E-08	**2.39**	**7.59E-16**	2.69	7.18E-17
HERPUD1	homocysteine-inducible. endoplasmic reticulum stress-inducible. ubiquitin-like domain member 1	NM_001010989	1.45	1.45E-18	**1.47**	**1.10E-15**	0.36	3.46E-05
GPR89A	G protein-coupled receptor 89A	XM_006711492	1.10	4.66E-16	**1.44**	**2.24E-15**	1.69	8.05E-17
NFKBIZ	nuclear factor of kappa light polypeptide gene enhancer in B-cells inhibitor. zeta	NM_001005474	0.84	2.64E-10	**2.32**	**7.66E-16**	2.60	8.20E-17
GPR89A	G protein-coupled receptor 89A	NM_001097612	1.05	1.23E-15	**1.44**	**2.19E-15**	1.70	8.33E-17
DUSP1	dual specificity phosphatase 1	NM_004417	0.76	2.57E-10	**1.84**	**1.11E-14**	2.31	1.20E-16
HSPA4L	heat shock 70kDa protein 4-like	NM_014278	1.22	9.72E-16	**1.52**	**1.25E-14**	1.90	1.45E-16
STXBP5-AS1	STXBP5 antisense RNA 1	NR_034115	0.56	2.46E-08	**1.46**	**3.28E-13**	2.12	2.15E-16
DNAJB9	DnaJ (Hsp40) homolog. subfamily B. member 9	NM_012328	1.81	5.93E-18	**1.19**	**2.02E-11**	0.53	1.00E-05
CHAC1	ChaC. cation transport regulator homolog 1 (E. coli)	ENST00000446533	1.01	1.68E-09	**3.74**	**1.72E-17**	3.28	2.49E-16
GADD45B	growth arrest and DNA-damage-inducible. beta	NM_015675	0.88	6.24E-13	**1.53**	**1.25E-14**	1.86	2.66E-16
IER5	immediate early response 5	NM_016545	1.01	1.06E-12	**1.85**	**6.09E-15**	2.16	2.98E-16
MXD1	MAX dimerization protein 1	NM_001202513	1.46	2.88E-17	**2.08**	**2.44E-17**	1.81	3.86E-16
ULBP1	UL16 binding protein 1	NM_025218	0.96	8.52E-10	**2.12**	**2.11E-13**	2.91	3.91E-16
RP11-274H2.3	novel transcript	OTTHUMT00000355183	2.01	1.27E-18	**2.29**	**9.69E-17**	2.14	3.92E-16
JMJD1C	jumonji domain containing 1C	NM_001282948	0.61	3.49E-11	**1.44**	**1.99E-15**	1.56	4.05E-16
BAG3	BCL2-associated athanogene 3	NM_004281	1.93	3.06E-19	**2.00**	**1.62E-16**	1.91	4.28E-16
KITLG	KIT ligand	NM_000899	1.52	7.96E-17	**2.12**	**1.05E-16**	1.97	4.77E-16
BMP2	bone morphogenetic protein 2	NM_001200	1.60	1.08E-16	**2.16**	**2.80E-16**	2.04	9.00E-16

ERN1	endoplasmic reticulum to nucleus signaling 1	NM_001433	1.32	1.37E-14	2.24	4.02E-16	2.12	1.16E-15
SESN2	sestrin 2	NM_031459	1.70	3.90E-15	2.81	1.70E-16	2.53	1.37E-15
SIX4	SIX homeobox 4	NM_017420	0.86	1.31E-11	1.55	1.40E-13	1.95	1.39E-15
GDF15	growth differentiation factor 15	NM_004864	0.67	3.73E-11	1.54	2.78E-15	1.58	1.80E-15
CAP2	CAP. adenylate cyclase-associated protein. 2 (yeast)	NM_006366	0.85	5.80E-15	1.09	4.04E-14	1.27	1.90E-15
C7orf60	chromosome 7 open reading frame 60	NM_152556	0.99	4.36E-12	1.79	3.96E-14	2.07	2.35E-15
LOC727896	cysteine and histidine-rich domain (CHORD) containing 1 pseudogene	NR_026659	1.33	1.29E-15	1.64	1.89E-14	1.80	3.16E-15
HSPD1	heat shock 60kDa protein 1 (chaperonin)	NM_002156	1.34	9.15E-18	1.36	7.26E-15	1.40	3.76E-15
KIF21A	kinesin family member 21A	NM_001173463	1.55	2.58E-17	1.91	4.47E-16	1.70	4.18E-15
NA	NA	---	1.50	2.08E-15	1.86	2.82E-14	2.04	4.78E-15
USP53	ubiquitin specific peptidase 53	NM_019050	0.93	5.15E-14	1.45	7.98E-15	1.49	4.92E-15
BACH1	BTB and CNC homology 1. basic leucine zipper transcription factor 1	NR_027655	1.12	1.74E-14	1.61	1.19E-14	1.67	5.86E-15
NA	NA	RPTR-U57609-1	-7.43	5.14E-19	-6.37	1.18E-14	-6.56	6.76E-15
NA	NA	RPTR-U43284-1	-7.43	5.14E-19	-6.37	1.18E-14	-6.56	6.76E-15
NA	NA	RPTR-AB076373-2	-7.43	5.14E-19	-6.37	1.18E-14	-6.56	6.76E-15
PPP1R15A	protein phosphatase 1. regulatory subunit 15A	NM_014330	1.16	8.86E-13	1.73	3.11E-13	2.08	8.84E-15
CPEB4	cytoplasmic polyadenylation element binding protein 4	NM_030627	1.42	6.06E-17	1.91	1.56E-16	1.56	9.34E-15
RP11-212D19.4	novel transcript. RBM7-REXO2 readthrough	OTTHUMT00000399017	2.51	1.98E-13	3.50	2.55E-13	4.13	1.03E-14
LOC100128233	uncharacterized LOC100128233	NR_103769	0.94	4.35E-09	2.15	6.61E-13	2.66	1.05E-14
TNFAIP3	tumor necrosis factor. alpha-induced protein 3	NM_001270507	1.20	5.69E-14	1.72	4.34E-14	1.83	1.28E-14
NAA16	N(alpha)-acetyltransferase 16. NatA auxiliary subunit	NM_001110798	0.91	3.86E-13	1.44	3.87E-14	1.51	1.51E-14
MLKL	mixed lineage kinase domain-like	NM_152649	0.71	3.62E-11	1.20	1.16E-12	1.49	1.86E-14
HSPH1	heat shock 105kDa/110kDa protein 1	NM_001286503	1.35	1.53E-17	1.41	6.52E-15	1.34	1.93E-14
MB21D1	Mab-21 domain containing 1	NM_138441	1.02	3.59E-10	2.02	6.35E-13	2.41	2.08E-14
TUBE1	tubulin. epsilon 1	NM_016262	0.57	6.14E-09	1.22	3.75E-12	1.60	2.08E-14
GOT1	glutamic-oxaloacetic transaminase 1. soluble	NM_002079	0.99	7.08E-13	1.75	8.15E-15	1.67	2.11E-14
IL1A	interleukin 1. alpha	NM_000575	1.35	5.27E-13	2.04	1.44E-13	2.19	3.66E-14
HSPA1A	heat shock 70kDa protein 1A	NM_005345	1.46	5.46E-19	1.38	1.97E-15	1.17	5.07E-14
GCLM	glutamate-cysteine ligase. modifier subunit	NM_002061	0.84	2.13E-14	0.77	8.75E-11	0.36	1.56E-05
ERO1LB	ERO1-like beta (S. cerevisiae)	NM_019891	1.29	2.88E-14	1.56	6.36E-13	0.72	4.47E-07
KDM7A	lysine (K)-specific demethylase 7A	NM_030647	1.61	1.28E-16	2.02	1.52E-15	1.66	6.87E-14
TSPYL2	TSPY-like 2	ENST00000556808	1.21	3.27E-15	1.21	2.84E-12	1.46	7.97E-14
TMEM217	transmembrane protein 217	NM_001162900	0.87	1.93E-10	1.67	5.53E-13	1.85	7.99E-14
CCDC117	coiled-coil domain containing 117	NM_173510	0.80	1.09E-11	1.24	2.15E-12	1.47	8.43E-14
DUSP16	dual specificity phosphatase 16	NM_030640	0.68	1.86E-11	1.08	2.11E-12	1.28	9.15E-14

FGF2	fibroblast growth factor 2 (basic)	NM_002006	0.62	2.31E-11	**1.01**	**1.68E-12**	1.16	1.16E-13
RAB23	RAB23. member RAS oncogene family	NM_001278666	0.96	7.49E-13	**1.51**	**8.40E-14**	1.48	1.24E-13
FKBP4	FK506 binding protein 4. 59kDa	NM_002014	0.98	1.37E-14	**1.02**	**4.80E-12**	1.23	1.27E-13
HSPA1A	heat shock 70kDa protein 1A	NM_005345	1.45	1.91E-18	**1.37**	**6.50E-15**	1.17	1.43E-13
IFI44	interferon-induced protein 44	NM_006417	1.85	1.07E-13	**1.35**	**1.93E-08**	0.64	4.14E-04
HSPA1B	heat shock 70kDa protein 1B	NM_005346	1.43	3.60E-18	**1.39**	**6.61E-15**	1.18	1.58E-13
IFIH1	interferon induced with helicase C domain 1	NM_022168	1.98	1.11E-13	**1.43**	**2.25E-08**	0.97	7.57E-06
RP11-762H8.4	novel transcript . sense intronic to WDR61	OTTHUMT00000471376	1.04	8.69E-10	**1.31**	**6.51E-09**	2.31	1.86E-13
PPID	peptidylprolyl isomerase D	NM_005038	0.71	1.59E-10	**0.99**	**1.86E-10**	1.43	1.97E-13
AHSA1	AHA1. activator of heat shock 90kDa protein ATPase homolog 1 (yeast)	NM_012111	1.05	1.15E-14	**1.13**	**2.45E-12**	1.28	2.15E-13
CEBPG	CCAAT/enhancer binding protein (C/EBP). gamma	NM_001252296	0.59	3.90E-09	**1.59**	**2.23E-14**	1.41	2.28E-13
NXT2	nuclear transport factor 2-like export factor 2	NM_001242617	0.63	3.97E-08	**1.11**	**9.77E-10**	1.73	2.38E-13
KBTBD8	kelch repeat and BTB (POZ) domain containing 8	NM_032505	0.97	5.52E-11	**1.49**	**1.17E-11**	1.81	2.85E-13
IRAK2	interleukin-1 receptor-associated kinase 2	NM_001570	0.90	1.63E-10	**1.65**	**1.28E-12**	1.78	2.94E-13
XBP1	X-box binding protein 1	NM_001079539	0.49	2.42E-10	**1.05**	**9.60E-14**	0.98	3.37E-13
ABHD3	abhydrolase domain containing 3	NM_138340	1.30	1.39E-12	**1.58**	**2.55E-11**	1.97	3.65E-13
GBE1	glucan (1.4-alpha-). branching enzyme 1	ENST00000429644	0.52	3.31E-09	**1.03**	**7.71E-12**	1.20	3.85E-13
HSPA1B	heat shock 70kDa protein 1B	NM_005346	1.25	1.30E-17	**1.23**	**1.68E-14**	1.05	3.92E-13
HSPA1A	heat shock 70kDa protein 1A	NM_005345	1.33	3.05E-18	**1.26**	**1.05E-14**	1.04	4.03E-13
TXNL1	thioredoxin-like 1	ENST00000587807	0.70	3.30E-13	**0.60**	**4.11E-09**	0.45	4.37E-07
TRAF6	TNF receptor-associated factor 6. E3 ubiquitin protein ligase	NM_004620	0.60	4.94E-10	**1.07**	**6.14E-12**	1.23	4.08E-13
LRRC8B	leucine rich repeat containing 8 family. member B	NM_001134476	1.01	3.23E-16	**1.03**	**1.96E-13**	1.00	4.13E-13
HECW2	HECT. C2 and WW domain containing E3 ubiquitin protein ligase 2	NM_020760	0.71	4.08E-11	**1.26**	**6.56E-13**	1.28	4.57E-13
RGS2	regulator of G-protein signaling 2	NM_002923	0.64	4.20E-09	**1.23**	**1.67E-11**	1.48	4.72E-13
HSPA1A	heat shock 70kDa protein 1A	NM_005345	1.33	2.81E-18	**1.24**	**1.08E-14**	1.02	4.79E-13
HSPA1A	heat shock 70kDa protein 1A	NM_005345	1.33	2.81E-18	**1.24**	**1.08E-14**	1.02	4.79E-13
HSPA1A	heat shock 70kDa protein 1A	NM_005345	1.33	2.81E-18	**1.24**	**1.08E-14**	1.02	4.79E-13
NOP58	NOP58 ribonucleoprotein	ENST00000264279	0.49	1.73E-10	**0.72**	**9.14E-11**	0.95	5.39E-13
PRKACB	protein kinase. cAMP-dependent. catalytic. beta	NM_001242857	0.58	3.88E-10	**0.78**	**1.10E-09**	1.17	5.64E-13
FST	follistatin	NM_006350	1.84	5.50E-13	**1.60**	**4.20E-09**	1.34	8.17E-08
CXCL8	chemokine (C-X-C motif) ligand 8	NM_000584	2.28	5.88E-17	**2.23**	**9.11E-14**	2.03	5.65E-13
NAV3	neuron navigator 3	NM_001024383	0.57	1.62E-10	**1.01**	**2.69E-12**	1.09	5.75E-13
HBEGF	heparin-binding EGF-like growth factor	NM_001945	0.49	1.10E-08	**1.02**	**1.35E-11**	1.20	6.46E-13
HSPA1B	heat shock 70kDa protein 1B	NM_005346	1.47	9.72E-18	**1.36**	**4.56E-14**	1.18	7.30E-13
GXYLT2	glucoside xylosyltransferase 2	NM_001080393	1.08	1.76E-08	**1.98**	**1.95E-10**	2.66	7.69E-13

ARG2	arginase 2	NM_001172	0.94	2.30E-13	**1.48**	**3.27E-14**	1.25	8.53E-13
DDX58	DEAD (Asp-Glu-Ala-Asp) box polypeptide 58	NM_014314	1.18	7.85E-13	**1.01**	**7.11E-09**	0.81	2.58E-07
NCOA7	nuclear receptor coactivator 7	NM_001199619	1.19	8.61E-13	**1.27**	**1.49E-10**	0.79	4.38E-07
OTULIN	OTU deubiquitinase with linear linkage specificity	NM_138348	0.74	1.31E-10	**1.17**	**1.74E-11**	1.38	8.75E-13
DEDD2	death effector domain containing 2	NM_001270614	1.55	5.06E-13	**2.16**	**6.66E-13**	2.12	9.04E-13
STX11	syntaxin 11	NM_003764	0.72	3.44E-10	**1.22**	**1.30E-11**	1.40	9.44E-13
CYLD	cylindromatosis (turban tumor syndrome)	NM_001042355	0.60	2.94E-10	**1.03**	**1.01E-11**	1.16	9.51E-13
STK38L	serine/threonine kinase 38 like	NM_015000	1.44	4.06E-17	**1.67**	**2.05E-15**	1.22	9.69E-13
ISG15	ISG15 ubiquitin-like modifier	NM_005101	1.45	1.83E-12	**1.37**	**2.97E-09**	0.92	1.74E-06
HSPA1B	heat shock 70kDa protein 1B	NM_005346	1.33	1.55E-17	**1.29**	**3.04E-14**	1.07	1.06E-12
FAM46A	family with sequence similarity 46. member A	ENST00000369754	1.76	3.05E-15	**1.82**	**1.37E-12**	1.84	1.14E-12
LYSMD3	LysM. putative peptidoglycan-binding. domain containing 3	NM_198273	0.82	2.09E-12	**0.72**	**1.19E-08**	0.38	1.05E-04
DNAJB6	DnaJ (Hsp40) homolog. subfamily B. member 6	NM_005494	0.77	1.56E-11	**1.24**	**1.26E-12**	1.24	1.22E-12
TRIM26	tripartite motif containing 26	NM_001242783	0.97	6.11E-14	**1.04**	**1.11E-11**	1.17	1.41E-12
XAF1	XIAP associated factor 1	NM_017523	1.97	3.63E-12	**1.96**	**2.10E-09**	1.56	1.01E-07
IFITM1	interferon induced transmembrane protein 1	NM_003641	1.49	3.99E-12	**1.22**	**5.93E-08**	0.99	1.61E-06
IKZF5	IKAROS family zinc finger 5 (Pegasus)	NM_001271840	0.85	1.63E-13	**0.93**	**1.96E-11**	1.07	1.52E-12
GPBP1	GC-rich promoter binding protein 1	NM_001203246	0.53	1.73E-09	**0.94**	**2.41E-11**	1.08	1.79E-12
CLIC2	chloride intracellular channel 2	NM_001289	1.10	1.05E-10	**1.70**	**2.13E-11**	1.93	1.82E-12
TRIM26	tripartite motif containing 26	NM_001242783	1.00	9.55E-14	**1.09**	**1.37E-11**	1.21	1.90E-12
TRIM26	tripartite motif containing 26	NM_001242783	0.96	9.89E-14	**1.04**	**1.67E-11**	1.16	2.20E-12
CACYBP	calcyclin binding protein	NM_001007214	0.89	5.80E-13	**1.14**	**4.23E-12**	1.18	2.27E-12
UBR2	ubiquitin protein ligase E3 component n-recognin 2	NM_015255	0.47	3.60E-10	**0.67**	**3.37E-10**	0.87	2.29E-12
TRIB3	tribbles pseudokinase 3	uc002wdn.3	0.83	1.68E-08	**1.68**	**3.13E-11**	1.92	2.60E-12
ELL2	elongation factor. RNA polymerase II. 2	NM_012081	0.83	3.86E-14	**1.27**	**7.84E-15**	0.94	2.71E-12
PDE4DIP	phosphodiesterase 4D interacting protein	NM_001198832	0.85	1.25E-10	**1.48**	**2.57E-12**	1.47	2.87E-12
CYB5R1	cytochrome b5 reductase 1	ENST00000482572	0.76	1.07E-11	**0.69**	**2.79E-08**	0.62	1.48E-07
TRIM26	tripartite motif containing 26	NM_001242783	0.99	2.46E-13	**1.13**	**1.42E-11**	1.23	2.88E-12
DNAJA1	DnaJ (Hsp40) homolog. subfamily A. member 1	NM_001539	0.82	2.23E-13	**0.98**	**5.07E-12**	1.01	2.92E-12
JMJD6	jumonji domain containing 6	NM_001081461	0.59	2.88E-09	**0.78**	**8.85E-09**	1.21	3.00E-12
RAB39A	RAB39A. member RAS oncogene family	NM_017516	1.21	3.78E-09	**2.32**	**1.56E-11**	2.53	3.03E-12
MICB	MHC class I polypeptide-related sequence B	NM_001289160	0.69	1.81E-11	**1.16**	**6.80E-13**	1.06	3.71E-12
MERTK	MER proto-oncogene. tyrosine kinase	NM_006343	0.49	5.20E-09	**0.97**	**1.09E-11**	1.02	4.01E-12
TRIM26	tripartite motif containing 26	NM_001242783	0.97	1.89E-13	**1.05**	**3.46E-11**	1.17	4.09E-12
TRIM26	tripartite motif containing 26	NM_001242783	0.97	1.89E-13	**1.05**	**3.46E-11**	1.17	4.09E-12
ABL2	ABL proto-oncogene 2. non-receptor tyrosine kinase	NM_001136000	0.66	2.19E-11	**1.06**	**1.85E-12**	1.01	4.58E-12

IFI6	interferon. alpha-inducible protein 6	NM_002038	1.74	1.63E-11	1.68	1.46E-08	1.39	3.13E-07
MICB	MHC class I polypeptide-related sequence B	NM_001289161	0.71	2.62E-11	1.13	2.94E-12	1.11	4.58E-12
ATP2C1	ATPase. Ca++ transporting. type 2C. member 1	NM_001001485	0.51	1.09E-09	0.76	4.95E-10	0.98	4.60E-12
NA	NA	RPTR-AF292560-1	-2.57	1.70E-14	-2.44	3.61E-11	-2.69	5.69E-12
SEC24A	SEC24 family member A	NM_021982	0.71	1.89E-11	0.66	3.19E-08	0.36	1.27E-04
MICB	MHC class I polypeptide-related sequence B	NM_005931	0.58	2.18E-10	1.01	4.82E-12	1.01	5.75E-12
MICB	MHC class I polypeptide-related sequence B	NM_005931	0.58	2.18E-10	1.01	4.82E-12	1.01	5.75E-12
NRIP3	nuclear receptor interacting protein 3	NM_020645	0.90	8.82E-11	1.32	4.46E-11	1.47	5.96E-12
MICB	MHC class I polypeptide-related sequence B	NM_005931	0.66	1.03E-10	1.06	9.07E-12	1.08	6.13E-12
NA	NA	RPTR-AY189981-1	-6.80	3.16E-16	-5.56	1.37E-11	-5.79	6.41E-12
C3orf38	chromosome 3 open reading frame 38	NM_173824	0.60	5.36E-10	1.00	2.46E-11	1.07	7.44E-12
PLOD2	procollagen-lysine. 2-oxoglutarate 5-dioxygenase 2	NM_000935	0.74	5.44E-13	0.95	2.84E-12	0.91	7.45E-12
DNAJB11	DnaJ (Hsp40) homolog. subfamily B. member 11	ENST00000439351	0.71	3.38E-11	0.72	1.36E-08	0.36	1.90E-04
STXBP5-AS1	STXBP5 antisense RNA 1	ENST00000433308	0.71	6.13E-08	2.01	2.22E-13	1.66	8.67E-12
RYBP	RING1 and YY1 binding protein	NM_012234	0.88	2.39E-11	1.27	1.61E-11	1.31	9.43E-12
STC1	stanniocalcin 1	NM_003155	-0.56	6.17E-10	-0.94	2.78E-11	-1.00	9.56E-12
ANXA1	annexin A1	NM_000700	0.43	5.67E-09	0.89	6.71E-12	0.87	9.62E-12
LOC344887	NmrA-like family domain containing 1 pseudogene	NR_033752	0.83	9.97E-13	1.07	6.33E-12	1.04	1.02E-11
TRIM26	tripartite motif containing 26	ENST00000436219	0.99	1.30E-13	1.07	2.13E-11	1.11	1.07E-11
ZCCHC6	zinc finger. CCHC domain containing 6	NM_001185059	0.57	3.17E-10	0.91	3.44E-11	0.97	1.14E-11
SNAI1	snail family zinc finger 1	NM_005985	0.79	3.35E-09	1.84	3.73E-13	1.54	1.20E-11
RASSF8	Ras association (RalGDS/AF-6) domain family (N-terminal) member 8	NM_001164746	0.56	1.69E-08	0.90	1.98E-09	1.19	1.22E-11
UHRF1BP1L	UHRF1 binding protein 1-like	NM_001006947	1.07	4.84E-13	1.37	3.60E-12	1.28	1.26E-11
ZFAND2A	zinc finger. AN1-type domain 2A	NM_182491	1.99	2.08E-19	1.84	1.12E-15	1.13	1.36E-11
FERMT3	fermitin family member 3	NM_031471	-0.65	6.25E-11	-0.66	2.25E-08	-0.37	9.26E-05
ENTPD7	ectonucleoside triphosphate diphosphohydrolase 7	NM_020354	0.63	6.77E-11	0.60	6.02E-08	0.51	8.23E-07
HSPA6	heat shock 70kDa protein 6 (HSP70B)	NM_002155	1.95	1.18E-13	2.17	1.16E-11	2.15	1.38E-11
TRIM26	tripartite motif containing 26	ENST00000415923	0.99	3.56E-13	1.11	2.71E-11	1.15	1.38E-11
SLFN5	schlafen family member 5	NM_144975	0.76	3.70E-11	1.27	1.48E-12	1.12	1.58E-11
MICB	MHC class I polypeptide-related sequence B	NM_005931	0.62	2.78E-10	1.05	9.28E-12	1.02	1.62E-11
MED13	mediator complex subunit 13	NM_005121	0.51	8.74E-11	0.86	3.30E-12	0.78	1.82E-11
PDE4DIP	phosphodiesterase 4D interacting protein	NM_001002811	0.63	5.31E-10	0.91	3.77E-10	1.07	1.92E-11
HSPA9	heat shock 70kDa protein 9 (mortalin)	NM_004134	0.69	4.25E-12	0.88	3.08E-11	0.90	1.94E-11
SNAP23	synaptosomal-associated protein. 23kDa	NM_003825	0.41	5.26E-09	0.71	1.90E-10	0.80	2.06E-11

SLC5A3	solute carrier family 5 (sodium/myo-inositol cotransporter). member 3	NM_006933	0.60	1.67E-08	**0.93**	**3.07E-09**	1.23	2.16E-11
PDE4DIP	phosphodiesterase 4D interacting protein	NM_001198832	0.99	6.83E-10	**1.73**	**1.42E-11**	1.68	2.38E-11
UBR1	ubiquitin protein ligase E3 component n-recognin 1	NM_174916	0.57	2.62E-11	**0.70**	**3.09E-10**	0.80	2.39E-11
GCNT2	glucosaminyl (N-acetyl) transferase 2. I-branching enzyme (I blood group)	NM_001491	0.93	9.16E-11	**0.98**	**1.79E-08**	0.77	7.03E-07
SLC3A2	solute carrier family 3 (amino acid transporter heavy chain). member 2	NM_001012662	0.52	5.39E-08	**1.18**	**1.39E-11**	1.14	2.52E-11
ALAS1	aminolevulinate. delta-. synthase 1	NM_000688	0.57	6.15E-11	**0.55**	**4.82E-08**	0.85	2.56E-11
HSP90AA1	heat shock protein 90kDa alpha (cytosolic). class A member 1	NM_001017963	0.89	4.90E-14	**0.91**	**2.19E-11**	0.90	2.93E-11
RNU6-71P	RNA. U6 small nuclear 71. pseudogene	NR_046940	2.11	7.23E-11	**3.37**	**7.85E-12**	3.14	2.98E-11
AZIN1	antizyme inhibitor 1	NM_015878	0.72	3.87E-11	**0.93**	**1.99E-10**	1.01	3.99E-11
DDIT3	DNA-damage-inducible transcript 3	NM_001195053	1.92	1.77E-14	**2.10**	**2.74E-12**	1.82	4.06E-11
PIGA	phosphatidylinositol glycan anchor biosynthesis. class A	NM_002641	0.80	4.52E-12	**1.12**	**5.41E-12**	1.00	4.27E-11
BRD2	bromodomain containing 2	NM_001113182	0.49	1.16E-10	**0.65**	**4.13E-10**	0.73	4.70E-11
YRDC	yrdC N(6)-threonylcarbamoyltransferase domain containing	NM_024640	0.67	6.52E-12	**0.83**	**8.03E-11**	0.85	4.79E-11
HYOU1	hypoxia up-regulated 1	NM_001130991	0.77	8.18E-14	**0.98**	**6.17E-13**	0.77	4.97E-11
BRD2	bromodomain containing 2	NM_001113182	0.50	1.19E-10	**0.66**	**3.74E-10**	0.73	5.85E-11
BRD2	bromodomain containing 2	NM_001113182	0.52	1.30E-10	**0.67**	**5.27E-10**	0.76	6.14E-11
RNU6-329P	RNA. U6 small nuclear 329. pseudogene	ENST00000459618	1.59	3.51E-09	**2.20**	**5.18E-09**	2.82	6.16E-11
HSPA4	heat shock 70kDa protein 4	NM_002154	0.78	2.06E-13	**0.97**	**2.32E-12**	0.81	6.54E-11
HSPA5	heat shock 70kDa protein 5 (glucose-regulated protein. 78kDa)	NM_005347	0.88	7.41E-16	**1.06**	**2.04E-14**	0.69	6.60E-11
NA	NA	RPTR-AJ510163-3	-6.55	3.02E-15	**-5.20**	**1.84E-10**	-5.48	7.04E-11
MICB	MHC class I polypeptide-related sequence B	ENST00000458032	0.57	2.57E-09	**0.95**	**1.20E-10**	0.98	7.09E-11
PTGES3	prostaglandin E synthase 3 (cytosolic)	NM_001282601	0.54	3.61E-10	**0.72**	**9.65E-10**	0.83	7.31E-11
ULBP3	UL16 binding protein 3	NM_024518	0.87	4.44E-08	**1.31**	**1.55E-08**	1.78	7.47E-11
ME1	malic enzyme 1. NADP(+)-dependent. cytosolic	NM_002395	0.68	7.04E-13	**0.90**	**2.39E-12**	0.75	7.84E-11
RND3	Rho family GTPase 3	NM_001254738	0.55	5.37E-10	**0.84**	**1.55E-10**	0.87	8.40E-11
UBR4	ubiquitin protein ligase E3 component n-recognin 4	NM_020765	0.51	1.64E-10	**0.57**	**1.02E-08**	0.30	1.23E-04
BRD2	bromodomain containing 2	NM_001291986	0.52	7.94E-11	**0.69**	**2.54E-10**	0.73	8.78E-11
NA	NA	NONHSAT098813	1.72	4.11E-14	**1.48**	**4.62E-10**	1.63	8.88E-11
MLLT11	myeloid/lymphoid or mixed-lineage leukemia (trithorax homolog. Drosophila); translocated to. 11	NM_006818	0.57	4.04E-12	**0.69**	**7.50E-11**	0.68	9.47E-11
SORBS1	sorbin and SH3 domain containing 1	NM_001034954	0.93	1.15E-08	**1.67**	**1.57E-10**	1.72	9.56E-11

OTUD7B	OTU deubiquitinase 7B	NM_020205	0.68	1.75E-10	**0.72**	**2.46E-08**	0.99	9.58E-11
DYRK3	dual-specificity tyrosine-(Y)-phosphorylation regulated kinase 3	ENST00000367106	0.53	6.04E-09	**0.92**	**1.92E-10**	0.95	9.77E-11
MAFF	v-maf avian musculoaponeurotic fibrosarcoma oncogene homolog F	NM_001161572	0.91	2.06E-10	**1.00**	**1.45E-08**	0.83	3.02E-07
NA	NA	TCONS_l2_00014930-XLOC_l2_008285	0.80	8.67E-12	**1.05**	**3.47E-11**	0.99	1.03E-10
NA	NA	---	0.94	9.50E-11	**1.19**	**6.98E-10**	1.32	1.05E-10
KCTD12	potassium channel tetramerization domain containing 12	NM_138444	-0.64	2.55E-09	**-1.25**	**7.53E-12**	-1.08	1.10E-10
VEGFC	vascular endothelial growth factor C	NM_005429	0.74	8.06E-11	**0.89**	**1.45E-09**	1.03	1.22E-10
CABLES2	Cdk5 and Abl enzyme substrate 2	NM_031215	0.70	1.24E-09	**1.07**	**2.92E-10**	1.13	1.22E-10
TSC22D2	TSC22 domain family. member 2	ENST00000361875	0.79	1.50E-10	**1.17**	**6.80E-11**	1.13	1.24E-10
GTPBP2	GTP binding protein 2	NM_001286216	0.56	7.47E-08	**0.93**	**5.46E-09**	1.15	1.28E-10
SQSTM1	sequestosome 1	NM_001142298	0.59	2.61E-10	**0.61**	**5.53E-08**	0.48	1.86E-06
CNST	consortin. connexin sorting protein	NM_152609	0.73	1.42E-09	**1.15**	**1.82E-10**	1.17	1.32E-10
TMEM47	transmembrane protein 47	NM_031442	0.47	1.58E-09	**0.80**	**4.99E-11**	0.75	1.45E-10
EIF5	eukaryotic translation initiation factor 5	NM_001969	0.65	3.68E-11	**0.75**	**1.44E-09**	0.85	1.45E-10
SPTY2D1	SPT2. Suppressor of Ty. domain containing 1 (S. cerevisiae)	NM_194285	0.68	3.12E-10	**0.70**	**7.64E-08**	0.63	4.36E-07
SPAG9	sperm associated antigen 9	NM_001130527	0.67	1.00E-10	**1.03**	**1.86E-11**	0.92	1.52E-10
SERPINH1	serpin peptidase inhibitor. clade H (heat shock protein 47). member 1. (collagen binding protein 1)	NM_001207014	0.56	1.55E-11	**0.71**	**1.18E-10**	0.70	1.58E-10
STIP1	stress-induced phosphoprotein 1	NM_001282652	0.70	1.20E-11	**0.69**	**6.79E-09**	0.86	1.66E-10
KDM2A	lysine (K)-specific demethylase 2A	NM_001256405	0.68	7.26E-11	**0.99**	**5.07E-11**	0.92	1.72E-10
PPME1	protein phosphatase methylesterase 1	ENST00000535205	0.79	1.18E-11	**0.95**	**2.14E-10**	0.96	1.83E-10
ATF3	activating transcription factor 3	NM_001030287	2.75	1.94E-12	**3.39**	**2.52E-11**	3.03	1.95E-10
HSPA1L	heat shock 70kDa protein 1-like	NM_005527	0.86	3.68E-10	**1.05**	**4.84E-09**	0.83	2.10E-07
SLC39A14	solute carrier family 39 (zinc transporter). member 14	NM_001128431	1.06	1.21E-12	**1.35**	**8.98E-12**	1.14	2.10E-10
HSPB1	heat shock 27kDa protein 1	NM_001540	0.51	2.21E-10	**0.76**	**8.35E-11**	0.72	2.21E-10
ADAMTS18	ADAM metallopeptidase with thrombospondin type 1 motif. 18	NM_199355	-0.58	1.05E-08	**-1.22**	**9.46E-12**	-1.03	2.27E-10
TMEM57	transmembrane protein 57	NM_018202	0.53	4.32E-10	**0.59**	**2.43E-08**	0.25	1.60E-03
SERPINE2	serpin peptidase inhibitor. clade E (nexin. plasminogen activator inhibitor type 1). member 2	NM_001136528	0.55	4.35E-10	**0.74**	**1.29E-09**	0.51	5.36E-07
EPB41	erythrocyte membrane protein band 4.1	NM_001166005	0.57	4.43E-10	**0.66**	**1.33E-08**	0.59	1.06E-07
TGOLN2	trans-golgi network protein 2	NM_001206840	-0.57	4.80E-10	**-0.62**	**3.85E-08**	-0.38	4.88E-05
HERC4	HECT and RLD domain containing E3 ubiquitin protein ligase 4	NM_001278185	0.45	7.73E-08	**0.64**	**6.49E-08**	0.89	2.29E-10

DNAJC24	DnaJ (Hsp40) homolog. subfamily C. member 24	ENST00000526042	0.57	4.92E-08	**0.98**	**2.06E-09**	1.10	2.63E-10
IGHJ1	immunoglobulin heavy joining 1	ENST00000390565	-1.44	3.45E-09	**-1.76**	**3.67E-08**	-2.34	2.79E-10
PHACTR4	phosphatase and actin regulator 4	NM_001048183	0.57	5.37E-10	**0.66**	**1.64E-08**	0.39	3.78E-05
FAM219A	family with sequence similarity 219. member A	NM_001184940	0.69	5.19E-09	**1.14**	**3.65E-10**	1.14	3.54E-10
CSRNP1	cysteine-serine-rich nuclear protein 1	NM_033027	0.76	5.94E-09	**1.30**	**1.91E-10**	1.25	3.90E-10
SAMD4B	sterile alpha motif domain containing 4B	NM_018028	0.64	1.15E-09	**1.06**	**6.32E-11**	0.95	4.22E-10
SIK1	salt-inducible kinase 1	NM_173354	0.98	1.04E-11	**1.60**	**6.61E-13**	1.12	4.44E-10
CCDC174	coiled-coil domain containing 174	NM_016474	0.74	4.23E-11	**0.94**	**3.30E-10**	0.92	4.67E-10
TYW3	tRNA-yW synthesizing protein 3 homolog (S. cerevisiae)	NM_001162916	0.62	3.52E-08	**0.98**	**4.88E-09**	1.12	4.87E-10
HSPB1	heat shock 27kDa protein 1	NM_001540	0.48	1.82E-10	**0.61**	**1.34E-09**	0.64	5.91E-10
SPEN	spen family transcriptional repressor	NM_015001	0.70	1.27E-10	**1.04**	**5.18E-11**	0.91	6.34E-10
NA	NA	---	0.86	2.48E-08	**1.17**	**4.42E-08**	1.51	6.42E-10
MICA	MHC class I polypeptide-related sequence A	ENST00000415525	0.53	2.47E-09	**0.83**	**3.46E-10**	0.80	7.01E-10
KIAA0513	16q24.1	NM_001286565	1.60	5.92E-13	**1.82**	**3.85E-11**	1.55	7.15E-10
RP11-143M1.4	putative novel transcript	OTTHUMT00000051455	1.34	9.76E-10	**1.78**	**2.99E-09**	1.09	5.88E-06
UBB	ubiquitin B	NM_018955	0.52	9.98E-10	**0.68**	**3.82E-09**	0.56	9.67E-08
FEM1B	fem-1 homolog b (C. elegans)	NM_015322	0.63	2.29E-10	**0.91**	**1.85E-10**	0.84	7.38E-10
TP53BP2	tumor protein p53 binding protein 2	NM_001031685	0.75	1.08E-10	**1.02**	**2.25E-10**	0.95	8.61E-10
KCTD5	potassium channel tetramerization domain containing 5	NM_018992	0.57	7.59E-11	**0.62**	**7.52E-09**	0.70	9.37E-10
RPS6KA3	ribosomal protein S6 kinase. 90kDa. polypeptide 3	NM_004586	0.54	6.10E-10	**0.72**	**1.65E-09**	0.73	1.08E-09
LURAP1L	leucine rich adaptor protein 1-like	NM_203403	0.65	3.81E-08	**0.95**	**2.48E-08**	1.14	1.12E-09
CCDC59	coiled-coil domain containing 59	NM_014167	0.50	2.17E-09	**0.86**	**7.63E-11**	0.74	1.16E-09
CPA4	carboxypeptidase A4	NM_001163446	-0.65	4.33E-09	**-1.23**	**2.31E-11**	-0.99	1.22E-09
MORC4	MORC family CW-type zinc finger 4	NM_001085354	0.66	1.87E-10	**0.75**	**8.07E-09**	0.84	1.24E-09
PSMC4	proteasome (prosome. macropain) 26S subunit. ATPase. 4	NM_006503	0.74	3.40E-12	**0.92**	**3.74E-11**	0.76	1.27E-09
UBB	ubiquitin B	NM_018955	0.60	1.62E-09	**0.75**	**1.45E-08**	0.60	5.17E-07
IPPK	inositol 1.3.4.5.6-pentakisphosphate 2-kinase	NM_022755	0.67	1.97E-10	**0.96**	**1.74E-10**	0.85	1.32E-09
EDA2R	ectodysplasin A2 receptor	NM_001199687	0.69	3.95E-09	**1.18**	**1.26E-10**	1.03	1.42E-09
ENC1	ectodermal-neural cortex 1 (with BTB domain)	NM_001256574	-0.58	1.76E-09	**-0.69**	**3.00E-08**	-0.57	5.60E-07
FXR1	fragile X mental retardation. autosomal homolog 1	NM_001013438	0.50	1.55E-09	**0.69**	**2.19E-09**	0.70	1.43E-09
KIF1B	kinesin family member 1B	NM_015074	0.48	1.89E-09	**0.57**	**3.06E-08**	0.23	2.28E-03
IFRD1	interferon-related developmental regulator 1	NM_001007245	0.64	7.77E-09	**0.96**	**2.49E-09**	0.99	1.50E-09
ERRFI1	ERBB receptor feedback inhibitor 1	ENST00000467067	0.38	8.01E-08	**1.01**	**1.11E-12**	0.68	1.66E-09

DYNC1I1	dynein. cytoplasmic 1. intermediate chain 1	NM_001278421	0.91	2.17E-09	**1.04**	**8.14E-08**	0.81	3.14E-06
C18orf25	chromosome 18 open reading frame 25	NM_001008239	0.84	1.70E-11	**0.99**	**5.25E-10**	0.92	1.86E-09
PHF1	PHD finger protein 1	NM_002636	0.55	2.48E-09	**0.67**	**3.10E-08**	0.54	9.46E-07
ITCH	itchy E3 ubiquitin protein ligase	NM_001257137	0.48	2.52E-09	**0.58**	**3.67E-08**	0.51	3.54E-07
BIRC2	baculoviral IAP repeat containing 2	NM_001166	0.61	1.55E-10	**0.92**	**4.97E-11**	0.75	1.86E-09
NA	NA	---	0.65	1.61E-08	**1.05**	**1.85E-09**	1.04	2.02E-09
LARP4	La ribonucleoprotein domain family. member 4	NM_001170803	0.71	2.20E-10	**0.81**	**9.24E-09**	0.88	2.05E-09
DCP1A	decapping mRNA 1A	NM_001290204	0.51	2.88E-09	**0.70**	**4.12E-09**	0.58	8.91E-08
SOD1	superoxide dismutase 1. soluble	NM_000454	0.50	3.25E-09	**0.70**	**3.69E-09**	0.52	5.04E-07
RP4-791M13.3	novel transcript	OTTHUMT00000384603	0.81	1.55E-08	**1.35**	**8.35E-10**	1.28	2.06E-09
HSPB1P1	heat shock 27kDa protein 1 pseudogene 1	ENST00000423240	0.37	3.96E-09	**0.53**	**3.79E-09**	0.54	2.28E-09
NFE2L2	nuclear factor. erythroid 2-like 2	NM_001145412	0.46	3.35E-09	**0.65**	**3.87E-09**	0.51	1.75E-07
SLC37A1	solute carrier family 37 (glucose-6-phosphate transporter). member 1	NM_018964	0.50	4.10E-08	**0.71**	**3.51E-08**	0.83	2.29E-09
BRPF3	bromodomain and PHD finger containing. 3	NM_015695	0.68	4.66E-09	**0.97**	**3.69E-09**	1.00	2.33E-09
TBC1D4	TBC1 domain family. member 4	NM_001286658	0.72	7.90E-10	**0.95**	**2.90E-09**	0.96	2.34E-09
IPMK	inositol polyphosphate multikinase	NM_152230	0.60	5.30E-08	**1.06**	**1.19E-09**	1.01	2.41E-09
MICA	MHC class I polypeptide-related sequence A	ENST00000400325	0.53	2.16E-08	**0.92**	**7.06E-10**	0.85	2.47E-09
MICA	MHC class I polypeptide-related sequence A	ENST00000400325	0.53	2.16E-08	**0.92**	**7.06E-10**	0.85	2.47E-09
ZNF317	zinc finger protein 317	NM_001190791	0.54	1.28E-08	**0.73**	**3.35E-08**	0.84	2.60E-09
ATG5	autophagy related 5	NM_001286106	0.50	1.70E-08	**0.78**	**3.03E-09**	0.78	2.85E-09
PLAA	phospholipase A2-activating protein	NM_001031689	0.80	1.65E-14	**0.73**	**6.74E-11**	0.59	2.91E-09
VPS37A	vacuolar protein sorting 37 homolog A (S. cerevisiae)	NM_001145152	0.54	4.38E-09	**0.66**	**3.89E-08**	0.49	4.34E-06
ACBD3	acyl-CoA binding domain containing 3	NM_022735	0.75	6.99E-11	**0.88**	**1.93E-09**	0.86	2.97E-09
ELL	elongation factor RNA polymerase II	NM_006532	0.66	5.99E-08	**1.09**	**4.43E-09**	1.11	2.99E-09
SLC7A2	solute carrier family 7 (cationic amino acid transporter. y+ system). member 2	NM_001008539	0.76	4.21E-10	**1.37**	**5.33E-12**	0.96	3.31E-09
HMOX1	heme oxygenase (decycling) 1	ENST00000216117	0.61	5.21E-09	**0.75**	**5.84E-08**	0.29	4.10E-03
RNU6-531P	RNA. U6 small nuclear 531. pseudogene	ENST00000516694	1.71	1.32E-11	**1.97**	**5.54E-10**	1.77	3.42E-09
CRY1	cryptochrome circadian clock 1	NM_004075	1.06	2.39E-13	**1.15**	**3.83E-11**	0.89	3.66E-09
P4HA2	prolyl 4-hydroxylase. alpha polypeptide II	ENST00000401867	0.62	1.59E-10	**0.74**	**3.34E-09**	0.74	3.77E-09
FNIP1	folliculin interacting protein 1	NM_001008738	0.55	8.39E-10	**0.70**	**5.64E-09**	0.71	4.25E-09
ALDH2	aldehyde dehydrogenase 2 family (mitochondrial)	NM_000690	0.48	8.60E-09	**0.73**	**2.33E-09**	0.70	4.42E-09
KCMF1	potassium channel modulatory factor 1	NM_020122	0.65	8.39E-12	**0.76**	**2.34E-10**	0.64	5.10E-09
HSPA13	heat shock protein 70kDa family. member 13	NM_006948	0.66	7.78E-11	**0.70**	**1.22E-08**	0.73	5.53E-09

DOCK10	dedicator of cytokinesis 10	NM_001290263	0.47	6.85E-09	**0.63**	**1.53E-08**	0.48	1.00E-06
SDE2	SDE2 telomere maintenance homolog (S. pombe)	NM_152608	1.17	1.99E-14	**0.81**	**9.37E-09**	0.84	5.71E-09
LRIG1	leucine-rich repeats and immunoglobulin-like domains 1	ENST00000273261	0.41	7.26E-08	**0.70**	**2.99E-09**	0.67	6.26E-09
FNDC3A	fibronectin type III domain containing 3A	NM_001079673	0.70	4.89E-13	**0.94**	**1.22E-12**	0.59	6.48E-09
LARP6	La ribonucleoprotein domain family. member 6	NM_018357	0.65	8.84E-09	**0.95**	**5.54E-09**	0.71	5.30E-07
UBC	ubiquitin C	ENST00000536769	1.04	7.82E-12	**1.27**	**1.30E-10**	1.01	6.97E-09
OSER1	oxidative stress responsive serine-rich 1	NM_016470	0.79	1.07E-10	**1.00**	**9.46E-10**	0.89	7.10E-09
CEBPB	CCAAT/enhancer binding protein (C/EBP). beta	NM_001285878	0.68	8.96E-09	**1.40**	**1.21E-11**	0.97	8.37E-09
AIFM2	apoptosis-inducing factor. mitochondrion-associated. 2	NM_001198696	0.66	9.78E-09	**0.84**	**5.71E-08**	0.75	3.78E-07
ZNF267	zinc finger protein 267	NM_001265588	0.64	1.78E-08	**0.86**	**4.61E-08**	0.95	8.52E-09
ATP13A3	ATPase type 13A3	NM_024524	0.46	1.01E-08	**0.65**	**1.03E-08**	0.55	1.32E-07
BLOC1S2	biogenesis of lysosomal organelles complex-1. subunit 2	NM_001001342	0.52	1.04E-08	**0.66**	**7.60E-08**	0.62	1.92E-07
UBR3	ubiquitin protein ligase E3 component n-recognin 3 (putative)	ENST00000272793	0.77	3.55E-13	**0.74**	**4.82E-10**	0.62	9.23E-09
PSMD14	proteasome (prosome. macropain) 26S subunit. non-ATPase. 14	NM_005805	0.72	9.29E-12	**0.83**	**4.72E-10**	0.70	9.45E-09
CPT1A	carnitine palmitoyltransferase 1A (liver)	NM_001031847	-0.49	9.69E-09	**-0.78**	**1.14E-09**	-0.69	9.64E-09
PRR3	proline rich 3	NM_001077497	0.59	1.15E-08	**0.81**	**2.10E-08**	0.71	1.64E-07
PRR3	proline rich 3	NM_001077497	0.59	1.15E-08	**0.81**	**2.10E-08**	0.71	1.64E-07
PRR3	proline rich 3	NM_001077497	0.59	1.15E-08	**0.81**	**2.10E-08**	0.71	1.64E-07
TULP3	tubby like protein 3	NM_001160408	0.40	2.08E-08	**0.78**	**7.17E-11**	0.59	1.02E-08
SLC19A2	solute carrier family 19 (thiamine transporter). member 2	NM_006996	1.30	7.50E-17	**1.32**	**5.41E-14**	0.68	1.07E-08
HSD17B7P2	hydroxysteroid (17-beta) dehydrogenase 7 pseudogene 2	NR_003086	0.91	1.30E-08	**1.24**	**2.66E-08**	0.61	3.51E-04
EAF1	ELL associated factor 1	NM_033083	0.62	1.36E-08	**0.82**	**4.34E-08**	0.72	3.35E-07
ZBTB21	zinc finger and BTB domain containing 21	NM_001098402	0.79	3.33E-10	**0.85**	**3.61E-08**	0.91	1.14E-08
MBNL2	muscleblind-like splicing regulator 2	NM_144778	0.47	1.43E-08	**0.79**	**7.50E-10**	0.57	1.54E-07
42434	membrane-associated ring finger (C3HC4) 5	ENST00000467521	0.43	1.45E-08	**0.62**	**9.46E-09**	0.62	1.17E-08
FAM124B	family with sequence similarity 124B	NM_024785	-0.68	4.29E-10	**-0.98**	**3.16E-10**	-0.79	1.22E-08
PRICKLE1	prickle homolog 1 (Drosophila)	NM_001144881	-0.42	1.56E-08	**-0.59**	**1.95E-08**	-0.50	2.41E-07
SETD5	SET domain containing 5	NM_001080517	0.38	5.54E-08	**0.76**	**1.16E-10**	0.58	1.26E-08
PALMD	palmdelphin	NM_017734	-0.57	1.63E-08	**-0.84**	**8.00E-09**	-0.67	3.48E-07
TES	testis derived transcript (3 LIM domains)	NM_015641	0.61	1.63E-08	**0.88**	**1.36E-08**	0.56	1.12E-05
CCT4	chaperonin containing TCP1. subunit 4 (delta)	NM_001256721	0.46	1.24E-09	**0.59**	**6.28E-09**	0.57	1.27E-08
PI4K2B	phosphatidylinositol 4-kinase type 2 beta	NM_018323	0.68	1.47E-09	**0.77**	**6.20E-08**	0.85	1.28E-08

EPSTI1	epithelial stromal interaction 1 (breast)	NM_001002264	1.52	1.10E-13	1.53	6.47E-11	1.13	1.43E-08
PDE4DIP	phosphodiesterase 4D interacting protein	NM_001198834	0.86	6.52E-09	1.18	1.10E-08	1.17	1.43E-08
AHNAK2	AHNAK nucleoprotein 2	NM_138420	-0.55	1.61E-10	-0.89	1.44E-11	-0.60	1.62E-08
MAFG	v-maf avian musculoaponeurotic fibrosarcoma oncogene homolog G	NM_032711	0.59	1.86E-09	0.77	6.59E-09	0.73	1.69E-08
XXYLT1-AS2	XXYLT1 antisense RNA 2	NR_102710	-0.60	1.83E-08	-1.00	1.16E-09	-0.57	7.30E-06
ARID5A	AT rich interactive domain 5A (MRF1-like)	NM_212481	1.01	5.46E-13	1.01	3.59E-10	0.80	1.93E-08
GFPT1	glutamine--fructose-6-phosphate transaminase 1	NM_001244710	0.66	1.35E-11	0.72	1.65E-09	0.62	2.24E-08
N4BP1	NEDD4 binding protein 1	NM_153029	0.59	1.14E-09	0.73	1.37E-08	0.70	2.29E-08
IFNE	interferon. epsilon	NM_176891	1.14	2.23E-08	1.55	4.06E-08	1.02	1.79E-05
TNFRSF21	tumor necrosis factor receptor superfamily. member 21	NM_014452	-0.44	2.39E-08	-0.63	2.09E-08	-0.24	3.20E-03
KANSL1L	KAT8 regulatory NSL complex subunit 1-like	NM_152519	0.61	3.32E-08	1.21	1.08E-10	0.89	2.31E-08
AC226119.4	novel transcript	OTTHUMT00000359670	-0.55	2.47E-08	-0.88	2.93E-09	-0.46	5.27E-05
NPLOC4	nuclear protein localization 4 homolog (S. cerevisiae)	NM_017921	0.55	1.03E-10	0.73	3.90E-10	0.57	2.55E-08
AFF1	AF4/FMR2 family. member 1	NM_001166693	0.42	2.74E-08	0.71	1.11E-09	0.41	7.76E-06
CDKL5	cyclin-dependent kinase-like 5	NM_001037343	0.58	2.95E-08	0.88	9.85E-09	0.50	4.02E-05
GIMAP7	GTPase. IMAP family member 7	NM_153236	-0.68	3.10E-08	-1.04	8.76E-09	-0.80	5.93E-07
TMEM39A	transmembrane protein 39A	NM_018266	0.58	9.47E-11	0.66	4.26E-09	0.59	2.70E-08
SSH1	slingshot protein phosphatase 1	NM_001161330	0.80	3.43E-11	1.25	5.08E-12	0.77	2.78E-08
NFKB1	nuclear factor of kappa light polypeptide gene enhancer in B-cells 1	NM_001165412	0.69	8.57E-10	0.99	6.40E-10	0.79	3.02E-08
NEDD4L	neural precursor cell expressed. developmentally down-regulated 4-like. E3 ubiquitin protein ligase	NM_001144964	0.46	3.81E-08	0.78	2.31E-09	0.42	2.14E-05
NRBF2	nuclear receptor binding factor 2	NM_001282405	0.51	5.35E-08	0.75	2.85E-08	0.74	3.56E-08
RP11-153M3.1	60 kDa heat shock protein pseudogene	OTTHUMT00000409621	0.86	6.49E-10	1.19	1.09E-09	0.97	3.60E-08
SRXN1	sulfiredoxin 1	NM_080725	0.85	1.49E-11	0.75	5.85E-08	0.77	3.73E-08
NFIB	nuclear factor I/B	NM_001190737	-0.42	9.82E-09	-0.55	3.14E-08	-0.55	3.90E-08
EFCAB7	EF-hand calcium binding domain 7	NM_032437	0.84	1.29E-08	1.15	2.06E-08	1.11	3.90E-08
GULP1	GULP. engulfment adaptor PTB domain containing 1	NM_001252668	0.62	9.21E-09	1.03	5.61E-10	0.80	3.99E-08
KDM6B	lysine (K)-specific demethylase 6B	NM_001080424	0.82	2.42E-11	0.95	8.23E-10	0.76	4.12E-08
TNFSF18	tumor necrosis factor (ligand) superfamily. member 18	NM_005092	-1.03	2.38E-12	-0.83	5.96E-08	-0.84	4.37E-08
F3	coagulation factor III (thromboplastin. tissue factor)	NM_001178096	1.01	2.35E-11	0.92	4.49E-08	0.92	4.56E-08
CBLB	Cbl proto-oncogene B. E3 ubiquitin protein ligase	NM_170662	0.49	5.67E-08	0.68	7.47E-08	0.07	4.08E-01
CNKSR3	CNKSR family member 3	NM_173515	0.86	6.32E-10	0.96	3.24E-08	0.94	4.66E-08
GBP3	guanylate binding protein 3	NM_018284	0.63	2.12E-09	0.72	6.85E-08	0.74	4.94E-08
SCD5	stearoyl-CoA desaturase 5	NM_001037582	-0.40	6.73E-08	-0.74	6.43E-10	-0.46	1.53E-06

CBLL1	Cbl proto-oncogene-like 1. E3 ubiquitin protein ligase	NM_001284291	0.42	6.78E-08	0.63	2.27E-08	0.54	3.10E-07
PITPNB	phosphatidylinositol transfer protein. beta	NM_001284277	0.48	6.89E-08	0.67	7.22E-08	0.57	9.36E-07
NBPF25P	neuroblastoma breakpoint family. member 25. pseudogene	NR_104217	0.80	7.01E-08	1.24	1.62E-08	1.09	1.31E-07
CTH	cystathionine gamma-lyase	ENST00000346806	1.01	7.56E-09	1.52	2.68E-09	1.27	5.98E-08
SDPR	serum deprivation response	NM_004657	-0.61	6.68E-09	-1.02	3.37E-10	-0.75	6.42E-08
TXNL4B	thioredoxin-like 4B	NM_001142317	0.47	7.63E-08	0.76	6.87E-09	0.52	2.74E-06
ABCB1	ATP-binding cassette. sub-family B (MDR/TAP). member 1	NM_000927	0.45	4.40E-08	0.69	1.23E-08	0.62	7.20E-08
TXNIP	thioredoxin interacting protein	NM_006472	-0.78	7.33E-11	-0.96	8.01E-10	-0.73	7.72E-08
YTHDC1	YTH domain containing 1	NM_001031732	0.40	8.16E-08	0.82	1.28E-10	0.86	5.10E-11
MED13L	mediator complex subunit 13-like	NM_015335	0.46	9.05E-08	0.93	2.18E-10	0.65	9.76E-08
HSPA7	heat shock 70kDa protein 7 (HSP70B)	NR_024151	0.97	9.27E-08	2.24	1.71E-11	3.05	4.66E-14
KIT	v-kit Hardy-Zuckerman 4 feline sarcoma viral oncogene homolog	NM_000222	-0.60	9.40E-08	-0.87	6.67E-08	-0.26	2.52E-02
YKT6	YKT6 v-SNARE homolog (S. cerevisiae)	NM_006555	0.47	9.60E-08	0.74	2.00E-08	0.78	7.88E-09
USPL1	ubiquitin specific peptidase like 1	NM_005800	0.45	1.02E-07	0.71	1.57E-08	1.21	1.01E-12
ZNF773	zinc finger protein 773	NM_198542	0.72	1.13E-07	1.33	1.43E-09	1.49	2.01E-10
C11orf84	chromosome 11 open reading frame 84	XM_005273782	0.65	1.21E-07	1.18	1.83E-09	1.14	3.03E-09
STXBP5-AS1	STXBP5 antisense RNA 1	ENST00000606831	0.66	1.26E-07	1.38	1.79E-10	1.87	5.85E-13
NRBF2	nuclear receptor binding factor 2	NM_001282405	0.51	1.31E-07	0.78	4.30E-08	0.83	1.46E-08
ZSWIM6	zinc finger. SWIM-type containing 6	NM_020928	0.40	1.31E-07	1.01	6.04E-12	1.00	7.90E-12
INO80	INO80 complex subunit	NM_017553	0.39	1.31E-07	0.59	4.69E-08	0.93	1.64E-11
ITPR3	inositol 1.4.5-trisphosphate receptor. type 3	NM_002224	-0.39	1.33E-07	-0.57	6.29E-08	-0.47	1.53E-06
CAT	catalase	NM_001752	-0.38	1.37E-07	-0.57	5.33E-08	-0.59	3.55E-08
MYOZ2	myozenin 2	NM_016599	0.91	1.41E-07	1.77	6.13E-10	2.01	6.69E-11
RP11-272L13.3	novel transcript	OTTHUMT00000467510	0.62	1.43E-07	1.17	1.07E-09	1.19	8.09E-10
PDE4DIP	phosphodiesterase 4D interacting protein	AB042555	1.10	1.60E-07	1.73	3.25E-08	2.27	2.78E-10
SYNE1	spectrin repeat containing. nuclear envelope 1	NM_033071	0.38	1.64E-07	0.84	9.56E-11	0.94	1.02E-11
NFIL3	nuclear factor. interleukin 3 regulated	NM_005384	0.69	1.67E-07	1.17	8.72E-09	1.07	4.12E-08
EPHB4	EPH receptor B4	NM_004444	-0.41	1.68E-07	-0.73	4.42E-09	-0.84	3.42E-10
DCBLD1	discoidin. CUB and LCCL domain containing 1	NM_173674	-0.43	1.69E-07	-0.64	6.49E-08	-0.50	2.81E-06
ADAM17	ADAM metallopeptidase domain 17	NM_003183	0.40	1.78E-07	0.67	1.20E-08	0.51	7.52E-07
MIR31HG	MIR31 host gene (non-protein coding)	NR_027054	0.57	1.98E-07	0.87	5.36E-08	1.00	6.22E-09
MIR2909	microRNA 2909	NR_036056	0.54	2.20E-07	0.94	6.39E-09	0.65	1.94E-06
TRAF1	TNF receptor-associated factor 1	NM_001190945	0.66	2.21E-07	1.41	2.50E-10	1.02	6.45E-08
ZRANB1	zinc finger. RAN-binding domain containing 1	NM_017580	0.38	2.35E-07	0.83	1.97E-10	0.93	2.14E-11
MCU	mitochondrial calcium uniporter	NM_001270679	0.39	2.36E-07	0.66	1.29E-08	0.75	1.43E-09

IL3RA	interleukin 3 receptor. alpha (low affinity)	NM_001267713	0.50	2.40E-07	0.80	3.67E-08	1.10	1.63E-10
ACSL4	acyl-CoA synthetase long-chain family member 4	NM_004458	0.35	2.43E-07	0.59	1.62E-08	0.68	1.35E-09
NA	NA	NONHSAT135562	-0.53	2.52E-07	-0.89	1.32E-08	-0.83	4.97E-08
THSD1	thrombospondin. type I. domain containing 1	NM_018676	-0.49	2.60E-07	-0.85	8.30E-09	-0.51	1.72E-05
NT5C2	5-nucleotidase. cytosolic II	NM_001134373	0.45	2.75E-07	0.85	3.14E-09	0.83	4.72E-09
VEGFA	vascular endothelial growth factor A	NM_001025366	0.59	2.89E-07	1.51	9.42E-12	1.12	2.17E-09
TMEM19	transmembrane protein 19	NM_018279	-0.47	3.14E-07	-0.75	4.57E-08	-0.46	4.08E-05
LOC100132167	uncharacterized LOC100132167	BC006438	0.41	3.22E-07	0.67	2.66E-08	0.67	2.77E-08
ICAM1	intercellular adhesion molecule 1	NM_000201	0.54	3.45E-07	1.07	1.43E-09	1.21	1.47E-10
EVA1C	eva-1 homolog C (C. elegans)	NM_001286556	-0.51	3.53E-07	-0.84	2.94E-08	-0.41	3.69E-04
SLC29A1	solute carrier family 29 (equilibrative nucleoside transporter). member 1	NM_001078175	-0.41	3.60E-07	-0.71	1.14E-08	-0.68	2.33E-08
MCL1	myeloid cell leukemia 1	NM_001197320	0.32	3.63E-07	0.50	7.58E-08	0.65	1.03E-09
RNF19B	ring finger protein 19B	NM_001127361	0.41	3.64E-07	0.70	1.63E-08	0.67	3.14E-08
RNU6-1213P	RNA. U6 small nuclear 1213. pseudogene	ENST00000517075	1.04	4.22E-07	1.90	6.99E-09	2.17	6.91E-10
C3orf52	chromosome 3 open reading frame 52	NM_024616	0.45	4.36E-07	1.03	1.49E-10	1.04	1.31E-10
SLBP	stem-loop binding protein	NM_006527	0.45	4.74E-07	0.81	9.49E-09	1.06	7.07E-11
SMURF1	SMAD specific E3 ubiquitin protein ligase 1	NM_001199847	0.43	5.28E-07	0.94	4.71E-10	0.98	2.08E-10
CEP350	centrosomal protein 350kDa	NM_014810	0.34	5.72E-07	0.58	2.75E-08	0.78	1.66E-10
CHIC2	cysteine-rich hydrophobic domain 2	NM_012110	0.44	6.35E-07	0.91	1.04E-09	1.21	5.93E-12
CLEC14A	C-type lectin domain family 14. member A	NM_175060	-0.41	6.37E-07	-0.98	1.21E-10	-0.86	1.25E-09
RP11-385D13.1	novel tripartite motif-containing 16 (TRIM16) and CMT1A duplicated region transcript 1 (CDRT1) protein	OTTHUMT00000363271	0.69	6.97E-07	1.74	4.45E-11	1.92	6.74E-12
RB1CC1	RB1-inducible coiled-coil 1	NM_001083617	0.38	7.17E-07	0.65	4.21E-08	0.43	1.46E-05
KDM6A	lysine (K)-specific demethylase 6A	NM_001291415	0.40	7.39E-07	0.72	1.95E-08	0.69	3.62E-08
RP9P	retinitis pigmentosa 9 pseudogene	NR_003500	0.87	7.46E-07	1.66	6.31E-09	1.86	7.97E-10
CECR2	cat eye syndrome chromosome region. candidate 2	NM_001290046	0.63	7.53E-07	1.54	8.80E-11	1.35	8.69E-10
GPX3	glutathione peroxidase 3 (plasma)	NM_002084	0.55	8.20E-07	1.15	1.39E-09	0.95	3.36E-08
MMP10	matrix metallopeptidase 10 (stromelysin 2)	NM_002425	0.39	8.28E-07	1.23	7.39E-13	1.42	4.47E-14
FBXL12	F-box and leucine-rich repeat protein 12	NM_017703	0.43	9.06E-07	0.75	3.73E-08	0.95	5.69E-10
SH3RF1	SH3 domain containing ring finger 1	NM_020870	0.36	9.18E-07	0.64	2.98E-08	0.60	7.18E-08
STXBP5-AS1	STXBP5 antisense RNA 1	NR_034115	0.72	9.67E-07	1.93	2.16E-11	2.52	1.37E-13
LOC100507165	uncharacterized LOC100507165	XR_171923	0.38	1.03E-06	0.72	1.00E-08	0.60	1.89E-07
ANKLE2	ankyrin repeat and LEM domain containing 2	NM_015114	0.32	1.06E-06	0.91	6.35E-12	1.02	7.33E-13

CLDN12	claudin 12	NM_001185072	0.46	1.08E-06	**0.84**	**1.79E-08**	0.86	1.13E-08
JUP	junction plakoglobin	NM_002230	-0.41	1.08E-06	**-0.73**	**3.11E-08**	-0.87	1.61E-09
NFKBIA	nuclear factor of kappa light polypeptide gene enhancer in B-cells inhibitor. alpha	NM_020529	0.37	1.17E-06	**0.84**	**4.75E-10**	1.07	6.30E-12
NFIA	nuclear factor I/A	NM_001134673	-0.39	1.17E-06	**-0.74**	**1.03E-08**	-0.78	3.68E-09
TENC1	tensin like C1 domain containing phosphatase (tensin 2)	NM_015319	-0.44	1.29E-06	**-0.79**	**3.08E-08**	-0.47	4.80E-05
SOCS6	suppressor of cytokine signaling 6	NM_004232	0.47	1.35E-06	**0.86**	**2.64E-08**	1.00	1.85E-09
JUNB	jun B proto-oncogene	NM_002229	0.53	1.36E-06	**1.22**	**3.99E-10**	1.23	3.49E-10
GCOM1	GRINL1A complex locus 1	NM_001018090	0.37	1.38E-06	**0.71**	**1.19E-08**	0.74	6.41E-09
NA	NA	---	0.72	1.45E-06	**1.44**	**5.65E-09**	1.63	6.55E-10
TSEN15	TSEN15 tRNA splicing endonuclease subunit	NR_023349	0.46	1.46E-06	**1.02**	**8.94E-10**	1.21	3.93E-11
CD24	CD24 molecule	BC064619	-0.80	1.57E-06	**-1.56**	**1.17E-08**	-1.52	1.82E-08
STXBP5-AS1	STXBP5 antisense RNA 1	NR_034115	0.62	1.61E-06	**1.84**	**4.83E-12**	2.32	5.73E-14
LINC00622	long intergenic non-protein coding RNA 622	NR_036540	0.63	1.71E-06	**1.34**	**2.47E-09**	0.72	2.72E-05
NA	NA	TCONS_l2_00001476-XLOC_l2_001076	0.47	1.74E-06	**1.04**	**1.46E-09**	0.62	4.97E-06
RNU6-630P	RNA. U6 small nuclear 630. pseudogene	ENST00000391258	1.53	1.78E-06	**2.67**	**7.59E-08**	2.22	1.25E-06
VCAN	versican	NM_001164097	-0.33	1.97E-06	**-0.59**	**6.78E-08**	-0.47	2.13E-06
ZNF521	zinc finger protein 521	NM_015461	-0.42	2.01E-06	**-0.88**	**3.69E-09**	-0.74	6.71E-08
RGS4	regulator of G-protein signaling 4	NM_001102445	-0.46	2.11E-06	**-1.11**	**4.00E-10**	-0.56	1.75E-05
SPIRE1	spire-type actin nucleation factor 1	NM_001128626	0.37	2.14E-06	**0.71**	**1.71E-08**	0.68	3.12E-08
INPP5D	inositol polyphosphate-5-phosphatase. 145kDa	NM_001017915	-0.38	2.14E-06	**-0.83**	**1.97E-09**	-0.81	2.93E-09
TAPT1	transmembrane anterior posterior transformation 1	NM_153365	0.36	2.55E-06	**0.70**	**1.97E-08**	0.63	1.10E-07
ZNF697	zinc finger protein 697	NM_001080470	0.41	2.74E-06	**0.75**	**6.06E-08**	0.62	1.30E-06
AARS	alanyl-tRNA synthetase	NM_001605	0.34	3.19E-06	**0.80**	**9.77E-10**	0.96	3.64E-11
RANGAP1	Ran GTPase activating protein 1	ENST00000455915	0.42	3.55E-06	**0.93**	**3.43E-09**	1.01	8.48E-10
ADAM23	ADAM metallopeptidase domain 23	NM_003812	-0.28	3.61E-06	**-0.59**	**7.11E-09**	-0.38	5.68E-06
DFNB31	deafness. autosomal recessive 31	NM_001083885	0.51	3.89E-06	**1.00**	**2.93E-08**	0.90	1.65E-07
RBBP6	retinoblastoma binding protein 6	NM_006910	0.33	4.23E-06	**0.63**	**5.91E-08**	0.59	1.56E-07
LEPREL1	leprecan-like 1	NM_001134418	0.68	4.38E-06	**1.28**	**5.46E-08**	1.15	2.85E-07
PAPD5	PAP associated domain containing 5	NM_001040284	0.39	4.48E-06	**1.04**	**1.55E-10**	1.55	8.70E-14
DAPK1	death-associated protein kinase 1	NM_001288729	-0.28	4.52E-06	**-0.55**	**3.47E-08**	-0.52	8.32E-08
GABBR2	gamma-aminobutyric acid (GABA) B receptor. 2	NM_005458	-0.26	4.85E-06	**-0.76**	**4.17E-11**	-0.88	2.62E-12
PPP1R15B	protein phosphatase 1. regulatory subunit 15B	NM_032833	0.30	5.09E-06	**0.96**	**7.06E-12**	0.95	9.28E-12
NA	NA	NONHSAT112254	0.92	5.14E-06	**1.78**	**5.33E-08**	1.24	9.92E-06
DDIT4	DNA-damage-inducible transcript 4	NM_019058	0.37	5.35E-06	**1.08**	**3.99E-11**	0.85	2.67E-09

MCM3	minichromosome maintenance complex component 3	NM_001270472	-0.32	5.52E-06	**-0.65**	**2.47E-08**	-0.55	3.53E-07
MGAT5	mannosyl (alpha-1.6-)-glycoprotein beta-1.6-N-acetyl-glucosaminyltransferase	NM_002410	-0.29	5.74E-06	**-0.76**	**3.11E-10**	-0.71	1.02E-09
MAP1LC3B	microtubule-associated protein 1 light chain 3 beta	ENST00000564844	0.54	6.29E-06	**1.15**	**1.08E-08**	0.63	6.42E-05
LRRFIP2	leucine rich repeat (in FLII) interacting protein 2	NM_006309	0.28	6.36E-06	**0.53**	**5.72E-08**	0.46	6.59E-07
TRIB1	tribbles pseudokinase 1	NM_001282985	0.40	6.37E-06	**1.69**	**3.31E-14**	2.19	2.13E-16
CASP2	caspase 2. apoptosis-related cysteine peptidase	NM_001224	-0.35	7.04E-06	**-0.70**	**4.30E-08**	-0.41	8.48E-05
ALOXE3	arachidonate lipoxygenase 3	NM_001165960	0.55	7.06E-06	**1.84**	**4.35E-12**	2.02	7.04E-13
TBC1D3H	TBC1 domain family. member 3H	NM_001123392	0.38	7.70E-06	**0.74**	**6.62E-08**	0.83	8.97E-09
DUSP8	dual specificity phosphatase 8	NM_004420	0.36	7.86E-06	**1.32**	**7.15E-13**	1.42	1.87E-13
ARHGAP32	Rho GTPase activating protein 32	NM_001142685	0.32	8.04E-06	**0.77**	**2.49E-09**	0.56	3.90E-07
PLK3	polo-like kinase 3	NM_004073	0.35	8.20E-06	**0.87**	**1.10E-09**	0.90	6.44E-10
VIP	vasoactive intestinal peptide	NM_003381	0.49	8.26E-06	**1.13**	**4.90E-09**	1.12	5.39E-09
LOC729218	uncharacterized LOC729218	NR_103825	0.44	8.99E-06	**0.99**	**8.41E-09**	0.93	2.53E-08
SRSF8	serine/arginine-rich splicing factor 8	NM_032102	-0.34	9.00E-06	**-0.67**	**7.95E-08**	-0.49	7.25E-06
TGM2	transglutaminase 2	ENST00000361475	-0.25	9.38E-06	**-0.56**	**8.93E-09**	-0.52	2.65E-08
FAM196B	family with sequence similarity 196. member B	NM_001129891	-0.34	1.05E-05	**-0.85**	**2.15E-09**	-0.58	8.84E-07
LOC100288069	uncharacterized LOC100288069	NR_033908	0.30	1.11E-05	**0.97**	**1.54E-11**	0.63	2.95E-08
RFK	riboflavin kinase	ENST00000479197	-0.30	1.15E-05	**-0.67**	**1.22E-08**	-0.65	2.32E-08
EVC2	Ellis van Creveld syndrome 2	NM_001166136	0.45	1.20E-05	**1.23**	**3.70E-10**	1.31	1.17E-10
XPC	xeroderma pigmentosum. complementation group C	NM_001145769	0.32	1.22E-05	**0.72**	**1.42E-08**	0.76	4.80E-09
TBC1D3B	TBC1 domain family. member 3B	NM_001001417	0.34	1.22E-05	**0.76**	**1.73E-08**	0.84	2.74E-09
NA	NA	TCONS_l2_00005763-XLOC_l2_003052	0.46	1.31E-05	**1.09**	**5.67E-09**	1.29	3.03E-10
GKAP1	G kinase anchoring protein 1	NM_001135953	0.65	1.36E-05	**1.40**	**2.57E-08**	1.62	2.13E-09
NA	NA	ENST00000384563	1.13	1.43E-05	**3.76**	**1.29E-11**	3.59	3.01E-11
FRS2	fibroblast growth factor receptor substrate 2	NM_001042555	0.41	1.45E-05	**1.14**	**3.98E-10**	1.38	1.23E-11
SLC41A1	solute carrier family 41 (magnesium transporter). member 1	NM_173854	0.28	1.53E-05	**0.59**	**3.88E-08**	0.62	1.49E-08
BMX	BMX non-receptor tyrosine kinase	NM_001721	-0.35	1.56E-05	**-0.88**	**2.28E-09**	-0.87	2.75E-09
CREBBP	CREB binding protein	NM_001079846	0.26	1.72E-05	**0.62**	**8.25E-09**	0.48	5.07E-07
CHD2	chromodomain helicase DNA binding protein 2	NM_001271	0.30	1.73E-05	**0.89**	**1.71E-10**	1.23	3.98E-13
ARRB1	arrestin. beta 1	NM_004041	-0.26	1.78E-05	**-0.59**	**1.20E-08**	-0.52	1.08E-07
PDE8A	phosphodiesterase 8A	ENST00000485596	0.23	1.90E-05	**0.51**	**2.34E-08**	0.39	1.58E-06
STS	steroid sulfatase (microsomal). isozyme S	NM_000351	-0.28	1.92E-05	**-0.66**	**8.40E-09**	-0.69	3.76E-09
CCIN	calicin	NM_005893	0.58	2.06E-05	**1.29**	**2.83E-08**	1.84	5.69E-11
AEN	apoptosis enhancing nuclease	XM_005254967	0.31	2.08E-05	**0.71**	**2.14E-08**	0.89	4.32E-10

HUNK	hormonally up-regulated Neu-associated kinase	NM_014586	0.70	2.14E-05	**1.76**	**3.36E-09**	2.32	2.40E-11
CLK1	CDC-like kinase 1	NM_001162407	0.33	2.15E-05	**0.99**	**2.13E-10**	1.58	2.91E-14
RICTOR	RPTOR independent companion of MTOR. complex 2	NM_001285439	0.29	2.34E-05	**0.61**	**7.06E-08**	0.70	7.90E-09
MTHFD2	methylenetetrahydrofolate dehydrogenase (NADP+ dependent) 2. methenyltetrahydrofolate cyclohydrolase	NM_006636	0.30	2.36E-05	**1.58**	**4.20E-15**	1.54	7.08E-15
EEF2K	eukaryotic elongation factor-2 kinase	NM_013302	-0.30	2.41E-05	**-0.64**	**5.87E-08**	-0.51	1.72E-06
CROT	carnitine O-octanoyltransferase	NM_001143935	-0.44	2.75E-05	**-0.96**	**5.35E-08**	-0.77	1.68E-06
ITSN2	intersectin 2	NM_006277	0.24	2.90E-05	**0.63**	**1.82E-09**	0.58	8.77E-09
ARMCX6	armadillo repeat containing. X-linked 6	NM_001009584	-0.27	2.96E-05	**-0.59**	**5.60E-08**	-0.41	1.28E-05
C8orf88	chromosome 8 open reading frame 88	NM_001190972	0.52	2.97E-05	**1.32**	**4.40E-09**	1.49	5.04E-10
JUN	jun proto-oncogene	NM_002228	0.29	3.00E-05	**0.95**	**5.49E-11**	1.39	4.33E-14
SNORD29	small nucleolar RNA. C/D box 29	ENST00000538654	-0.46	3.29E-05	**-1.00**	**7.29E-08**	-0.20	1.13E-01
RWDD2B	RWD domain containing 2B	ENST00000472184	-0.33	3.40E-05	**-0.82**	**6.83E-09**	-0.95	5.60E-10
NQO1	NAD(P)H dehydrogenase. quinone 1	NM_000903	-0.25	3.54E-05	**-0.55**	**5.45E-08**	-0.68	1.50E-09
RAPGEF5	Rap guanine nucleotide exchange factor (GEF) 5	NM_012294	-0.40	3.56E-05	**-0.88**	**6.77E-08**	-0.60	1.80E-05
NHSL2	NHS-like 2	NM_001013627	-0.30	3.65E-05	**-0.66**	**7.97E-08**	-0.80	3.21E-09
NR4A1	nuclear receptor subfamily 4. group A. member 1	ENST00000550557	0.51	3.90E-05	**1.47**	**9.09E-10**	1.08	1.46E-07
GPN1	GPN-loop GTPase 1	NM_001145047	0.25	4.39E-05	**0.56**	**4.68E-08**	0.74	4.54E-10
MPZL2	myelin protein zero-like 2	NM_144765	-0.36	4.39E-05	**-0.85**	**3.09E-08**	-0.56	1.41E-05
SPRY4	sprouty homolog 4 (Drosophila)	NM_001293289	0.31	4.49E-05	**1.25**	**2.73E-12**	1.41	2.53E-13
LOC100288069	uncharacterized LOC100288069	NR_033908	0.29	4.51E-05	**0.94**	**1.20E-10**	0.72	1.29E-08
ZC3H12A	zinc finger CCCH-type containing 12A	NM_025079	0.32	5.00E-05	**0.89**	**1.72E-09**	1.42	3.20E-13
SNX9	sorting nexin 9	NM_016224	0.24	5.09E-05	**0.56**	**3.88E-08**	0.49	2.77E-07
PPARD	peroxisome proliferator-activated receptor delta	NM_001171818	0.29	6.07E-05	**0.69**	**3.36E-08**	0.61	2.72E-07
FAT4	FAT atypical cadherin 4	NM_001291285	-0.30	6.53E-05	**-0.91**	**5.31E-10**	-0.84	2.33E-09
MUM1	melanoma associated antigen (mutated) 1	NM_032853	0.30	7.31E-05	**0.70**	**6.25E-08**	0.82	5.38E-09
HDAC9	histone deacetylase 9	NM_001204144	0.32	7.52E-05	**1.14**	**4.57E-11**	0.96	1.08E-09
LRIF1	ligand dependent nuclear receptor interacting factor 1	NM_018372	0.34	7.55E-05	**1.02**	**1.25E-09**	1.62	2.54E-13
KCNT2	potassium channel. subfamily T. member 2	NM_001287819	0.49	7.59E-05	**1.22**	**2.56E-08**	1.77	3.37E-11
TLK2	tousled-like kinase 2	NM_001284333	0.25	7.62E-05	**0.70**	**2.98E-09**	0.61	2.62E-08
NA	NA	NONHSAT068218	0.28	7.68E-05	**0.73**	**1.47E-08**	0.84	1.39E-09
RAB39B	RAB39B. member RAS oncogene family	NM_171998	0.50	7.90E-05	**1.21**	**3.96E-08**	0.94	1.71E-06
PARP4	poly (ADP-ribose) polymerase family. member 4	NM_006437	-0.24	9.19E-05	**-0.63**	**1.28E-08**	-0.43	4.74E-06
LDB2	LIM domain binding 2	NM_001130834	-0.27	1.06E-04	**-0.80**	**2.52E-09**	-1.10	6.64E-12
RGS7BP	regulator of G-protein signaling 7 binding protein	NM_001029875	-0.40	1.11E-04	**-1.02**	**2.74E-08**	-0.84	6.47E-07

TP53I3	tumor protein p53 inducible protein 3	NM_004881	-0.26	1.16E-04	**-0.71**	**9.58E-09**	-0.52	1.44E-06
ARNTL	aryl hydrocarbon receptor nuclear translocator-like	NM_001030272	0.25	1.16E-04	**0.83**	**2.55E-10**	0.94	2.41E-11
PECAM1	platelet/endothelial cell adhesion molecule 1	NM_000442	-0.23	1.24E-04	**-0.58**	**4.97E-08**	-0.60	2.81E-08
TNFRSF9	tumor necrosis factor receptor superfamily. member 9	NM_001561	0.48	1.33E-04	**1.41**	**3.09E-09**	2.77	1.00E-14
ARSJ	arylsulfatase family. member J	NM_024590	0.26	1.36E-04	**0.75**	**5.35E-09**	1.11	4.65E-12
DPYSL2	dihydropyrimidinase-like 2	NM_001197293	-0.23	1.51E-04	**-0.57**	**5.38E-08**	-0.39	1.74E-05
NA	NA	NONHSAT097987	0.45	1.62E-04	**1.11**	**7.88E-08**	0.74	2.73E-05
SLC1A4	solute carrier family 1 (glutamate/neutral amino acid transporter). member 4	NM_003038	0.32	1.72E-04	**0.88**	**1.31E-08**	1.11	2.09E-10
CRYAB	crystallin. alpha B	NM_001289808	0.33	1.72E-04	**0.99**	**3.92E-09**	0.91	1.67E-08
EGR1	early growth response 1	NM_001964	0.38	1.79E-04	**1.04**	**1.49E-08**	1.89	3.25E-13
ALDH3A2	aldehyde dehydrogenase 3 family. member A2	NM_000382	-0.24	1.80E-04	**-0.61**	**4.82E-08**	-0.59	7.92E-08
ZBTB33	zinc finger and BTB domain containing 33	NM_001184742	-0.24	1.86E-04	**-0.61**	**5.81E-08**	-0.39	4.03E-05
LINC01061	long intergenic non-protein coding RNA 1061	NR_037596	0.28	1.98E-04	**1.02**	**1.45E-10**	0.69	1.16E-07
LOC101928451	serine/threonine-protein kinase tousled-like 2-like	XR_428252	0.26	2.02E-04	**0.96**	**1.23E-10**	0.71	2.77E-08
EXTL2	exostosin-like glycosyltransferase 2	NM_001261440	-0.35	2.08E-04	**-0.90**	**7.06E-08**	-0.73	1.72E-06
TRPS1	trichorhinophalangeal syndrome I	NM_001282903	0.31	2.12E-04	**0.83**	**3.07E-08**	0.99	1.39E-09
APMAP	adipocyte plasma membrane associated protein	NM_020531	-0.22	2.21E-04	**-0.59**	**2.35E-08**	-0.27	6.67E-04
PIP4K2B	phosphatidylinositol-5-phosphate 4-kinase. type II. beta	NM_003559	-0.27	2.55E-04	**-0.77**	**1.35E-08**	-0.42	7.39E-05
SRSF3	serine/arginine-rich splicing factor 3	NR_036610	0.25	2.58E-04	**0.64**	**6.64E-08**	0.77	3.62E-09
LOC101930489	uncharacterized LOC101930489	XR_249188	0.22	3.06E-04	**0.69**	**5.46E-09**	0.81	2.76E-10
SLC7A1	solute carrier family 7 (cationic amino acid transporter. y+ system). member 1	NM_003045	0.29	3.52E-04	**1.12**	**1.88E-10**	1.18	6.80E-11
HEATR3	HEAT repeat containing 3	NM_182922	-0.22	3.56E-04	**-0.59**	**5.24E-08**	-0.26	1.49E-03
LGR4	leucine-rich repeat containing G protein-coupled receptor 4	NM_018490	-0.36	4.18E-04	**-1.16**	**4.07E-09**	-0.90	2.48E-07
LOC101928706	uncharacterized LOC101928706	ENST00000440038	0.21	4.25E-04	**0.96**	**8.49E-12**	0.62	2.10E-08
PARP4	poly (ADP-ribose) polymerase family. member 4	NM_006437	-0.19	4.25E-04	**-0.56**	**2.27E-08**	-0.47	4.09E-07
IL6	interleukin 6	NM_000600	0.33	4.27E-04	**1.04**	**7.45E-09**	1.24	3.00E-10
LOC101930489	uncharacterized LOC101930489	XR_249188	0.23	4.57E-04	**0.67**	**1.87E-08**	0.96	3.70E-11
MAFK	v-maf avian musculoaponeurotic fibrosarcoma oncogene homolog K	NM_002360	0.37	4.60E-04	**1.03**	**7.12E-08**	1.17	9.16E-09
SIAH2	siah E3 ubiquitin protein ligase 2	NM_005067	0.27	4.62E-04	**0.74**	**5.76E-08**	0.77	3.03E-08
LOC101927270	uncharacterized LOC101927270	XR_252843	0.29	4.73E-04	**0.97**	**3.15E-09**	0.71	4.42E-07
ZCCHC8	zinc finger. CCHC domain containing 8	NM_017612	0.30	4.82E-04	**0.89**	**2.34E-08**	0.82	9.55E-08
PAQR3	progestin and adipoQ receptor family member III	ENST00000342820	0.29	5.93E-04	**1.09**	**5.25E-10**	1.75	7.72E-14

LOC100134822	uncharacterized LOC100134822	ENST00000446912	0.25	5.97E-04	0.88	1.51E-09	0.68	1.28E-07
RN7SKP172	RNA. 7SK small nuclear pseudogene 172	ENST00000411341	0.42	6.33E-04	1.46	2.62E-09	1.12	2.26E-07
CCDC84	coiled-coil domain containing 84	NM_198489	0.30	7.62E-04	0.96	1.18E-08	1.32	4.58E-11
TCEAL8	transcription elongation factor A (SII)-like 8	NM_001006684	-0.23	8.29E-04	-0.69	4.32E-08	-0.54	2.04E-06
LOC101929038	uncharacterized LOC101929038	XR_242175	0.22	8.40E-04	0.99	3.46E-11	0.63	8.84E-08
KLHL4	kelch-like family member 4	NM_019117	-0.29	8.62E-04	-0.89	3.98E-08	-0.71	1.44E-06
PPP1R3C	protein phosphatase 1. regulatory subunit 3C	NM_005398	0.30	8.77E-04	1.40	1.91E-11	1.92	4.67E-14
SLC7A5P1	solute carrier family 7 (amino acid transporter light chain. L system). member 5 pseudogene 1	NR_002593	0.30	8.89E-04	1.04	5.40E-09	0.99	1.28E-08
LOC101928451	serine/threonine-protein kinase tousled-like 2-like	XR_428252	0.26	8.90E-04	0.95	2.52E-09	0.69	4.91E-07
MEF2A	myocyte enhancer factor 2A	NM_001130926	0.20	9.40E-04	0.61	6.75E-08	0.43	1.04E-05
GARS	glycyl-tRNA synthetase	NM_002047	0.17	1.07E-03	0.69	5.39E-10	0.84	1.67E-11
SLC44A1	solute carrier family 44 (choline transporter). member 1	NM_001286730	-0.19	1.14E-03	-0.60	5.29E-08	-0.54	2.75E-07
RNA5SP148	RNA. 5S ribosomal pseudogene 148	ENST00000516527	0.46	1.15E-03	1.65	4.93E-09	0.62	1.85E-03
LINC01000	long intergenic non-protein coding RNA 1000	NR_024368	0.22	1.17E-03	0.98	1.07E-10	0.64	1.67E-07
MKI67	marker of proliferation Ki-67	NM_001145966	-0.19	1.34E-03	-0.63	2.68E-08	-0.44	5.40E-06
CREB5	cAMP responsive element binding protein 5	NM_004904	0.21	1.41E-03	0.69	3.39E-08	0.70	2.87E-08
LOC100132062	uncharacterized LOC100132062	uc001aau.3	0.20	1.49E-03	1.02	1.31E-11	0.67	2.35E-08
ANKFN1	ankyrin-repeat and fibronectin type III domain containing 1	NM_153228	0.31	1.64E-03	2.25	2.30E-14	2.35	9.50E-15
PDZD2	PDZ domain containing 2	NM_178140	0.32	1.75E-03	1.12	1.83E-08	1.37	5.92E-10
DUSP5	dual specificity phosphatase 5	NM_004419	0.19	1.79E-03	1.08	2.36E-12	1.21	2.76E-13
LOC729737	uncharacterized LOC729737	NR_039983	0.20	1.84E-03	1.27	4.62E-13	0.82	1.57E-09
CARS	cysteinyl-tRNA synthetase	NM_001014437	0.18	1.86E-03	0.94	1.01E-11	1.17	1.77E-13
CYYR1	cysteine/tyrosine-rich 1	AK304124	-0.29	1.90E-03	-1.14	3.03E-09	-0.94	7.54E-08
NA	NA	---	0.19	2.04E-03	0.73	7.85E-09	0.57	3.33E-07
LINC01000	long intergenic non-protein coding RNA 1000	NR_024368	0.22	2.14E-03	1.01	2.53E-10	0.61	1.17E-06
LINC01000	long intergenic non-protein coding RNA 1000	NR_024368	0.24	2.39E-03	1.06	7.00E-10	0.69	7.05E-07
LINC01000	long intergenic non-protein coding RNA 1000	NR_024368	0.24	2.39E-03	1.06	7.00E-10	0.69	7.05E-07
LINC01000	long intergenic non-protein coding RNA 1000	NR_024368	0.21	2.72E-03	0.95	6.65E-10	0.64	4.14E-07
MAP4K3	mitogen-activated protein kinase kinase kinase kinase 3	NM_003618	0.17	3.08E-03	0.64	2.09E-08	0.60	5.53E-08
NA	NA	NONHSAT128401	0.28	4.79E-03	1.08	2.16E-08	0.91	3.06E-07
TLK2	tousled-like kinase 2	NM_001284333	0.17	5.13E-03	0.79	1.17E-09	0.62	6.55E-08
DUSP10	dual specificity phosphatase 10	NR_111940	0.24	5.47E-03	0.93	4.58E-08	1.35	7.15E-11
MYO10	myosin X	NM_012334	0.14	5.71E-03	0.56	2.15E-08	0.26	5.61E-04
CEACAM19	carcinoembryonic antigen-related cell adhesion molecule 19	ENST00000480278	-0.17	6.64E-03	0.71	1.17E-08	0.47	6.92E-06
OXTR	oxytocin receptor	NM_000916	0.22	8.44E-03	0.88	5.10E-08	1.07	1.80E-09
LOC101928344	protein GVQW1-like	XM_006726770	0.21	9.39E-03	0.92	2.54E-08	0.70	1.62E-06

IL1RL1	interleukin 1 receptor-like 1	NM_003856	0.11	1.06E-02	**0.56**	**2.83E-09**	0.40	7.81E-07
NA	NA	TCONS_l2_00025851-XLOC_l2_013383	0.22	1.17E-02	**1.00**	**1.15E-08**	0.82	2.84E-07
PIK3R3	phosphoinositide-3-kinase. regulatory subunit 3 (gamma)	NM_001114172	-0.19	1.18E-02	**-0.92**	**4.85E-09**	-1.01	9.79E-10
ZNF75A	zinc finger protein 75a	NM_153028	0.18	1.27E-02	**0.80**	**3.59E-08**	0.76	8.87E-08
DNAJC18	DnaJ (Hsp40) homolog. subfamily C. member 18	NM_152686	0.18	1.38E-02	**0.77**	**4.40E-08**	0.39	3.76E-04
ESM1	endothelial cell-specific molecule 1	NM_007036	0.18	1.45E-02	**1.02**	**9.79E-10**	0.72	2.85E-07
LINC01347	long intergenic non-protein coding RNA 1347	NR_029401	0.16	1.74E-02	**0.89**	**8.78E-10**	0.66	1.47E-07
LINC-PINT	long intergenic non-protein coding RNA. p53 induced transcript	NR_034120	0.22	2.31E-02	**1.31**	**1.52E-09**	1.40	4.16E-10
NAV2	neuron navigator 2	NM_001111018	0.12	2.67E-02	**0.71**	**1.37E-09**	0.65	6.38E-09
NA	NA	---	0.17	2.79E-02	**0.84**	**3.70E-08**	0.46	1.53E-04
OTUD1	OTU deubiquitinase 1	NM_001145373	0.15	3.23E-02	**0.84**	**1.06E-08**	1.04	3.00E-10
GEMIN5	gem (nuclear organelle) associated protein 5	NM_001252156	-0.12	3.36E-02	**-0.61**	**3.19E-08**	-0.23	5.02E-03
HAS2	hyaluronan synthase 2	NM_005328	0.19	3.80E-02	**1.05**	**1.69E-08**	1.38	1.57E-10
LOC101928451	serine/threonine-protein kinase tousled-like 2-like	XR_428252	0.14	4.12E-02	**0.78**	**1.90E-08**	0.64	4.87E-07
LOC101926901	uncharacterized LOC101926901	ENST00000415989	0.16	4.24E-02	**0.94**	**9.73E-09**	0.72	6.45E-07
KLF6	Kruppel-like factor 6	NM_001160124	0.12	4.49E-02	**0.98**	**2.44E-11**	1.14	1.41E-12
ATF4	activating transcription factor 4	NM_001675	0.10	4.74E-02	**0.69**	**1.49E-09**	0.89	1.27E-11
RP11-819C21.1	novel transcript	OTTHUMT00000421595	0.23	4.99E-02	**1.32**	**2.57E-08**	1.66	5.20E-10
PKD1P1	polycystic kidney disease 1 (autosomal dominant) pseudogene 1	NR_036447	0.10	5.91E-02	**0.82**	**2.05E-10**	0.82	2.16E-10
NA	NA	---	0.12	6.10E-02	**0.97**	**1.67E-10**	0.42	7.00E-05
ATP6V0D2	ATPase. H+ transporting. lysosomal 38kDa. V0 subunit d2	ENST00000285393	0.18	6.64E-02	**2.33**	**7.42E-14**	2.17	2.97E-13
NA	NA	NONHSAT127377	0.22	7.05E-02	**1.30**	**6.95E-08**	1.74	5.04E-10
OLAH	oleoyl-ACP hydrolase	NM_001039702	-0.22	7.13E-02	**1.32**	**7.18E-08**	1.80	3.39E-10
RP11-3L8.3	novel transcript	OTTHUMT00000051732	-0.16	7.50E-02	**0.99**	**7.25E-08**	1.22	2.45E-09
PELI1	pellino E3 ubiquitin protein ligase 1	NM_020651	0.09	8.23E-02	**0.56**	**6.37E-08**	0.76	3.68E-10
PKD1	polycystic kidney disease 1 (autosomal dominant)	NM_000296	0.12	9.69E-02	**0.88**	**1.89E-08**	0.51	4.98E-05
PKD1P1	polycystic kidney disease 1 (autosomal dominant) pseudogene 1	NR_036447	0.08	1.39E-01	**0.88**	**5.54E-11**	0.82	1.99E-10
C17orf51	chromosome 17 open reading frame 51	NM_001113434	0.11	1.41E-01	**0.95**	**5.48E-09**	1.00	2.09E-09
TRIO	trio Rho guanine nucleotide exchange factor	NM_007118	0.07	1.42E-01	**0.57**	**4.67E-08**	0.37	3.04E-05
RASGRF2	Ras protein-specific guanine nucleotide-releasing factor 2	NM_006909	0.10	1.45E-01	**0.83**	**1.79E-08**	0.84	1.66E-08
UBALD2	UBA-like domain containing 2	NM_182565	0.08	1.56E-01	**1.03**	**2.27E-11**	0.90	2.90E-10
SOS2	son of sevenless homolog 2 (Drosophila)	NM_006939	-0.08	1.63E-01	**0.73**	**1.50E-08**	0.54	1.89E-06
RP11-7F17.7	novel transcript	OTTHUMT00000414297	0.14	1.63E-01	**1.10**	**6.98E-08**	0.65	1.08E-04
LOC101928451	serine/threonine-protein kinase tousled-like 2-like	XM_006716223	0.09	1.66E-01	**0.79**	**8.65E-09**	0.64	2.81E-07

SDC4	syndecan 4	NM_002999	0.09	1.79E-01	**0.83**	**1.14E-08**	1.46	4.22E-13
LOC101930489	uncharacterized LOC101930489	XR_249188	0.07	2.00E-01	**0.69**	**8.36E-09**	0.36	9.81E-05
LOC101060341	putative uncharacterized protein FLJ46235-like	XM_005276289	0.07	2.60E-01	**0.87**	**1.20E-09**	0.66	1.43E-07
ANKRD11	ankyrin repeat domain 11	NM_001256182	0.06	2.79E-01	**0.76**	**7.70E-09**	0.58	5.62E-07
NA	NA	ENST00000365346	0.07	3.38E-01	**0.90**	**5.60E-09**	0.51	2.66E-05
NPIPB8	nuclear pore complex interacting protein family. member B8	ENST00000357796	0.06	4.01E-01	**0.88**	**6.45E-09**	0.54	1.02E-05
RHOB	ras homolog family member B	NM_004040	0.04	4.22E-01	**0.89**	**8.02E-11**	0.90	7.02E-11
GRPEL2	GrpE-like 2. mitochondrial (E. coli)	NM_152407	0.05	4.78E-01	**1.04**	**2.31E-10**	1.07	1.35E-10
CTB-134H23.2	novel protein similar to nuclear pore complex interacting protein-like 1 NPIPL1	OTTHUMT00000409230	0.04	5.00E-01	**0.75**	**1.25E-09**	0.53	3.91E-07
SLC22A15	solute carrier family 22. member 15	NM_018420	-0.05	5.15E-01	**1.04**	**5.00E-09**	0.84	1.82E-07
CSF3	colony stimulating factor 3 (granulocyte)	NM_000759	0.06	5.29E-01	**1.75**	**1.43E-11**	3.09	2.38E-16
DLGAP1-AS2	DLGAP1 antisense RNA 2	ENST00000572856	0.03	6.63E-01	**1.07**	**1.44E-09**	1.13	5.52E-10
NPIPB5	nuclear pore complex interacting protein family. member B5	NM_001135865	-0.02	6.83E-01	**0.70**	**1.07E-08**	0.38	7.60E-05
NPIPA5	nuclear pore complex interacting protein family. member A5	NM_001277325	0.02	6.87E-01	**0.80**	**1.21E-09**	0.59	2.26E-07
PKD1P5	polycystic kidney disease 1 (autosomal dominant) pseudogene 5	ENST00000532415	0.02	7.69E-01	**0.84**	**8.23E-10**	0.91	1.76E-10
LOC613037	nuclear pore complex interacting protein pseudogene	NR_002555	0.01	7.73E-01	**0.72**	**2.14E-09**	0.37	4.02E-05
NPIPA3	nuclear pore complex interacting protein family. member A3	NM_001277323	-0.02	7.91E-01	**0.86**	**2.67E-09**	0.60	9.39E-07
NPIPB5	nuclear pore complex interacting protein family. member B5	NM_001135865	0.01	8.02E-01	**0.74**	**1.09E-09**	0.37	3.83E-05
NPIPB11	nuclear pore complex interacting protein family. member B11	ENST00000524087	0.01	8.38E-01	**0.76**	**2.73E-09**	0.39	4.77E-05
EGFR	epidermal growth factor receptor	NM_005228	-0.01	8.94E-01	**0.63**	**7.92E-08**	0.55	5.76E-07
NPIPA1	nuclear pore complex interacting protein family. member A1	ENST00000472413	-0.01	9.25E-01	**0.83**	**6.96E-10**	0.77	2.76E-09
AC090044.2	novel transcript	OTTHUMT00000337494	0.00	9.78E-01	**0.86**	**1.71E-08**	1.23	2.96E-11

NA; not applicable. FC; fold change n= 6 for 12 h. *n*= 3 for 18 h and 24 h.

Table S3: Altered gene expression due to NOX5-β expression at 24 h (bold, highlighted in yellow).

GeneName	GeneDescription	TranscriptID	N12h *vs* G12h		N18h *vs* G18h		N24h *vs* G24h	
			logFC	*p-value*	logFC	*p-value*	logFC	*p-value*
FAM129A	family with sequence similarity 129. member A	NM_052966	1.45	1.20E-17	2.44	3.27E-19	**2.96**	**6.24E-21**
DNAJA4	DnaJ (Hsp40) homolog. subfamily A. member 4	NM_001130182	2.19	9.83E-20	2.94	2.90E-19	**3.01**	**1.68E-19**
PMAIP1	phorbol-12-myristate-13-acetate-induced protein 1	NM_021127	0.93	1.84E-12	2.41	1.32E-17	**2.69**	**1.43E-18**
E2F7	E2F transcription factor 7	NM_203394	0.71	8.35E-11	2.20	2.21E-17	**2.48**	**1.84E-18**
DNAJB1	DnaJ (Hsp40) homolog. subfamily B. member 1	NM_006145	1.95	2.72E-20	2.30	1.04E-18	**2.13**	**5.07E-18**
PTGS2	prostaglandin-endoperoxide synthase 2 (prostaglandin G/H synthase and cyclooxygenase)	NM_000963	3.80	3.74E-24	3.89	2.89E-21	**2.68**	**5.83E-18**
RND1	Rho family GTPase 1	ENST00000548445	3.09	2.20E-20	3.42	3.17E-18	**3.25**	**8.99E-18**
CHRNA5	cholinergic receptor. nicotinic. alpha 5 (neuronal)	NM_000745	1.30	6.71E-17	1.78	1.31E-16	**1.99**	**1.44E-17**
IL7R	interleukin 7 receptor	NM_002185	2.17	1.43E-19	2.62	3.61E-18	**2.36**	**3.12E-17**
ACTRT3	actin-related protein T3	NM_032487	1.55	1.52E-14	2.48	1.35E-15	**2.94**	**4.50E-17**
CCRN4L	CCR4 carbon catabolite repression 4-like (S. cerevisiae)	NM_012118	1.92	4.22E-19	2.05	1.30E-16	**2.13**	**6.21E-17**
SAT1	spermidine/spermine N1-acetyltransferase 1	NM_002970	0.82	2.04E-13	1.76	4.50E-17	**1.73**	**6.60E-17**
SLC7A5	solute carrier family 7 (amino acid transporter light chain. L system). member 5	NM_003486	0.69	1.39E-08	2.39	7.59E-16	**2.69**	**7.18E-17**
GPR89A	G protein-coupled receptor 89A	XM_006711492	1.10	4.66E-16	1.44	2.24E-15	**1.69**	**8.05E-17**
NFKBIZ	nuclear factor of kappa light polypeptide gene enhancer in B-cells inhibitor. zeta	NM_001005474	0.84	2.64E-10	2.32	7.66E-16	**2.60**	**8.20E-17**
GPR89A	G protein-coupled receptor 89A	NM_001097612	1.05	1.23E-15	1.44	2.19E-15	**1.7**	**8.33E-17**
DUSP1	dual specificity phosphatase 1	NM_004417	0.76	2.57E-10	1.84	1.11E-14	**2.31**	**1.20E-16**
HSPA4L	heat shock 70kDa protein 4-like	NM_014278	1.22	9.72E-16	1.52	1.25E-14	**1.90**	**1.45E-16**
STXBP5-AS1	STXBP5 antisense RNA 1	NR_034115	0.56	2.46E-08	1.46	3.28E-13	**2.12**	**2.15E-16**
CHAC1	ChaC. cation transport regulator homolog 1 (E. coli)	ENST00000446533	1.01	1.68E-09	3.74	1.72E-17	**3.28**	**2.49E-16**
GADD45B	growth arrest and DNA-damage-inducible. beta	NM_015675	0.88	6.24E-13	1.53	1.25E-14	**1.86**	**2.66E-16**
IER5	immediate early response 5	NM_016545	1.01	1.06E-12	1.85	6.09E-15	**2.16**	**2.98E-16**
MXD1	MAX dimerization protein 1	NM_001202513	1.46	2.88E-17	2.08	2.44E-17	**1.81**	**3.86E-16**
ULBP1	UL16 binding protein 1	NM_025218	0.96	8.52E-10	2.12	2.11E-13	**2.91**	**3.91E-16**
RP11-274H2.3	novel transcript	OTTHUMT00000355183	2.01	1.27E-18	2.29	9.69E-17	**2.14**	**3.92E-16**
JMJD1C	jumonji domain containing 1C	NM_001282948	0.61	3.49E-11	1.44	1.99E-15	**1.56**	**4.05E-16**
BAG3	BCL2-associated athanogene 3	NM_004281	1.93	3.06E-19	2.00	1.62E-16	**1.91**	**4.28E-16**
KITLG	KIT ligand	NM_000899	1.52	7.96E-17	2.12	1.05E-16	**1.97**	**4.77E-16**
BMP2	bone morphogenetic protein 2	NM_001200	1.60	1.08E-16	2.16	2.80E-16	**2.04**	**9.00E-16**
ERN1	endoplasmic reticulum to nucleus signaling 1	NM_001433	1.32	1.37E-14	2.24	4.02E-16	**2.12**	**1.16E-15**
SESN2	sestrin 2	NM_031459	1.70	3.90E-15	2.81	1.70E-16	**2.53**	**1.37E-15**
SIX4	SIX homeobox 4	NM_017420	0.86	1.31E-11	1.55	1.40E-13	**1.95**	**1.39E-15**
GDF15	growth differentiation factor 15	NM_004864	0.67	3.73E-11	1.54	2.78E-15	**1.58**	**1.80E-15**

CAP2	CAP. adenylate cyclase-associated protein. 2 (yeast)	NM_006366	0.85	5.80E-15	1.09	4.04E-14	**1.27**	**1.90E-15**
C7orf60	chromosome 7 open reading frame 60	NM_152556	0.99	4.36E-12	1.79	3.96E-14	**2.07**	**2.35E-15**
LOC727896	cysteine and histidine-rich domain (CHORD) containing 1 pseudogene	NR_026659	1.33	1.29E-15	1.64	1.89E-14	**1.80**	**3.16E-15**
HSPD1	heat shock 60kDa protein 1 (chaperonin)	NM_002156	1.34	9.15E-18	1.36	7.26E-15	**1.40**	**3.76E-15**
KIF21A	kinesin family member 21A	NM_001173463	1.55	2.58E-17	1.91	4.47E-16	**1.70**	**4.18E-15**
NA	NA	---	1.500	2.08E-15	1.86	2.82E-14	**2.04**	**4.78E-15**
USP53	ubiquitin specific peptidase 53	NM_019050	0.93	5.15E-14	1.45	7.98E-15	**1.49**	**4.92E-15**
BACH1	BTB and CNC homology 1. basic leucine zipper transcription factor 1	NR_027655	1.12	1.74E-14	1.61	1.19E-14	**1.67**	**5.86E-15**
NA	NA	RPTR-U57609-1	-7.43	5.14E-19	-6.37	1.18E-14	**-6.56**	**6.76E-15**
NA	NA	RPTR-U43284-1	-7.43	5.14E-19	-6.37	1.18E-14	**-6.56**	**6.76E-15**
NA	NA	RPTR-AB076373-2	-7.43	5.14E-19	-6.37	1.18E-14	**-6.56**	**6.76E-15**
PPP1R15A	protein phosphatase 1. regulatory subunit 15A	NM_014330	1.16	8.86E-13	1.73	3.11E-13	**2.08**	**8.84E-15**
CPEB4	cytoplasmic polyadenylation element binding protein 4	NM_030627	1.42	6.06E-17	1.91	1.56E-16	**1.56**	**9.34E-15**
RP11-212D19.4	novel transcript. RBM7-REXO2 readthrough	OTTHUMT00000399017	2.51	1.98E-13	3.5	2.55E-13	**4.13**	**1.03E-14**
LOC100128233	uncharacterized LOC100128233	NR_103769	0.94	4.35E-09	2.15	6.61E-13	**2.66**	**1.05E-14**
TNFAIP3	tumor necrosis factor. alpha-induced protein 3	NM_001270507	1.20	5.69E-14	1.72	4.34E-14	**1.83**	**1.28E-14**
NAA16	N(alpha)-acetyltransferase 16. NatA auxiliary subunit	NM_001110798	0.91	3.86E-13	1.44	3.87E-14	**1.51**	**1.51E-14**
MLKL	mixed lineage kinase domain-like	NM_152649	0.71	3.62E-11	1.20	1.16E-12	**1.49**	**1.86E-14**
HSPH1	heat shock 105kDa/110kDa protein 1	NM_001286503	1.35	1.53E-17	1.41	6.52E-15	**1.34**	**1.93E-14**
MB21D1	Mab-21 domain containing 1	NM_138441	1.02	3.59E-10	2.02	6.35E-13	**2.41**	**2.08E-14**
TUBE1	tubulin. epsilon 1	NM_016262	0.57	6.14E-09	1.22	3.75E-12	**1.60**	**2.08E-14**
GOT1	glutamic-oxaloacetic transaminase 1. soluble	NM_002079	0.99	7.08E-13	1.75	8.15E-15	**1.67**	**2.11E-14**
IL1A	interleukin 1. alpha	NM_000575	1.35	5.27E-13	2.04	1.44E-13	**2.19**	**3.66E-14**
HSPA1A	heat shock 70kDa protein 1A	NM_005345	1.46	5.46E-19	1.38	1.97E-15	**1.17**	**5.07E-14**
KDM7A	lysine (K)-specific demethylase 7A	NM_030647	1.61	1.28E-16	2.02	1.52E-15	**1.66**	**6.87E-14**
TSPYL2	TSPY-like 2	ENST00000556808	1.21	3.27E-15	1.21	2.84E-12	**1.46**	**7.97E-14**
TMEM217	transmembrane protein 217	NM_001162900	0.87	1.93E-10	1.67	5.53E-13	**1.85**	**7.99E-14**
CCDC117	coiled-coil domain containing 117	NM_173510	0.80	1.09E-11	1.24	2.15E-12	**1.47**	**8.43E-14**
DUSP16	dual specificity phosphatase 16	NM_030640	0.68	1.86E-11	1.08	2.11E-12	**1.28**	**9.15E-14**
FGF2	fibroblast growth factor 2 (basic)	NM_002006	0.62	2.31E-11	1.01	1.68E-12	**1.16**	**1.16E-13**
RAB23	RAB23. member RAS oncogene family	NM_001278666	0.96	7.49E-13	1.51	8.40E-14	**1.48**	**1.24E-13**
FKBP4	FK506 binding protein 4. 59kDa	NM_002014	0.98	1.37E-14	1.02	4.80E-12	**1.23**	**1.27E-13**
HSPA1A	heat shock 70kDa protein 1A	NM_005345	1.45	1.91E-18	1.37	6.50E-15	**1.17**	**1.43E-13**
HSPA1B	heat shock 70kDa protein 1B	NM_005346	1.43	3.60E-18	1.39	6.61E-15	**1.18**	**1.58E-13**
RP11-762H8.4	novel transcript . sense intronic to WDR61	OTTHUMT00000471376	1.04	8.69E-10	1.31	6.51E-09	**2.31**	**1.86E-13**
PPID	peptidylprolyl isomerase D	NM_005038	0.71	1.59E-10	0.99	1.86E-10	**1.43**	**1.97E-13**

AHSA1	AHA1. activator of heat shock 90kDa protein ATPase homolog 1 (yeast)	NM_012111	1.05	1.15E-14	1.13	2.45E-12	**1.28**	**2.15E-13**
CEBPG	CCAAT/enhancer binding protein (C/EBP). gamma	NM_001252296	0.59	3.90E-09	1.59	2.23E-14	**1.41**	**2.28E-13**
NXT2	nuclear transport factor 2-like export factor 2	NM_001242617	0.63	3.97E-08	1.11	9.77E-10	**1.73**	**2.38E-13**
KBTBD8	kelch repeat and BTB (POZ) domain containing 8	NM_032505	0.97	5.52E-11	1.49	1.17E-11	**1.81**	**2.85E-13**
IRAK2	interleukin-1 receptor-associated kinase 2	NM_001570	0.90	1.63E-10	1.65	1.28E-12	**1.78**	**2.94E-13**
XBP1	X-box binding protein 1	NM_001079539	0.49	2.42E-10	1.05	9.60E-14	**0.98**	**3.37E-13**
ABHD3	abhydrolase domain containing 3	NM_138340	1.30	1.39E-12	1.58	2.55E-11	**1.97**	**3.65E-13**
GBE1	glucan (1.4-alpha-). branching enzyme 1	ENST00000429644	0.52	3.31E-09	1.03	7.71E-12	**1.20**	**3.85E-13**
HSPA1B	heat shock 70kDa protein 1B	NM_005346	1.25	1.30E-17	1.23	1.68E-14	**1.05**	**3.92E-13**
HSPA1A	heat shock 70kDa protein 1A	NM_005345	1.33	3.05E-18	1.26	1.05E-14	**1.04**	**4.03E-13**
TRAF6	TNF receptor-associated factor 6. E3 ubiquitin protein ligase	NM_004620	0.6	4.94E-10	1.07	6.14E-12	**1.23**	**4.08E-13**
LRRC8B	leucine rich repeat containing 8 family. member B	NM_001134476	1.01	3.23E-16	1.03	1.96E-13	**1.00**	**4.13E-13**
HECW2	HECT. C2 and WW domain containing E3 ubiquitin protein ligase 2	NM_020760	0.71	4.08E-11	1.26	6.56E-13	**1.28**	**4.57E-13**
RGS2	regulator of G-protein signaling 2	NM_002923	0.64	4.20E-09	1.23	1.67E-11	**1.48**	**4.72E-13**
HSPA1A	heat shock 70kDa protein 1A	NM_005345	1.33	2.81E-18	1.24	1.08E-14	**1.02**	**4.79E-13**
HSPA1A	heat shock 70kDa protein 1A	NM_005345	1.33	2.81E-18	1.24	1.08E-14	**1.02**	**4.79E-13**
HSPA1A	heat shock 70kDa protein 1A	NM_005345	1.33	2.81E-18	1.24	1.08E-14	**1.02**	**4.79E-13**
NOP58	NOP58 ribonucleoprotein	ENST00000264279	0.49	1.73E-10	0.72	9.14E-11	**0.95**	**5.39E-13**
PRKACB	protein kinase. cAMP-dependent. catalytic. beta	NM_001242857	0.58	3.88E-10	0.78	1.10E-09	**1.17**	**5.64E-13**
CXCL8	chemokine (C-X-C motif) ligand 8	NM_000584	2.28	5.88E-17	2.23	9.11E-14	**2.03**	**5.65E-13**
NAV3	neuron navigator 3	NM_001024383	0.57	1.62E-10	1.01	2.69E-12	**1.09**	**5.75E-13**
HBEGF	heparin-binding EGF-like growth factor	NM_001945	0.49	1.10E-08	1.02	1.35E-11	**1.20**	**6.46E-13**
HSPA1B	heat shock 70kDa protein 1B	NM_005346	1.47	9.72E-18	1.36	4.56E-14	**1.18**	**7.30E-13**
GXYLT2	glucoside xylosyltransferase 2	NM_001080393	1.08	1.76E-08	1.98	1.95E-10	**2.66**	**7.69E-13**
ARG2	arginase 2	NM_001172	0.94	2.30E-13	1.48	3.27E-14	**1.25**	**8.53E-13**
OTULIN	OTU deubiquitinase with linear linkage specificity	NM_138348	0.74	1.31E-10	1.17	1.74E-11	**1.38**	**8.75E-13**
DEDD2	death effector domain containing 2	NM_001270614	1.55	5.06E-13	2.16	6.66E-13	**2.12**	**9.04E-13**
STX11	syntaxin 11	NM_003764	0.72	3.44E-10	1.22	1.30E-11	**1.40**	**9.44E-13**
CYLD	cylindromatosis (turban tumor syndrome)	NM_001042355	0.60	2.94E-10	1.03	1.01E-11	**1.16**	**9.51E-13**
STK38L	serine/threonine kinase 38 like	NM_015000	1.44	4.06E-17	1.67	2.05E-15	**1.22**	**9.69E-13**
HSPA1B	heat shock 70kDa protein 1B	NM_005346	1.33	1.55E-17	1.29	3.04E-14	**1.07**	**1.06E-12**
FAM46A	family with sequence similarity 46. member A	ENST00000369754	1.76	3.05E-15	1.82	1.37E-12	**1.84**	**1.14E-12**
DNAJB6	DnaJ (Hsp40) homolog. subfamily B. member 6	NM_005494	0.77	1.56E-11	1.24	1.26E-12	**1.24**	**1.22E-12**
TRIM26	tripartite motif containing 26	NM_001242783	0.97	6.11E-14	1.04	1.11E-11	**1.17**	**1.41E-12**
IKZF5	IKAROS family zinc finger 5 (Pegasus)	NM_001271840	0.85	1.63E-13	0.93	1.96E-11	**1.07**	**1.52E-12**

GPBP1	GC-rich promoter binding protein 1	NM_001203246	0.53	1.73E-09	0.94	2.41E-11	**1.08**	**1.79E-12**
CLIC2	chloride intracellular channel 2	NM_001289	1.10	1.05E-10	1.70	2.13E-11	**1.93**	**1.82E-12**
TRIM26	tripartite motif containing 26	NM_001242783	1.00	9.55E-14	1.09	1.37E-11	**1.21**	**1.90E-12**
TRIM26	tripartite motif containing 26	NM_001242783	0.96	9.89E-14	1.04	1.67E-11	**1.16**	**2.20E-12**
CACYBP	calcyclin binding protein	NM_001007214	0.89	5.80E-13	1.14	4.23E-12	**1.18**	**2.27E-12**
UBR2	ubiquitin protein ligase E3 component n-recognin 2	NM_015255	0.47	3.60E-10	0.67	3.37E-10	**0.87**	**2.29E-12**
TRIB3	tribbles pseudokinase 3	uc002wdn.3	0.83	1.68E-08	1.68	3.13E-11	**1.92**	**2.60E-12**
FGF5	fibroblast growth factor 5	NM_004464	0.85	8.97E-12	0.69	1.69E-07	**0.84**	**5.74E-09**
ELL2	elongation factor. RNA polymerase II. 2	NM_012081	0.83	3.86E-14	1.27	7.84E-15	**0.94**	**2.71E-12**
PDE4DIP	phosphodiesterase 4D interacting protein	NM_001198832	0.85	1.25E-10	1.48	2.57E-12	**1.47**	**2.87E-12**
TRIM26	tripartite motif containing 26	NM_001242783	0.99	2.46E-13	1.13	1.42E-11	**1.23**	**2.88E-12**
DNAJA1	DnaJ (Hsp40) homolog. subfamily A. member 1	NM_001539	0.82	2.23E-13	0.98	5.07E-12	**1.01**	**2.92E-12**
JMJD6	jumonji domain containing 6	NM_001081461	0.59	2.88E-09	0.78	8.85E-09	**1.21**	**3.00E-12**
HSPB8	heat shock 22kDa protein 8	NM_014365	1.26	1.30E-11	0.97	4.48E-07	**1.35**	**1.80E-09**
RAB39A	RAB39A. member RAS oncogene family	NM_017516	1.21	3.78E-09	2.32	1.56E-11	**2.53**	**3.03E-12**
MICB	MHC class I polypeptide-related sequence B	NM_001289160	0.69	1.81E-11	1.16	6.80E-13	**1.06**	**3.71E-12**
MERTK	MER proto-oncogene. tyrosine kinase	NM_006343	0.49	5.20E-09	0.97	1.09E-11	**1.02**	**4.01E-12**
TRIM26	tripartite motif containing 26	NM_001242783	0.97	1.89E-13	1.05	3.46E-11	**1.17**	**4.09E-12**
TRIM26	tripartite motif containing 26	NM_001242783	0.97	1.89E-13	1.05	3.46E-11	**1.17**	**4.09E-12**
ABL2	ABL proto-oncogene 2. non-receptor tyrosine kinase	NM_001136000	0.66	2.19E-11	1.06	1.85E-12	**1.01**	**4.58E-12**
MICB	MHC class I polypeptide-related sequence B	NM_001289161	0.71	2.62E-11	1.13	2.94E-12	**1.11**	**4.58E-12**
ATP2C1	ATPase. Ca++ transporting. type 2C. member 1	NM_001001485	0.51	1.09E-09	0.76	4.95E-10	**0.98**	**4.60E-12**
NA	NA	RPTR-AF292560-1	-2.57	1.70E-14	-2.44	3.61E-11	**-2.69**	**5.69E-12**
MICB	MHC class I polypeptide-related sequence B	NM_005931	0.58	2.18E-10	1.01	4.82E-12	**1.01**	**5.75E-12**
MICB	MHC class I polypeptide-related sequence B	NM_005931	0.58	2.18E-10	1.01	4.82E-12	**1.01**	**5.75E-12**
NRIP3	nuclear receptor interacting protein 3	NM_020645	0.90	8.82E-11	1.32	4.46E-11	**1.47**	**5.96E-12**
MICB	MHC class I polypeptide-related sequence B	NM_005931	0.66	1.03E-10	1.06	9.07E-12	**1.08**	**6.13E-12**
NA	NA	RPTR-AY189981-1	-6.80	3.16E-16	-5.56	1.37E-11	**-5.79**	**6.41E-12**
C3orf38	chromosome 3 open reading frame 38	NM_173824	0.60	5.36E-10	1.00	2.46E-11	**1.07**	**7.44E-12**
PLOD2	procollagen-lysine. 2-oxoglutarate 5-dioxygenase 2	NM_000935	0.74	5.44E-13	0.95	2.84E-12	**0.91**	**7.45E-12**
STXBP5-AS1	STXBP5 antisense RNA 1	ENST00000433308	0.71	6.13E-08	2.01	2.22E-13	**1.66**	**8.67E-12**
RYBP	RING1 and YY1 binding protein	NM_012234	0.88	2.39E-11	1.27	1.61E-11	**1.31**	**9.43E-12**
STC1	stanniocalcin 1	NM_003155	-0.56	6.17E-10	-0.94	2.78E-11	**-1.00**	**9.56E-12**
ANXA1	annexin A1	NM_000700	0.43	5.67E-09	0.89	6.71E-12	**0.87**	**9.62E-12**
LOC344887	NmrA-like family domain containing 1 pseudogene	NR_033752	0.83	9.97E-13	1.07	6.33E-12	**1.04**	**1.02E-11**
TRIM26	tripartite motif containing 26	ENST00000436219	0.99	1.30E-13	1.07	2.13E-11	**1.11**	**1.07E-11**

ZCCHC6	zinc finger. CCHC domain containing 6	NM_001185059	0.57	3.17E-10	0.91	3.44E-11	**0.97**	**1.14E-11**
SNAI1	snail family zinc finger 1	NM_005985	0.79	3.35E-09	1.84	3.73E-13	**1.54**	**1.20E-11**
RASSF8	Ras association (RalGDS/AF-6) domain family (N-terminal) member 8	NM_001164746	0.56	1.69E-08	0.9	1.98E-09	**1.19**	**1.22E-11**
UHRF1BP1L	UHRF1 binding protein 1-like	NM_001006947	1.07	4.84E-13	1.37	3.60E-12	**1.28**	**1.26E-11**
ZFAND2A	zinc finger. AN1-type domain 2A	NM_182491	1.99	2.08E-19	1.84	1.12E-15	**1.13**	**1.36E-11**
HSPA6	heat shock 70kDa protein 6 (HSP70B)	NM_002155	1.95	1.18E-13	2.17	1.16E-11	**2.15**	**1.38E-11**
TRIM26	tripartite motif containing 26	ENST00000415923	0.99	3.56E-13	1.11	2.71E-11	**1.15**	**1.38E-11**
SLFN5	schlafen family member 5	NM_144975	0.76	3.70E-11	1.27	1.48E-12	**1.12**	**1.58E-11**
MICB	MHC class I polypeptide-related sequence B	NM_005931	0.62	2.78E-10	1.05	9.28E-12	**1.02**	**1.62E-11**
MED13	mediator complex subunit 13	NM_005121	0.51	8.74E-11	0.86	3.30E-12	**0.78**	**1.82E-11**
PDE4DIP	phosphodiesterase 4D interacting protein	NM_001002811	0.63	5.31E-10	0.91	3.77E-10	**1.07**	**1.92E-11**
HSPA9	heat shock 70kDa protein 9 (mortalin)	NM_004134	0.69	4.25E-12	0.88	3.08E-11	**0.90**	**1.94E-11**
SNAP23	synaptosomal-associated protein. 23kDa	NM_003825	0.41	5.26E-09	0.71	1.90E-10	**0.80**	**2.06E-11**
SLC5A3	solute carrier family 5 (sodium/myo-inositol cotransporter). member 3	NM_006933	0.60	1.67E-08	0.93	3.07E-09	**1.23**	**2.16E-11**
PDE4DIP	phosphodiesterase 4D interacting protein	NM_001198832	0.99	6.83E-10	1.73	1.42E-11	**1.68**	**2.38E-11**
UBR1	ubiquitin protein ligase E3 component n-recognin 1	NM_174916	0.57	2.62E-11	0.70	3.09E-10	**0.80**	**2.39E-11**
SLC3A2	solute carrier family 3 (amino acid transporter heavy chain). member 2	NM_001012662	0.52	5.39E-08	1.18	1.39E-11	**1.14**	**2.52E-11**
ALAS1	aminolevulinate. delta-. synthase 1	NM_000688	0.57	6.15E-11	0.55	4.82E-08	**0.85**	**2.56E-11**
HSP90AA1	heat shock protein 90kDa alpha (cytosolic). class A member 1	NM_001017963	0.89	4.90E-14	0.91	2.19E-11	**0.90**	**2.93E-11**
RNU6-71P	RNA. U6 small nuclear 71. pseudogene	NR_046940	2.11	7.23E-11	3.37	7.85E-12	**3.14**	**2.98E-11**
AZIN1	antizyme inhibitor 1	NM_015878	0.72	3.87E-11	0.93	1.99E-10	**1.01**	**3.99E-11**
DDIT3	DNA-damage-inducible transcript 3	NM_001195053	1.92	1.77E-14	2.1	2.74E-12	**1.82**	**4.06E-11**
PIGA	phosphatidylinositol glycan anchor biosynthesis. class A	NM_002641	0.80	4.52E-12	1.12	5.41E-12	**1.00**	**4.27E-11**
BRD2	bromodomain containing 2	NM_001113182	0.49	1.16E-10	0.65	4.13E-10	**0.73**	**4.70E-11**
P4HA1	prolyl 4-hydroxylase. alpha polypeptide I	NM_000917	0.67	1.12E-10	0.58	4.53E-07	**0.66**	**5.67E-08**
YRDC	yrdC N(6)-threonylcarbamoyltransferase domain containing	NM_024640	0.67	6.52E-12	0.83	8.03E-11	**0.85**	**4.79E-11**
HYOU1	hypoxia up-regulated 1	NM_001130991	0.77	8.18E-14	0.98	6.17E-13	**0.77**	**4.97E-11**
BRD2	bromodomain containing 2	NM_001113182	0.5	1.19E-10	0.66	3.74E-10	**0.73**	**5.85E-11**
BRD2	bromodomain containing 2	NM_001113182	0.52	1.30E-10	0.67	5.27E-10	**0.76**	**6.14E-11**
RNU6-329P	RNA. U6 small nuclear 329. pseudogene	ENST00000459618	1.59	3.51E-09	2.2	5.18E-09	**2.82**	**6.16E-11**
HSPA4	heat shock 70kDa protein 4	NM_002154	0.78	2.06E-13	0.97	2.32E-12	**0.81**	**6.54E-11**
HSPA5	heat shock 70kDa protein 5 (glucose-regulated protein. 78kDa)	NM_005347	0.88	7.41E-16	1.06	2.04E-14	**0.69**	**6.60E-11**

NA	NA	RPTR-AJ510163-3	-6.55	3.02E-15	-5.20	1.84E-10	**-5.48**	**7.04E-11**
MICB	MHC class I polypeptide-related sequence B	ENST00000458032	0.57	2.57E-09	0.95	1.20E-10	**0.98**	**7.09E-11**
PTGES3	prostaglandin E synthase 3 (cytosolic)	NM_001282601	0.54	3.61E-10	0.72	9.65E-10	**0.83**	**7.31E-11**
ULBP3	UL16 binding protein 3	NM_024518	0.87	4.44E-08	1.31	1.55E-08	**1.78**	**7.47E-11**
ME1	malic enzyme 1. NADP(+)-dependent. cytosolic	NM_002395	0.68	7.04E-13	0.90	2.39E-12	**0.75**	**7.84E-11**
RND3	Rho family GTPase 3	NM_001254738	0.55	5.37E-10	0.84	1.55E-10	**0.87**	**8.40E-11**
BRD2	bromodomain containing 2	NM_001291986	0.52	7.94E-11	0.69	2.54E-10	**0.73**	**8.78E-11**
NA	NA	NONHSAT098813	1.72	4.11E-14	1.48	4.62E-10	**1.63**	**8.88E-11**
MLLT11	myeloid/lymphoid or mixed-lineage leukemia (trithorax homolog. Drosophila); translocated to. 11	NM_006818	0.57	4.04E-12	0.69	7.50E-11	**0.68**	**9.47E-11**
SORBS1	sorbin and SH3 domain containing 1	NM_001034954	0.93	1.15E-08	1.67	1.57E-10	**1.72**	**9.56E-11**
OTUD7B	OTU deubiquitinase 7B	NM_020205	0.68	1.75E-10	0.72	2.46E-08	**0.99**	**9.58E-11**
DYRK3	dual-specificity tyrosine-(Y)-phosphorylation regulated kinase 3	ENST00000367106	0.53	6.04E-09	0.92	1.92E-10	**0.95**	**9.77E-11**
AZI2	5-azacytidine induced 2	NM_001271650	0.76	2.10E-10	0.66	6.53E-07	**0.85**	**1.09E-08**
NA	NA	TCONS_l2_00014930-XLOC_l2_008285	0.80	8.67E-12	1.05	3.47E-11	**0.99**	**1.03E-10**
NA	NA	---	0.94	9.50E-11	1.19	6.98E-10	**1.32**	**1.05E-10**
KCTD12	potassium channel tetramerization domain containing 12	NM_138444	-0.64	2.55E-09	-1.25	7.53E-12	**-1.08**	**1.10E-10**
VEGFC	vascular endothelial growth factor C	NM_005429	0.74	8.06E-11	0.89	1.45E-09	**1.03**	**1.22E-10**
CABLES2	Cdk5 and Abl enzyme substrate 2	NM_031215	0.70	1.24E-09	1.07	2.92E-10	**1.13**	**1.22E-10**
TSC22D2	TSC22 domain family. member 2	ENST00000361875	0.79	1.50E-10	1.17	6.80E-11	**1.13**	**1.24E-10**
GTPBP2	GTP binding protein 2	NM_001286216	0.56	7.47E-08	0.93	5.46E-09	**1.15**	**1.28E-10**
CNST	consortin. connexin sorting protein	NM_152609	0.73	1.42E-09	1.15	1.82E-10	**1.17**	**1.32E-10**
TMEM47	transmembrane protein 47	NM_031442	0.47	1.58E-09	0.80	4.99E-11	**0.75**	**1.45E-10**
EIF5	eukaryotic translation initiation factor 5	NM_001969	0.65	3.68E-11	0.75	1.44E-09	**0.85**	**1.45E-10**
SPAG9	sperm associated antigen 9	NM_001130527	0.67	1.00E-10	1.03	1.86E-11	**0.92**	**1.52E-10**
SERPINH1	serpin peptidase inhibitor. clade H (heat shock protein 47). member 1. (collagen binding protein 1)	NM_001207014	0.56	1.55E-11	0.71	1.18E-10	**0.70**	**1.58E-10**
STIP1	stress-induced phosphoprotein 1	NM_001282652	0.70	1.20E-11	0.69	6.79E-09	**0.86**	**1.66E-10**
KDM2A	lysine (K)-specific demethylase 2A	NM_001256405	0.68	7.26E-11	0.99	5.07E-11	**0.92**	**1.72E-10**
PPME1	protein phosphatase methylesterase 1	ENST00000535205	0.79	1.18E-11	0.95	2.14E-10	**0.96**	**1.83E-10**
ATF3	activating transcription factor 3	NM_001030287	2.75	1.94E-12	3.39	2.52E-11	**3.03**	**1.95E-10**
SLC39A14	solute carrier family 39 (zinc transporter). member 14	NM_001128431	1.06	1.21E-12	1.35	8.98E-12	**1.14**	**2.10E-10**
HSPB1	heat shock 27kDa protein 1	NM_001540	0.51	2.21E-10	0.76	8.35E-11	**0.72**	**2.21E-10**
ADAMTS18	ADAM metallopeptidase with thrombospondin type 1 motif. 18	NM_199355	-0.58	1.05E-08	-1.22	9.46E-12	**-1.03**	**2.27E-10**
HERC4	HECT and RLD domain containing E3 ubiquitin protein ligase 4	NM_001278185	0.45	7.73E-08	0.64	6.49E-08	**0.89**	**2.29E-10**

DNAJC24	DnaJ (Hsp40) homolog. subfamily C. member 24	ENST00000526042	0.57	4.92E-08	0.98	2.06E-09	**1.10**	**2.63E-10**
IGHJ1	immunoglobulin heavy joining 1	ENST00000390565	-1.44	3.45E-09	-1.76	3.67E-08	**-2.34**	**2.79E-10**
FAM219A	family with sequence similarity 219. member A	NM_001184940	0.69	5.19E-09	1.14	3.65E-10	**1.14**	**3.54E-10**
CSRNP1	cysteine-serine-rich nuclear protein 1	NM_033027	0.76	5.94E-09	1.30	1.91E-10	**1.25**	**3.90E-10**
SAMD4B	sterile alpha motif domain containing 4B	NM_018028	0.64	1.15E-09	1.06	6.32E-11	**0.95**	**4.22E-10**
SIK1	salt-inducible kinase 1	NM_173354	0.98	1.04E-11	1.60	6.61E-13	**1.12**	**4.44E-10**
CCDC174	coiled-coil domain containing 174	NM_016474	0.74	4.23E-11	0.94	3.30E-10	**0.92**	**4.67E-10**
TYW3	tRNA-yW synthesizing protein 3 homolog (S. cerevisiae)	NM_001162916	0.62	3.52E-08	0.98	4.88E-09	**1.12**	**4.87E-10**
HSPB1	heat shock 27kDa protein 1	NM_001540	0.48	1.82E-10	0.61	1.34E-09	**0.64**	**5.91E-10**
SPEN	spen family transcriptional repressor	NM_015001	0.70	1.27E-10	1.04	5.18E-11	**0.91**	**6.34E-10**
NA	NA	---	0.86	2.48E-08	1.17	4.42E-08	**1.51**	**6.42E-10**
MICA	MHC class I polypeptide-related sequence A	ENST00000415525	0.53	2.47E-09	0.83	3.46E-10	**0.80**	**7.01E-10**
KIAA0513	16q24.1	NM_001286565	1.60	5.92E-13	1.82	3.85E-11	**1.55**	**7.15E-10**
RNA5SP422	RNA. 5S ribosomal pseudogene 422	ENST00000516778	-1.23	9.59E-10	-1.03	3.97E-06	**-1.79**	**5.64E-10**
FEM1B	fem-1 homolog b (C. elegans)	NM_015322	0.63	2.29E-10	0.91	1.85E-10	**0.84**	**7.38E-10**
TP53BP2	tumor protein p53 binding protein 2	NM_001031685	0.75	1.08E-10	1.02	2.25E-10	**0.95**	**8.61E-10**
KCTD5	potassium channel tetramerization domain containing 5	NM_018992	0.57	7.59E-11	0.62	7.52E-09	**0.70**	**9.37E-10**
RPS6KA3	ribosomal protein S6 kinase. 90kDa. polypeptide 3	NM_004586	0.54	6.10E-10	0.72	1.65E-09	**0.73**	**1.08E-09**
LURAP1L	leucine rich adaptor protein 1-like	NM_203403	0.65	3.81E-08	0.95	2.48E-08	**1.14**	**1.12E-09**
HSP90AB3P	heat shock protein 90kDa alpha (cytosolic). class B member 3. pseudogene	ENST00000505987	0.66	1.30E-09	0.65	4.67E-07	**0.80**	**1.61E-08**
CCDC59	coiled-coil domain containing 59	NM_014167	0.50	2.17E-09	0.86	7.63E-11	**0.74**	**1.16E-09**
CPA4	carboxypeptidase A4	NM_001163446	-0.65	4.33E-09	-1.23	2.31E-11	**-0.99**	**1.22E-09**
MORC4	MORC family CW-type zinc finger 4	NM_001085354	0.66	1.87E-10	0.75	8.07E-09	**0.84**	**1.24E-09**
PSMC4	proteasome (prosome. macropain) 26S subunit. ATPase. 4	NM_006503	0.74	3.40E-12	0.92	3.74E-11	**0.76**	**1.27E-09**
IPPK	inositol 1.3.4.5.6-pentakisphosphate 2-kinase	NM_022755	0.67	1.97E-10	0.96	1.74E-10	**0.85**	**1.32E-09**
EDA2R	ectodysplasin A2 receptor	NM_001199687	0.69	3.95E-09	1.18	1.26E-10	**1.03**	**1.42E-09**
FXR1	fragile X mental retardation. autosomal homolog 1	NM_001013438	0.50	1.55E-09	0.69	2.19E-09	**0.70**	**1.43E-09**
IFRD1	interferon-related developmental regulator 1	NM_001007245	0.64	7.77E-09	0.96	2.49E-09	**0.99**	**1.50E-09**
ERRFI1	ERBB receptor feedback inhibitor 1	ENST00000467067	0.38	8.01E-08	1.01	1.11E-12	**0.68**	**1.66E-09**
C18orf25	chromosome 18 open reading frame 25	NM_001008239	0.84	1.70E-11	0.99	5.25E-10	**0.92**	**1.86E-09**
SNX3	sorting nexin 3	NM_003795	0.52	2.50E-09	0.54	3.59E-07	**0.64**	**2.55E-08**
BIRC2	baculoviral IAP repeat containing 2	NM_001166	0.61	1.55E-10	0.92	4.97E-11	**0.75**	**1.86E-09**
NA	NA	---	0.65	1.61E-08	1.05	1.85E-09	**1.04**	**2.02E-09**
BRD2	bromodomain containing 2	NM_001113182	0.52	2.81E-09	0.57	1.58E-07	**0.68**	**9.86E-09**

LARP4	La ribonucleoprotein domain family. member 4	NM_001170803	0.71	2.20E-10	0.81	9.24E-09	**0.88**	**2.05E-09**
RP4-791M13.3	novel transcript	OTTHUMT00000384603	0.81	1.55E-08	1.35	8.35E-10	**1.28**	**2.06E-09**
HSPB1P1	heat shock 27kDa protein 1 pseudogene 1	ENST00000423240	0.37	3.96E-09	0.53	3.79E-09	**0.54**	**2.28E-09**
SLC37A1	solute carrier family 37 (glucose-6-phosphate transporter). member 1	NM_018964	0.50	4.10E-08	0.71	3.51E-08	**0.83**	**2.29E-09**
BRPF3	bromodomain and PHD finger containing. 3	NM_015695	0.68	4.66E-09	0.97	3.69E-09	**1.00**	**2.33E-09**
TBC1D4	TBC1 domain family. member 4	NM_001286658	0.72	7.90E-10	0.95	2.90E-09	**0.96**	**2.34E-09**
IPMK	inositol polyphosphate multikinase	NM_152230	0.6	5.30E-08	1.06	1.19E-09	**1.01**	**2.41E-09**
MICA	MHC class I polypeptide-related sequence A	ENST00000400325	0.53	2.16E-08	0.92	7.06E-10	**0.85**	**2.47E-09**
MICA	MHC class I polypeptide-related sequence A	ENST00000400325	0.53	2.16E-08	0.92	7.06E-10	**0.85**	**2.47E-09**
ZNF317	zinc finger protein 317	NM_001190791	0.54	1.28E-08	0.73	3.35E-08	**0.84**	**2.60E-09**
TAF7	TAF7 RNA polymerase II. TATA box binding protein (TBP)-associated factor. 55kDa	NM_005642	0.49	4.30E-09	0.45	3.09E-06	**0.75**	**9.48E-10**
ATG5	autophagy related 5	NM_001286106	0.5	1.70E-08	0.78	3.03E-09	**0.78**	**2.85E-09**
PLAA	phospholipase A2-activating protein	NM_001031689	0.8	1.65E-14	0.73	6.74E-11	**0.59**	**2.91E-09**
ACBD3	acyl-CoA binding domain containing 3	NM_022735	0.75	6.99E-11	0.88	1.93E-09	**0.86**	**2.97E-09**
ELL	elongation factor RNA polymerase II	NM_006532	0.66	5.99E-08	1.09	4.43E-09	**1.11**	**2.99E-09**
SLC7A2	solute carrier family 7 (cationic amino acid transporter. y+ system). member 2	NM_001008539	0.76	4.21E-10	1.37	5.33E-12	**0.96**	**3.31E-09**
GORAB	golgin. RAB6-interacting	NM_001146039	0.62	5.24E-09	0.65	6.23E-07	**0.84**	**1.08E-08**
RNU6-531P	RNA. U6 small nuclear 531. pseudogene	ENST00000516694	1.71	1.32E-11	1.97	5.54E-10	**1.77**	**3.42E-09**
ZNF484	zinc finger protein 484	NM_001007101	0.71	5.45E-09	0.82	1.70E-07	**0.86**	**7.89E-08**
TAF2	TAF2 RNA polymerase II. TATA box binding protein (TBP)-associated factor. 150kDa	NM_003184	0.45	5.50E-09	0.53	1.13E-07	**0.57**	**4.01E-08**
CRY1	cryptochrome circadian clock 1	NM_004075	1.06	2.39E-13	1.15	3.83E-11	**0.89**	**3.66E-09**
P4HA2	prolyl 4-hydroxylase. alpha polypeptide II	ENST00000401867	0.62	1.59E-10	0.74	3.34E-09	**0.74**	**3.77E-09**
FNIP1	folliculin interacting protein 1	NM_001008738	0.55	8.39E-10	0.70	5.64E-09	**0.71**	**4.25E-09**
ALDH2	aldehyde dehydrogenase 2 family (mitochondrial)	NM_000690	0.48	8.60E-09	0.73	2.33E-09	**0.70**	**4.42E-09**
KCMF1	potassium channel modulatory factor 1	NM_020122	0.65	8.39E-12	0.76	2.34E-10	**0.64**	**5.10E-09**
HSPA13	heat shock protein 70kDa family. member 13	NM_006948	0.66	7.78E-11	0.70	1.22E-08	**0.73**	**5.53E-09**
SLC25A33	solute carrier family 25 (pyrimidine nucleotide carrier). member 33	NM_032315	0.63	6.83E-09	0.69	4.27E-07	**0.80**	**4.03E-08**
SDE2	SDE2 telomere maintenance homolog (S. pombe)	NM_152608	1.17	1.99E-14	0.81	9.37E-09	**0.84**	**5.71E-09**
LRIG1	leucine-rich repeats and immunoglobulin-like domains 1	ENST00000273261	0.41	7.26E-08	0.70	2.99E-09	**0.67**	**6.26E-09**
FNDC3A	fibronectin type III domain containing 3A	NM_001079673	0.7	4.89E-13	0.94	1.22E-12	**0.59**	**6.48E-09**

UBC	ubiquitin C	ENST00000536769	1.04	7.82E-12	1.27	1.30E-10	1.01	6.97E-09
OSER1	oxidative stress responsive serine-rich 1	NM_016470	0.79	1.07E-10	1.00	9.46E-10	0.89	7.10E-09
CEBPB	CCAAT/enhancer binding protein (C/EBP). beta	NM_001285878	0.68	8.96E-09	1.40	1.21E-11	0.97	8.37E-09
ZNF267	zinc finger protein 267	NM_001265588	0.64	1.78E-08	0.86	4.61E-08	0.95	8.52E-09
UBR3	ubiquitin protein ligase E3 component n-recognin 3 (putative)	ENST00000272793	0.77	3.55E-13	0.74	4.82E-10	0.62	9.23E-09
PSMD14	proteasome (prosome. macropain) 26S subunit. non-ATPase. 14	NM_005805	0.72	9.29E-12	0.83	4.72E-10	0.70	9.45E-09
CPT1A	carnitine palmitoyltransferase 1A (liver)	NM_001031847	-0.49	9.69E-09	-0.78	1.14E-09	-0.69	9.64E-09
TULP3	tubby like protein 3	NM_001160408	0.40	2.08E-08	0.78	7.17E-11	0.59	1.02E-08
SLC19A2	solute carrier family 19 (thiamine transporter). member 2	NM_006996	1.30	7.50E-17	1.32	5.41E-14	0.68	1.07E-08
ZBTB21	zinc finger and BTB domain containing 21	NM_001098402	0.79	3.33E-10	0.85	3.61E-08	0.91	1.14E-08
42434	membrane-associated ring finger (C3HC4) 5	ENST00000467521	0.43	1.45E-08	0.62	9.46E-09	0.62	1.17E-08
FAM124B	family with sequence similarity 124B	NM_024785	-0.68	4.29E-10	-0.98	3.16E-10	-0.79	1.22E-08
SETD5	SET domain containing 5	NM_001080517	0.38	5.54E-08	0.76	1.16E-10	0.58	1.26E-08
CCT4	chaperonin containing TCP1. subunit 4 (delta)	NM_001256721	0.46	1.24E-09	0.59	6.28E-09	0.57	1.27E-08
PI4K2B	phosphatidylinositol 4-kinase type 2 beta	NM_018323	0.68	1.47E-09	0.77	6.20E-08	0.85	1.28E-08
EPSTI1	epithelial stromal interaction 1 (breast)	NM_001002264	1.52	1.10E-13	1.53	6.47E-11	1.13	1.43E-08
PDE4DIP	phosphodiesterase 4D interacting protein	NM_001198834	0.86	6.52E-09	1.18	1.10E-08	1.17	1.43E-08
AHNAK2	AHNAK nucleoprotein 2	NM_138420	-0.55	1.61E-10	-0.89	1.44E-11	-0.60	1.62E-08
MAFG	v-maf avian musculoaponeurotic fibrosarcoma oncogene homolog G	NM_032711	0.59	1.86E-09	0.77	6.59E-09	0.73	1.69E-08
ZUFSP	zinc finger with UFM1-specific peptidase domain	NM_145062	0.62	1.89E-08	0.67	1.44E-06	0.91	1.11E-08
RLF	rearranged L-myc fusion	NM_012421	0.70	1.95E-08	0.77	9.43E-07	1.14	1.83E-09
BIRC3	baculoviral IAP repeat containing 3	ENST00000263464	1.30	1.99E-08	1.45	8.10E-07	1.93	8.19E-09
ARID5A	AT rich interactive domain 5A (MRF1-like)	NM_212481	1.01	5.46E-13	1.01	3.59E-10	0.80	1.93E-08
GFPT1	glutamine--fructose-6-phosphate transaminase 1	NM_001244710	0.66	1.35E-11	0.72	1.65E-09	0.62	2.24E-08
N4BP1	NEDD4 binding protein 1	NM_153029	0.59	1.14E-09	0.73	1.37E-08	0.7	2.29E-08
SNIP1	Smad nuclear interacting protein 1	ENST00000468040	0.5	2.35E-08	0.57	6.34E-07	0.71	1.88E-08
KANSL1L	KAT8 regulatory NSL complex subunit 1-like	NM_152519	0.61	3.32E-08	1.21	1.08E-10	0.89	2.31E-08
NPLOC4	nuclear protein localization 4 homolog (S. cerevisiae)	NM_017921	0.55	1.03E-10	0.73	3.90E-10	0.57	2.55E-08
C9orf72	chromosome 9 open reading frame 72	NM_001256054	0.78	2.66E-08	1.03	9.34E-08	1.23	4.87E-09
SERTAD1	SERTA domain containing 1	NM_013376	0.59	2.88E-08	0.66	1.16E-06	1.05	5.56E-10
TMEM39A	transmembrane protein 39A	NM_018266	0.58	9.47E-11	0.66	4.26E-09	0.59	2.70E-08
PLD1	phospholipase D1. phosphatidylcholine-specific	NM_001130081	-0.54	3.33E-08	-0.54	5.90E-06	-0.77	3.61E-08
SSH1	slingshot protein phosphatase 1	NM_001161330	0.8	3.43E-11	1.25	5.08E-12	0.77	2.78E-08

LINC01252	long intergenic non-protein coding RNA 1252	NR_033890	0.66	3.67E-08	0.86	1.35E-07	**0.96**	**1.95E-08**
NFKB1	nuclear factor of kappa light polypeptide gene enhancer in B-cells 1	NM_001165412	0.69	8.57E-10	0.99	6.40E-10	**0.79**	**3.02E-08**
NRBF2	nuclear receptor binding factor 2	NM_001282405	0.51	5.35E-08	0.75	2.85E-08	**0.74**	**3.56E-08**
RP11-153M3.1	60 kDa heat shock protein pseudogene	OTTHUMT00000409621	0.86	6.49E-10	1.19	1.09E-09	**0.97**	**3.60E-08**
SRXN1	sulfiredoxin 1	NM_080725	0.85	1.49E-11	0.75	5.85E-08	**0.77**	**3.73E-08**
NFIB	nuclear factor I/B	NM_001190737	-0.42	9.82E-09	-0.55	3.14E-08	**-0.55**	**3.90E-08**
TCP1	t-complex 1	NM_001008897	0.45	4.68E-08	0.56	2.67E-07	**0.61**	**7.84E-08**
EFCAB7	EF-hand calcium binding domain 7	NM_032437	0.84	1.29E-08	1.15	2.06E-08	**1.11**	**3.90E-08**
GULP1	GULP. engulfment adaptor PTB domain containing 1	NM_001252668	0.62	9.21E-09	1.03	5.61E-10	**0.8**	**3.99E-08**
KDM6B	lysine (K)-specific demethylase 6B	NM_001080424	0.82	2.42E-11	0.95	8.23E-10	**0.76**	**4.12E-08**
TNFSF18	tumor necrosis factor (ligand) superfamily. member 18	NM_005092	-1.03	2.38E-12	-0.83	5.96E-08	**-0.84**	**4.37E-08**
F3	coagulation factor III (thromboplastin. tissue factor)	NM_001178096	1.01	2.35E-11	0.92	4.49E-08	**0.92**	**4.56E-08**
CNKSR3	CNKSR family member 3	NM_173515	0.86	6.32E-10	0.96	3.24E-08	**0.94**	**4.66E-08**
GBP3	guanylate binding protein 3	NM_018284	0.63	2.12E-09	0.72	6.85E-08	**0.74**	**4.94E-08**
CTH	cystathionine gamma-lyase	ENST00000346806	1.01	7.56E-09	1.52	2.68E-09	**1.27**	**5.98E-08**
SDPR	serum deprivation response	NM_004657	-0.61	6.68E-09	-1.02	3.37E-10	**-0.75**	**6.42E-08**
ABCB1	ATP-binding cassette. sub-family B (MDR/TAP). member 1	NM_000927	0.45	4.40E-08	0.69	1.23E-08	**0.62**	**7.20E-08**
TXNIP	thioredoxin interacting protein	NM_006472	-0.78	7.33E-11	-0.96	8.01E-10	**-0.73**	**7.72E-08**
YTHDC1	YTH domain containing 1	NM_001031732	0.40	8.16E-08	0.82	1.28E-10	**0.86**	**5.10E-11**
HELB	helicase (DNA) B	NM_033647	0.78	8.51E-08	0.83	5.82E-06	**1.17**	**2.89E-08**
HSPA7	heat shock 70kDa protein 7 (HSP70B)	NR_024151	0.97	9.27E-08	2.24	1.71E-11	**3.05**	**4.66E-14**
YKT6	YKT6 v-SNARE homolog (S. cerevisiae)	NM_006555	0.47	9.60E-08	0.74	2.00E-08	**0.78**	**7.88E-09**
ALKBH1	alkB. alkylation repair homolog 1 (E. coli)	NM_006020	0.48	1.01E-07	0.65	1.82E-07	**0.69**	**7.81E-08**
USPL1	ubiquitin specific peptidase like 1	NM_005800	0.45	1.02E-07	0.71	1.57E-08	**1.21**	**1.01E-12**
ZNF773	zinc finger protein 773	NM_198542	0.72	1.13E-07	1.33	1.43E-09	**1.49**	**2.01E-10**
C11orf84	chromosome 11 open reading frame 84	XM_005273782	0.65	1.21E-07	1.18	1.83E-09	**1.14**	**3.03E-09**
STXBP5-AS1	STXBP5 antisense RNA 1	ENST00000606831	0.66	1.26E-07	1.38	1.79E-10	**1.87**	**5.85E-13**
NRBF2	nuclear receptor binding factor 2	NM_001282405	0.51	1.31E-07	0.78	4.30E-08	**0.83**	**1.46E-08**
ZSWIM6	zinc finger. SWIM-type containing 6	NM_020928	0.40	1.31E-07	1.01	6.04E-12	**1.00**	**7.90E-12**
INO80	INO80 complex subunit	NM_017553	0.39	1.31E-07	0.59	4.69E-08	**0.93**	**1.64E-11**
CAT	catalase	NM_001752	-0.38	1.37E-07	-0.57	5.33E-08	**-0.59**	**3.55E-08**
MYOZ2	myozenin 2	NM_016599	0.91	1.41E-07	1.77	6.13E-10	**2.01**	**6.69E-11**
RP11-272L13.3	novel transcript	OTTHUMT00000467510	0.62	1.43E-07	1.17	1.07E-09	**1.19**	**8.09E-10**
PPIL4	peptidylprolyl isomerase (cyclophilin)-like 4	NM_139126	0.44	1.46E-07	0.49	4.68E-06	**0.73**	**1.05E-08**
PDE4DIP	phosphodiesterase 4D interacting protein	AB042555	1.10	1.60E-07	1.73	3.25E-08	**2.27**	**2.78E-10**
SYNE1	spectrin repeat containing. nuclear envelope 1	NM_033071	0.38	1.64E-07	0.84	9.56E-11	**0.94**	**1.02E-11**

NFATC3	nuclear factor of activated T-cells. cytoplasmic. calcineurin-dependent 3	uc010vkn.2	0.45	1.65E-07	0.53	2.29E-06	**0.90**	**4.52E-10**
NFIL3	nuclear factor. interleukin 3 regulated	NM_005384	0.69	1.67E-07	1.17	8.72E-09	**1.07**	**4.12E-08**
TBC1D2B	TBC1 domain family. member 2B	NM_015079	-0.38	1.67E-07	-0.27	1.06E-03	**-0.63**	**1.63E-08**
EPHB4	EPH receptor B4	NM_004444	-0.41	1.68E-07	-0.73	4.42E-09	**-0.84**	**3.42E-10**
CWC25	CWC25 spliceosome-associated protein homolog (S. cerevisiae)	NM_017748	0.49	1.72E-07	0.5	1.51E-05	**0.74**	**5.19E-08**
ITGB3	integrin. beta 3 (platelet glycoprotein IIIa. antigen CD61)	NM_000212	-0.41	1.80E-07	-0.58	1.37E-07	**-0.73**	**3.69E-09**
MIR31HG	MIR31 host gene (non-protein coding)	NR_027054	0.57	1.98E-07	0.87	5.36E-08	**1.00**	**6.22E-09**
IL3RA	interleukin 3 receptor. alpha (low affinity)	NM_001267713	0.53	2.19E-07	0.78	1.35E-07	**1.21**	**6.88E-11**
TRAF1	TNF receptor-associated factor 1	NM_001190945	0.66	2.21E-07	1.41	2.50E-10	**1.02**	**6.45E-08**
ZRANB1	zinc finger. RAN-binding domain containing 1	NM_017580	0.38	2.35E-07	0.83	1.97E-10	**0.93**	**2.14E-11**
MCU	mitochondrial calcium uniporter	NM_001270679	0.39	2.36E-07	0.66	1.29E-08	**0.75**	**1.43E-09**
IL3RA	interleukin 3 receptor. alpha (low affinity)	NM_001267713	0.50	2.40E-07	0.80	3.67E-08	**1.10**	**1.63E-10**
ACSL4	acyl-CoA synthetase long-chain family member 4	NM_004458	0.35	2.43E-07	0.59	1.62E-08	**0.68**	**1.35E-09**
NA	NA	NONHSAT135562	-0.53	2.52E-07	-0.89	1.32E-08	**-0.83**	**4.97E-08**
NT5C2	5-nucleotidase. cytosolic II	NM_001134373	0.45	2.75E-07	0.85	3.14E-09	**0.83**	**4.72E-09**
TRAF3	TNF receptor-associated factor 3	NM_001199427	0.54	2.78E-07	0.83	8.65E-08	**1.26**	**6.28E-11**
VEGFA	vascular endothelial growth factor A	NM_001025366	0.59	2.89E-07	1.51	9.42E-12	**1.12**	**2.17E-09**
LOC100132167	uncharacterized LOC100132167	BC006438	0.41	3.22E-07	0.67	2.66E-08	**0.67**	**2.77E-08**
ICAM1	intercellular adhesion molecule 1	NM_000201	0.54	3.45E-07	1.07	1.43E-09	**1.21**	**1.47E-10**
SLC29A1	solute carrier family 29 (equilibrative nucleoside transporter). member 1	NM_001078175	-0.41	3.60E-07	-0.71	1.14E-08	**-0.68**	**2.33E-08**
MCL1	myeloid cell leukemia 1	NM_001197320	0.32	3.63E-07	0.50	7.58E-08	**0.65**	**1.03E-09**
RNF19B	ring finger protein 19B	NM_001127361	0.41	3.64E-07	0.70	1.63E-08	**0.67**	**3.14E-08**
YWHAG	tyrosine 3-monooxygenase/tryptophan 5-monooxygenase activation protein. gamma	NM_012479	0.38	4.09E-07	0.38	4.21E-05	**0.62**	**4.31E-08**
RNU6-1213P	RNA. U6 small nuclear 1213. pseudogene	ENST00000517075	1.04	4.22E-07	1.90	6.99E-09	**2.17**	**6.91E-10**
KPNA5	karyopherin alpha 5 (importin alpha 6)	NM_002269	0.52	4.26E-07	0.53	3.58E-05	**0.85**	**4.11E-08**
C3orf52	chromosome 3 open reading frame 52	NM_024616	0.45	4.36E-07	1.03	1.49E-10	**1.04**	**1.31E-10**
SPATS2L	spermatogenesis associated. serine-rich 2-like	NM_001100422	0.35	4.48E-07	0.35	5.24E-05	**0.57**	**4.49E-08**
TBPL1	TBP-like 1	NM_001253676	0.49	4.49E-07	0.66	1.10E-06	**0.87**	**1.22E-08**
SLBP	stem-loop binding protein	NM_006527	0.45	4.74E-07	0.81	9.49E-09	**1.06**	**7.07E-11**
ATP6V0A4	ATPase. H+ transporting. lysosomal V0 subunit a4	NM_020632	0.56	5.09E-07	0.85	1.62E-07	**0.92**	**4.78E-08**
SMURF1	SMAD specific E3 ubiquitin protein ligase 1	NM_001199847	0.43	5.28E-07	0.94	4.71E-10	**0.98**	**2.08E-10**
CEP350	centrosomal protein 350kDa	NM_014810	0.34	5.72E-07	0.58	2.75E-08	**0.78**	**1.66E-10**

CHIC2	cysteine-rich hydrophobic domain 2	NM_012110	0.44	6.35E-07	0.91	1.04E-09	**1.21**	**5.93E-12**
CLEC14A	C-type lectin domain family 14. member A	NM_175060	-0.41	6.37E-07	-0.98	1.21E-10	**-0.86**	**1.25E-09**
ZNF460	zinc finger protein 460	NM_006635	0.45	6.51E-07	0.70	1.27E-07	**0.88**	**2.95E-09**
GFM2	G elongation factor. mitochondrial 2	NM_001281302	0.43	6.57E-07	0.68	1.26E-07	**0.89**	**1.36E-09**
RP11-385D13.1	novel tripartite motif-containing 16 (TRIM16) and CMT1A duplicated region transcript 1 (CDRT1) protein	OTTHUMT00000363271	0.69	6.97E-07	1.74	4.45E-11	**1.92**	**6.74E-12**
RRAD	Ras-related associated with diabetes	NM_001128850	0.53	7.31E-07	0.77	4.45E-07	**1.55**	**2.32E-12**
KDM6A	lysine (K)-specific demethylase 6A	NM_001291415	0.40	7.39E-07	0.72	1.95E-08	**0.69**	**3.62E-08**
RP9P	retinitis pigmentosa 9 pseudogene	NR_003500	0.87	7.46E-07	1.66	6.31E-09	**1.86**	**7.97E-10**
CECR2	cat eye syndrome chromosome region. candidate 2	NM_001290046	0.63	7.53E-07	1.54	8.80E-11	**1.35**	**8.69E-10**
ZNF227	zinc finger protein 227	NM_001289166	0.47	7.59E-07	0.69	3.76E-07	**0.87**	**1.03E-08**
GPX3	glutathione peroxidase 3 (plasma)	NM_002084	0.55	8.20E-07	1.15	1.39E-09	**0.95**	**3.36E-08**
MMP10	matrix metallopeptidase 10 (stromelysin 2)	NM_002425	0.39	8.28E-07	1.23	7.39E-13	**1.42**	**4.47E-14**
C2orf44	chromosome 2 open reading frame 44	NM_001142319	0.43	8.68E-07	0.49	1.67E-05	**0.89**	**1.86E-09**
ZNF426	zinc finger protein 426	NM_024106	0.46	8.90E-07	0.65	8.99E-07	**0.86**	**1.04E-08**
FBXL12	F-box and leucine-rich repeat protein 12	NM_017703	0.43	9.06E-07	0.75	3.73E-08	**0.95**	**5.69E-10**
SH3RF1	SH3 domain containing ring finger 1	NM_020870	0.36	9.18E-07	0.64	2.98E-08	**0.60**	**7.18E-08**
STXBP5-AS1	STXBP5 antisense RNA 1	NR_034115	0.72	9.67E-07	1.93	2.16E-11	**2.52**	**1.37E-13**
ANKLE2	ankyrin repeat and LEM domain containing 2	NM_015114	0.32	1.06E-06	0.91	6.35E-12	**1.02**	**7.33E-13**
CLDN12	claudin 12	NM_001185072	0.46	1.08E-06	0.84	1.79E-08	**0.86**	**1.13E-08**
JUP	junction plakoglobin	NM_002230	-0.41	1.08E-06	-0.73	3.11E-08	**-0.87**	**1.61E-09**
NFKBIA	nuclear factor of kappa light polypeptide gene enhancer in B-cells inhibitor. alpha	NM_020529	0.37	1.17E-06	0.84	4.75E-10	**1.07**	**6.30E-12**
NFIA	nuclear factor I/A	NM_001134673	-0.39	1.17E-06	-0.74	1.03E-08	**-0.78**	**3.68E-09**
MIER3	mesoderm induction early response 1. family member 3	NM_152622	0.36	1.28E-06	0.48	2.73E-06	**0.70**	**6.94E-09**
SOCS6	suppressor of cytokine signaling 6	NM_004232	0.47	1.35E-06	0.86	2.64E-08	**1.00**	**1.85E-09**
TBC1D3B	TBC1 domain family. member 3B	NM_001001417	0.45	1.35E-06	0.6	3.03E-06	**0.83**	**2.01E-08**
JUNB	jun B proto-oncogene	NM_002229	0.53	1.36E-06	1.22	3.99E-10	**1.23**	**3.49E-10**
GCOM1	GRINL1A complex locus 1	NM_001018090	0.37	1.38E-06	0.71	1.19E-08	**0.74**	**6.41E-09**
RHBDD2	rhomboid domain containing 2	NM_001040456	0.56	1.38E-06	0.93	1.18E-07	**1.08**	**1.04E-08**
GJA5	gap junction protein. alpha 5. 40kDa	NM_005266	-0.67	1.40E-06	-0.93	1.81E-06	**-1.23**	**2.61E-08**
MALT1	mucosa associated lymphoid tissue lymphoma translocation gene 1	NM_006785	0.40	1.43E-06	0.47	1.83E-05	**0.87**	**1.24E-09**
NA	NA	---	0.72	1.45E-06	1.44	5.65E-09	**1.63**	**6.55E-10**
TSEN15	TSEN15 tRNA splicing endonuclease subunit	NR_023349	0.46	1.46E-06	1.02	8.94E-10	**1.21**	**3.93E-11**
TBC1D3B	TBC1 domain family. member 3B	NM_001001417	0.44	1.49E-06	0.73	1.34E-07	**0.93**	**2.38E-09**
CD24	CD24 molecule	BC064619	-0.8	1.57E-06	-1.56	1.17E-08	**-1.52**	**1.82E-08**

STXBP5-AS1	STXBP5 antisense RNA 1	NR_034115	0.62	1.61E-06	1.84	4.83E-12	**2.32**	**5.73E-14**
ZMYM5	zinc finger. MYM-type 5	NM_001039649	0.48	1.69E-06	0.65	3.52E-06	**0.86**	**4.58E-08**
ANKMY2	ankyrin repeat and MYND domain containing 2	NM_020319	0.44	1.89E-06	0.70	3.37E-07	**0.96**	**2.26E-09**
MRPL18	mitochondrial ribosomal protein L18	NM_014161	0.44	1.99E-06	0.68	6.27E-07	**1.12**	**1.57E-10**
ZNF521	zinc finger protein 521	NM_015461	-0.42	2.01E-06	-0.88	3.69E-09	**-0.74**	**6.71E-08**
PKI55	DKFZp434H1419	NR_037701	-0.38	2.07E-06	-0.51	3.49E-06	**-0.74**	**1.38E-08**
SPIRE1	spire-type actin nucleation factor 1	NM_001128626	0.37	2.14E-06	0.71	1.71E-08	**0.68**	**3.12E-08**
INPP5D	inositol polyphosphate-5-phosphatase. 145kDa	NM_001017915	-0.38	2.14E-06	-0.83	1.97E-09	**-0.81**	**2.93E-09**
TOM1L1	target of myb1 (chicken)-like 1	NM_005486	0.48	2.21E-06	0.37	1.96E-03	**0.98**	**6.58E-09**
SERPINB8	serpin peptidase inhibitor. clade B (ovalbumin). member 8	NM_002640	0.30	2.36E-06	0.50	2.50E-07	**0.58**	**2.27E-08**
BTN2A2	butyrophilin. subfamily 2. member A2	NM_001197237	0.56	2.37E-06	0.62	6.06E-05	**1.00**	**6.50E-08**
ZNF281	zinc finger protein 281	NM_001281293	0.42	2.47E-06	0.66	5.97E-07	**0.83**	**1.66E-08**
EIF1AD	eukaryotic translation initiation factor 1A domain containing	NM_001242481	0.37	2.68E-06	0.36	2.76E-04	**0.79**	**3.87E-09**
SUGT1	SGT1. suppressor of G2 allele of SKP1 (S. cerevisiae)	NM_001130912	0.43	2.74E-06	0.48	6.50E-05	**0.84**	**1.89E-08**
HIBCH	3-hydroxyisobutyryl-CoA hydrolase	NM_014362	0.42	2.86E-06	0.57	4.62E-06	**0.86**	**9.75E-09**
NA	NA	ENST00000391069	0.66	2.86E-06	0.96	2.16E-06	**1.20**	**6.99E-08**
NAV1	neuron navigator 1	NM_001167738	-0.29	2.89E-06	-0.45	5.66E-07	**-0.66**	**1.30E-09**
MORC3	MORC family CW-type zinc finger 3	NM_015358	0.45	3.07E-06	0.81	8.84E-08	**1.08**	**6.86E-10**
NPC1	Niemann-Pick disease. type C1	NM_000271	0.29	3.08E-06	0.46	6.17E-07	**0.55**	**3.18E-08**
AARS	alanyl-tRNA synthetase	NM_001605	0.34	3.19E-06	0.8	9.77E-10	**0.96**	**3.64E-11**
RHOBTB1	Rho-related BTB domain containing 1	XR_428729	-0.38	3.39E-06	-0.54	3.38E-06	**-0.71**	**5.96E-08**
RANGAP1	Ran GTPase activating protein 1	ENST00000455915	0.42	3.55E-06	0.93	3.43E-09	**1.01**	**8.48E-10**
GPR180	G protein-coupled receptor 180	NM_180989	0.37	3.61E-06	0.59	6.66E-07	**0.72**	**2.62E-08**
ACSL4	acyl-CoA synthetase long-chain family member 4	AK294197	0.51	3.98E-06	0.85	3.74E-07	**1.37**	**1.22E-10**
NA	NA	---	0.49	4.00E-06	0.57	5.17E-05	**1.06**	**5.99E-09**
PAPD5	PAP associated domain containing 5	NM_001040284	0.39	4.48E-06	1.04	1.55E-10	**1.55**	**8.70E-14**
ZNF562	zinc finger protein 562	NM_001130031	0.51	4.50E-06	0.79	1.15E-06	**1.22**	**9.85E-10**
ZBTB43	zinc finger and BTB domain containing 43	NM_014007	0.52	4.53E-06	0.89	2.86E-07	**1.12**	**7.60E-09**
ZNF134	zinc finger protein 134	NM_003435	0.36	4.82E-06	0.55	1.38E-06	**0.76**	**8.50E-09**
GABBR2	gamma-aminobutyric acid (GABA) B receptor. 2	NM_005458	-0.26	4.85E-06	-0.76	4.17E-11	**-0.88**	**2.62E-12**
PPP1R15B	protein phosphatase 1. regulatory subunit 15B	NM_032833	0.30	5.09E-06	0.96	7.06E-12	**0.95**	**9.28E-12**
RSRC2	arginine/serine-rich coiled-coil 2	NM_023012	0.36	5.29E-06	0.65	1.43E-07	**0.96**	**1.89E-10**
DDIT4	DNA-damage-inducible transcript 4	NM_019058	0.37	5.35E-06	1.08	3.99E-11	**0.85**	**2.67E-09**
CCDC9	coiled-coil domain containing 9	NM_015603	0.39	5.36E-06	0.51	1.60E-05	**1.08**	**1.16E-10**
MGAT5	mannosyl (alpha-1.6-)-glycoprotein beta-1.6-N-acetyl-glucosaminyltransferase	NM_002410	-0.29	5.74E-06	-0.76	3.11E-10	**-0.71**	**1.02E-09**
TRIB1	tribbles pseudokinase 1	NM_001282985	0.40	6.37E-06	1.69	3.31E-14	**2.19**	**2.13E-16**

ALOXE3	arachidonate lipoxygenase 3	NM_001165960	0.55	7.06E-06	1.84	4.35E-12	**2.02**	**7.04E-13**
TBC1D3H	TBC1 domain family. member 3H	NM_001123392	0.38	7.70E-06	0.74	6.62E-08	**0.83**	**8.97E-09**
DUSP8	dual specificity phosphatase 8	NM_004420	0.36	7.86E-06	1.32	7.15E-13	**1.42**	**1.87E-13**
FEM1C	fem-1 homolog c (C. elegans)	NM_020177	0.37	8.13E-06	0.60	1.10E-06	**0.80**	**1.37E-08**
PLK3	polo-like kinase 3	NM_004073	0.35	8.20E-06	0.87	1.10E-09	**0.90**	**6.44E-10**
VIP	vasoactive intestinal peptide	NM_003381	0.49	8.26E-06	1.13	4.90E-09	**1.12**	**5.39E-09**
PPP1R16B	protein phosphatase 1. regulatory subunit 16B	NM_001172735	-0.34	8.69E-06	-0.62	2.06E-07	**-0.88**	**5.30E-10**
VLDLR	very low density lipoprotein receptor	NM_001018056	-0.36	8.85E-06	0.12	1.75E-01	**0.87**	**2.21E-09**
LTBP2	latent transforming growth factor beta binding protein 2	NM_000428	-0.29	8.90E-06	-0.49	8.12E-07	**-0.62**	**1.78E-08**
LOC729218	uncharacterized LOC729218	NR_103825	0.44	8.99E-06	0.99	8.41E-09	**0.93**	**2.53E-08**
TGM2	transglutaminase 2	ENST00000361475	-0.25	9.38E-06	-0.56	8.93E-09	**-0.52**	**2.65E-08**
SLC35F6	solute carrier family 35. member F6	NM_017877	0.37	9.73E-06	0.70	1.74E-07	**0.85**	**7.02E-09**
PSPH	phosphoserine phosphatase	NM_004577	-0.32	9.78E-06	0.42	2.96E-05	**0.88**	**2.69E-10**
ZNF175	zinc finger protein 175	NM_007147	0.44	9.89E-06	0.65	6.32E-06	**1.05**	**3.50E-09**
LOC100288069	uncharacterized LOC100288069	NR_033908	0.30	1.11E-05	0.97	1.54E-11	**0.63**	**2.95E-08**
RFK	riboflavin kinase	ENST00000479197	-0.30	1.15E-05	-0.67	1.22E-08	**-0.65**	**2.32E-08**
NETO2	neuropilin (NRP) and tolloid (TLL)-like 2	NM_001201477	-0.29	1.17E-05	-0.57	9.51E-08	**-0.69**	**3.82E-09**
EVC2	Ellis van Creveld syndrome 2	NM_001166136	0.45	1.20E-05	1.23	3.70E-10	**1.31**	**1.17E-10**
XPC	xeroderma pigmentosum. complementation group C	NM_001145769	0.32	1.22E-05	0.72	1.42E-08	**0.76**	**4.80E-09**
TBC1D3B	TBC1 domain family. member 3B	NM_001001417	0.34	1.22E-05	0.76	1.73E-08	**0.84**	**2.74E-09**
PHF8	PHD finger protein 8	NM_001184896	0.45	1.24E-05	0.64	1.07E-05	**1.08**	**3.54E-09**
NA	NA	TCONS_l2_00005763-XLOC_l2_003052	0.46	1.31E-05	1.09	5.67E-09	**1.29**	**3.03E-10**
GKAP1	G kinase anchoring protein 1	NM_001135953	0.65	1.36E-05	1.40	2.57E-08	**1.62**	**2.13E-09**
NFKB2	nuclear factor of kappa light polypeptide gene enhancer in B-cells 2 (p49/p100)	NM_001077494	0.49	1.41E-05	0.90	3.61E-07	**1.09**	**1.51E-08**
NA	NA	ENST00000384563	1.13	1.43E-05	3.76	1.29E-11	**3.59**	**3.01E-11**
FRS2	fibroblast growth factor receptor substrate 2	NM_001042555	0.41	1.45E-05	1.14	3.98E-10	**1.38**	**1.23E-11**
SLC41A1	solute carrier family 41 (magnesium transporter). member 1	NM_173854	0.28	1.53E-05	0.59	3.88E-08	**0.62**	**1.49E-08**
BMX	BMX non-receptor tyrosine kinase	NM_001721	-0.35	1.56E-05	-0.88	2.28E-09	**-0.87**	**2.75E-09**
TNFRSF12A	tumor necrosis factor receptor superfamily. member 12A	NM_016639	0.45	1.66E-05	0.74	2.20E-06	**1.02**	**1.37E-08**
CHD2	chromodomain helicase DNA binding protein 2	NM_001271	0.30	1.73E-05	0.89	1.71E-10	**1.23**	**3.98E-13**
STS	steroid sulfatase (microsomal). isozyme S	NM_000351	-0.28	1.92E-05	-0.66	8.40E-09	**-0.69**	**3.76E-09**
PSAT1	phosphoserine aminotransferase 1	NM_021154	-0.26	1.94E-05	0.14	4.51E-02	**0.98**	**2.52E-12**
MIR3616	microRNA 3616	NR_037410	-0.68	2.06E-05	-0.94	2.41E-05	**-1.44**	**5.45E-08**
CCIN	calicin	NM_005893	0.58	2.06E-05	1.29	2.83E-08	**1.84**	**5.69E-11**
AEN	apoptosis enhancing nuclease	XM_005254967	0.31	2.08E-05	0.71	2.14E-08	**0.89**	**4.32E-10**
HUNK	hormonally up-regulated Neu-associated kinase	NM_014586	0.70	2.14E-05	1.76	3.36E-09	**2.32**	**2.40E-11**
CLK1	CDC-like kinase 1	NM_001162407	0.33	2.15E-05	0.99	2.13E-10	**1.58**	**2.91E-14**

RICTOR	RPTOR independent companion of MTOR. complex 2	NM_001285439	0.29	2.34E-05	0.61	7.06E-08	**0.70**	**7.90E-09**
MTHFD2	methylenetetrahydrofolate dehydrogenase (NADP+ dependent) 2. methenyltetrahydrofolate cyclohydrolase	NM_006636	0.30	2.36E-05	1.58	4.20E-15	**1.54**	**7.08E-15**
ITSN2	intersectin 2	NM_006277	0.24	2.90E-05	0.63	1.82E-09	**0.58**	**8.77E-09**
C8orf88	chromosome 8 open reading frame 88	NM_001190972	0.52	2.97E-05	1.32	4.40E-09	**1.49**	**5.04E-10**
JUN	jun proto-oncogene	NM_002228	0.29	3.00E-05	0.95	5.49E-11	**1.39**	**4.33E-14**
RWDD2B	RWD domain containing 2B	ENST00000472184	-0.33	3.40E-05	-0.82	6.83E-09	**-0.95**	**5.60E-10**
ZNF461	zinc finger protein 461	NM_153257	0.49	3.48E-05	0.93	5.81E-07	**1.13**	**2.88E-08**
NQO1	NAD(P)H dehydrogenase. quinone 1	NM_000903	-0.25	3.54E-05	-0.55	5.45E-08	**-0.68**	**1.50E-09**
DYSF	dysferlin	NM_001130455	-0.25	3.64E-05	-0.48	4.81E-07	**-0.61**	**1.10E-08**
NHSL2	NHS-like 2	NM_001013627	-0.30	3.65E-05	-0.66	7.97E-08	**-0.80**	**3.21E-09**
GPN1	GPN-loop GTPase 1	NM_001145047	0.25	4.39E-05	0.56	4.68E-08	**0.74**	**4.54E-10**
SPRY4	sprouty homolog 4 (Drosophila)	NM_001293289	0.31	4.49E-05	1.25	2.73E-12	**1.41**	**2.53E-13**
LOC100288069	uncharacterized LOC100288069	NR_033908	0.29	4.51E-05	0.94	1.20E-10	**0.72**	**1.29E-08**
TRIM2	tripartite motif containing 2	NM_001130067	0.37	4.55E-05	0.53	4.04E-05	**0.87**	**2.71E-08**
GMEB2	glucocorticoid modulatory element binding protein 2	NM_012384	0.30	4.78E-05	0.64	1.62E-07	**0.73**	**1.69E-08**
UAP1	UDP-N-acteylglucosamine pyrophosphorylase 1	NM_003115	0.28	4.96E-05	0.47	5.95E-06	**0.78**	**2.60E-09**
ZC3H12A	zinc finger CCCH-type containing 12A	NM_025079	0.32	5.00E-05	0.89	1.72E-09	**1.42**	**3.20E-13**
ANKRD37	ankyrin repeat domain 37	NM_181726	0.41	5.34E-05	0.73	2.97E-06	**0.95**	**5.08E-08**
TIPARP	TCDD-inducible poly(ADP-ribose) polymerase	NM_001184717	0.29	5.36E-05	0.63	1.03E-07	**0.74**	**8.50E-09**
SLC2A1	solute carrier family 2 (facilitated glucose transporter). member 1	NM_006516	0.28	5.40E-05	0.44	1.77E-05	**0.72**	**1.07E-08**
FAT4	FAT atypical cadherin 4	NM_001291285	-0.30	6.53E-05	-0.91	5.31E-10	**-0.84**	**2.33E-09**
ABTB2	ankyrin repeat and BTB (POZ) domain containing 2	NM_145804	0.39	7.09E-05	0.73	1.88E-06	**1.22**	**4.89E-10**
MUM1	melanoma associated antigen (mutated) 1	NM_032853	0.3	7.31E-05	0.70	6.25E-08	**0.82**	**5.38E-09**
BRAF	B-Raf proto-oncogene. serine/threonine kinase	NM_004333	0.28	7.43E-05	0.61	2.08E-07	**0.80**	**2.94E-09**
HDAC9	histone deacetylase 9	NM_001204144	0.32	7.52E-05	1.14	4.57E-11	**0.96**	**1.08E-09**
LRIF1	ligand dependent nuclear receptor interacting factor 1	NM_018372	0.34	7.55E-05	1.02	1.25E-09	**1.62**	**2.54E-13**
KCNT2	potassium channel. subfamily T. member 2	NM_001287819	0.49	7.59E-05	1.22	2.56E-08	**1.77**	**3.37E-11**
TLK2	tousled-like kinase 2	NM_001284333	0.25	7.62E-05	0.70	2.98E-09	**0.61**	**2.62E-08**
NA	NA	NONHSAT068218	0.28	7.68E-05	0.73	1.47E-08	**0.84**	**1.39E-09**
GDPD5	glycerophosphodiester phosphodiesterase domain containing 5	NM_030792	-0.35	8.01E-05	-0.60	7.32E-06	**-0.81**	**7.62E-08**
ASNS	asparagine synthetase (glutamine-hydrolyzing)	NM_001178075	0.32	9.01E-05	0.76	8.66E-08	**1.39**	**1.80E-12**
TBC1D22B	TBC1 domain family. member 22B	NM_017772	0.32	9.13E-05	0.62	1.58E-06	**0.84**	**1.46E-08**
SNORA29	small nucleolar RNA. H/ACA box 29	NR_002965	0.55	1.03E-04	1.12	1.00E-06	**1.53**	**6.63E-09**

SENP1	SUMO1/sentrin specific peptidase 1	NM_001267594	0.28	1.03E-04	0.56	8.17E-07	**0.95**	**1.51E-10**
LDB2	LIM domain binding 2	NM_001130834	-0.27	1.06E-04	-0.80	2.52E-09	**-1.1**	**6.64E-12**
PTX3	pentraxin 3. long	NM_002852	-0.23	1.10E-04	0.00	9.98E-01	**0.58**	**2.78E-08**
GPATCH8	G patch domain containing 8	NM_001002909	0.23	1.12E-04	0.39	1.29E-05	**0.69**	**1.89E-09**
ARNTL	aryl hydrocarbon receptor nuclear translocator-like	NM_001030272	0.25	1.16E-04	0.83	2.55E-10	**0.94**	**2.41E-11**
PECAM1	platelet/endothelial cell adhesion molecule 1	NM_000442	-0.23	1.24E-04	-0.58	4.97E-08	**-0.60**	**2.81E-08**
CHRNB1	cholinergic receptor. nicotinic. beta 1 (muscle)	NM_000747	0.44	1.30E-04	0.93	7.41E-07	**1.22**	**8.97E-09**
TNFRSF9	tumor necrosis factor receptor superfamily. member 9	NM_001561	0.48	1.33E-04	1.41	3.09E-09	**2.77**	**1.00E-14**
ARNTL2	aryl hydrocarbon receptor nuclear translocator-like 2	NM_001248002	0.25	1.35E-04	0.59	1.62E-07	**0.84**	**3.97E-10**
ARSJ	arylsulfatase family. member J	NM_024590	0.26	1.36E-04	0.75	5.35E-09	**1.11**	**4.65E-12**
TRA2B	transformer 2 beta homolog (Drosophila)	NM_001243879	0.23	1.50E-04	0.51	3.32E-07	**0.63**	**1.14E-08**
SLC1A4	solute carrier family 1 (glutamate/neutral amino acid transporter). member 4	NM_003038	0.32	1.72E-04	0.88	1.31E-08	**1.11**	**2.09E-10**
CRYAB	crystallin. alpha B	NM_001289808	0.33	1.72E-04	0.99	3.92E-09	**0.91**	**1.67E-08**
PIK3C2B	phosphatidylinositol-4-phosphate 3-kinase. catalytic subunit type 2 beta	NM_002646	-0.29	1.73E-04	-0.61	1.05E-06	**-0.78**	**2.22E-08**
EGR1	early growth response 1	NM_001964	0.38	1.79E-04	1.04	1.49E-08	**1.89**	**3.25E-13**
ALDH3A2	aldehyde dehydrogenase 3 family. member A2	NM_000382	-0.24	1.80E-04	-0.61	4.82E-08	**-0.59**	**7.92E-08**
FOSL1	FOS-like antigen 1	NM_005438	0.31	1.87E-04	0.62	2.50E-06	**0.80**	**4.36E-08**
LOC101928451	serine/threonine-protein kinase tousled-like 2-like	XR_428252	0.26	2.02E-04	0.96	1.23E-10	**0.71**	**2.77E-08**
TRPS1	trichorhinophalangeal syndrome I	NM_001282903	0.31	2.12E-04	0.83	3.07E-08	**0.99**	**1.39E-09**
LOC100506544	uncharacterized LOC100506544	ENST00000441316	0.37	2.15E-04	0.58	7.30E-05	**0.97**	**6.12E-08**
NA	NA	NONHSAT005623	0.68	2.27E-04	0.50	3.00E-02	**2.04**	**5.08E-09**
TICAM1	toll-like receptor adaptor molecule 1	NM_182919	0.35	2.30E-04	0.86	1.41E-07	**1.00**	**1.36E-08**
SRGAP1	SLIT-ROBO Rho GTPase activating protein 1	NM_020762	0.31	2.38E-04	0.74	2.14E-07	**0.88**	**1.33E-08**
SRSF3	serine/arginine-rich splicing factor 3	NR_036610	0.25	2.58E-04	0.64	6.64E-08	**0.77**	**3.62E-09**
HSPA9	heat shock 70kDa protein 9 (mortalin)	AK023317	0.70	3.00E-04	1.39	4.85E-06	**1.89**	**4.60E-08**
NA	NA	---	0.48	3.03E-04	0.79	6.22E-05	**1.44**	**9.96E-09**
NA	NA	---	0.48	3.03E-04	0.79	6.22E-05	**1.44**	**9.96E-09**
LOC101930489	uncharacterized LOC101930489	XR_249188	0.22	3.06E-04	0.69	5.46E-09	**0.81**	**2.76E-10**
SAMD4A	sterile alpha motif domain containing 4A	NM_001161576	0.26	3.19E-04	0.56	2.19E-06	**0.77**	**1.28E-08**
PIEZO2	piezo-type mechanosensitive ion channel component 2	NM_022068	-0.21	3.44E-04	-0.35	5.11E-05	**-0.57**	**3.92E-08**
SLC7A1	solute carrier family 7 (cationic amino acid transporter. y+ system). member 1	NM_003045	0.29	3.52E-04	1.12	1.88E-10	**1.18**	**6.80E-11**
CMBL	carboxymethylenebutenolidase homolog (Pseudomonas)	NM_138809	-0.22	3.93E-04	-0.43	9.69E-06	**-0.68**	**7.17E-09**
LOC101928706	uncharacterized LOC101928706	ENST00000440038	0.21	4.25E-04	0.96	8.49E-12	**0.62**	**2.10E-08**

IL6	interleukin 6	NM_000600	0.33	4.27E-04	1.04	7.45E-09	**1.24**	**3.00E-10**
NRIP1	nuclear receptor interacting protein 1	NM_003489	0.23	4.50E-04	0.58	3.66E-07	**0.74**	**8.07E-09**
IARS	isoleucyl-tRNA synthetase	NM_002161	0.17	4.53E-04	0.45	1.47E-07	**0.74**	**3.43E-11**
LOC101930489	uncharacterized LOC101930489	XR_249188	0.23	4.57E-04	0.67	1.87E-08	**0.96**	**3.70E-11**
SNHG8	small nucleolar RNA host gene 8 (non-protein coding)	NR_003584	0.21	4.58E-04	0.14	6.57E-02	**0.60**	**5.86E-08**
MAFK	v-maf avian musculoaponeurotic fibrosarcoma oncogene homolog K	NM_002360	0.37	4.60E-04	1.03	7.12E-08	**1.17**	**9.16E-09**
SIAH2	siah E3 ubiquitin protein ligase 2	NM_005067	0.27	4.62E-04	0.74	5.76E-08	**0.77**	**3.03E-08**
HOXA1	homeobox A1	NM_005522	0.24	4.85E-04	0.45	2.50E-05	**0.89**	**6.38E-10**
CLIP4	CAP-GLY domain containing linker protein family. member 4	NM_001287527	0.23	5.48E-04	0.54	1.24E-06	**0.71**	**1.63E-08**
KDM5A	lysine (K)-specific demethylase 5A	NM_001042603	0.21	5.52E-04	0.36	7.08E-05	**0.62**	**3.11E-08**
PAQR3	progestin and adipoQ receptor family member III	ENST00000342820	0.29	5.93E-04	1.09	5.25E-10	**1.75**	**7.72E-14**
PSAT1	phosphoserine aminotransferase 1	NM_021154	-0.32	6.89E-04	-0.08	5.09E-01	**0.98**	**2.33E-08**
ASNSP1	asparagine synthetase pseudogene 1	ENST00000518311	0.31	6.96E-04	0.76	9.37E-07	**1.14**	**1.44E-09**
CRISPLD1	cysteine-rich secretory protein LCCL domain containing 1	NM_031461	-0.25	7.12E-04	-0.71	8.36E-08	**-0.74**	**3.67E-08**
ADCY7	adenylate cyclase 7	NM_001114	0.33	7.27E-04	0.22	7.99E-02	**0.97**	**5.39E-08**
CCDC84	coiled-coil domain containing 84	NM_198489	0.30	7.62E-04	0.96	1.18E-08	**1.32**	**4.58E-11**
bP-21201H5.1	ribosomal protein L7a (RPL7A) pseudogene	OTTHUMT00000469287	0.27	8.69E-04	0.52	3.37E-05	**0.81**	**6.45E-08**
PPP1R3C	protein phosphatase 1. regulatory subunit 3C	NM_005398	0.30	8.77E-04	1.40	1.91E-11	**1.92**	**4.67E-14**
SLC7A5P1	solute carrier family 7 (amino acid transporter light chain. L system). member 5 pseudogene 1	NR_002593	0.30	8.89E-04	1.04	5.40E-09	**0.99**	**1.28E-08**
NKRF	NFKB repressing factor	NM_001173487	0.25	9.82E-04	0.44	1.28E-04	**0.75**	**7.50E-08**
GARS	glycyl-tRNA synthetase	NM_002047	0.17	1.07E-03	0.69	5.39E-10	**0.84**	**1.67E-11**
TTLL12	tubulin tyrosine ligase-like family. member 12	NM_015140	-0.22	1.28E-03	-0.53	2.85E-06	**-0.73**	**2.06E-08**
ZSCAN31	zinc finger and SCAN domain containing 31	ENST00000414429	0.32	1.40E-03	-0.38	5.47E-03	**-1.17**	**5.18E-09**
BCL2A1	BCL2-related protein A1	NM_001114735	0.51	1.41E-03	1.44	2.92E-07	**1.95**	**2.14E-09**
CREB5	cAMP responsive element binding protein 5	NM_004904	0.21	1.41E-03	0.69	3.39E-08	**0.70**	**2.87E-08**
LOC100132062	uncharacterized LOC100132062	uc001aau.3	0.20	1.49E-03	1.02	1.31E-11	**0.67**	**2.35E-08**
STK40	serine/threonine kinase 40	NM_001282546	0.25	1.63E-03	0.72	2.83E-07	**0.86**	**1.71E-08**
ANKFN1	ankyrin-repeat and fibronectin type III domain containing 1	NM_153228	0.31	1.64E-03	2.25	2.30E-14	**2.35**	**9.50E-15**
PDZD2	PDZ domain containing 2	NM_178140	0.32	1.75E-03	1.12	1.83E-08	**1.37**	**5.92E-10**
DUSP5	dual specificity phosphatase 5	NM_004419	0.19	1.79E-03	1.08	2.36E-12	**1.21**	**2.76E-13**
LOC729737	uncharacterized LOC729737	NR_039983	0.20	1.84E-03	1.27	4.62E-13	**0.82**	**1.57E-09**
YARS	tyrosyl-tRNA synthetase	NM_003680	0.16	1.85E-03	0.39	4.23E-06	**0.69**	**4.12E-10**
CARS	cysteinyl-tRNA synthetase	NM_001014437	0.18	1.86E-03	0.94	1.01E-11	**1.17**	**1.77E-13**
UHRF1BP1	UHRF1 binding protein 1	NM_017754	0.27	1.86E-03	0.83	1.61E-07	**0.86**	**7.92E-08**
MTF1	metal-regulatory transcription factor 1	NM_005955	0.22	1.89E-03	0.61	5.18E-07	**0.84**	**3.41E-09**
CYYR1	cysteine/tyrosine-rich 1	AK304124	-0.29	1.90E-03	-1.14	3.03E-09	**-0.94**	**7.54E-08**

CYR61	cysteine-rich. angiogenic inducer. 61	NM_001554	0.17	2.15E-03	0.54	1.30E-07	**0.63**	**9.12E-09**
ABL1	ABL proto-oncogene 1. non-receptor tyrosine kinase	NM_005157	0.19	2.40E-03	0.59	1.56E-07	**0.67**	**2.28E-08**
PDGFA	platelet-derived growth factor alpha polypeptide	NM_002607	0.19	2.64E-03	0.62	1.44E-07	**0.70**	**2.13E-08**
ADAMTS4	ADAM metallopeptidase with thrombospondin type 1 motif. 4	NM_005099	-0.22	2.72E-03	-0.45	5.53E-05	**-0.78**	**2.30E-08**
B3GNT5	UDP-GlcNAc:betaGal beta-1.3-N-acetylglucosaminyltransferase 5	NM_032047	0.24	2.76E-03	0.65	2.33E-06	**0.98**	**3.52E-09**
ASNSP1	asparagine synthetase pseudogene 1	ENST00000518311	0.28	2.85E-03	0.81	9.04E-07	**1.62**	**6.80E-12**
C9orf64	chromosome 9 open reading frame 64	NM_032307	-0.23	3.03E-03	0.20	5.87E-02	**0.81**	**5.29E-08**
MAP4K3	mitogen-activated protein kinase kinase kinase kinase 3	NM_003618	0.17	3.08E-03	0.64	2.09E-08	**0.6**	**5.53E-08**
TAOK3	TAO kinase 3	NM_016281	0.17	3.27E-03	0.43	7.56E-06	**0.64**	**1.47E-08**
FOXN2	forkhead box N2	NM_002158	0.23	3.51E-03	0.56	1.41E-05	**0.90**	**1.29E-08**
UGCG	UDP-glucose ceramide glucosyltransferase	ENST00000374279	-0.24	3.82E-03	0.29	1.22E-02	**0.97**	**7.78E-09**
TRPM7	transient receptor potential cation channel. subfamily M. member 7	NM_017672	0.15	3.85E-03	0.43	2.81E-06	**0.56**	**4.95E-08**
NOTCH2	notch 2	NM_024408	0.17	4.26E-03	0.58	1.54E-07	**0.73**	**3.36E-09**
INA	internexin neuronal intermediate filament protein. alpha	NM_032727	0.27	4.87E-03	0.44	1.44E-03	**1.10**	**9.00E-09**
HNRNPU-AS1	HNRNPU antisense RNA 1	NR_026778	0.35	4.88E-03	1.14	4.63E-07	**1.29**	**6.56E-08**
NRP2	neuropilin 2	NM_003872	-0.17	4.94E-03	-0.34	1.86E-04	**-0.62**	**5.09E-08**
POU2F1	POU class 2 homeobox 1	NM_001198783	0.17	5.08E-03	0.53	1.02E-06	**0.79**	**1.57E-09**
TLK2	tousled-like kinase 2	NM_001284333	0.17	5.13E-03	0.79	1.17E-09	**0.62**	**6.55E-08**
DUSP10	dual specificity phosphatase 10	NR_111940	0.24	5.47E-03	0.93	4.58E-08	**1.35**	**7.15E-11**
PHYH	phytanoyl-CoA 2-hydroxylase	NM_001037537	0.18	5.63E-03	0.33	6.85E-04	**0.70**	**3.91E-08**
FNBP4	formin binding protein 4	NM_015308	0.14	5.68E-03	0.48	3.02E-07	**0.63**	**3.63E-09**
LPIN2	lipin 2	NM_014646	0.17	5.73E-03	0.53	1.81E-06	**0.75**	**6.01E-09**
CD200	CD200 molecule	NM_001004196	-0.29	6.32E-03	-0.87	1.82E-06	**-1.08**	**7.23E-08**
ZNF274	zinc finger protein 274	NM_001278734	0.30	6.59E-03	0.83	6.93E-06	**1.31**	**6.14E-09**
PYROXD1	pyridine nucleotide-disulphide oxidoreductase domain 1	NM_024854	0.20	6.71E-03	0.46	8.86E-05	**0.79**	**4.01E-08**
NA	NA	NONHSAT113325	0.50	7.60E-03	0.55	3.30E-02	**1.99**	**5.09E-08**
CLIP1	CAP-GLY domain containing linker protein 1	NM_001247997	0.13	7.96E-03	0.39	4.62E-06	**0.55**	**2.44E-08**
OXTR	oxytocin receptor	NM_000916	0.22	8.44E-03	0.88	5.10E-08	**1.07**	**1.80E-09**
MTMR9	myotubularin related protein 9	NM_015458	0.20	9.81E-03	0.64	2.91E-06	**0.81**	**8.00E-08**
LRP4	low density lipoprotein receptor-related protein 4	NM_002334	0.25	1.11E-02	0.84	1.73E-06	**1.14**	**1.50E-08**
RAET1K	retinoic acid early transcript 1K pseudogene	NR_024045	0.47	1.12E-02	1.44	6.14E-06	**2.42**	**1.77E-09**
LINC00152	long intergenic non-protein coding RNA 152	ENST00000439362	0.17	1.17E-02	0.6	6.25E-07	**0.79**	**8.27E-09**
PIK3R3	phosphoinositide-3-kinase. regulatory subunit 3 (gamma)	NM_001114172	-0.19	1.18E-02	-0.92	4.85E-09	**-1.01**	**9.79E-10**
MMRN1	multimerin 1	NM_007351	-0.16	1.29E-02	-0.58	5.17E-07	**-0.66**	**6.43E-08**
AC100802.3	putative novel transcript	OTTHUMT00000375116	0.33	1.36E-02	1.10	2.98E-06	**2.06**	**1.01E-10**
ZC3HAV1	zinc finger CCCH-type. antiviral 1	NM_020119	-0.13	1.62E-02	0.45	2.30E-06	**0.71**	**2.05E-09**

CWF19L2	CWF19-like 2. cell cycle control (S. pombe)	ENST00000532251	0.23	2.04E-02	0.71	2.36E-05	1.97	1.18E-12
EIF1B	eukaryotic translation initiation factor 1B	NM_005875	0.14	2.11E-02	0.54	1.37E-06	0.66	6.58E-08
LINC-PINT	long intergenic non-protein coding RNA. p53 induced transcript	NR_034120	0.22	2.31E-02	1.31	1.52E-09	1.40	4.16E-10
NAV2	neuron navigator 2	NM_001111018	0.12	2.67E-02	0.71	1.37E-09	0.65	6.38E-09
BIN1	bridging integrator 1	NM_139343	-0.13	2.68E-02	-0.43	1.42E-05	-0.63	6.73E-08
STC2	stanniocalcin 2	NM_003714	-0.12	2.69E-02	0.57	1.13E-07	0.79	4.23E-10
SEPHS2	selenophosphate synthetase 2	NM_012248	0.14	3.09E-02	0.42	7.07E-05	0.69	7.22E-08
JUND	jun D proto-oncogene	NM_001286968	0.15	3.15E-02	0.65	6.22E-07	0.76	4.72E-08
OTUD1	OTU deubiquitinase 1	NM_001145373	0.15	3.23E-02	0.84	1.06E-08	1.04	3.00E-10
LAMB3	laminin. beta 3	NM_000228	0.18	3.23E-02	0.61	1.99E-05	1.06	5.15E-09
RGS7	regulator of G-protein signaling 7	NM_001282773	0.24	3.27E-02	0.76	6.01E-05	1.33	1.90E-08
LAMP3	lysosomal-associated membrane protein 3	NM_014398	0.22	3.43E-02	0.93	1.21E-06	1.27	8.82E-09
NOTCH2NL	notch 2 N-terminal like	AK075065	0.13	3.46E-02	0.36	1.58E-04	0.73	7.12E-09
CDC42EP2	CDC42 effector protein (Rho GTPase binding) 2	ENST00000533419	-0.16	3.63E-02	0.50	4.70E-05	0.97	2.50E-09
HAS2	hyaluronan synthase 2	NM_005328	0.19	3.80E-02	1.05	1.69E-08	1.38	1.57E-10
LOC541472	uncharacterized LOC541472	ENST00000325042	0.21	3.90E-02	0.52	8.58E-04	1.34	1.72E-09
PLXDC2	plexin domain containing 2	NM_001282736	-0.14	4.45E-02	-0.55	8.09E-06	-0.78	4.23E-08
KLF6	Kruppel-like factor 6	NM_001160124	0.12	4.49E-02	0.98	2.44E-11	1.14	1.41E-12
ATF4	activating transcription factor 4	NM_001675	0.10	4.74E-02	0.69	1.49E-09	0.89	1.27E-11
NOTCH2NL	notch 2 N-terminal like	ENST00000479995	0.15	4.76E-02	0.71	5.35E-07	0.82	5.89E-08
LYVE1	lymphatic vessel endothelial hyaluronan receptor 1	NM_006691	-0.11	4.90E-02	-0.36	8.88E-05	-0.68	1.12E-08
RP11-819C21.1	novel transcript	OTTHUMT00000421595	0.23	4.99E-02	1.32	2.57E-08	1.66	5.20E-10
IL31RA	interleukin 31 receptor A	NM_001242636	0.14	5.44E-02	0.60	3.18E-06	1.01	6.42E-10
PKD1P1	polycystic kidney disease 1 (autosomal dominant) pseudogene 1	NR_036447	0.10	5.91E-02	0.82	2.05E-10	0.82	2.16E-10
HRH1	histamine receptor H1	NM_000861	0.13	6.11E-02	0.55	1.02E-05	0.92	4.51E-09
ATP6V0D2	ATPase. H+ transporting. lysosomal 38kDa. V0 subunit d2	ENST00000285393	0.18	6.64E-02	2.33	7.42E-14	2.17	2.97E-13
FAF2	Fas associated factor family member 2	NM_014613	0.09	6.85E-02	0.41	6.48E-06	0.58	3.50E-08
C6orf58	chromosome 6 open reading frame 58	NM_001010905	0.21	6.95E-02	1.17	3.33E-07	1.70	6.65E-10
NA	NA	NONHSAT127377	0.22	7.05E-02	1.30	6.95E-08	1.74	5.04E-10
OLAH	oleoyl-ACP hydrolase	NM_001039702	-0.22	7.13E-02	1.32	7.18E-08	1.8	3.39E-10
SGTB	small glutamine-rich tetratricopeptide repeat (TPR)-containing. beta	NM_019072	0.11	7.45E-02	0.59	4.50E-07	0.78	5.05E-09
RRAS2	related RAS viral (r-ras) oncogene homolog 2	NM_001102669	0.12	7.49E-02	0.62	6.82E-07	0.95	7.26E-10
RP11-3L8.3	novel transcript	OTTHUMT00000051732	-0.16	7.50E-02	0.99	7.25E-08	1.22	2.45E-09
PELI1	pellino E3 ubiquitin protein ligase 1	NM_020651	0.09	8.23E-02	0.56	6.37E-08	0.76	3.68E-10
MARS	methionyl-tRNA synthetase	NM_004990	0.09	8.75E-02	0.53	1.39E-07	0.77	2.92E-10
LOC101929475	uncharacterized LOC101929475	XR_242755	-0.13	1.03E-01	0.58	3.46E-05	1.27	1.63E-10
TOB2P1	transducer of ERBB2. 2 pseudogene 1	NR_002936	0.16	1.16E-01	0.61	2.39E-04	1.22	1.96E-08

RPRD1B	regulation of nuclear pre-mRNA domain containing 1B	NM_021215	0.10	1.17E-01	0.62	7.25E-07	**0.81**	**1.02E-08**
METTL7A	methyltransferase like 7A	NM_014033	-0.15	1.32E-01	-0.77	1.27E-05	**-1.22**	**1.39E-08**
TNFRSF10B	tumor necrosis factor receptor superfamily. member 10b	NM_003842	0.09	1.38E-01	0.55	1.69E-06	**0.81**	**4.20E-09**
PKD1P1	polycystic kidney disease 1 (autosomal dominant) pseudogene 1	NR_036447	0.08	1.39E-01	0.88	5.54E-11	**0.82**	**1.99E-10**
C17orf51	chromosome 17 open reading frame 51	NM_001113434	0.11	1.41E-01	0.95	5.48E-09	**1.00**	**2.09E-09**
RASGRF2	Ras protein-specific guanine nucleotide-releasing factor 2	NM_006909	0.10	1.45E-01	0.83	1.79E-08	**0.84**	**1.66E-08**
UBALD2	UBA-like domain containing 2	NM_182565	0.08	1.56E-01	1.03	2.27E-11	**0.90**	**2.90E-10**
FBXL14	F-box and leucine-rich repeat protein 14	NM_152441	0.12	1.79E-01	0.73	3.99E-06	**1.07**	**1.23E-08**
SDC4	syndecan 4	NM_002999	0.09	1.79E-01	0.83	1.14E-08	**1.46**	**4.22E-13**
ZSCAN29	zinc finger and SCAN domain containing 29	NM_152455	0.09	1.87E-01	0.54	9.32E-06	**0.96**	**1.04E-09**
AKAP10	A kinase (PRKA) anchor protein 10	NM_007202	0.06	1.94E-01	0.37	2.12E-05	**0.74**	**5.68E-10**
MARK3	MAP/microtubule affinity-regulating kinase 3	NM_001128918	0.07	1.97E-01	0.41	2.91E-05	**0.67**	**2.47E-08**
DISC1	disrupted in schizophrenia 1	NM_001012957	0.08	2.17E-01	0.56	1.28E-06	**0.77**	**8.93E-09**
TLL1	tolloid-like 1	NM_012464	0.10	2.22E-01	-0.69	2.91E-06	**-0.93**	**3.33E-08**
RALGAPA2	Ral GTPase activating protein. alpha subunit 2 (catalytic)	NM_020343	0.07	2.26E-01	-0.19	1.60E-02	**-0.60**	**7.40E-08**
EFTUD1	elongation factor Tu GTP binding domain containing 1	NM_001040610	0.07	2.52E-01	0.62	8.39E-07	**0.79**	**1.51E-08**
ZNF827	zinc finger protein 827	NM_178835	0.06	2.56E-01	0.54	6.17E-07	**0.65**	**3.13E-08**
LOC101929475	uncharacterized LOC101929475	XR_242755	-0.09	2.59E-01	0.6	1.16E-05	**1.27**	**6.97E-11**
PLSCR4	phospholipid scramblase 4	NM_001128304	-0.07	2.72E-01	-0.65	1.39E-07	**-0.69**	**5.05E-08**
PVR	poliovirus receptor	NM_001135770	0.05	2.84E-01	0.34	5.28E-05	**0.62**	**9.89E-09**
DCBLD2	discoidin. CUB and LCCL domain containing 2	NM_080927	0.04	3.21E-01	0.43	4.92E-07	**0.52**	**1.97E-08**
NA	NA	NONHSAT008377	0.06	3.68E-01	0.75	1.39E-07	**1.08**	**3.36E-10**
SH2D4A	SH2 domain containing 4A	NM_022071	0.05	3.82E-01	0.66	2.17E-07	**1.01**	**1.39E-10**
RHOB	ras homolog family member B	NM_004040	0.04	4.22E-01	0.89	8.02E-11	**0.90**	**7.02E-11**
IL20RB	interleukin 20 receptor beta	NM_144717	0.08	4.37E-01	0.84	9.60E-06	**1.32**	**1.06E-08**
METRNL	meteorin. glial cell differentiation regulator-like	NM_001004431	-0.04	4.78E-01	0.43	2.88E-05	**0.68**	**4.49E-08**
GRPEL2	GrpE-like 2. mitochondrial (E. coli)	NM_152407	0.05	4.78E-01	1.04	2.31E-10	**1.07**	**1.35E-10**
NEK11	NIMA-related kinase 11	NM_001146003	0.06	4.94E-01	0.54	1.34E-04	**0.94**	**6.51E-08**
FBXL13	F-box and leucine-rich repeat protein 13	NR_105043	0.05	5.10E-01	0.53	1.50E-04	**0.96**	**4.70E-08**
SCML2	sex comb on midleg-like 2 (Drosophila)	NM_006089	0.04	5.21E-01	0.17	7.96E-02	**0.82**	**1.76E-08**
CSF3	colony stimulating factor 3 (granulocyte)	NM_000759	0.06	5.29E-01	1.75	1.43E-11	**3.09**	**2.38E-16**
CBS	cystathionine-beta-synthase	NM_001178008	0.03	5.75E-01	0.40	5.53E-05	**0.71**	**1.36E-08**
ZNF143	zinc finger protein 143	NM_001282656	0.03	5.88E-01	0.47	1.38E-05	**1.16**	**4.69E-12**
IFFO2	intermediate filament family orphan 2	NM_001136265	-0.03	5.93E-01	0.48	1.20E-05	**0.79**	**5.21E-09**
STARD13	StAR-related lipid transfer (START) domain containing 13	NM_001243466	-0.04	6.02E-01	0.18	6.78E-02	**0.79**	**5.13E-08**

ZFP36	ZFP36 ring finger protein	NM_003407	0.04	6.61E-01	0.90	1.64E-07	**1.24**	**7.92E-10**
DLGAP1-AS2	DLGAP1 antisense RNA 2	ENST00000572856	0.03	6.63E-01	1.07	1.44E-09	**1.13**	**5.52E-10**
GPR176	G protein-coupled receptor 176	NM_001271854	-0.04	6.63E-01	0.41	2.09E-03	**0.98**	**4.19E-08**
PKD1P5	polycystic kidney disease 1 (autosomal dominant) pseudogene 5	ENST00000532415	0.02	7.69E-01	0.84	8.23E-10	**0.91**	**1.76E-10**
SLC8B1	solute carrier family 8 (sodium/lithium/calcium exchanger). member B1	NM_024959	-0.02	7.84E-01	0.27	8.68E-03	**0.79**	**3.18E-08**
LOC101930106	uncharacterized LOC101930106	XR_252253	0.05	7.93E-01	1.55	2.94E-06	**2.12**	**2.50E-08**
RP11-54A9.1	novel transcript	OTTHUMT00000406353	0.01	8.75E-01	0.44	2.69E-04	**1.11**	**4.09E-10**
KCNMB1	potassium large conductance calcium-activated channel. subfamily M. beta member 1	NM_004137	0.01	9.08E-01	0.41	3.57E-04	**0.89**	**9.26E-09**
NPIPA1	nuclear pore complex interacting protein family. member A1	ENST00000472413	-0.01	9.25E-01	0.83	6.96E-10	**0.77**	**2.76E-09**
TARS	threonyl-tRNA synthetase	NM_001258438	0.00	9.39E-01	0.47	9.68E-06	**0.65**	**7.02E-08**
CCL20	chemokine (C-C motif) ligand 20	NM_001130046	0.01	9.41E-01	0.79	4.48E-05	**1.45**	**5.58E-09**
AC090044.2	novel transcript	OTTHUMT00000337494	0.00	9.78E-01	0.86	1.71E-08	**1.23**	**2.96E-11**

NA; not applicable. FC; fold change n= 6 for 12 h. n= 3 for 18 h and 24 h.

Table S4. Altered gene expression due to NOX5-β expression at all the analysed times.

GeneName	GeneDescription	TranscriptID	N12h *vs* G12h		N18h *vs* G18h		N24h *vs* G24h	
			logFC	*p-value*	logFC	*p-value*	logFC	*p-value*
PTGS2	prostaglandin-endoperoxide synthase 2 (prostaglandin G/H synthase and cyclooxygenase)	NM_000963	3.80	3.74E-24	3.89	2.89E-21	2.68	5.83E-18
RND1	Rho family GTPase 1	ENST00000548445	3.09	2.20E-20	3.42	3.17E-18	3.25	8.99E-18
DNAJB1	DnaJ (Hsp40) homolog. subfamily B. member 1	NM_006145	1.95	2.72E-20	2.30	1.04E-18	2.13	5.07E-18
DNAJA4	DnaJ (Hsp40) homolog. subfamily A. member 4	NM_001130182	2.19	9.83E-20	2.94	2.90E-19	3.01	1.68E-19
IL7R	interleukin 7 receptor	NM_002185	2.17	1.43E-19	2.62	3.61E-18	2.36	3.12E-17
ZFAND2A	zinc finger. AN1-type domain 2A	NM_182491	1.99	2.08E-19	1.84	1.12E-15	1.13	1.36E-11
BAG3	BCL2-associated athanogene 3	NM_004281	1.93	3.06E-19	2.00	1.62E-16	1.91	4.28E-16
CCRN4L	CCR4 carbon catabolite repression 4-like (S. cerevisiae)	NM_012118	1.92	4.22E-19	2.05	1.30E-16	2.13	6.21E-17
NA	NA	RPTR-U57609-1	-7.43	5.14E-19	-6.37	1.18E-14	-6.56	6.76E-15
NA	NA	RPTR-U43284-1	-7.43	5.14E-19	-6.37	1.18E-14	-6.56	6.76E-15
NA	NA	RPTR-AB076373-2	-7.43	5.14E-19	-6.37	1.18E-14	-6.56	6.76E-15
HSPA1A	heat shock 70kDa protein 1A	NM_005345	1.46	5.46E-19	1.38	1.97E-15	1.17	5.07E-14
RP11-274H2.3	novel transcript	OTTHUMT00000355183	2.01	1.27E-18	2.29	9.69E-17	2.14	3.92E-16
HSPA1A	heat shock 70kDa protein 1A	NM_005345	1.45	1.91E-18	1.37	6.50E-15	1.17	1.43E-13
HSPA1A	heat shock 70kDa protein 1A	NM_005345	1.33	2.81E-18	1.24	1.08E-14	1.02	4.79E-13
HSPA1A	heat shock 70kDa protein 1A	NM_005345	1.33	2.81E-18	1.24	1.08E-14	1.02	4.79E-13
HSPA1A	heat shock 70kDa protein 1A	NM_005345	1.33	2.81E-18	1.24	1.08E-14	1.02	4.79E-13
HSPA1A	heat shock 70kDa protein 1A	NM_005345	1.33	3.05E-18	1.26	1.05E-14	1.04	4.03E-13
HSPA1B	heat shock 70kDa protein 1B	NM_005346	1.43	3.60E-18	1.39	6.61E-15	1.18	1.58E-13
HSPD1	heat shock 60kDa protein 1 (chaperonin)	NM_002156	1.34	9.15E-18	1.36	7.26E-15	1.40	3.76E-15
HSPA1B	heat shock 70kDa protein 1B	NM_005346	1.47	9.72E-18	1.36	4.56E-14	1.18	7.30E-13
FAM129A	family with sequence similarity 129. member A	NM_052966	1.45	1.20E-17	2.44	3.27E-19	2.96	6.24E-21
HSPA1B	heat shock 70kDa protein 1B	NM_005346	1.25	1.30E-17	1.23	1.68E-14	1.05	3.92E-13
HSPH1	heat shock 105kDa/110kDa protein 1	NM_001286503	1.35	1.53E-17	1.41	6.52E-15	1.34	1.93E-14
HSPA1B	heat shock 70kDa protein 1B	NM_005346	1.33	1.55E-17	1.29	3.04E-14	1.07	1.06E-12
KIF21A	kinesin family member 21A	NM_001173463	1.55	2.58E-17	1.91	4.47E-16	1.70	4.18E-15
MXD1	MAX dimerization protein 1	NM_001202513	1.46	2.88E-17	2.08	2.44E-17	1.81	3.86E-16
STK38L	serine/threonine kinase 38 like	NM_015000	1.44	4.06E-17	1.67	2.05E-15	1.22	9.69E-13
CXCL8	chemokine (C-X-C motif) ligand 8	NM_000584	2.28	5.88E-17	2.23	9.11E-14	2.03	5.65E-13
CPEB4	cytoplasmic polyadenylation element binding protein 4	NM_030627	1.42	6.06E-17	1.91	1.56E-16	1.56	9.34E-15
CHRNA5	cholinergic receptor. nicotinic. alpha 5 (neuronal)	NM_000745	1.30	6.71E-17	1.78	1.31E-16	1.99	1.44E-17
SLC19A2	solute carrier family 19 (thiamine transporter). member 2	NM_006996	1.30	7.50E-17	1.32	5.41E-14	0.68	1.07E-08
KITLG	KIT ligand	NM_000899	1.52	7.96E-17	2.12	1.05E-16	1.97	4.77E-16
BMP2	bone morphogenetic protein 2	NM_001200	1.60	1.08E-16	2.16	2.80E-16	2.04	9.00E-16
KDM7A	lysine (K)-specific demethylase 7A	NM_030647	1.61	1.28E-16	2.02	1.52E-15	1.66	6.87E-14

NA	NA	RPTR-AY189981-1	-6.80	3.16E-16	-5.56	1.37E-11	-5.79	6.41E-12
LRRC8B	leucine rich repeat containing 8 family. member B	NM_001134476	1.01	3.23E-16	1.03	1.96E-13	1.00	4.13E-13
GPR89A	G protein-coupled receptor 89A	XM_006711492	1.10	4.66E-16	1.44	2.24E-15	1.69	8.05E-17
HSPA5	heat shock 70kDa protein 5 (glucose-regulated protein. 78kDa)	NM_005347	0.88	7.41E-16	1.06	2.04E-14	0.69	6.60E-11
HSPA4L	heat shock 70kDa protein 4-like	NM_014278	1.22	9.72E-16	1.52	1.25E-14	1.90	1.45E-16
GPR89A	G protein-coupled receptor 89A	NM_001097612	1.05	1.23E-15	1.44	2.19E-15	1.70	8.33E-17
LOC727896	cysteine and histidine-rich domain (CHORD) containing 1 pseudogene	NR_026659	1.33	1.29E-15	1.64	1.89E-14	1.80	3.16E-15
NA	NA	---	1.50	2.08E-15	1.86	2.82E-14	2.04	4.78E-15
NA	NA	RPTR-AJ510163-3	-6.55	3.02E-15	-5.20	1.84E-10	-5.48	7.04E-11
FAM46A	family with sequence similarity 46. member A	ENST00000369754	1.76	3.05E-15	1.82	1.37E-12	1.84	1.14E-12
TSPYL2	TSPY-like 2	ENST00000556808	1.21	3.27E-15	1.21	2.84E-12	1.46	7.97E-14
SESN2	sestrin 2	NM_031459	1.70	3.90E-15	2.81	1.70E-16	2.53	1.37E-15
CAP2	CAP. adenylate cyclase-associated protein. 2 (yeast)	NM_006366	0.85	5.80E-15	1.09	4.04E-14	1.27	1.90E-15
AHSA1	AHA1. activator of heat shock 90kDa protein ATPase homolog 1 (yeast)	NM_012111	1.05	1.15E-14	1.13	2.45E-12	1.28	2.15E-13
FKBP4	FK506 binding protein 4. 59kDa	NM_002014	0.98	1.37E-14	1.02	4.80E-12	1.23	1.27E-13
ERN1 (IRE1α)	endoplasmic reticulum to nucleus signaling 1	NM_001433	1.32	1.37E-14	2.24	4.02E-16	2.12	1.16E-15
ACTRT3	actin-related protein T3	NM_032487	1.55	1.52E-14	2.48	1.35E-15	2.94	4.50E-17
PLAA	phospholipase A2-activating protein	NM_001031689	0.80	1.65E-14	0.73	6.74E-11	0.59	2.91E-09
NA	NA	RPTR-AF292560-1	-2.57	1.70E-14	-2.44	3.61E-11	-2.69	5.69E-12
BACH1	BTB and CNC homology 1. basic leucine zipper transcription factor 1	NR_027655	1.12	1.74E-14	1.61	1.19E-14	1.67	5.86E-15
DDIT3 (CHOP)	DNA-damage-inducible transcript 3	NM_001195053	1.92	1.77E-14	2.10	2.74E-12	1.82	4.06E-11
SDE2	SDE2 telomere maintenance homolog (S. pombe)	NM_152608	1.17	1.99E-14	0.81	9.37E-09	0.84	5.71E-09
ELL2	elongation factor. RNA polymerase II. 2	NM_012081	0.83	3.86E-14	1.27	7.84E-15	0.94	2.71E-12
NA	NA	NONHSAT098813	1.72	4.11E-14	1.48	4.62E-10	1.63	8.88E-11
HSP90AA1	heat shock protein 90kDa alpha (cytosolic). class A member 1	NM_001017963	0.89	4.90E-14	0.91	2.19E-11	0.90	2.93E-11
USP53	ubiquitin specific peptidase 53	NM_019050	0.93	5.15E-14	1.45	7.98E-15	1.49	4.92E-15
TNFAIP3	tumor necrosis factor. alpha-induced protein 3	NM_001270507	1.20	5.69E-14	1.72	4.34E-14	1.83	1.28E-14
TRIM26	tripartite motif containing 26	NM_001242783	0.97	6.11E-14	1.04	1.11E-11	1.17	1.41E-12
HYOU1	hypoxia up-regulated 1	NM_001130991	0.77	8.18E-14	0.98	6.17E-13	0.77	4.97E-11
TRIM26	tripartite motif containing 26	NM_001242783	1.00	9.55E-14	1.09	1.37E-11	1.21	1.90E-12
TRIM26	tripartite motif containing 26	NM_001242783	0.96	9.89E-14	1.04	1.67E-11	1.16	2.20E-12
EPSTI1	epithelial stromal interaction 1 (breast)	NM_001002264	1.52	1.10E-13	1.53	6.47E-11	1.13	1.43E-08
HSPA6	heat shock 70kDa protein 6 (HSP70B)	NM_002155	1.95	1.18E-13	2.17	1.16E-11	2.15	1.38E-11

TRIM26	tripartite motif containing 26	ENST00000436219	0.99	1.30E-13	1.07	2.13E-11	1.11	1.07E-11
IKZF5	IKAROS family zinc finger 5 (Pegasus)	NM_001271840	0.85	1.63E-13	0.93	1.96E-11	1.07	1.52E-12
TRIM26	tripartite motif containing 26	NM_001242783	0.97	1.89E-13	1.05	3.46E-11	1.17	4.09E-12
TRIM26	tripartite motif containing 26	NM_001242783	0.97	1.89E-13	1.05	3.46E-11	1.17	4.09E-12
RP11-212D19.4	novel transcript. RBM7-REXO2 readthrough	OTTHUMT00000399017	2.51	1.98E-13	3.50	2.55E-13	4.13	1.03E-14
SAT1	spermidine/spermine N1-acetyltransferase 1	NM_002970	0.82	2.04E-13	1.76	4.50E-17	1.73	6.60E-17
HSPA4	heat shock 70kDa protein 4	NM_002154	0.78	2.06E-13	0.97	2.32E-12	0.81	6.54E-11
DNAJA1	DnaJ (Hsp40) homolog. subfamily A. member 1	NM_001539	0.82	2.23E-13	0.98	5.07E-12	1.01	2.92E-12
ARG2	arginase 2	NM_001172	0.94	2.30E-13	1.48	3.27E-14	1.25	8.53E-13
CRY1	cryptochrome circadian clock 1	NM_004075	1.06	2.39E-13	1.15	3.83E-11	0.89	3.66E-09
TRIM26	tripartite motif containing 26	NM_001242783	0.99	2.46E-13	1.13	1.42E-11	1.23	2.88E-12
UBR3	ubiquitin protein ligase E3 component n-recognin 3 (putative)	ENST00000272793	0.77	3.55E-13	0.74	4.82E-10	0.62	9.23E-09
TRIM26	tripartite motif containing 26	ENST00000415923	0.99	3.56E-13	1.11	2.71E-11	1.15	1.38E-11
NAA16	N(alpha)-acetyltransferase 16. NatA auxiliary subunit	NM_001110798	0.91	3.86E-13	1.44	3.87E-14	1.51	1.51E-14
UHRF1BP1L	UHRF1 binding protein 1-like	NM_001006947	1.07	4.84E-13	1.37	3.60E-12	1.28	1.26E-11
FNDC3A	fibronectin type III domain containing 3A	NM_001079673	0.70	4.89E-13	0.94	1.22E-12	0.59	6.48E-09
DEDD2	death effector domain containing 2	NM_001270614	1.55	5.06E-13	2.16	6.66E-13	2.12	9.04E-13
IL1A	interleukin 1. alpha	NM_000575	1.35	5.27E-13	2.04	1.44E-13	2.19	3.66E-14
PLOD2	procollagen-lysine. 2-oxoglutarate 5-dioxygenase 2	NM_000935	0.74	5.44E-13	0.95	2.84E-12	0.91	7.45E-12
ARID5A	AT rich interactive domain 5A (MRF1-like)	NM_212481	1.01	5.46E-13	1.01	3.59E-10	0.80	1.93E-08
CACYBP	calcyclin binding protein	NM_001007214	0.89	5.80E-13	1.14	4.23E-12	1.18	2.27E-12
KIAA0513	16q24.1	NM_001286565	1.60	5.92E-13	1.82	3.85E-11	1.55	7.15E-10
GADD45B	growth arrest and DNA-damage-inducible. beta	NM_015675	0.88	6.24E-13	1.53	1.25E-14	1.86	2.66E-16
ME1	malic enzyme 1. NADP(+)-dependent. cytosolic	NM_002395	0.68	7.04E-13	0.90	2.39E-12	0.75	7.84E-11
GOT1	glutamic-oxaloacetic transaminase 1. soluble	NM_002079	0.99	7.08E-13	1.75	8.15E-15	1.67	2.11E-14
RAB23	RAB23. member RAS oncogene family	NM_001278666	0.96	7.49E-13	1.51	8.40E-14	1.48	1.24E-13
PPP1R15A	protein phosphatase 1. regulatory subunit 15A	NM_014330	1.16	8.86E-13	1.73	3.11E-13	2.08	8.84E-15
LOC344887	NmrA-like family domain containing 1 pseudogene	NR_033752	0.83	9.97E-13	1.07	6.33E-12	1.04	1.02E-11
IER5	immediate early response 5	NM_016545	1.01	1.06E-12	1.85	6.09E-15	2.16	2.98E-16
SLC39A14	solute carrier family 39 (zinc transporter). member 14	NM_001128431	1.06	1.21E-12	1.35	8.98E-12	1.14	2.10E-10
ABHD3	abhydrolase domain containing 3	NM_138340	1.30	1.39E-12	1.58	2.55E-11	1.97	3.65E-13
PMAIP1	phorbol-12-myristate-13-acetate-induced protein 1	NM_021127	0.93	1.84E-12	2.41	1.32E-17	2.69	1.43E-18
ATF3	activating transcription factor 3	NM_001030287	2.75	1.94E-12	3.39	2.52E-11	3.03	1.95E-10
TNFSF18	tumor necrosis factor (ligand) superfamily. member 18	NM_005092	-1.03	2.38E-12	-0.83	5.96E-08	-0.84	4.37E-08

PSMC4	proteasome (prosome. macropain) 26S subunit. ATPase. 4	NM_006503	0.74	3.40E-12	0.92	3.74E-11	0.76	1.27E-09
MLLT11	myeloid/lymphoid or mixed-lineage leukemia (trithorax homolog. Drosophila); translocated to. 11	NM_006818	0.57	4.04E-12	0.69	7.50E-11	0.68	9.47E-11
HSPA9	heat shock 70kDa protein 9 (mortalin)	NM_004134	0.69	4.25E-12	0.88	3.08E-11	0.90	1.94E-11
C7orf60	chromosome 7 open reading frame 60	NM_152556	0.99	4.36E-12	1.79	3.96E-14	2.07	2.35E-15
PIGA	phosphatidylinositol glycan anchor biosynthesis. class A	NM_002641	0.80	4.52E-12	1.12	5.41E-12	1.00	4.27E-11
YRDC	yrdC N(6)-threonylcarbamoyltransferase domain containing	NM_024640	0.67	6.52E-12	0.83	8.03E-11	0.85	4.79E-11
UBC	ubiquitin C	ENST00000536769	1.04	7.82E-12	1.27	1.30E-10	1.01	6.97E-09
KCMF1	potassium channel modulatory factor 1	NM_020122	0.65	8.39E-12	0.76	2.34E-10	0.64	5.10E-09
NA	NA	TCONS_l2_00014930-XLOC_l2_008285	0.80	8.67E-12	1.05	3.47E-11	0.99	1.03E-10
PSMD14	proteasome (prosome. macropain) 26S subunit. non-ATPase. 14	NM_005805	0.72	9.29E-12	0.83	4.72E-10	0.70	9.45E-09
SIK1	salt-inducible kinase 1	NM_173354	0.98	1.04E-11	1.60	6.61E-13	1.12	4.44E-10
CCDC117	coiled-coil domain containing 117	NM_173510	0.80	1.09E-11	1.24	2.15E-12	1.47	8.43E-14
PPME1	protein phosphatase methylesterase 1	ENST00000535205	0.79	1.18E-11	0.95	2.14E-10	0.96	1.83E-10
STIP1	stress-induced phosphoprotein 1	NM_001282652	0.70	1.20E-11	0.69	6.79E-09	0.86	1.66E-10
SIX4	SIX homeobox 4	NM_017420	0.86	1.31E-11	1.55	1.40E-13	1.95	1.39E-15
RNU6-531P	RNA. U6 small nuclear 531. pseudogene	ENST00000516694	1.71	1.32E-11	1.97	5.54E-10	1.77	3.42E-09
GFPT1	glutamine--fructose-6-phosphate transaminase 1	NM_001244710	0.66	1.35E-11	0.72	1.65E-09	0.62	2.24E-08
SRXN1	sulfiredoxin 1	NM_080725	0.85	1.49E-11	0.75	5.85E-08	0.77	3.73E-08
SERPINH1	serpin peptidase inhibitor. clade H (heat shock protein 47). member 1. (collagen binding protein 1)	NM_001207014	0.56	1.55E-11	0.71	1.18E-10	0.70	1.58E-10
DNAJB6	DnaJ (Hsp40) homolog. subfamily B. member 6	NM_005494	0.77	1.56E-11	1.24	1.26E-12	1.24	1.22E-12
C18orf25	chromosome 18 open reading frame 25	NM_001008239	0.84	1.70E-11	0.99	5.25E-10	0.92	1.86E-09
MICB	MHC class I polypeptide-related sequence B	NM_001289160	0.69	1.81E-11	1.16	6.80E-13	1.06	3.71E-12
DUSP16	dual specificity phosphatase 16	NM_030640	0.68	1.86E-11	1.08	2.11E-12	1.28	9.15E-14
ABL2	ABL proto-oncogene 2. non-receptor tyrosine kinase	NM_001136000	0.66	2.19E-11	1.06	1.85E-12	1.01	4.58E-12
FGF2	fibroblast growth factor 2 (basic)	NM_002006	0.62	2.31E-11	1.01	1.68E-12	1.16	1.16E-13
F3	coagulation factor III (thromboplastin. tissue factor)	NM_001178096	1.01	2.35E-11	0.92	4.49E-08	0.92	4.56E-08
RYBP	RING1 and YY1 binding protein	NM_012234	0.88	2.39E-11	1.27	1.61E-11	1.31	9.43E-12
KDM6B	lysine (K)-specific demethylase 6B	NM_001080424	0.82	2.42E-11	0.95	8.23E-10	0.76	4.12E-08
MICB	MHC class I polypeptide-related sequence B	NM_001289161	0.71	2.62E-11	1.13	2.94E-12	1.11	4.58E-12

UBR1	ubiquitin protein ligase E3 component n-recognin 1	NM_174916	0.57	2.62E-11	0.70	3.09E-10	0.80	2.39E-11
SSH1	slingshot protein phosphatase 1	NM_001161330	0.80	3.43E-11	1.25	5.08E-12	0.77	2.78E-08
JMJD1C	jumonji domain containing 1C	NM_001282948	0.61	3.49E-11	1.44	1.99E-15	1.56	4.05E-16
MLKL	mixed lineage kinase domain-like	NM_152649	0.71	3.62E-11	1.20	1.16E-12	1.49	1.86E-14
EIF5	eukaryotic translation initiation factor 5	NM_001969	0.65	3.68E-11	0.75	1.44E-09	0.85	1.45E-10
SLFN5	schlafen family member 5	NM_144975	0.76	3.70E-11	1.27	1.48E-12	1.12	1.58E-11
GDF15	growth differentiation factor 15	NM_004864	0.67	3.73E-11	1.54	2.78E-15	1.58	1.80E-15
AZIN1	antizyme inhibitor 1	NM_015878	0.72	3.87E-11	0.93	1.99E-10	1.01	3.99E-11
HECW2	HECT. C2 and WW domain containing E3 ubiquitin protein ligase 2	NM_020760	0.71	4.08E-11	1.26	6.56E-13	1.28	4.57E-13
CCDC174	coiled-coil domain containing 174	NM_016474	0.74	4.23E-11	0.94	3.30E-10	0.92	4.67E-10
KBTBD8	kelch repeat and BTB (POZ) domain containing 8	NM_032505	0.97	5.52E-11	1.49	1.17E-11	1.81	2.85E-13
ALAS1	aminolevulinate. delta-. synthase 1	NM_000688	0.57	6.15E-11	0.55	4.82E-08	0.85	2.56E-11
ACBD3	acyl-CoA binding domain containing 3	NM_022735	0.75	6.99E-11	0.88	1.93E-09	0.86	2.97E-09
RNU6-71P	RNA. U6 small nuclear 71. pseudogene	NR_046940	2.11	7.23E-11	3.37	7.85E-12	3.14	2.98E-11
KDM2A	lysine (K)-specific demethylase 2A	NM_001256405	0.68	7.26E-11	0.99	5.07E-11	0.92	1.72E-10
TXNIP	thioredoxin interacting protein	NM_006472	-0.78	7.33E-11	-0.96	8.01E-10	-0.73	7.72E-08
KCTD5	potassium channel tetramerization domain containing 5	NM_018992	0.57	7.59E-11	0.62	7.52E-09	0.70	9.37E-10
HSPA13	heat shock protein 70kDa family. member 13	NM_006948	0.66	7.78E-11	0.70	1.22E-08	0.73	5.53E-09
BRD2	bromodomain containing 2	NM_001291986	0.52	7.94E-11	0.69	2.54E-10	0.73	8.78E-11
VEGFC	vascular endothelial growth factor C	NM_005429	0.74	8.06E-11	0.89	1.45E-09	1.03	1.22E-10
E2F7	E2F transcription factor 7	NM_203394	0.71	8.35E-11	2.20	2.21E-17	2.48	1.84E-18
MED13	mediator complex subunit 13	NM_005121	0.51	8.74E-11	0.86	3.30E-12	0.78	1.82E-11
NRIP3	nuclear receptor interacting protein 3	NM_020645	0.90	8.82E-11	1.32	4.46E-11	1.47	5.96E-12
TMEM39A	transmembrane protein 39A	NM_018266	0.58	9.47E-11	0.66	4.26E-09	0.59	2.70E-08
NA	NA	---	0.94	9.50E-11	1.19	6.98E-10	1.32	1.05E-10
SPAG9	sperm associated antigen 9	NM_001130527	0.67	1.00E-10	1.03	1.86E-11	0.92	1.52E-10
MICB	MHC class I polypeptide-related sequence B	NM_005931	0.66	1.03E-10	1.06	9.07E-12	1.08	6.13E-12
NPLOC4	nuclear protein localization 4 homolog (S. cerevisiae)	NM_017921	0.55	1.03E-10	0.73	3.90E-10	0.57	2.55E-08
CLIC2	chloride intracellular channel 2	NM_001289	1.10	1.05E-10	1.70	2.13E-11	1.93	1.82E-12
OSER1	oxidative stress responsive serine-rich 1	NM_016470	0.79	1.07E-10	1.00	9.46E-10	0.89	7.10E-09
TP53BP2	tumor protein p53 binding protein 2	NM_001031685	0.75	1.08E-10	1.02	2.25E-10	0.95	8.61E-10
BRD2	bromodomain containing 2	NM_001113182	0.49	1.16E-10	0.65	4.13E-10	0.73	4.70E-11
BRD2	bromodomain containing 2	NM_001113182	0.50	1.19E-10	0.66	3.74E-10	0.73	5.85E-11
PDE4DIP	phosphodiesterase 4D interacting protein	NM_001198832	0.85	1.25E-10	1.48	2.57E-12	1.47	2.87E-12

SPEN	spen family transcriptional repressor	NM_015001	0.70	1.27E-10	1.04	5.18E-11	0.91	6.34E-10
BRD2	bromodomain containing 2	NM_001113182	0.52	1.30E-10	0.67	5.27E-10	0.76	6.14E-11
OTULIN	OTU deubiquitinase with linear linkage specificity	NM_138348	0.74	1.31E-10	1.17	1.74E-11	1.38	8.75E-13
TSC22D2	TSC22 domain family. member 2	ENST00000361875	0.79	1.50E-10	1.17	6.80E-11	1.13	1.24E-10
BIRC2	baculoviral IAP repeat containing 2	NM_001166	0.61	1.55E-10	0.92	4.97E-11	0.75	1.86E-09
PPID	peptidylprolyl isomerase D	NM_005038	0.71	1.59E-10	0.99	1.86E-10	1.43	1.97E-13
P4HA2	prolyl 4-hydroxylase. alpha polypeptide II	ENST00000401867	0.62	1.59E-10	0.74	3.34E-09	0.74	3.77E-09
AHNAK2	AHNAK nucleoprotein 2	NM_138420	-0.55	1.61E-10	-0.89	1.44E-11	-0.60	1.62E-08
NAV3	neuron navigator 3	NM_001024383	0.57	1.62E-10	1.01	2.69E-12	1.09	5.75E-13
IRAK2	interleukin-1 receptor-associated kinase 2	NM_001570	0.90	1.63E-10	1.65	1.28E-12	1.78	2.94E-13
NOP58	NOP58 ribonucleoprotein	ENST00000264279	0.49	1.73E-10	0.72	9.14E-11	0.95	5.39E-13
OTUD7B	OTU deubiquitinase 7B	NM_020205	0.68	1.75E-10	0.72	2.46E-08	0.99	9.58E-11
HSPB1	heat shock 27kDa protein 1	NM_001540	0.48	1.82E-10	0.61	1.34E-09	0.64	5.91E-10
MORC4	MORC family CW-type zinc finger 4	NM_001085354	0.66	1.87E-10	0.75	8.07E-09	0.84	1.24E-09
TMEM217	transmembrane protein 217	NM_001162900	0.87	1.93E-10	1.67	5.53E-13	1.85	7.99E-14
IPPK	inositol 1.3.4.5.6-pentakisphosphate 2-kinase	NM_022755	0.67	1.97E-10	0.96	1.74E-10	0.85	1.32E-09
MICB	MHC class I polypeptide-related sequence B	NM_005931	0.58	2.18E-10	1.01	4.82E-12	1.01	5.75E-12
MICB	MHC class I polypeptide-related sequence B	NM_005931	0.58	2.18E-10	1.01	4.82E-12	1.01	5.75E-12
LARP4	La ribonucleoprotein domain family. member 4	NM_001170803	0.71	2.20E-10	0.81	9.24E-09	0.88	2.05E-09
HSPB1	heat shock 27kDa protein 1	NM_001540	0.51	2.21E-10	0.76	8.35E-11	0.72	2.21E-10
FEM1B	fem-1 homolog b (C. elegans)	NM_015322	0.63	2.29E-10	0.91	1.85E-10	0.84	7.38E-10
XBP1	X-box binding protein 1	NM_001079539	0.49	2.42E-10	1.05	9.60E-14	0.98	3.37E-13
DUSP1	dual specificity phosphatase 1	NM_004417	0.76	2.57E-10	1.84	1.11E-14	2.31	1.20E-16
NFKBIZ	nuclear factor of kappa light polypeptide gene enhancer in B-cells inhibitor. zeta	NM_001005474	0.84	2.64E-10	2.32	7.66E-16	2.60	8.20E-17
MICB	MHC class I polypeptide-related sequence B	NM_005931	0.62	2.78E-10	1.05	9.28E-12	1.02	1.62E-11
CYLD	cylindromatosis (turban tumor syndrome)	NM_001042355	0.60	2.94E-10	1.03	1.01E-11	1.16	9.51E-13
ZCCHC6	zinc finger. CCHC domain containing 6	NM_001185059	0.57	3.17E-10	0.91	3.44E-11	0.97	1.14E-11
ZBTB21	zinc finger and BTB domain containing 21	NM_001098402	0.79	3.33E-10	0.85	3.61E-08	0.91	1.14E-08
STX11	syntaxin 11	NM_003764	0.72	3.44E-10	1.22	1.30E-11	1.40	9.44E-13
MB21D1	Mab-21 domain containing 1	NM_138441	1.02	3.59E-10	2.02	6.35E-13	2.41	2.08E-14
UBR2	ubiquitin protein ligase E3 component n-recognin 2	NM_015255	0.47	3.60E-10	0.67	3.37E-10	0.87	2.29E-12
PTGES3	prostaglandin E synthase 3 (cytosolic)	NM_001282601	0.54	3.61E-10	0.72	9.65E-10	0.83	7.31E-11
PRKACB	protein kinase. cAMP-dependent. catalytic. beta	NM_001242857	0.58	3.88E-10	0.78	1.10E-09	1.17	5.64E-13

SLC7A2	solute carrier family 7 (cationic amino acid transporter. y+ system). member 2	NM_001008539	0.76	4.21E-10	1.37	5.33E-12	0.96	3.31E-09
FAM124B	family with sequence similarity 124B	NM_024785	-0.68	4.29E-10	-0.98	3.16E-10	-0.79	1.22E-08
TRAF6	TNF receptor-associated factor 6. E3 ubiquitin protein ligase	NM_004620	0.60	4.94E-10	1.07	6.14E-12	1.23	4.08E-13
PDE4DIP	phosphodiesterase 4D interacting protein	NM_001002811	0.63	5.31E-10	0.91	3.77E-10	1.07	1.92E-11
C3orf38	chromosome 3 open reading frame 38	NM_173824	0.60	5.36E-10	1.00	2.46E-11	1.07	7.44E-12
RND3	Rho family GTPase 3	NM_001254738	0.55	5.37E-10	0.84	1.55E-10	0.87	8.40E-11
RPS6KA3	ribosomal protein S6 kinase. 90kDa. polypeptide 3	NM_004586	0.54	6.10E-10	0.72	1.65E-09	0.73	1.08E-09
STC1	stanniocalcin 1	NM_003155	-0.56	6.17E-10	-0.94	2.78E-11	-1.00	9.56E-12
CNKSR3	CNKSR family member 3	NM_173515	0.86	6.32E-10	0.96	3.24E-08	0.94	4.66E-08
RP11-153M3.1	60 kDa heat shock protein pseudogene	OTTHUMT00000409621	0.86	6.49E-10	1.19	1.09E-09	0.97	3.60E-08
PDE4DIP	phosphodiesterase 4D interacting protein	NM_001198832	0.99	6.83E-10	1.73	1.42E-11	1.68	2.38E-11
TBC1D4	TBC1 domain family. member 4	NM_001286658	0.72	7.90E-10	0.95	2.90E-09	0.96	2.34E-09
FNIP1	folliculin interacting protein 1	NM_001008738	0.55	8.39E-10	0.70	5.64E-09	0.71	4.25E-09
ULBP1	UL16 binding protein 1	NM_025218	0.96	8.52E-10	2.12	2.11E-13	2.91	3.91E-16
NFKB1	nuclear factor of kappa light polypeptide gene enhancer in B-cells 1	NM_001165412	0.69	8.57E-10	0.99	6.40E-10	0.79	3.02E-08
RP11-762H8.4	novel transcript . sense intronic to WDR61	OTTHUMT00000471376	1.04	8.69E-10	1.31	6.51E-09	2.31	1.86E-13
ATP2C1	ATPase. Ca++ transporting. type 2C. member 1	NM_001001485	0.51	1.09E-09	0.76	4.95E-10	0.98	4.60E-12
N4BP1	NEDD4 binding protein 1	NM_153029	0.59	1.14E-09	0.73	1.37E-08	0.70	2.29E-08
SAMD4B	sterile alpha motif domain containing 4B	NM_018028	0.64	1.15E-09	1.06	6.32E-11	0.95	4.22E-10
CABLES2	Cdk5 and Abl enzyme substrate 2	NM_031215	0.70	1.24E-09	1.07	2.92E-10	1.13	1.22E-10
CCT4	chaperonin containing TCP1. subunit 4 (delta)	NM_001256721	0.46	1.24E-09	0.59	6.28E-09	0.57	1.27E-08
CNST	consortin. connexin sorting protein	NM_152609	0.73	1.42E-09	1.15	1.82E-10	1.17	1.32E-10
PI4K2B	phosphatidylinositol 4-kinase type 2 beta	NM_018323	0.68	1.47E-09	0.77	6.20E-08	0.85	1.28E-08
FXR1	fragile X mental retardation. autosomal homolog 1	NM_001013438	0.50	1.55E-09	0.69	2.19E-09	0.70	1.43E-09
TMEM47	transmembrane protein 47	NM_031442	0.47	1.58E-09	0.80	4.99E-11	0.75	1.45E-10
CHAC1	ChaC. cation transport regulator homolog 1 (E. coli)	ENST00000446533	1.01	1.68E-09	3.74	1.72E-17	3.28	2.49E-16
GPBP1	GC-rich promoter binding protein 1	NM_001203246	0.53	1.73E-09	0.94	2.41E-11	1.08	1.79E-12
MAFG	v-maf avian musculoaponeurotic fibrosarcoma oncogene homolog G	NM_032711	0.59	1.86E-09	0.77	6.59E-09	0.73	1.69E-08
GBP3	guanylate binding protein 3	NM_018284	0.63	2.12E-09	0.72	6.85E-08	0.74	4.94E-08
CCDC59	coiled-coil domain containing 59	NM_014167	0.50	2.17E-09	0.86	7.63E-11	0.74	1.16E-09
MICA	MHC class I polypeptide-related sequence A	ENST00000415525	0.53	2.47E-09	0.83	3.46E-10	0.80	7.01E-10

KCTD12	potassium channel tetramerization domain containing 12	NM_138444	-0.64	2.55E-09	-1.25	7.53E-12	-1.08	1.10E-10
MICB	MHC class I polypeptide-related sequence B	ENST00000458032	0.57	2.57E-09	0.95	1.20E-10	0.98	7.09E-11
JMJD6	jumonji domain containing 6	NM_001081461	0.59	2.88E-09	0.78	8.85E-09	1.21	3.00E-12
GBE1	glucan (1.4-alpha-). branching enzyme 1	ENST00000429644	0.52	3.31E-09	1.03	7.71E-12	1.20	3.85E-13
SNAI1	snail family zinc finger 1	NM_005985	0.79	3.35E-09	1.84	3.73E-13	1.54	1.20E-11
IGHJ1	immunoglobulin heavy joining 1	ENST00000390565	-1.44	3.45E-09	-1.76	3.67E-08	-2.34	2.79E-10
RNU6-329P	RNA. U6 small nuclear 329. pseudogene	ENST00000459618	1.59	3.51E-09	2.20	5.18E-09	2.82	6.16E-11
RAB39A	RAB39A. member RAS oncogene family	NM_017516	1.21	3.78E-09	2.32	1.56E-11	2.53	3.03E-12
CEBPG	CCAAT/enhancer binding protein (C/EBP). gamma	NM_001252296	0.59	3.90E-09	1.59	2.23E-14	1.41	2.28E-13
EDA2R	ectodysplasin A2 receptor	NM_001199687	0.69	3.95E-09	1.18	1.26E-10	1.03	1.42E-09
HSPB1P1	heat shock 27kDa protein 1 pseudogene 1	ENST00000423240	0.37	3.96E-09	0.53	3.79E-09	0.54	2.28E-09
RGS2	regulator of G-protein signaling 2	NM_002923	0.64	4.20E-09	1.23	1.67E-11	1.48	4.72E-13
CPA4	carboxypeptidase A4	NM_001163446	-0.65	4.33E-09	-1.23	2.31E-11	-0.99	1.22E-09
LOC100128233	uncharacterized LOC100128233	NR_103769	0.94	4.35E-09	2.15	6.61E-13	2.66	1.05E-14
BRPF3	bromodomain and PHD finger containing. 3	NM_015695	0.68	4.66E-09	0.97	3.69E-09	1.00	2.33E-09
FAM219A	family with sequence similarity 219. member A	NM_001184940	0.69	5.19E-09	1.14	3.65E-10	1.14	3.54E-10
MERTK	MER proto-oncogene. tyrosine kinase	NM_006343	0.49	5.20E-09	0.97	1.09E-11	1.02	4.01E-12
SNAP23	synaptosomal-associated protein. 23kDa	NM_003825	0.41	5.26E-09	0.71	1.90E-10	0.80	2.06E-11
ANXA1	annexin A1	NM_000700	0.43	5.67E-09	0.89	6.71E-12	0.87	9.62E-12
CSRNP1	cysteine-serine-rich nuclear protein 1	NM_033027	0.76	5.94E-09	1.30	1.91E-10	1.25	3.90E-10
DYRK3	dual-specificity tyrosine-(Y)-phosphorylation regulated kinase 3	ENST00000367106	0.53	6.04E-09	0.92	1.92E-10	0.95	9.77E-11
TUBE1	tubulin. epsilon 1	NM_016262	0.57	6.14E-09	1.22	3.75E-12	1.60	2.08E-14
PDE4DIP	phosphodiesterase 4D interacting protein	NM_001198834	0.86	6.52E-09	1.18	1.10E-08	1.17	1.43E-08
SDPR	serum deprivation response	NM_004657	-0.61	6.68E-09	-1.02	3.37E-10	-0.75	6.42E-08
CTH	cystathionine gamma-lyase	ENST00000346806	1.01	7.56E-09	1.52	2.68E-09	1.27	5.98E-08
IFRD1	interferon-related developmental regulator 1	NM_001007245	0.64	7.77E-09	0.96	2.49E-09	0.99	1.50E-09
ALDH2	aldehyde dehydrogenase 2 family (mitochondrial)	NM_000690	0.48	8.60E-09	0.73	2.33E-09	0.70	4.42E-09
CEBPB	CCAAT/enhancer binding protein (C/EBP). beta	NM_001285878	0.68	8.96E-09	1.40	1.21E-11	0.97	8.37E-09
GULP1	GULP. engulfment adaptor PTB domain containing 1	NM_001252668	0.62	9.21E-09	1.03	5.61E-10	0.80	3.99E-08
CPT1A	carnitine palmitoyltransferase 1A (liver)	NM_001031847	-0.49	9.69E-09	-0.78	1.14E-09	-0.69	9.64E-09
NFIB	nuclear factor I/B	NM_001190737	-0.42	9.82E-09	-0.55	3.14E-08	-0.55	3.90E-08
ADAMTS18	ADAM metallopeptidase with thrombospondin type 1 motif. 18	NM_199355	-0.58	1.05E-08	-1.22	9.46E-12	-1.03	2.27E-10

HBEGF	heparin-binding EGF-like growth factor	NM_001945	0.49	1.10E-08	1.02	1.35E-11	1.20	6.46E-13
SORBS1	sorbin and SH3 domain containing 1	NM_001034954	0.93	1.15E-08	1.67	1.57E-10	1.72	9.56E-11
ZNF317	zinc finger protein 317	NM_001190791	0.54	1.28E-08	0.73	3.35E-08	0.84	2.60E-09
EFCAB7	EF-hand calcium binding domain 7	NM_032437	0.84	1.29E-08	1.15	2.06E-08	1.11	3.90E-08
SLC7A5	solute carrier family 7 (amino acid transporter light chain. L system). member 5	NM_003486	0.69	1.39E-08	2.39	7.59E-16	2.69	7.18E-17
42434	membrane-associated ring finger (C3HC4) 5	ENST00000467521	0.43	1.45E-08	0.62	9.46E-09	0.62	1.17E-08
RP4-791M13.3	novel transcript	OTTHUMT00000384603	0.81	1.55E-08	1.35	8.35E-10	1.28	2.06E-09
NA	NA	---	0.65	1.61E-08	1.05	1.85E-09	1.04	2.02E-09
SLC5A3	solute carrier family 5 (sodium/myo-inositol cotransporter). member 3	NM_006933	0.60	1.67E-08	0.93	3.07E-09	1.23	2.16E-11
TRIB3	tribbles pseudokinase 3	uc002wdn.3	0.83	1.68E-08	1.68	3.13E-11	1.92	2.60E-12
RASSF8	Ras association (RalGDS/AF-6) domain family (N-terminal) member 8	NM_001164746	0.56	1.69E-08	0.90	1.98E-09	1.19	1.22E-11
ATG5	autophagy related 5	NM_001286106	0.50	1.70E-08	0.78	3.03E-09	0.78	2.85E-09
GXYLT2	glucoside xylosyltransferase 2	NM_001080393	1.08	1.76E-08	1.98	1.95E-10	2.66	7.69E-13
ZNF267	zinc finger protein 267	NM_001265588	0.64	1.78E-08	0.86	4.61E-08	0.95	8.52E-09
TULP3	tubby like protein 3	NM_001160408	0.40	2.08E-08	0.78	7.17E-11	0.59	1.02E-08
MICA	MHC class I polypeptide-related sequence A	ENST00000400325	0.53	2.16E-08	0.92	7.06E-10	0.85	2.47E-09
MICA	MHC class I polypeptide-related sequence A	ENST00000400325	0.53	2.16E-08	0.92	7.06E-10	0.85	2.47E-09
STXBP5-AS1	STXBP5 antisense RNA 1	NR_034115	0.56	2.46E-08	1.46	3.28E-13	2.12	2.15E-16
NA	NA	---	0.86	2.48E-08	1.17	4.42E-08	1.51	6.42E-10
KANSL1L	KAT8 regulatory NSL complex subunit 1-like	NM_152519	0.61	3.32E-08	1.21	1.08E-10	0.89	2.31E-08
TYW3	tRNA-yW synthesizing protein 3 homolog (S. cerevisiae)	NM_001162916	0.62	3.52E-08	0.98	4.88E-09	1.12	4.87E-10
LURAP1L	leucine rich adaptor protein 1-like	NM_203403	0.65	3.81E-08	0.95	2.48E-08	1.14	1.12E-09
NXT2	nuclear transport factor 2-like export factor 2	NM_001242617	0.63	3.97E-08	1.11	9.77E-10	1.73	2.38E-13
SLC37A1	solute carrier family 37 (glucose-6-phosphate transporter). member 1	NM_018964	0.50	4.10E-08	0.71	3.51E-08	0.83	2.29E-09
ABCB1	ATP-binding cassette. sub-family B (MDR/TAP). member 1	NM_000927	0.45	4.40E-08	0.69	1.23E-08	0.62	7.20E-08
ULBP3	UL16 binding protein 3	NM_024518	0.87	4.44E-08	1.31	1.55E-08	1.78	7.47E-11
DNAJC24	DnaJ (Hsp40) homolog. subfamily C. member 24	ENST00000526042	0.57	4.92E-08	0.98	2.06E-09	1.10	2.63E-10
IPMK	inositol polyphosphate multikinase	NM_152230	0.60	5.30E-08	1.06	1.19E-09	1.01	2.41E-09
NRBF2	nuclear receptor binding factor 2	NM_001282405	0.51	5.35E-08	0.75	2.85E-08	0.74	3.56E-08
SLC3A2	solute carrier family 3 (amino acid transporter heavy chain). member 2	NM_001012662	0.52	5.39E-08	1.18	1.39E-11	1.14	2.52E-11
SETD5	SET domain containing 5	NM_001080517	0.38	5.54E-08	0.76	1.16E-10	0.58	1.26E-08

ELL	elongation factor RNA polymerase II	NM_006532	0.66	5.99E-08	1.09	4.43E-09	1.11	2.99E-09
STXBP5-AS1	STXBP5 antisense RNA 1	ENST00000433308	0.71	6.13E-08	2.01	2.22E-13	1.66	8.67E-12
LRIG1	leucine-rich repeats and immunoglobulin-like domains 1	ENST00000273261	0.41	7.26E-08	0.70	2.99E-09	0.67	6.26E-09
GTPBP2	GTP binding protein 2	NM_001286216	0.56	7.47E-08	0.93	5.46E-09	1.15	1.28E-10
HERC4	HECT and RLD domain containing E3 ubiquitin protein ligase 4	NM_001278185	0.45	7.73E-08	0.64	6.49E-08	0.89	2.29E-10
ERRFI1	ERBB receptor feedback inhibitor 1	ENST00000467067	0.38	8.01E-08	1.01	1.11E-12	0.68	1.66E-09

NA; not applicable. FC; fold change. n= 6 for 12 h, *n*= 3 for 18 h and 24 h.

Table S5. UPR components transcriptome expression in HAEC after infection with GFP and NOX5-β adenoviruses

	12 h			18 h			24 h		
	GFP	NOX5-β	*p*-value	GFP	NOX5-β	*p*-value	GFP	NOX5-β	*p*-value
**ATF6**	10.24± 0.04	10.39± 0.01	0.0004	10.20± 0.02	10.54± 0.02	<0.0001	10.15 ±0.02	10.43± 0.02	<0.0001
**IRE1α**	7.15± 0.05	8.47± 0.06	<0.0001	7.11± 0.02	9.35± 0.06	<0.0001	7.16± 0.07	9.28± 0.01	<0.0001
**PERK**	7.27± 0.03	7.75± 0.03	<0.0001	7.25± 0.02	7.60± 0.05	<0.0001	7.79± 0.03	7.54± 0.05	0.0021
**CHOP**	5.12± 0.12	7.04± 0.06	<0.0001	5.02± 0.08	7.11± 0.06	<0.0001	5.19± 0.06	7.01± 0.06	<0.0001
**BIP**	12.36± 0.02	13.19± 0.01	<0.0001	12.18± 0.0	13.24± 0.02	<0.0001	12.49 ±0.02	13.18± 0.02	<0.0001
**XBP1**	8.72± 0.02	9.21± 0.02	<0.0001	8.36± 0.02	9.41± 0.01	<0.0001	8.40± 0.06	9.39± 0.01	<0.0001
**ATF4**	9.57± 0.01	9.67± 0.03	0.0247	9.15± 0.04	9.84± 0.02	<0.0001	9.00± 0.04	9.89± 0.03	<0.0001
**EIF2α**	9.76± 0.05	9.91± 0.03	0.006	9.54± 0.03	9.74± 0.01	0.0102	9.62± 0.03	9.74± 0.01	0.157
**Calnexin**	11.47± 0.02	11.67± 0.03	<0.0001	11.38± 0.02	11.65± 0.03	<0.0001	11.49 ±0.01	11.58± 0.03	0.0866
**HSP90**	7.76± 0.04	8.65± 0.01	<0.0001	7.79± 0.06	8.71± 0.02	<0.0001	7.81± 0.03	8.71± 0.04	0.0001

Results expressed as mean ± SEM. *n*= 6 for 12 h, *n*= 3 for 18 h and 24 h.

Table S6. NOX family transcriptome expression in HAEC cells after infection with GFP and NOX5-β adenoviruses.

	12 h			18 h			24 h		
	GFP	NOX5-β	*p*-value	GFP	NOX5-β	*p*-value	GFP	NOX5-β	*p*-value
**NOX1**	4.44± 0.07	4.47± 0.11	0.8649	4.50± 0.12	4.72± 0.11	0.5589	4.30± 0.11	4.31± 0.03	>0.999
**NOX2**	4.32± 0.09	4.40± 0.05	0.9023	4.23± 0.08	4.55± 0.11	0.072	4.39± 0.02	4.47± 0.11	0.899
**NOX3**	4.49± 0.09	4.57± 0.07	0.8304	4.56± 0.10	4.62± 0.03	0.9747	4.69± 0.18	4.61± 0.13	0.952
**NOX4**	7.82± 0.04	7.57± 0.03	0.0087	7.35± 0.07	7.03± 0.09	0.4524	7.35± 0.05	6.98± 0.07	0.058
**NOX5**	7.64± 0.09	8.72± 0.07	<0.0001	7.92± 0.27	8.72± 0.13	0.0018	7.82± 0.10	8.67± 0.05	0.001
**DUOX1**	5.06± 0.08	4.83± 0.07	0.0493	4.94± 0.07	5.08± 0.09	0.6218	5.01± 0.04	4.94± 0.06	0.934
**DUOX2**	4.85± 0.09	4.91± 0.06	0.9408	4.93± 0.05	4.81± 0.08	0.8297	4.86± 0.12	4.84± 0.10	0.999

Results expressed as mean ± SEM. *n*= 6 for 12 h, *n*= 3 for 18 h and 24 h.
